# Supplementary material for: Analytical and computational workflow for in-depth analysis of oxidized complex lipids in blood plasma
Source: Nat Commun. 2022 Nov 1;13:6547. doi: 10.1038/s41467-022-33225-9 (PMC9626469; doi:10.1038/s41467-022-33225-9)
Supplement: Supplementary file 1 — Supplementary Information [file 41467_2022_33225_MOESM1_ESM.pdf]

Supplementary Information

Analytical and computational workflow for in-depth analysis of oxidized complex lipids in blood plasma

Angela Criscuolo[1,2,3,#], Palina Nepachalovich[1,2,4#], Diego Fernando Garcia-del Rio[1,2], Mike Lange[1,2], Zhixu Ni[1,2,4], Massimo Baroni[5], Gabriele Cruciani[6], Laura Goracci[6], Matthias Blüher[7,8], Maria Fedorova[1,2, 4,*]

[1]Institute of Bioanalytical Chemistry, Faculty of Chemistry and Mineralogy, University of Leipzig, 04013 Leipzig, Germany; [2]Center for Biotechnology and Biomedicine, University of Leipzig, 04013 Leipzig, Germany; [3]Thermo Fisher Scientific, 63303 Dreieich, Germany; [4]Present address: Center of Membrane Biochemistry and Lipid Research, Faculty of Medicine Carl Gustav Carus of TU Dresden, 01307 Dresden, Germany; [5]Molecular Discovery, Kinetic Business Centre, Borehamwood, WD6 4PJ Hertfordshire, UK; [6]Department of Chemistry, Biology and Biotechnology, University of Perugia, 06123 Perugia, Italy; [7]Medical Department III (Endocrinology, Nephrology and Rheumatology), University of Leipzig, 04103 Leipzig, Germany; [8]Helmholtz Institute for Metabolic, Obesity and Vascular Research (HI-MAG) of the Helmholtz Zentrum München at the University of Leipzig and University Hospital Leipzig, 04103 Leipzig, Germany. email: maria.fedorova@tu-dresden.de.
These authors contributed equally: Angela Criscuolo, Palina Nepachalovich

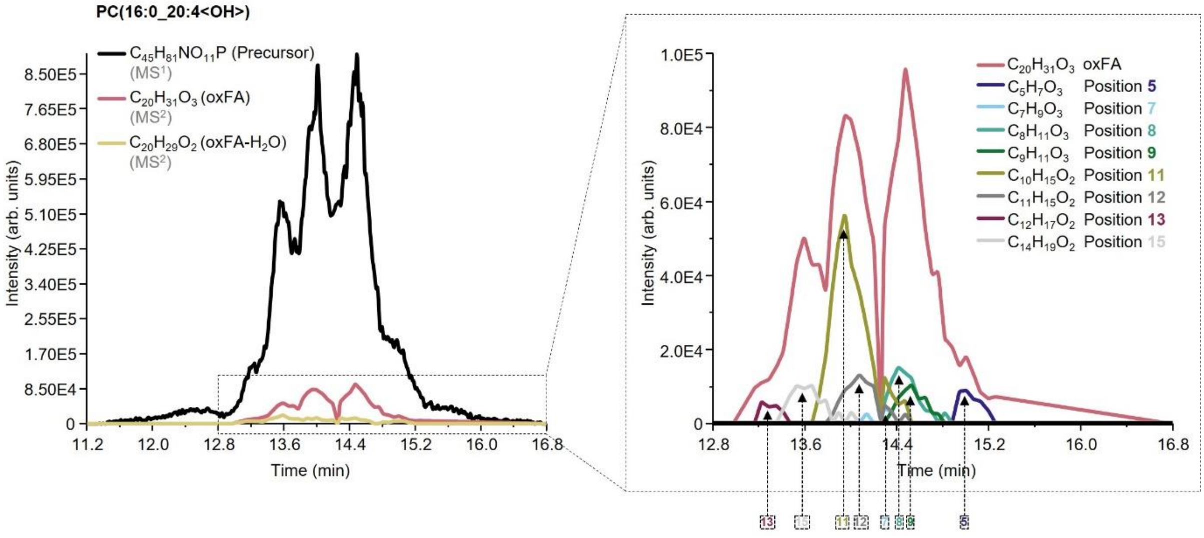

Supplementary Figure 1. Extracted ion chromatograms (XICs) for PC(16:0_20:4<OH>) in negative ion mode demonstrating elution order of different regioisomers. XIC of PC(16:0_20:4<OH>) precursor ion plotted from MS1 data is shown in black. Signals of deprotonated FA20:4<OH> (oxFA) and FA20:4<OH>-H_2O (oxFA-H_2O) are extracted from the corresponding MS2 spectra and depicted as red and orange XICs, respectively. Zoom in showing regioisomers specific MS2 fragment ion XICs. OxFA XIC is depicted in red, position-specific fragment for FA20:4<OH{5}> in dark blue, for FA20:4<OH{7}> in light blue, for FA20:4<OH{8}> in olive, for FA20:4<OH{9}> in pink, for FA20:4<OH{11}> in green, for FA20:4<OH{12}> in grey, for FA20:4<OH{13}> in yellow, and for FA20:4<OH{15}> in purple.

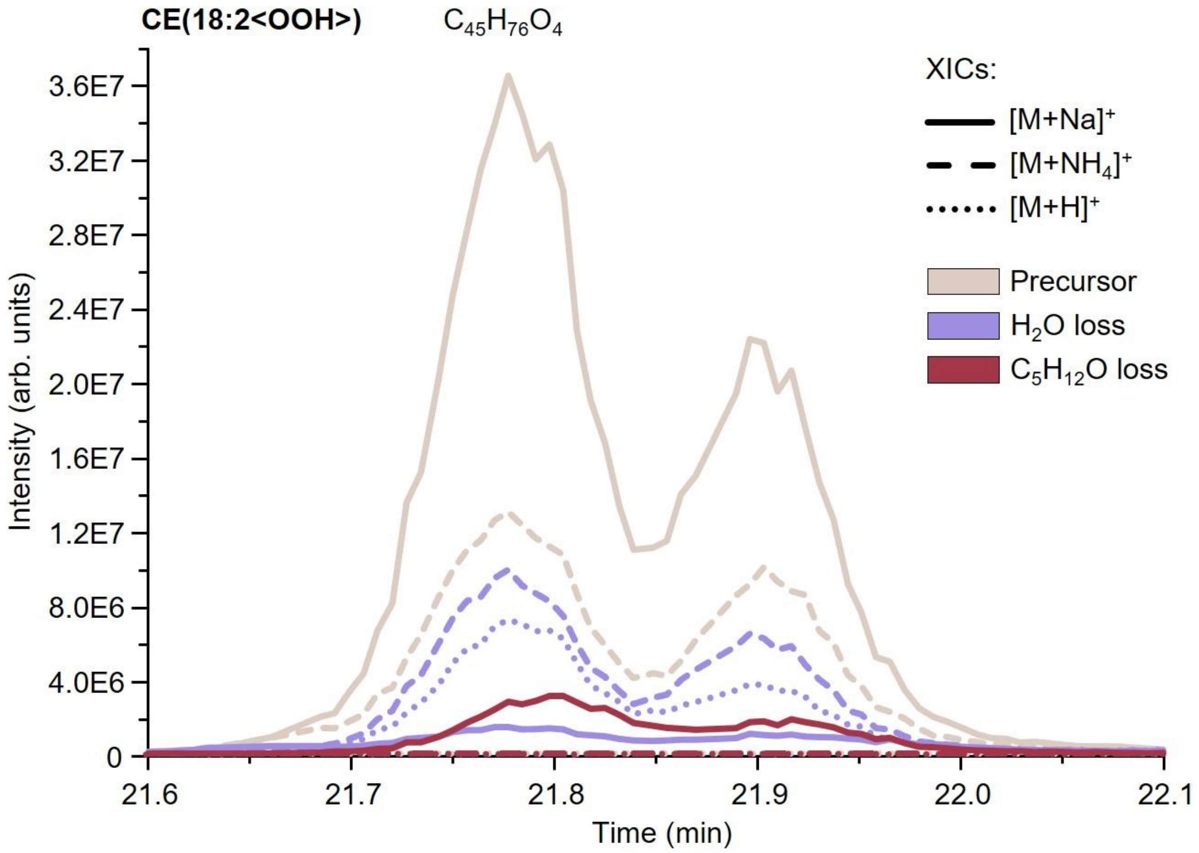

Supplementary Figure 2. Extracted ion chromatograms (XICs) of CE(18:2<OOH>) ionized as sodiated (solid XICs), ammoniated (dash-dot XICs), and protonated (dot XICs) species and corresponding in-source fragments. Precursor ions of all three adduct types are in grey, and in-source fragments corresponding to water loss and acyl chain truncation are shown in purple and pink, respectively.

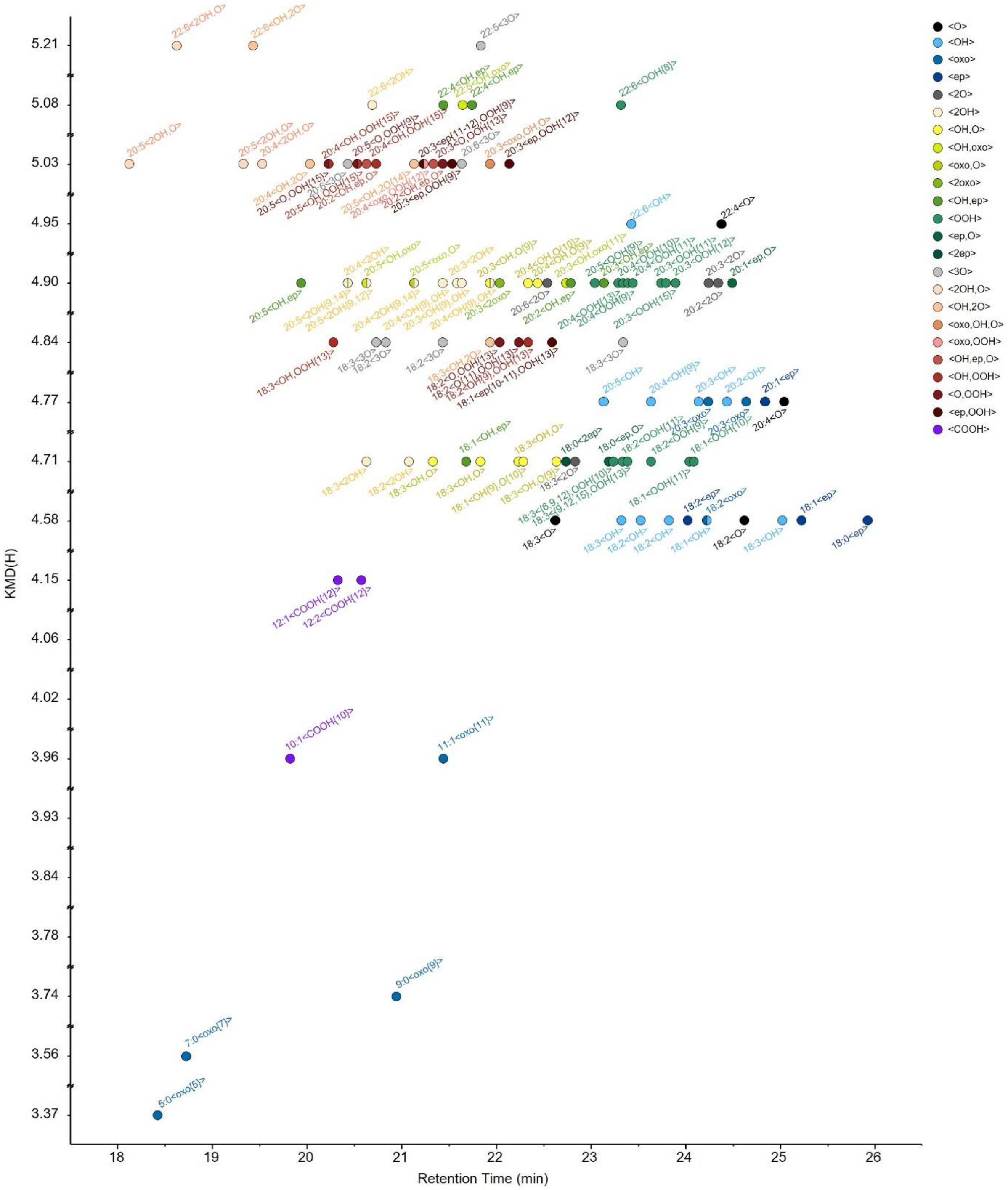

Supplementary Figure 3. KMD(H) vs retention time plot for annotation of oxCE lipids identified in blood plasma lipid extracts of obese non-diabetic and obese with type 2 diabetes individuals. Source data are provided as a Source Data file.

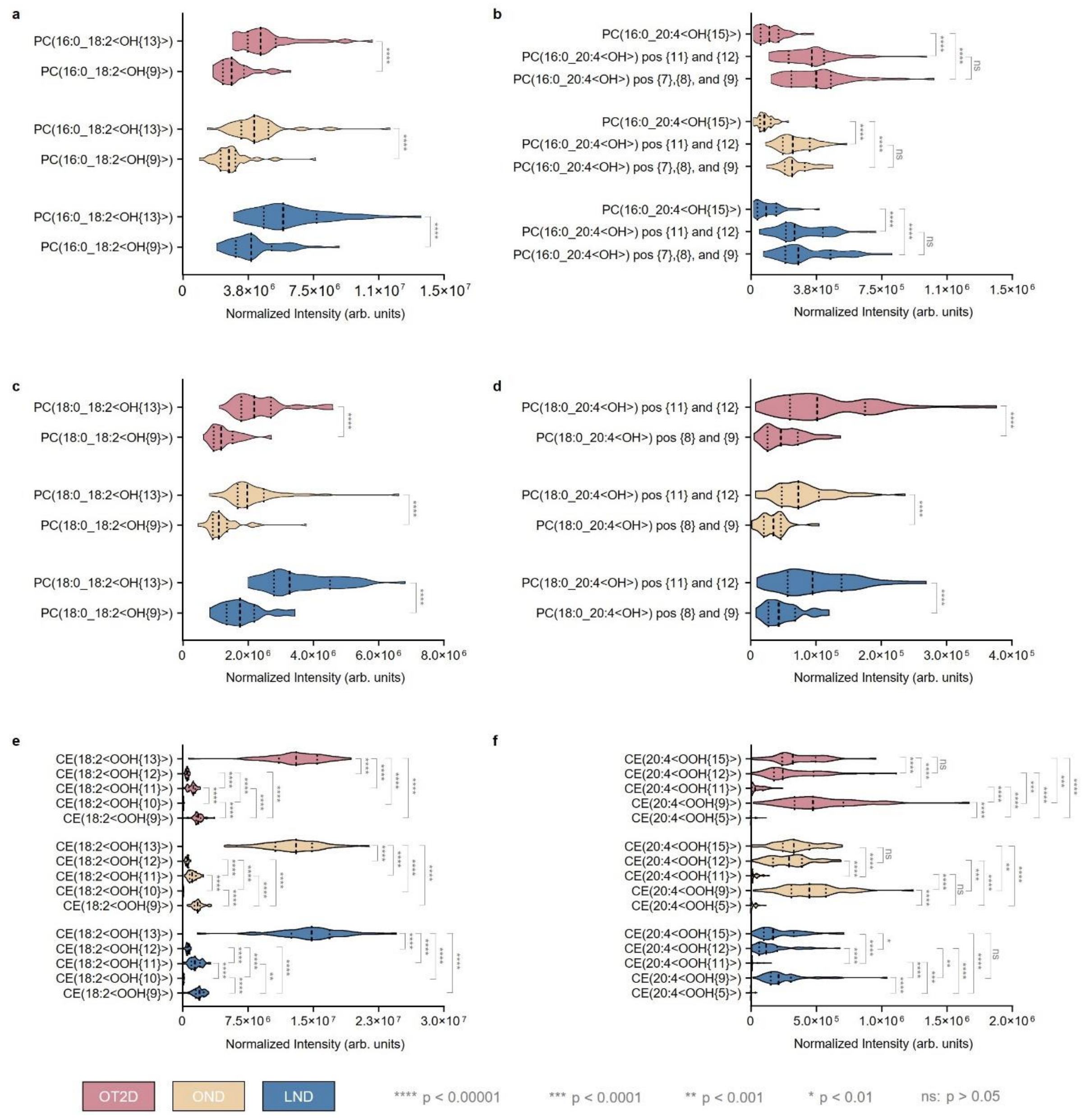

Supplementary Figure 4. Comparison of abundances of regioisomeric hydroxylated PC (a - PC(16:0_18:2<OH>), b - PC(16:0_20:4<OH>), c - PC(18:0_18:2<OH>), d - PC(18:0_20:4<OH>)) and CE hydroperoxides (e – CE(18:2<OOH>), f – CE(20:4<OOH>)). Species were quantified by PRM method using "Precursor → oxFA" and "Precursor → Position-specific fragment" transitions for oxPC and oxCE, correspondingly, in lean non-diabetic (LND, blue), obese non-diabetic (OND, yellow), and obese with type 2 diabetes (OT2D, pink) groups. Each group comprises 50 individual samples except LND for oxPCs (N = 49). Truncated violin plots represent kernel density, median values as dashed lines in the center, and interquartile ranges (dotted lines) with the 1st quartile (25th percentile) and 3rd quartile (75th percentile) to the left and right of the median values, respectively. Two-tailed t tests (unpaired, parametric, the same SD in both populations assumed) with confidence interval of 95% ($p < 0.05$) were performed for oxPCs and oxCEs in each sample group. Source data (including t test results with exact p-values) are provided as a Source Data file.

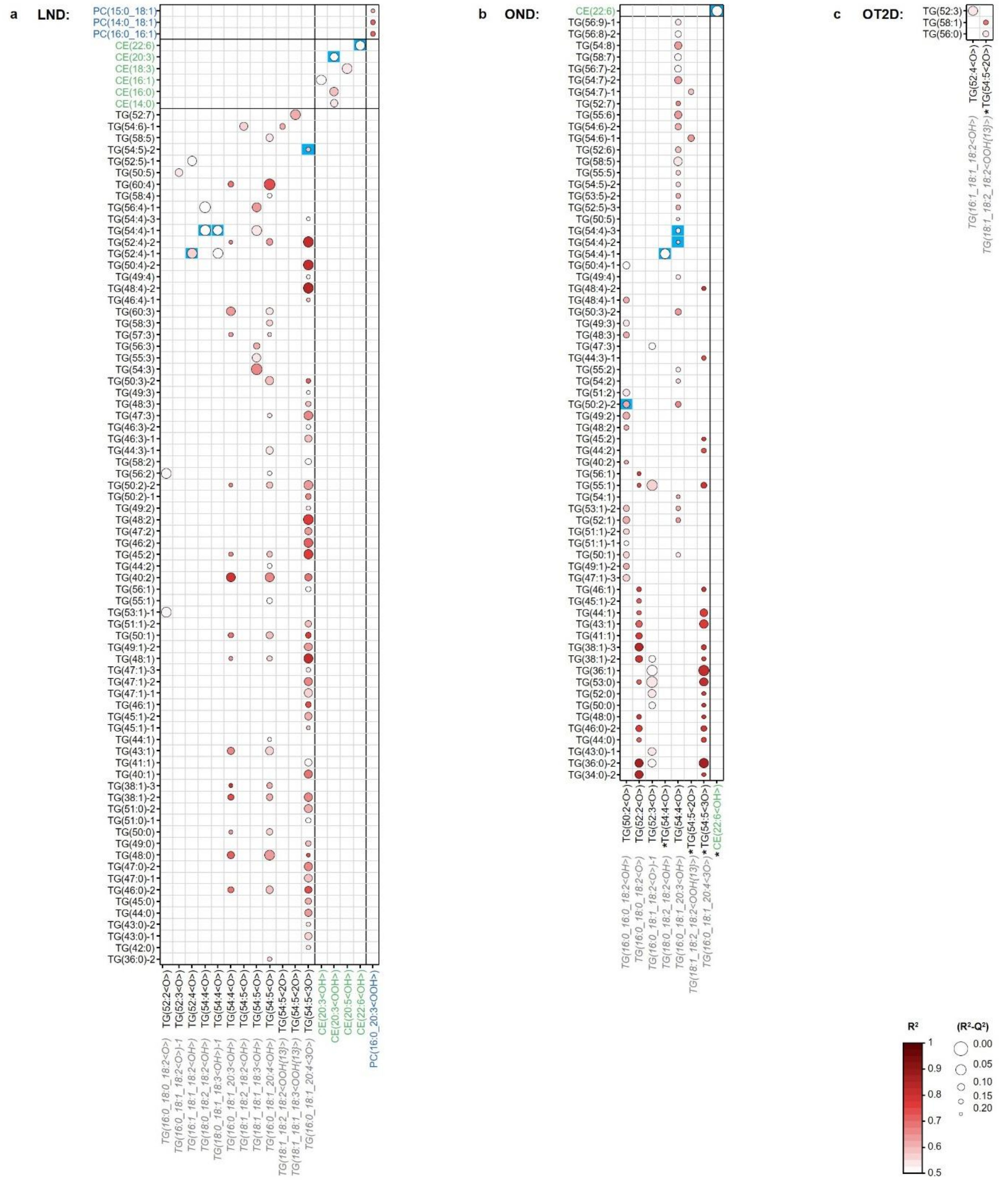

Supplementary Figure 5. Bubble plots representing correlation coefficients (R2, bubble colour) and the robustness of correlation (R2-Q2, bubble size) for "lipid-oxidized lipid" pairs in lean non-diabetic (LND, A), obese non-diabetic (OND, B), and obese with type 2 diabetes (OT2D, C) groups. Only datapoints with R2 ≥ 0.5, (R2-Q2) ≤ 0.2 are considered as statistically significant correlations and plotted. Correlations of oxidized complex lipids with the corresponding (potential) lipid substrates are highlighted in blue. Oxidized species showing statistically significant upregulation in a given group compared to the other two are marked with asterisks. Source data are provided as a Source Data file.
