# Supplementary material for: Analytical and computational workflow for in-depth analysis of oxidized complex lipids in blood plasma
Source: Nat Commun. 2022 Nov 1;13:6547. doi: 10.1038/s41467-022-33225-9 (PMC9626469; doi:10.1038/s41467-022-33225-9)
Supplement: Supplementary file 3 — Description of Additional Supplementary Files [file 41467_2022_33225_MOESM3_ESM.pdf]

**Description of Additional Supplementary Files**

File name: Supplementary Data 1
Description: List of annotated oxylipins standards (1.1), *in vitro* oxPC (1.2), oxCE (1.3) and oxTG (1.4) with corresponding IDs at fatty acyl, modification type and modification position levels. Neutral/ion formula, theoretical and measured mass, detected adducts and their *m/z* as well as all detected structure specific fragments are listed.

File name: Supplementary Data 2
Description: MS2 fragmentation patterns for oxidatively truncated and full-length oxygenated lipids.

File name: Supplementary Data 3
Description: List of annotated oxPC (3.1), oxCE (3.2) and oxTG (3.3) in *in vitro* oxidized human blood plasma with corresponding IDs at fatty acyl, modification type and modification position levels. Neutral/ion formula, theoretical and measured mass, detected adducts and their *m/z* as well as all detected structure specific fragments are listed.

File name: Supplementary Data 4
Description: List of human blood plasma PUFA-containing PC, CE and TG molecular species used for *in silico* oxidation.

File name: Supplementary Data 5
Description: List of annotated oxPC (5.1 for OND and 5.2 for OT2D pools), oxCE (5.3 for OND and 5.4 for OT2D pools) and oxTG (5.5 for OND and 5.6 for OT2D pools) in human blood plasma of obese non-diabetic (OND) and obese diabetic (OT2D) individulas with corresponding IDs at fatty acyl, modification type and modification position levels. Neutral/ion formula, theoretical and measured mass, detected adducts and their *m/z* as well as all detected structure specific fragments are listed.

File name: Supplementary Data 6
Description: MS2 spectra acquired using stDDA and used for the annotation of oxidized PC (oxPC) species in group-pooled blood plasma samples of obese non-diabetic (OND) and obese with type 2 diabetes (OT2D) individuals. Structure-related fragmentation ions are colour-coded according to the legend provided. Annotated lipids for each group pool are sorted by their precursor *m/z*.

File name: Supplementary Data 7
Description: MS2 spectra acquired using stDDA and used for the annotation of oxidized CE (oxCE) species in group-pooled blood plasma samples of obese non-diabetic (OND) and obese with type 2 diabetes (OT2D) individuals. Structure-related fragmentation ions are colour-coded according to the legend provided. Annotated lipids for each group pool are sorted by their precursor *m/z*.

File name: Supplementary Data 8
Description: MS2 spectra acquired using stDDA and used for the annotation of oxidized TG (oxTG) species in group-pooled blood plasma samples of obese non-diabetic (OND) and obese with type 2 diabetes (OT2D) individuals. Structure-related fragmentation ions are colour-coded according to the legend provided. Annotated lipids for each group pool are sorted by their precursor *m/z*.

File name: Supplementary Data 9
Description: Normalized intensities for PRM quantified oxidized complex lipids in individual blood plasma lipid extracts of lean non-diabetic (LND), obese non-diabetic (OND), and obese with type II diabetes (OT2D) individuals.

File name: Supplementary Data 10
Description: List of oxPC (10.1), oxCE (10.2) and oxTG (10.3) species showed significant differences (ANOVA < 0.05) between the groups upon targeted PRM based quantification.

File name: Supplementary Data 11
Description: Annotation of the raw MS data files uploaded to MassIVE MSV000088608.
