# Supplementary material for: Analytical and computational workflow for in-depth analysis of oxidized complex lipids in blood plasma
Source: Nat Commun. 2022 Nov 1;13:6547. doi: 10.1038/s41467-022-33225-9 (PMC9626469; doi:10.1038/s41467-022-33225-9)
Supplement: Supplementary file 5 — Supplementary Data 2 [file 41467_2022_33225_MOESM5_ESM.pdf]

Supplementary Data 2. MS^2 fragmentation patterns for oxidatively truncated and full-length oxygenated lipids.

Table of Contents

PHOSPHATIDYLCHOLINES 2
Oxidatively truncated: <COOH> 2
Oxidatively truncated: <oxo> 3
Full-length oxygenated: <oxo> 4
Full-length oxygenated: <OH> 6
Full-length oxygenated: <ep> 8
Full-length oxygenated: <OOH> 9
CHOLESTERYL ESTERS 11
Oxidatively truncated: <COOH> 11
Oxidatively truncated: <oxo> 12
Full-length oxygenated: <oxo> 13
Full-length oxygenated: <OH> 14
Full-length oxygenated: <ep> 15
Full-length oxygenated: <OOH> 16
TRIACYLGLYCEROLS 18
Oxidatively truncated: <COOH> 18
Oxidatively truncated: <oxo> 19
Full-length oxygenated: <oxo> 20
Full-length oxygenated: <OH> 21
Full-length oxygenated: <ep> 22
Full-length oxygenated: <OOH> 23
REFERENCES 25

General notes:

1. All *m/z* values given in the table were calculated considering the charge.
2. Representative MS^2 spectra do not always contain signals corresponding to the position-specific fragments (the latter were acquired in MS^3 experiments).
3. For simplicity, only *E*-conformers of PUFAs are depicted. Fragmentation patterns do not allow to elucidate the configuration of a double bond, but keep in mind the natural *Z*-configuration for some double bonds in (ox)PUFAs.
4. Modification- and position-specific fragments: the most intense ones are highlighted in **bold**, the intermediate intensity ones are regular, and the weak ones are grayed out.

PHOSPHATIDYLCHOLINES

Oxidatively truncated: <COOH>

[M-H]$^-$ adducts (precursors)

Mod. position	Precursor	Chemical Formula	*m/z*	MS^2 Fragmentation	Mod./pos.-specific fragment[1]	Chemical Formula	*m/z*
4	PC(16:0/4:0<COOH>)	$C_{28}H_{53}O_{10}NP$	594.3413	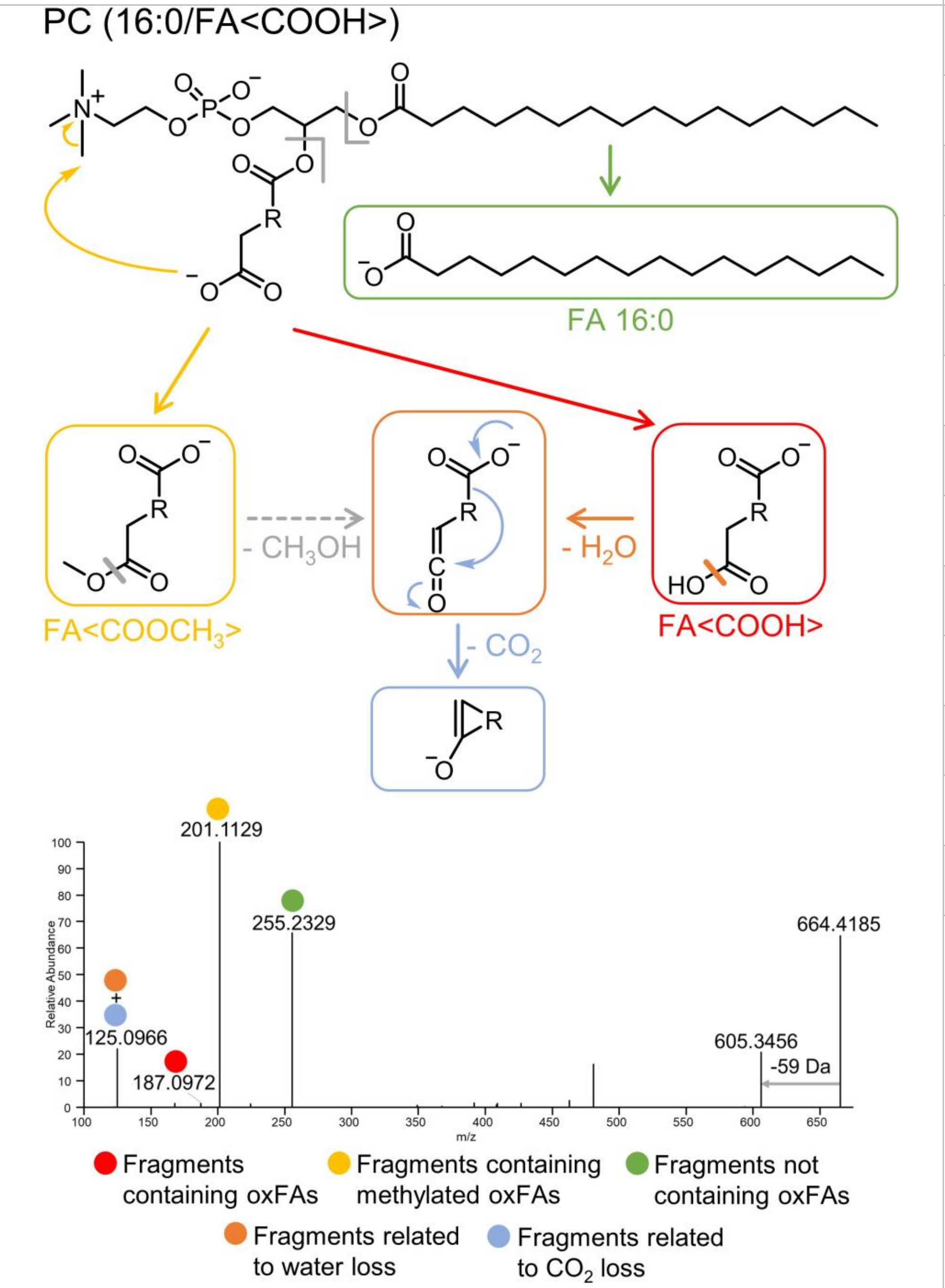	FA(4:0<COOCH$_3$>)	$C_5H_7O_4$	131.0350
5	PC(16:0/5:0<COOH>)	$C_{29}H_{55}O_{10}NP$	608.3569		FA(5:0<COOCH$_3$>)	$C_6H_9O_4$	145.0506
7	PC(16:0/7:0<COOH>)	$C_{31}H_{59}O_{10}NP$	636.3882		FA(7:0<COOCH$_3$>)	$C_8H_{13}O_4$	173.0819
8	PC(16:0/8:0<COOH>)	$C_{32}H_{61}O_{10}NP$	650.4039		FA(8:0<COOCH$_3$>)	$C_9H_{15}O_4$	187.0976
9	PC(16:0/9:0<COOH>)	$C_{33}H_{63}O_{10}NP$	664.4195		FA(9:0<COOCH3>)	$C_{10}H_{17}O_4$	201.1132
10	PC(16:0/10:0<COOH>)	$C_{34}H_{65}O_{10}NP$	678.4352		FA(10:0<COOCH$_3$>)	$C_{11}H_{19}O_4$	215.1289
	PC(16:0/10:1<COOH>)	$C_{34}H_{63}O_{10}NP$	676.4195		FA(10:1<COOCH$_3$>)	$C_{11}H_{17}O_4$	213.1132
11	PC(16:0/11:1<COOH>)	$C_{35}H_{65}O_{10}NP$	690.4352		FA(11:1<COOCH$_3$>)	$C_{12}H_{19}O_4$	227.1289
	PC(16:0/11:2<COOH>)	$C_{35}H_{63}O_{10}NP$	688.4195		FA(11:2<COOCH$_3$>)	$C_{12}H_{17}O_4$	225.1132
12	PC(16:0/12:1<COOH>)	$C_{36}H_{67}O_{10}NP$	704.4508		FA(12:1<COOCH$_3$>)	$C_{13}H_{21}O_4$	241.1445
	PC(16:0/12:2<COOH>)	$C_{36}H_{65}O_{10}NP$	702.4352		FA(12:2<COOCH$_3$>)	$C_{13}H_{19}O_4$	239.1289
13	PC(16:0/13:2<COOH>)	$C_{37}H_{67}O_{10}NP$	716.4508		FA(13:2<COOCH$_3$>)	$C_{14}H_{21}O_4$	253.1445
	PC(16:0/13:3<COOH>)	$C_{37}H_{65}O_{10}NP$	714.4352		FA(13:3<COOCH$_3$>)	$C_{14}H_{19}O_4$	251.1289
14	PC(16:0/14:3<COOH>)	$C_{38}H_{67}O_{10}NP$	728.4508		FA(14:3<COOCH$_3$>)	$C_{15}H_{21}O_4$	265.1445

[1] Although other characteristic fragments can be formed occasionally, "Mod./pos.-specific fragment" column contains the most reproducible fragment ion *m/z*. Such fragments as FA<COOH>, FA<COOH>-CO_2, FA<COOH>-H_2O (or FA<COOCH$_3$>-CH_3OH) were occasionally observed, and might be also used as supporting the annotation.

Oxidatively truncated: <oxo>

[M+HCOO]$^-$ adducts (precursors)

Mod. position	Precursor	Chemical Formula	*m/z*	MS^2 Fragmentation	Mod./pos.-specific fragment[2]	Chemical Formula	*m/z*
4	PC(16:0/4:0<oxo>)	$C_{29}H_{55}O_{11}NP$	624.3518	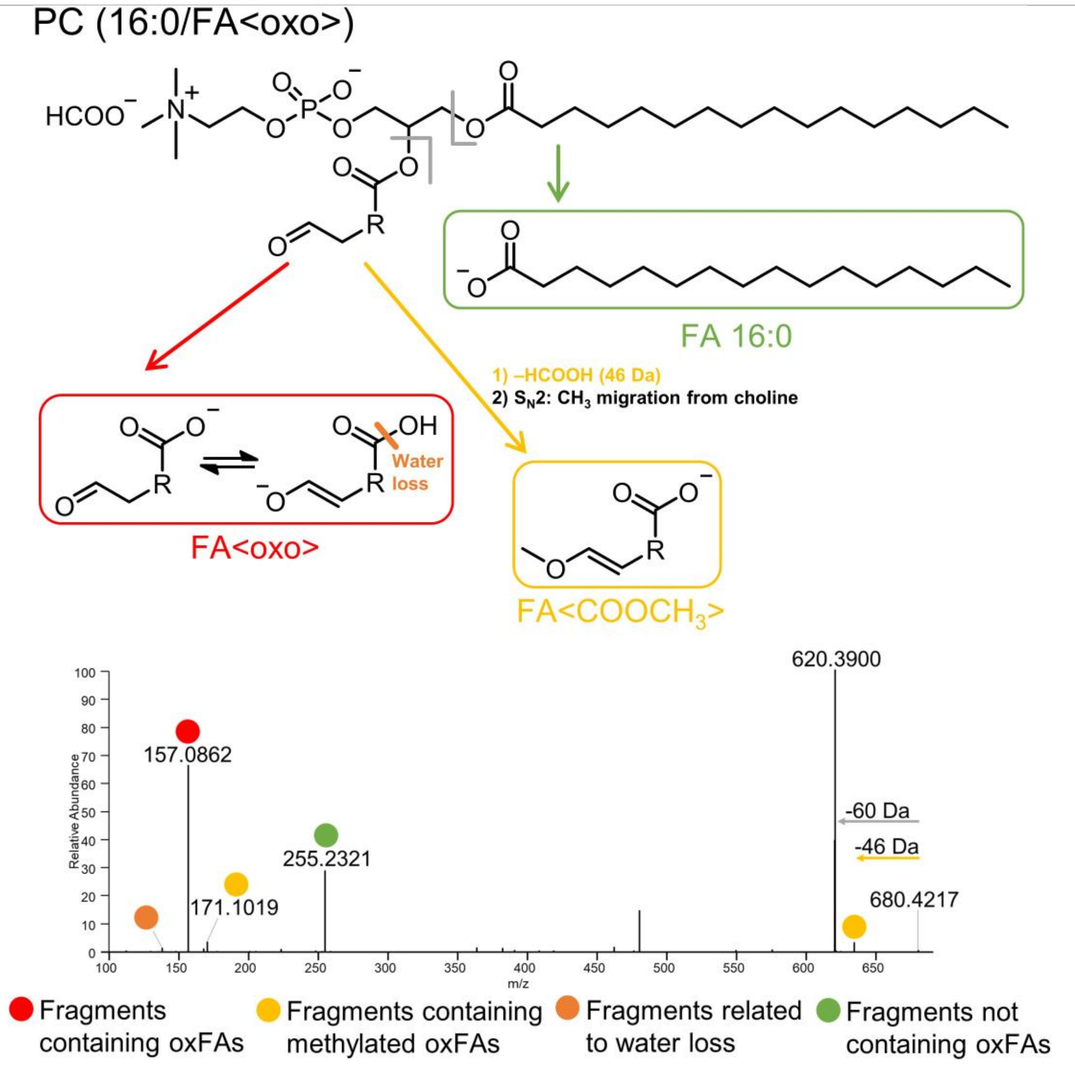	FA(4:0<oxo>)	$C_4H_5O_3$	101.0244
5	PC(16:0/5:0<oxo>)	$C_{30}H_{57}O_{11}NP$	638.3675		FA(5:0<oxo>)	$C_5H_7O_3$	115.0401
7	PC(16:0/7:0<oxo>)	$C_{32}H_{61}O_{11}NP$	666.3988		FA(7:0<oxo>)	$C_7H_{11}O_3$	143.0714
8	PC(16:0/8:0<oxo>)	$C_{33}H_{63}O_{11}NP$	680.4144		FA(8:0<oxo>)	$C_8H_{13}O_3$	157.0870
9	PC(16:0/9:0<oxo>)	$C_{34}H_{65}O_{11}NP$	694.4301		FA(9:0<oxo>)	$C_9H_{15}O_3$	171.1027
10	PC(16:0/10:0<oxo>)	$C_{35}H_{67}O_{11}NP$	708.4457		FA(10:0<oxo>)	$C_{10}H_{17}O_3$	185.1183
	PC(16:0/10:1<oxo>)	$C_{35}H_{65}O_{11}NP$	706.4301		FA(10:1<oxo>)	$C_{10}H_{15}O_3$	183.1027
11	PC(16:0/11:1<oxo>)	$C_{36}H_{67}O_{11}NP$	720.4457		FA(11:1<oxo>)	$C_{11}H_{17}O_3$	197.1183
	PC(16:0/11:2<oxo>)	$C_{36}H_{65}O_{11}NP$	718.4301		FA(11:2<oxo>)	$C_{11}H_{15}O_3$	195.1027
12	PC(16:0/12:1<oxo>)	$C_{37}H_{69}O_{11}NP$	734.4614		FA(12:1<oxo>)	$C_{12}H_{19}O_3$	211.1340
	PC(16:0/12:2<oxo>)	$C_{37}H_{67}O_{11}NP$	732.4457		FA(12:2<oxo>)	$C_{12}H_{17}O_3$	209.1183
13	PC(16:0/13:2<oxo>)	$C_{38}H_{69}O_{11}NP$	746.4614		FA(13:2<oxo>)	$C_{13}H_{19}O_3$	223.1340
	PC(16:0/13:3<oxo>)	$C_{38}H_{67}O_{11}NP$	744.4457		FA(13:3<oxo>)	$C_{13}H_{17}O_3$	221.1183
14	PC(16:0/14:3<oxo>)	$C_{39}H_{69}O_{11}NP$	758.4614		FA(14:3<oxo>)	$C_{14}H_{19}O_3$	235.1340

[2] + additional weak signals in MS^2 spectra: FA<COOH> -H_2O (or FA<$COOCH_3$>-CH_3OH), FA<COOH>-CO_2.

Full-length oxygenated: <oxo>

$[M+HCOO]^-$ adducts (precursors)

Precursor	Chemical Formula & *m/z*	MS^2 Fragmentation	Mod.-specific fragment	Chemical Formula & *m/z*	Pos.-specific fragmentation	Chemical Formula	*m/z*
**PC(16:0/18:1<oxo>)**	$C_{43}H_{81}O_{11}NP$<br>818.5553	PC (16:0/FA<oxo>)<br>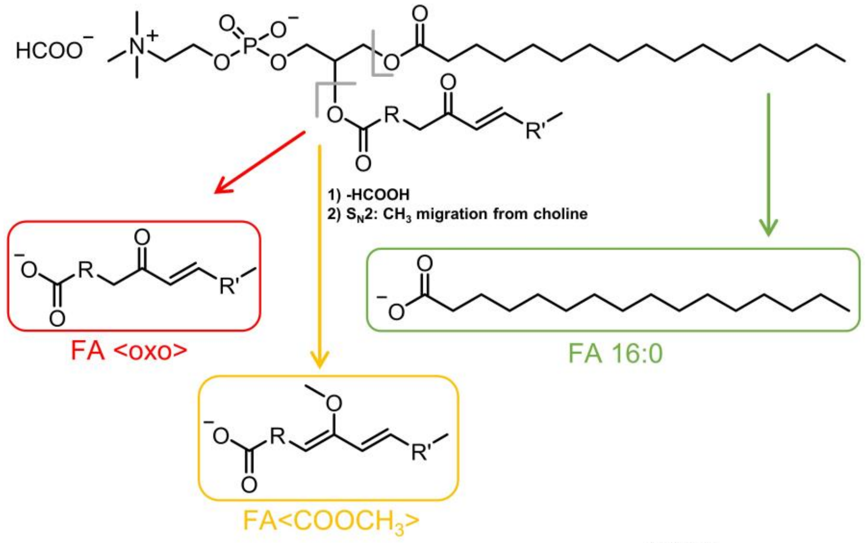<br>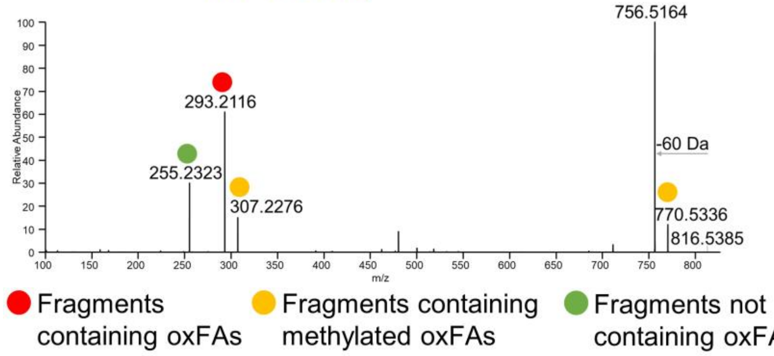<br>* Signal of precursor ion might not always be present in MS^2 spectra due to the intensive fragmentation<br>**FA fragmentation pattern:**<br>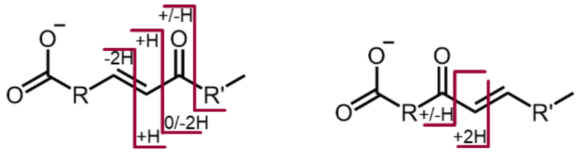	**FA(18:1<oxo>)**<br>Methylation<br>CO2 loss	**$C_{18}H_{31}O_3$**<br>**295.2279**<br>$C_{19}H_{33}O_3$<br>309.2435<br>$C_{17}H_{31}O$<br>251.2380	9<br>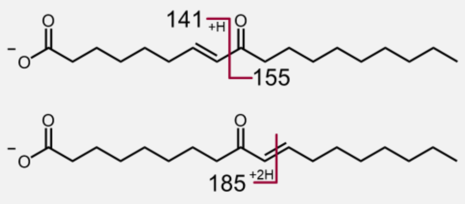	$C_8H_{13}O_2$<br>$C_{10}H_{19}O$<br>$C_{10}H_{17}O_3$	141.0921<br>155.1441<br>185.1183
**PC(16:0/18:2<oxo>)**	$C_{43}H_{79}O_{11}NP$<br>816.5396		**FA(18:2<oxo>)**<br>Methylation<br>CO2 loss	**$C_{18}H_{29}O_3$**<br>**293.2122**<br>$C_{19}H_{31}O_3$<br>307.2279<br>$C_{17}H_{29}O$<br>249.2224	8<br>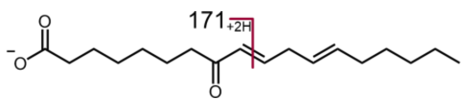	**$C_9H_{15}O_3$**	**171.1027**
					9<br>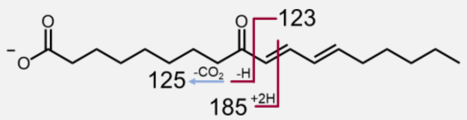	C_9H_{15}<br>$C_8H_{13}O$<br>**$C_{10}H_{17}O_3$**	123.1179<br>125.0972<br>**185.1183**
					10<br>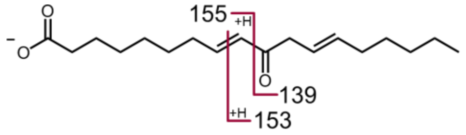	**$C_9H_{15}O$**<br>$C_{10}H_{17}O$<br>$C_9H_{15}O_2$	**139.1128**<br>153.1285<br>155.1078
					11<br>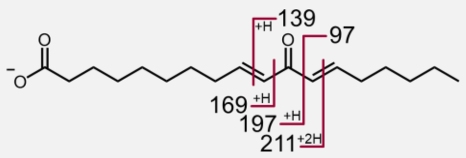	C_7H_{13}<br>$C_9H_{15}O$<br>$C_{10}H_{17}O_2$<br>$C_{11}H_{17}O_3$<br>$C_{12}H_{19}O_3$	97.1023<br>139.1128<br>169.1234<br>197.1183<br>211.1340
					12<br>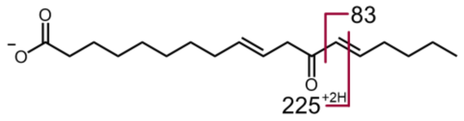	C_6H_{11}<br>$C_{13}H_{21}O_3$	83.0866<br>225.1496
					13<br>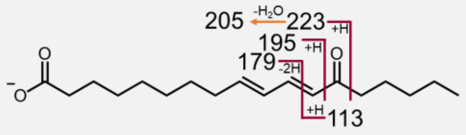	**$C_7H_{13}O$**<br>$C_{11}H_{15}O_2$<br>$C_{12}H_{19}O_2$<br>$C_{13}H_{17}O_2$<br>$C_{13}H_{19}O_3$	**113.0972**<br>179.1078<br>195.1391<br>205.1234[3]<br>223.1340[4]
					14<br>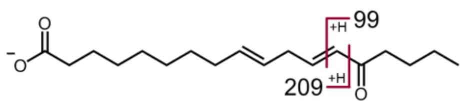	$C_6H_{11}O$<br>$C_{13}H_{21}O_2$	99.0815<br>209.1547

[3] The signal is seen in the case of FA(18:2<OOH{13}>).

[4] The signal is seen in the case of FA(18:2<OOH{13}>).

Full-length oxygenated: <oxo> (continued)

[M+HCOO]- adducts (precursors)

Precursor	Chemical Formula & *m/z*	MS² Fragmentation	Mod.-specific fragment	Chemical Formula & *m/z*	Pos.-specific fragmentation	Chemical Formula	*m/z*
**PC(16:0/20:4<oxo>)**	$C_{45}H_{79}O_{11}NP$ 840.5396	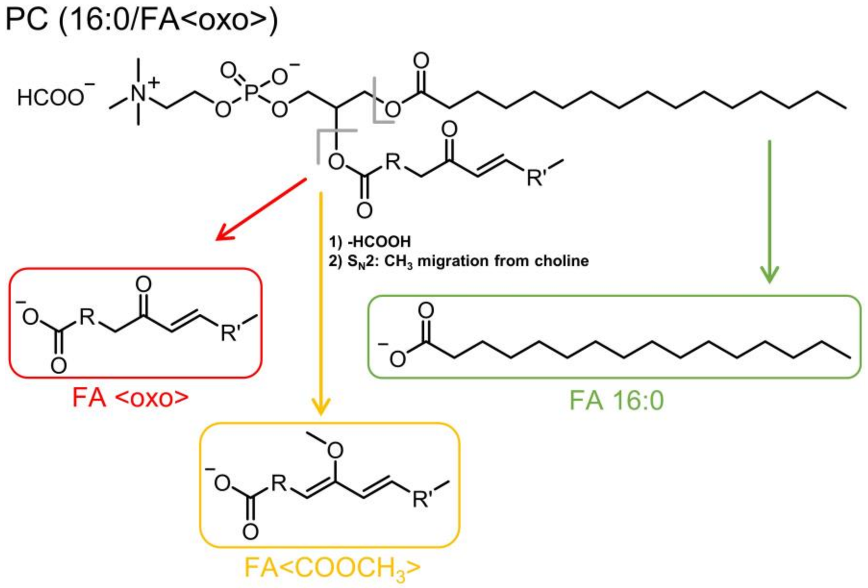<br>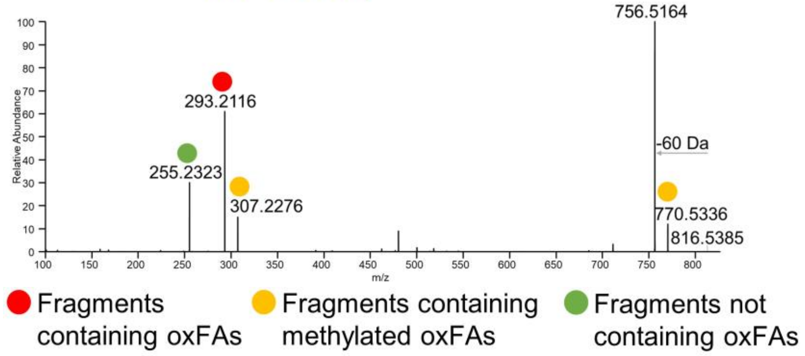<br>* The precursor ion m/z may not always present in MS² spectra due to the intensive fragmentation<br>**FA fragmentation pattern:**<br>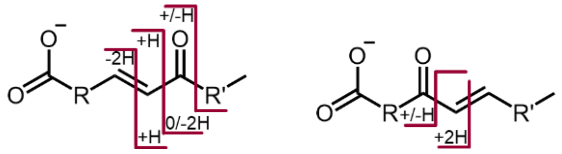	**FA(20:4<oxo>)**<br>Methylation<br>CO_2 loss	**$C_{20}H_{29}O_3$ 317.2122**<br>$C_{21}H_{31}O_3$ 331.2279<br>$C_{19}H_{29}O$ 273.2224	5 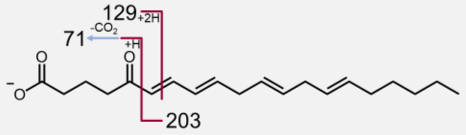	C_4H_7O<br>$C_6H_9O_3$<br>**$C_{15}H_{23}$**	71.0502<br>129.0557<br>**203.1805**
					6 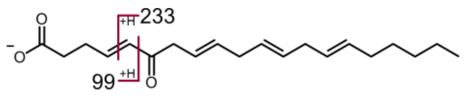	$C_5H_7O_2$<br>$C_{16}H_{25}O$	99.0452<br>233.1911
					7 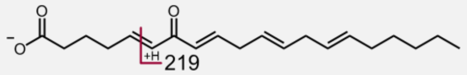	$C_{15}H_{23}O$	219.1754
					8 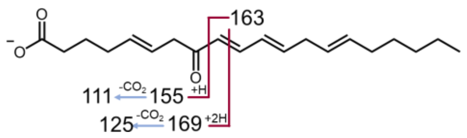	$C_7H_{11}O$<br>$C_8H_{13}O$<br>$C_8H_{11}O_3$<br>$C_{12}H_{19}$<br>$C_9H_{13}O_3$	111.0815<br>125.0972<br>155.0714<br>163.1492<br>169.0870
					9 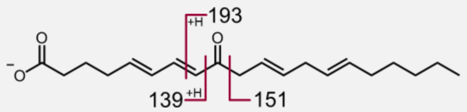	$C_8H_{11}O_2$<br>$C_{11}H_{19}$<br>$C_{13}H_{21}O$	139.0765<br>151.1492<br>193.1598
					11 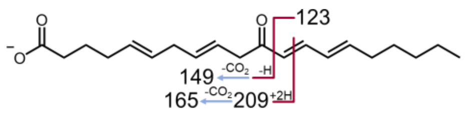	C_9H_{15}<br>$C_{10}H_{13}O$<br>$C_{11}H_{17}O$<br>$C_{12}H_{17}O_3$	123.1128<br>149.0972<br>165.1285<br>209.1183
					12 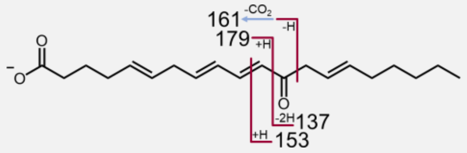	$C_9H_{13}O$<br>**$C_{10}H_{17}O$**<br>$C_{11}H_{13}O$<br>$C_{11}H_{15}O_2$	137.0972<br>**153.1285**<br>161.0971<br>179.1078
					13 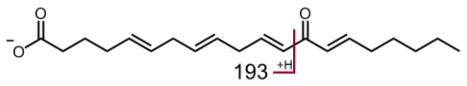	$C_{12}H_{17}O_2$	193.1234
					14 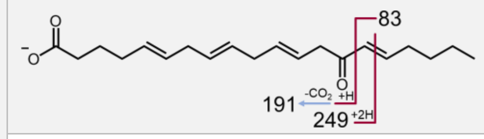	C_6H_{11}<br>**$C_{13}H_{19}O$**<br>$C_{15}H_{21}O_3$	83.0866<br>**191.1441**<br>249.1496
					15 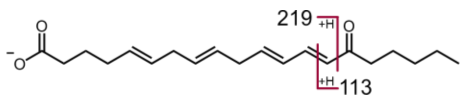	$C_7H_{13}O$<br>$C_9H_{15}O$<br>$C_{14}H_{19}O_2$	**113.0972**<br>139.1128<br>219.1391

Full-length oxygenated: <OH>

[M+HCOO]$^-$ adducts (precursors)

Precursor	Chemical Formula & *m/z*	MS^2 Fragmentation	Mod.-specific fragment	Chemical Formula & *m/z*	Pos.-specific fragmentation	Chemical Formula	*m/z*
**PC(16:0/18:1<OH>)**	$C_{43}H_{83}O_{11}NP$<br>820.5709	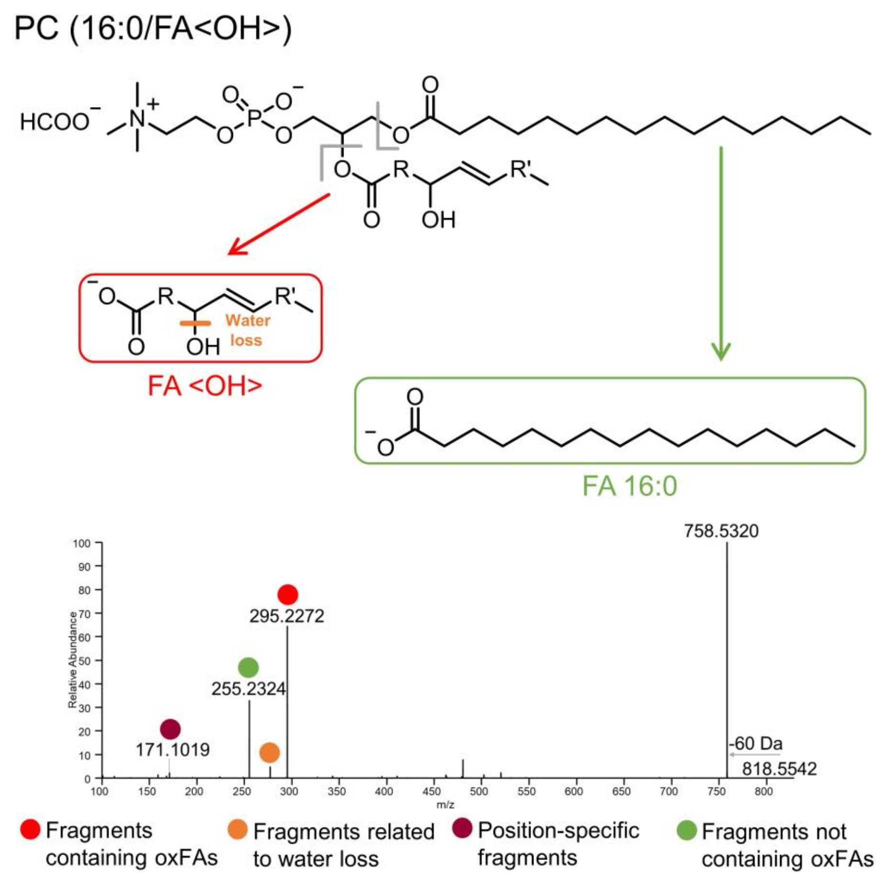	**FA(18:1<OH>)**<br>H_2O loss<br>CO_2 loss<br>H_2O&CO_2 loss	**$C_{18}H_{33}O_3$**<br>**297.2435**<br>$C_{18}H_{31}O_2$<br>279.2330<br>$C_{17}H_{33}O$<br>253.2536<br>$C_{17}H_{31}$<br>235.2431	9<br>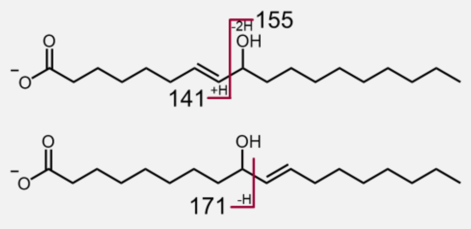	$C_8H_{13}O_2$<br>$C_{10}H_{19}O$<br>**$C_9H_{15}O_3$**	141.0921<br>155.1441<br>**171.1027**
**PC(16:0/18:2<OH>)**	$C_{43}H_{81}O_{11}NP$<br>818.5553	**FA fragmentation pattern:**<br>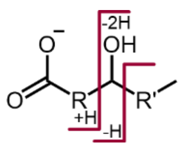	**FA(18:2<OH>)**<br>H_2O loss<br>CO_2 loss<br>H_2O&CO_2 loss	**$C_{18}H_{31}O_3$**<br>**295.2279**<br>$C_{18}H_{29}O_2$<br>277.2173<br>$C_{17}H_{31}O$<br>251.2380<br>$C_{17}H_{29}$<br>233.2275	9<br>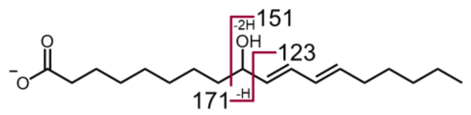	C_9H_{15}<br>$C_{10}H_{15}O$<br>**$C_9H_{15}O_3$**	123.1179<br>151.1128<br>**171.1027**
					10<br>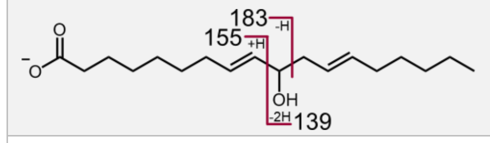	$C_9H_{15}O$<br>$C_9H_{15}O_2$<br>$C_{10}H_{15}O_3$	139.1128<br>155.1078<br>183.1027
					11<br>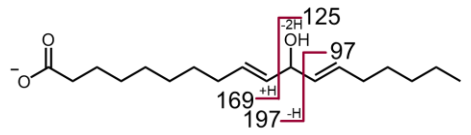	C_7H_{13}<br>$C_8H_{13}O$<br>$C_{10}H_{17}O_2$<br>$C_{11}H_{17}O_3$	97.1023<br>125.0972<br>169.1234<br>197.1183
					12<br>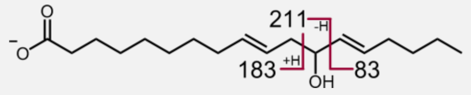	C_6H_{11}<br>**$C_{11}H_{19}O_2$**<br>$C_{12}H_{19}O_3$	83.0866<br>**183.1391**<br>211.1340
					13<br>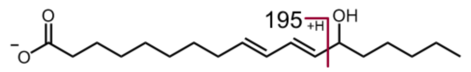	**$C_{12}H_{19}O_2$**	**195.1391**
					14<br>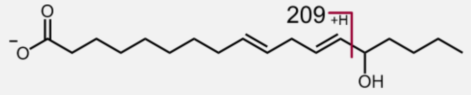	$C_{13}H_{21}O_2$	209.1547

Full-length oxygenated: <OH> (continued)

[M+HCOO]- adducts

Precursor	Chemical Formula & *m/z*	MS2 Fragmentation	Mod.-specific fragment	Chemical Formula & *m/z*	Pos.-specific fragmentation	Chemical Formula	*m/z*
**PC(16:0/20:4<OH>)**	$C_{45}H_{81}O_{11}NP$ 842.5553	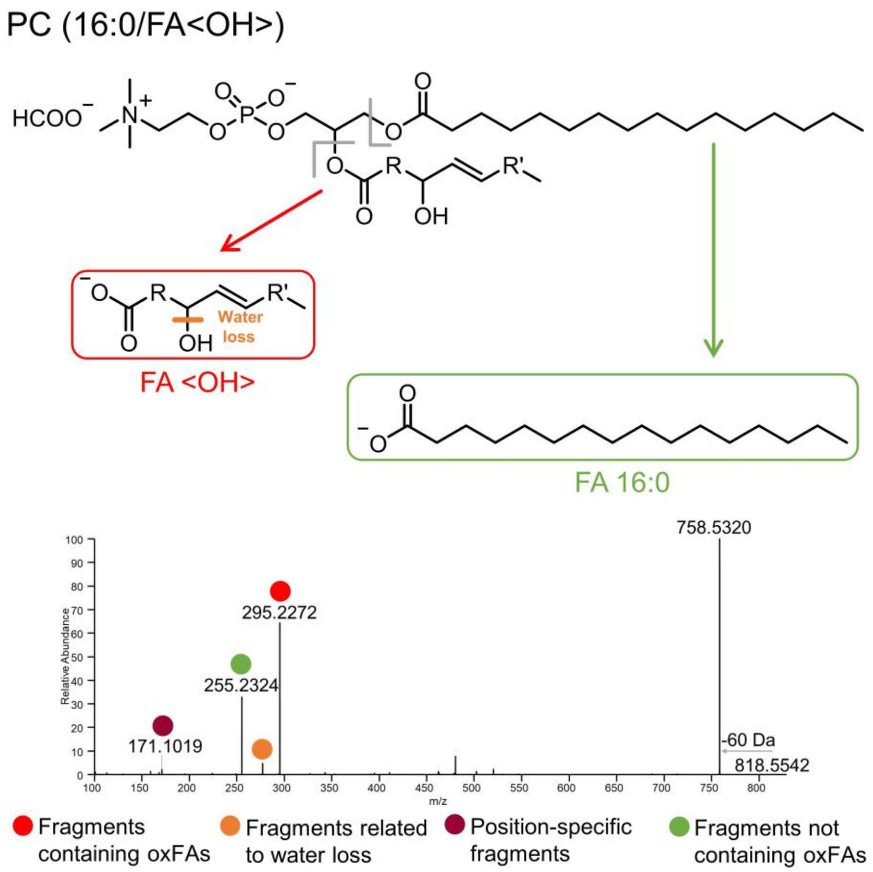 **FA fragmentation pattern:** 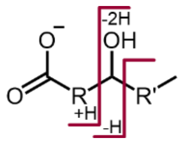	**FA(20:4<OH>)**<br>H_2O loss<br>CO_2 loss<br>H_2O&CO_2 loss	**$C_{20}H_{31}O_3$ 319.2279**<br>$C_{20}H_{29}O_2$ 301.2173<br>$C_{19}H_{31}O$ 275.2380<br>$C_{19}H_{29}$ 257.2275	5 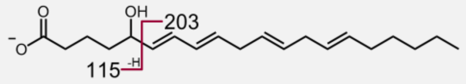	**$C_5H_7O_3$**<br>$C_{15}H_{23}$	**115.0401**<br>203.1805[5]
					6 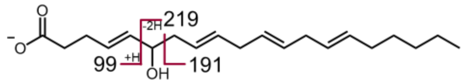	$C_5H_7O_2$<br>$C_{14}H_{23}$<br>$C_{15}H_{23}O$	99.0452<br>191.1805<br>219.1754
					7 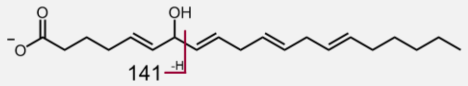	$C_7H_9O_3$	141.0557
					8 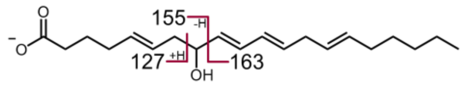	$C_7H_{11}O_2$<br>**$C_8H_{11}O_3$**<br>**$C_{12}H_{19}$**	127.0765<br>**155.0714**<br>**163.1492**
					9 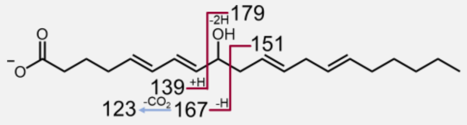	**$C_8H_{11}O$**<br>$C_8H_{11}O_2$<br>$C_{11}H_{19}$<br>$C_9H_{11}O_3$<br>**$C_{12}H_{19}O$**	**123.0815**<br>139.0765<br>151.1492<br>167.0714<br>**179.1441**
					11 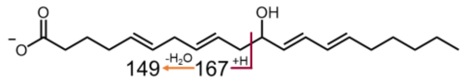	$C_{10}H_{13}O$<br>**$C_{10}H_{15}O_2$**	149.0972<br>**167.1078**
					12 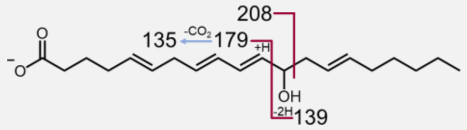	$C_{10}H_{15}$<br>$C_9H_{15}O$<br>**$C_{11}H_{15}O_2$**<br>$C_{12}H_{16}O_3$	135.1179<br>139.1128<br>**179.1078**<br>208.1105[6]
					13 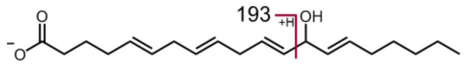	$C_{12}H_{17}O_2$	193.1234
					14 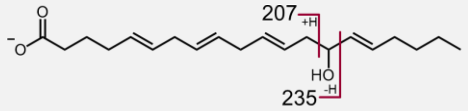	**$C_{13}H_{19}O_2$**<br>$C_{14}H_{19}O_3$	**207.1391**<br>235.1340
					15 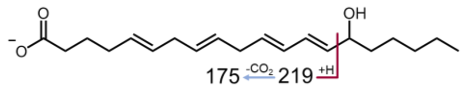	**$C_{13}H_{19}$**<br>**$C_{14}H_{19}O_2$**	**175.1492**<br>**219.1391**

[5] The signal is not OH{5}-specific (can be found in the spectra of other isomers, but less intense)
[6] The signal should correspond to an anion-radical that is the unique case for position 12.

Full-length oxygenated: <ep>

[M+HCOO]⁻ adducts

Precursor	Chemical Formula & *m/z*	MS^2 Fragmentation	Mod.-specific fragment	Chemical Formula & *m/z*	Pos.-specific fragmentation	Chemical Formula	*m/z*
**PC(16:0/18:0<ep>)**	$C_{43}H_{83}O_{11}NP$<br>820.5709	PC (16:0/FA<ep>)<br>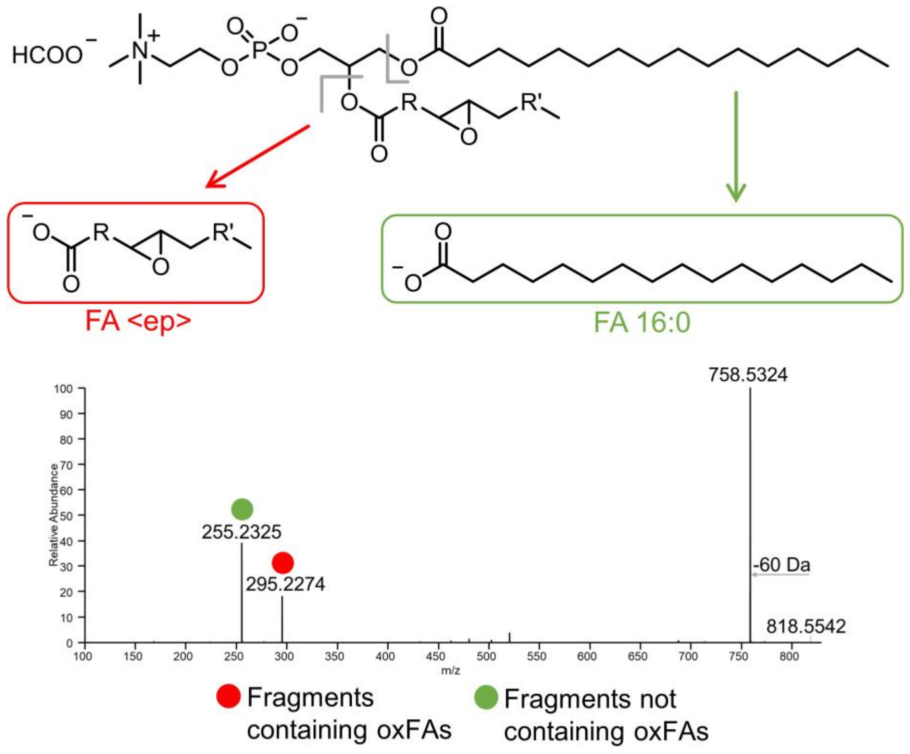<br>**FA fragmentation pattern:**<br>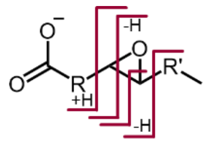	**FA(18:0<ep>)**<br>H_2O loss	**$C_{18}H_{33}O_3$**<br>**297.2435**<br>$C_{18}H_{31}O_2$<br>279.2330	9-10<br>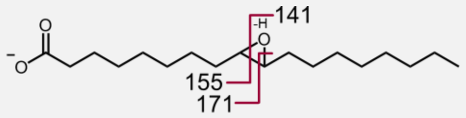	$C_9H_{17}O$<br>$C_9H_{15}O_2$<br>**$C_9H_{15}O_3$**	141.1285<br>155.1078<br>**171.1027**
**PC(16:0/18:1<ep>)**	$C_{43}H_{81}O_{11}NP$<br>818.5553		**FA(18:1<ep>)**<br>H_2O loss	**$C_{18}H_{31}O_3$**<br>**295.2279**<br>$C_{18}H_{29}O_2$<br>277.2173	9-10<br>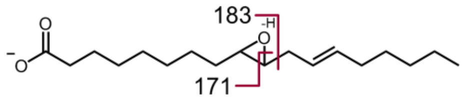	**$C_9H_{15}O_3$**<br>$C_{10}H_{15}O_3$	**171.1027**<br>183.1027
					12-13<br>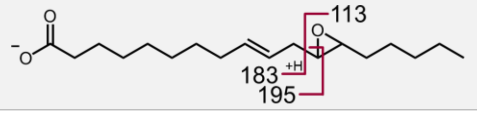	$C_7H_{13}O$<br>**$C_{11}H_{19}O_2$**<br>**$C_{12}H_{19}O_2$**	113.0972<br>**183.1391**<br>**195.1391**
**PC(16:0/20:3<ep>)**	$C_{45}H_{81}O_{11}NP$<br>842.5553		**FA(20:3<ep>)**<br>H_2O loss<br>H_2O&CO_2 loss	**$C_{20}H_{31}O_3$**<br>**319.2279**<br>$C_{20}H_{29}O_2$<br>301.2173<br>$C_{19}H_{29}$<br>257.2275	5-6<br>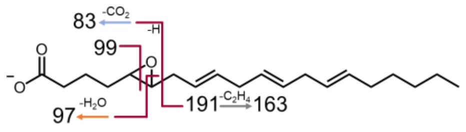	C_5H_7O<br>$C_5H_5O_2$<br>$C_5H_7O_2$<br>**$C_{12}H_{19}$**<br>**$C_{14}H_{23}$**	83.0502<br>97.0295<br>99.0452<br>**163.1492**<br>**191.1805**
					8-9<br>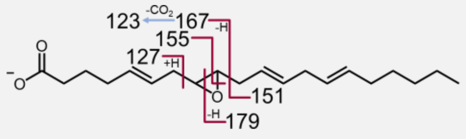	**$C_7H_{11}O_2$**<br>**$C_8H_{11}O$**<br>**$C_{11}H_{19}$**<br>**$C_8H_{11}O_3$**<br>**$C_9H_{11}O_3$**<br>**$C_{12}H_{19}O$**	**127.0765**[7]<br>**123.0815**<br>**151.1492**<br>**155.0714**<br>**167.0714**<br>**179.1441**
					11-12<br>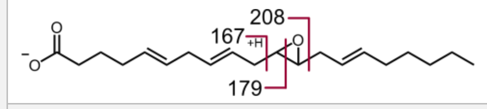	**$C_{10}H_{15}O_2$**<br>**$C_{11}H_{15}O_2$**<br>$C_{12}H_{15}O_3$	**167.1078**<br>**179.1078**<br>208.1104[8]
					14-15<br>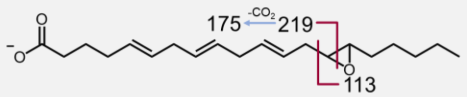	$C_7H_{13}O$<br>**$C_{13}H_{19}$**<br>**$C_{14}H_{19}O_2$**	113.0972<br>**175.1492**<br>**219.1391**

[7] The abundances of fragments do not differ much, so it is difficult to choose the major one.
[8] The signal should correspond to an anion-radical that is the unique case for position 12.

Full-length oxygenated: <OOH>

[M+HCOO]⁻ adducts

Precursor	Chemical Formula & *m/z*	MS^2 Fragmentation	Mod.-specific fragment	Chemical Formula & *m/z*	Pos.-specific fragmentation
**PC(16:0/18:1<OOH>)**	$C_{43}H_{83}O_{12}NP$<br>836.5658	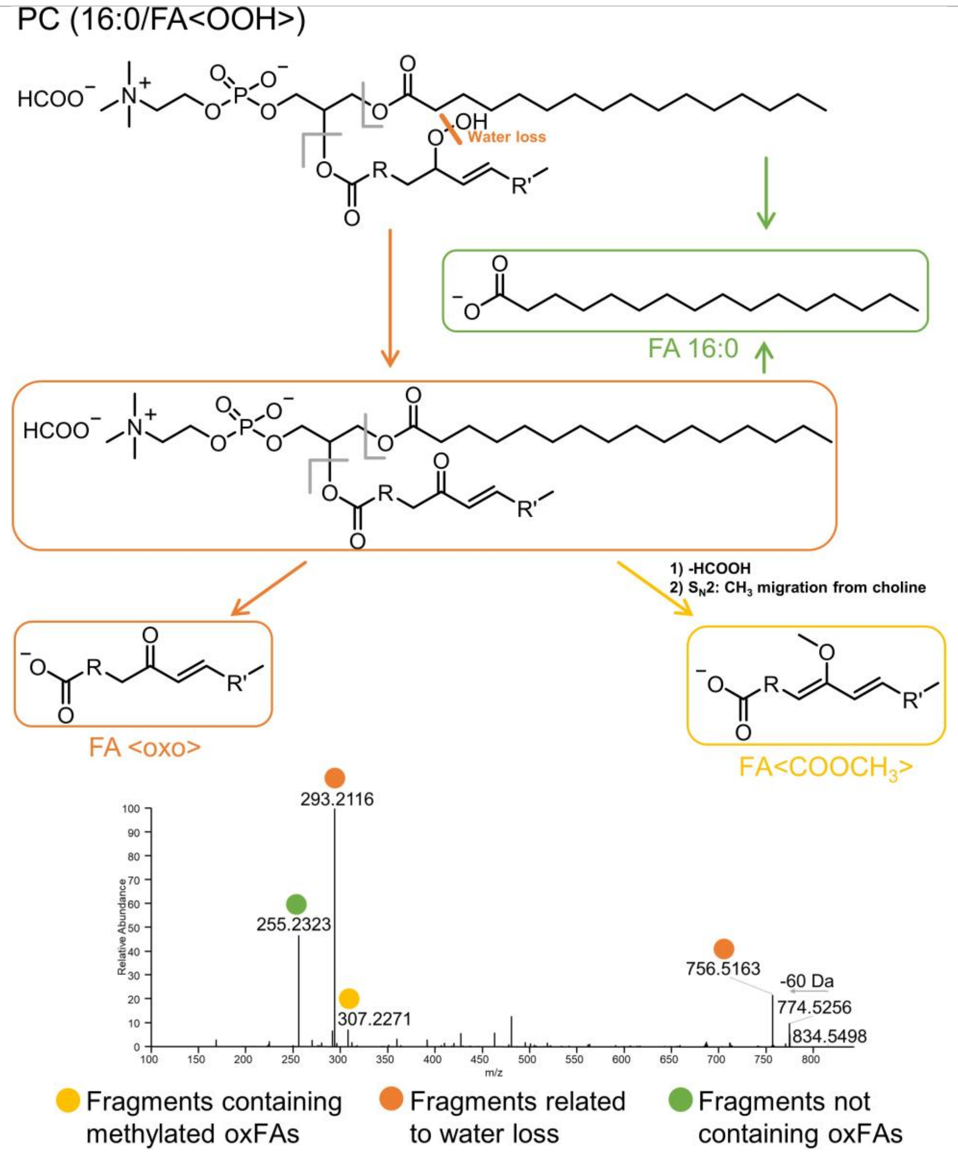	FA(18:1<OOH>)<br>**H_2O loss**<br>Methylation<br>H_2O&CO_2 loss<br>2H_2O loss<br>H_2O_2 loss	$C_{18}H_{33}O_4$<br>313.2384<br>**$C_{18}H_{31}O_3$**<br>**295.2279**<br>$C_{19}H_{33}O_3$<br>309.2435<br>$C_{17}H_{31}O$<br>251.2380<br>$C_{18}H_{29}O_2$<br>277.2173<br>$C_{18}H_{27}O_2$<br>275.2016	
**PC(16:0/18:2<OOH>)**	$C_{43}H_{81}O_{12}NP$<br>834.5502		FA(18:2<OOH>)<br>**H_2O loss**<br>Methylation<br>H_2O&CO_2 loss<br>2H_2O loss<br>H_2O_2 loss	$C_{18}H_{31}O_4$<br>311.2228<br>**$C_{18}H_{29}O_3$**<br>**293.2122**<br>$C_{19}H_{31}O_3$<br>307.2279<br>$C_{17}H_{29}O$<br>249.2224<br>$C_{18}H_{27}O_2$<br>275.2016<br>$C_{18}H_{29}O_2$<br>277.2173	See above (Full-length oxygenated: <oxo>)

Full-length oxygenated: <OOH> (continued)

[M+HCOO]$^-$ adducts

Precursor	Chemical Formula & *m/z*	MS^2 Fragmentation	Mod.-specific fragment	Chemical Formula & *m/z*	Pos.-specific fragmentation
**PC(16:0/20:4<OOH>)**	$C_{45}H_{81}O_{12}NP$<br>858.5502	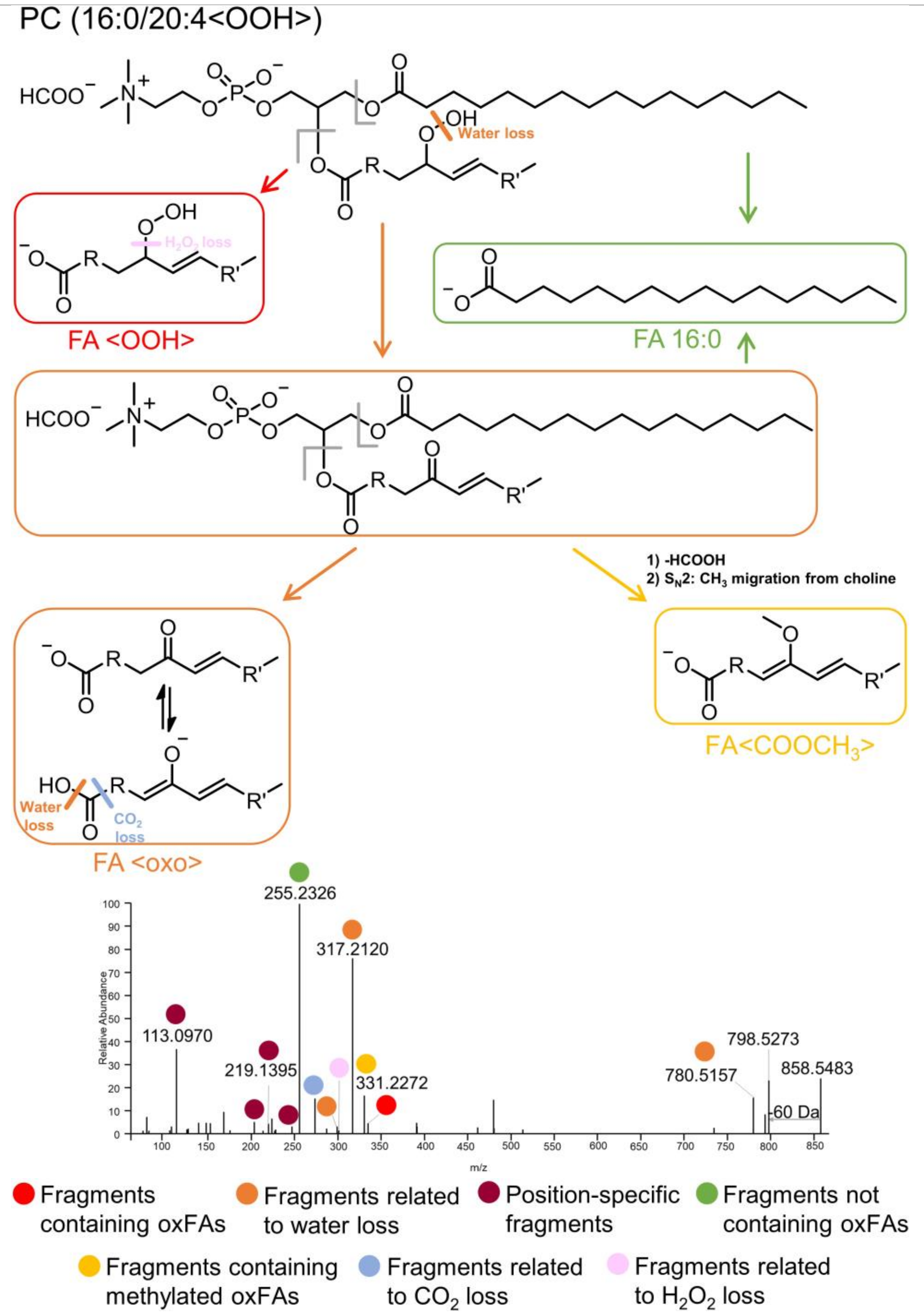	FA(20:4<OOH>)<br>**H_2O loss**<br>Methylation<br>H_2O&CO_2 loss<br>$2H_2O$ loss<br>H_2O_2 loss	$C_{20}H_{31}O_4$<br>335.2228<br>**$C_{20}H_{29}O_3$**<br>**317.2122**<br>$C_{21}H_{31}O_3$<br>331.2279<br>$C_{19}H_{29}O$<br>273.2224<br>$C_{20}H_{27}O_2$<br>299.2017<br>$C_{20}H_{29}O_2$<br>301.2173	See above (Full-length oxygenated: <oxo>)

CHOLESTERYL ESTERS

Oxidatively truncated: <COOH>

[M+Na]$^+$ adducts

Mod. position	Precursor	Chemical Formula	*m/z*	MS^2 Fragmentation	Mod./pos.-specific fragment	Chemical Formula	*m/z*
4	CE(4:0<COOH>)	$C_{31}H_{50}O_4Na$	509.3601		FA(4:0<COOH>)	$C_4H_6O_4Na$	141.0158
5	CE(5:0<COOH>)	$C_{32}H_{52}O_4Na$	523.3758		FA(5:0<COOH>)	$C_5H_8O_4Na$	155.0315
7	CE(7:0<COOH>)	$C_{34}H_{56}O_4Na$	551.4071		FA(7:0<COOH>)	$C_7H_{12}O_4Na$	183.0628
8	CE(8:0<COOH>)	$C_{35}H_{58}O_4Na$	565.4227		FA(8:0<COOH>)	$C_8H_{14}O_4Na$	197.0784
9	CE(9:0<COOH>)	$C_{36}H_{60}O_4Na$	579.4384		FA(9:0<COOH>)	$C_9H_{16}O_4Na$	211.0941
10	CE(10:0<COOH>)	$C_{37}H_{62}O_4Na$	593.4540		FA(10:0<COOH>)	$C_{10}H_{18}O_4Na$	225.1097
	CE(10:1<COOH>)	$C_{37}H_{60}O_4Na$	591.4384		FA(10:1<COOH>)	$C_{10}H_{16}O_4Na$	223.0941
11	CE(11:1<COOH>)	$C_{38}H_{62}O_4Na$	605.4540		FA(11:1<COOH>)	$C_{11}H_{18}O_4Na$	237.1097
	CE(11:2<COOH>)	$C_{38}H_{60}O_4Na$	603.4384		FA(11:2<COOH>)	$C_{11}H_{16}O_4Na$	235.0941
12	CE(12:1<COOH>)	$C_{39}H_{64}O_4Na$	619.4697		FA(12:1<COOH>)	$C_{12}H_{20}O_4Na$	251.1254
	CE(12:2<COOH>)	$C_{39}H_{62}O_4Na$	617.4540		FA(12:2<COOH>)	$C_{12}H_{18}O_4Na$	249.1097
13	CE(13:2<COOH>)	$C_{40}H_{64}O_4Na$	631.4697		FA(13:2<COOH>)	$C_{13}H_{20}O_4Na$	263.1254
	CE(13:3<COOH>)	$C_{40}H_{62}O_4Na$	629.4540		FA(13:3<COOH>)	$C_{13}H_{18}O_4Na$	261.1097
14	CE(14:3<COOH>)	$C_{41}H_{64}O_4Na$	643.4697		FA(14:3<COOH>)	$C_{14}H_{20}O_4Na$	275.1254

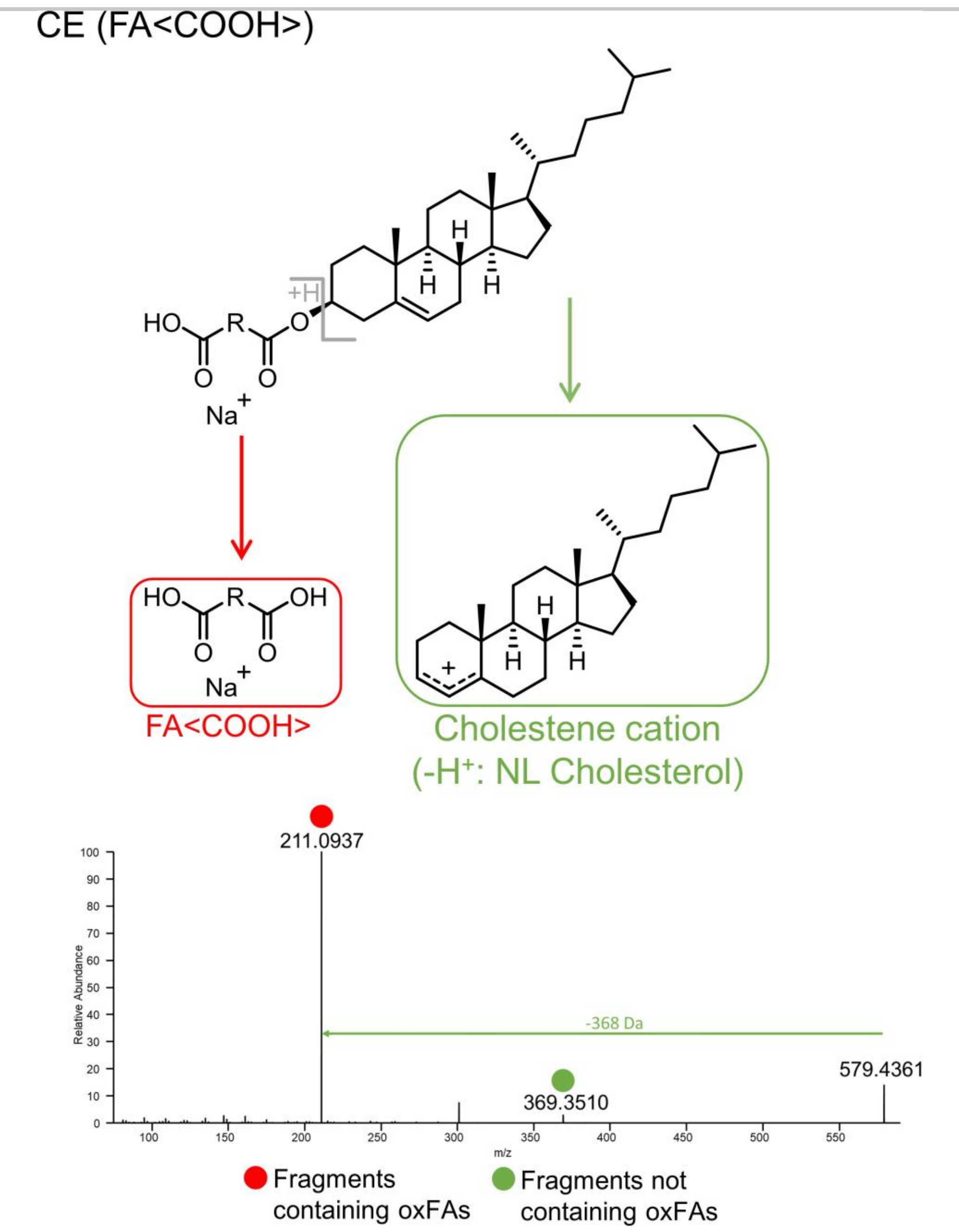

Oxidatively truncated: <oxo>

$[M+Na]^+$ adducts

Mod. position	Precursor	Chemical Formula	*m/z*	MS² Fragmentation	Mod./pos.-specific fragment	Chemical Formula	*m/z*
**4**	CE(4:0<oxo>)	$C_{31}H_{50}O_3Na$	493.3652	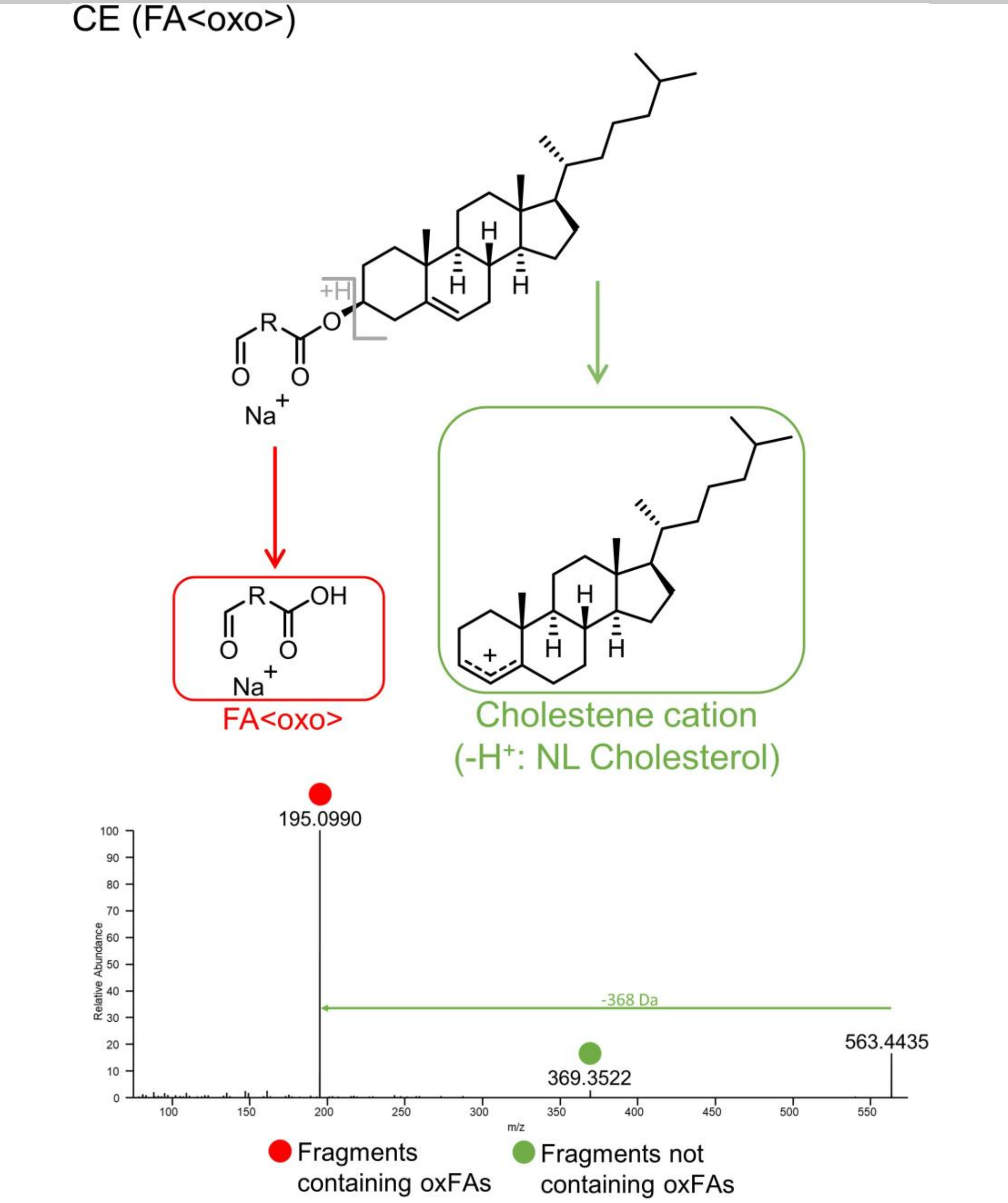	FA(4:0<oxo>)	$C_4H_6O_3Na$	125.0209
**5**	CE(5:0<oxo>)	$C_{32}H_{52}O_3Na$	507.3809		FA(5:0<oxo>)	$C_5H_8O_3Na$	139.0366
**7**	CE(7:0<oxo>)	$C_{34}H_{56}O_3Na$	535.4122		FA(7:0<oxo>)	$C_7H_{12}O_3Na$	167.0679
**8**	CE(8:0<oxo>)	$C_{35}H_{58}O_3Na$	549.4278		FA(8:0<oxo>)	$C_8H_{14}O_3Na$	181.0835
**9**	CE(9:0<oxo>)	$C_{36}H_{60}O_3Na$	563.4435		FA(9:0<oxo>)	$C_9H_{16}O_3Na$	195.0992
**10**	CE(10:0<oxo>)	$C_{37}H_{62}O_3Na$	577.4591		FA(10:0<oxo>)	$C_{10}H_{18}O_3Na$	209.1148
	CE(10:1<oxo>)	$C_{37}H_{60}O_3Na$	575.4435		FA(10:1<oxo>)	$C_{10}H_{16}O_3Na$	207.0992
**11**	CE(11:1<oxo>)	$C_{38}H_{62}O_3Na$	589.4591		FA(11:1<oxo>)	$C_{11}H_{18}O_3Na$	221.1148
	CE(11:2<oxo>)	$C_{38}H_{60}O_3Na$	587.4435		FA(11:2<oxo>)	$C_{11}H_{16}O_3Na$	219.0992
**12**	CE(12:1<oxo>)	$C_{39}H_{64}O_3Na$	603.4748		FA(12:1<oxo>)	$C_{12}H_{20}O_3Na$	235.1305
	CE(12:2<oxo>)	$C_{39}H_{62}O_3Na$	601.4591		FA(12:2<oxo>)	$C_{12}H_{18}O_3Na$	233.1148
**13**	CE(13:2<oxo>)	$C_{40}H_{64}O_3Na$	615.4748		FA(13:2<oxo>)	$C_{13}H_{20}O_3Na$	247.1305
	CE(13:3<oxo>)	$C_{40}H_{62}O_3Na$	613.4591		FA(13:3<oxo>)	$C_{13}H_{18}O_3Na$	245.1148
**14**	CE(14:3<oxo>)	$C_{41}H_{64}O_3Na$	627.4748		FA(14:3<oxo>)	$C_{14}H_{20}O_3Na$	259.1305

Full-length oxygenated: <oxo>

$[M+Na]^+$ adducts

Precursor	Chemical Formula	*m/z*	MS^2 Fragmentation	Mod.-specific fragment	Chemical Formula	*m/z*
**CE(18:1)<oxo>**	$C_{45}H_{76}O_3Na$	687.5687	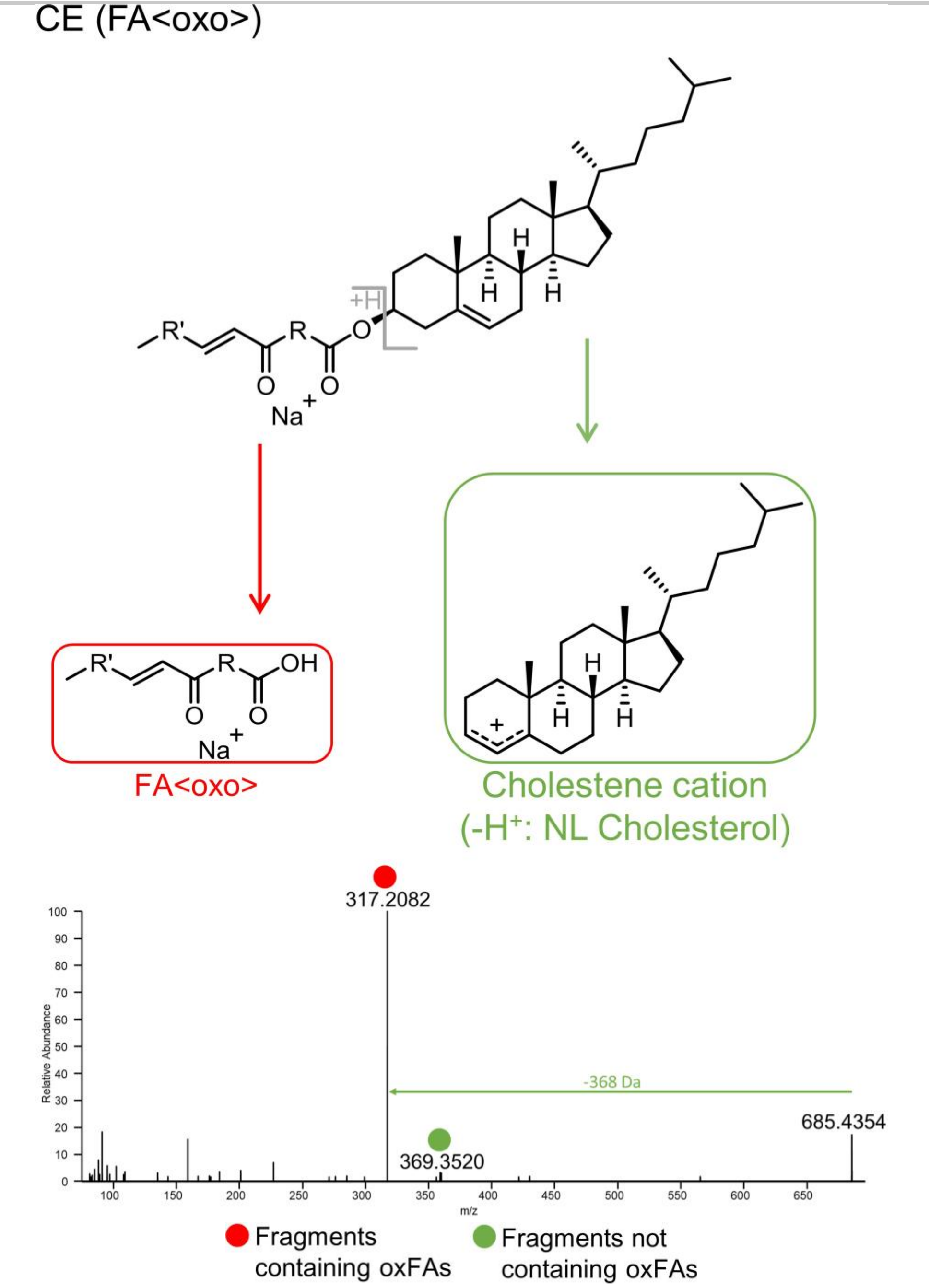	FA(18:1)<oxo>	$C_{18}H_{32}O_3Na$	319.2244
**CE(18:2)<oxo>**	$C_{45}H_{74}O_3Na$	685.5530		FA(18:2)<oxo>	$C_{18}H_{30}O_3Na$	317.2087
**CE(20:4)<oxo>**	$C_{47}H_{74}O_3Na$	709.5530		FA(20:4)<oxo>	$C_{20}H_{30}O_3Na$	341.2087

Full-length oxygenated: <OH>

$[M+Na]^+$ adducts

Precursor	Chemical Formula	*m/z*	MS² Fragmentation	Mod.-specific fragment	Chemical Formula	*m/z*
**CE(18:1<OH>)**	$C_{45}H_{78}O_3Na$	689.5843		**FA(18:1<OH>)**<br>H₂O loss	**$C_{18}H_{34}O_3Na$**<br>$C_{18}H_{32}O_2Na$	**321.2400**<br>303.2295
**CE(18:2<OH>)**	$C_{45}H_{76}O_3Na$	687.5687		**FA(18:2<OH>)**<br>H₂O loss	**$C_{18}H_{32}O_3Na$**<br>$C_{18}H_{30}O_2Na$	**319.2244**<br>301.2138
**CE(20:4<OH>)**	$C_{47}H_{76}O_3Na$	711.5687		**FA(20:4<OH>)**<br>H₂O loss	**$C_{20}H_{32}O_3Na$**<br>$C_{20}H_{30}O_2Na$	**343.2244**<br>325.2138

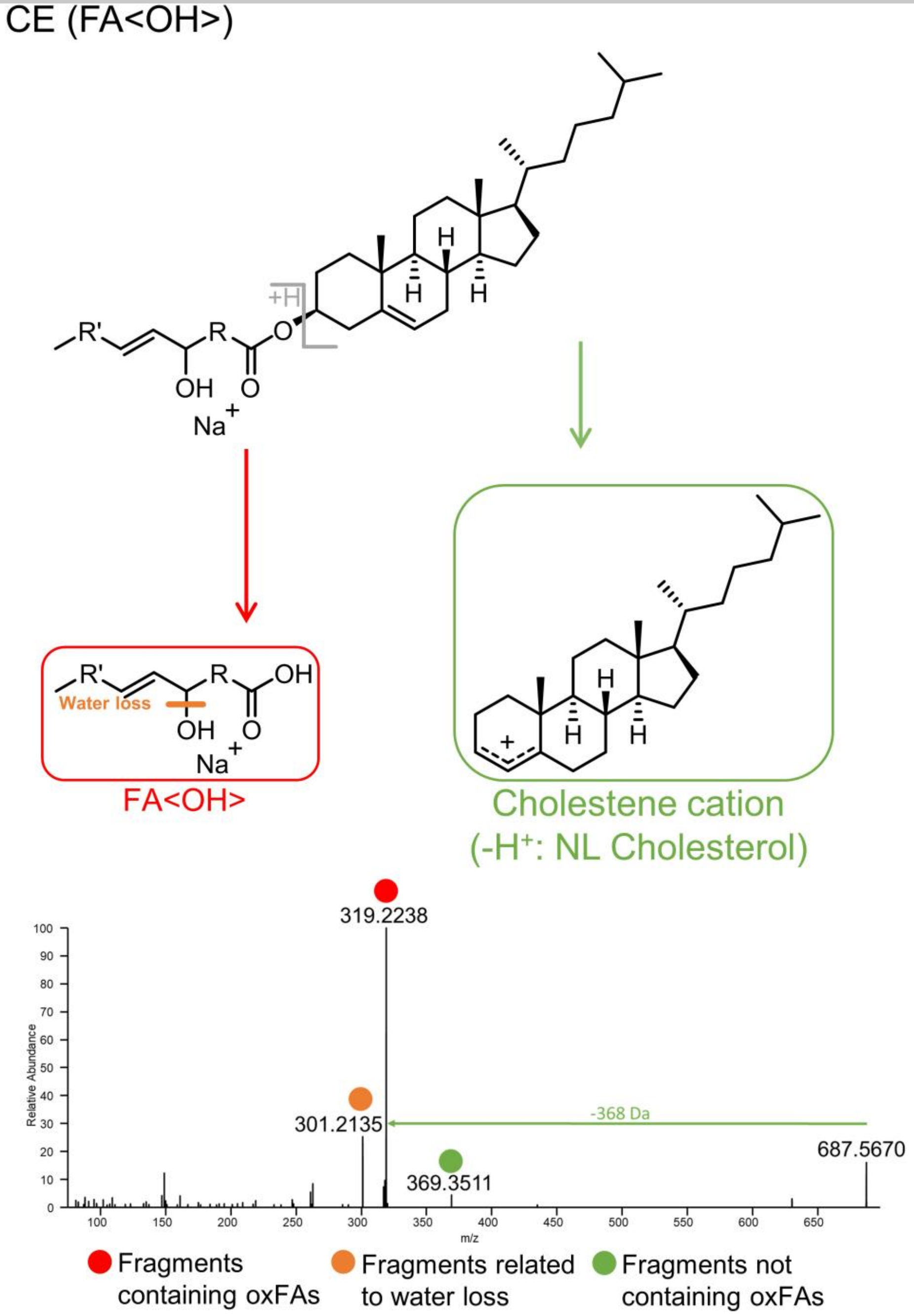

Full-length oxygenated: <ep>

$[M+Na]^+$ adducts

Precursor	Chemical Formula	*m/z*	MS^2 Fragmentation	Mod.-specific fragment	Chemical Formula	*m/z*
**CE(18:0<ep>)**	$C_{45}H_{78}O_3Na$	689.5843	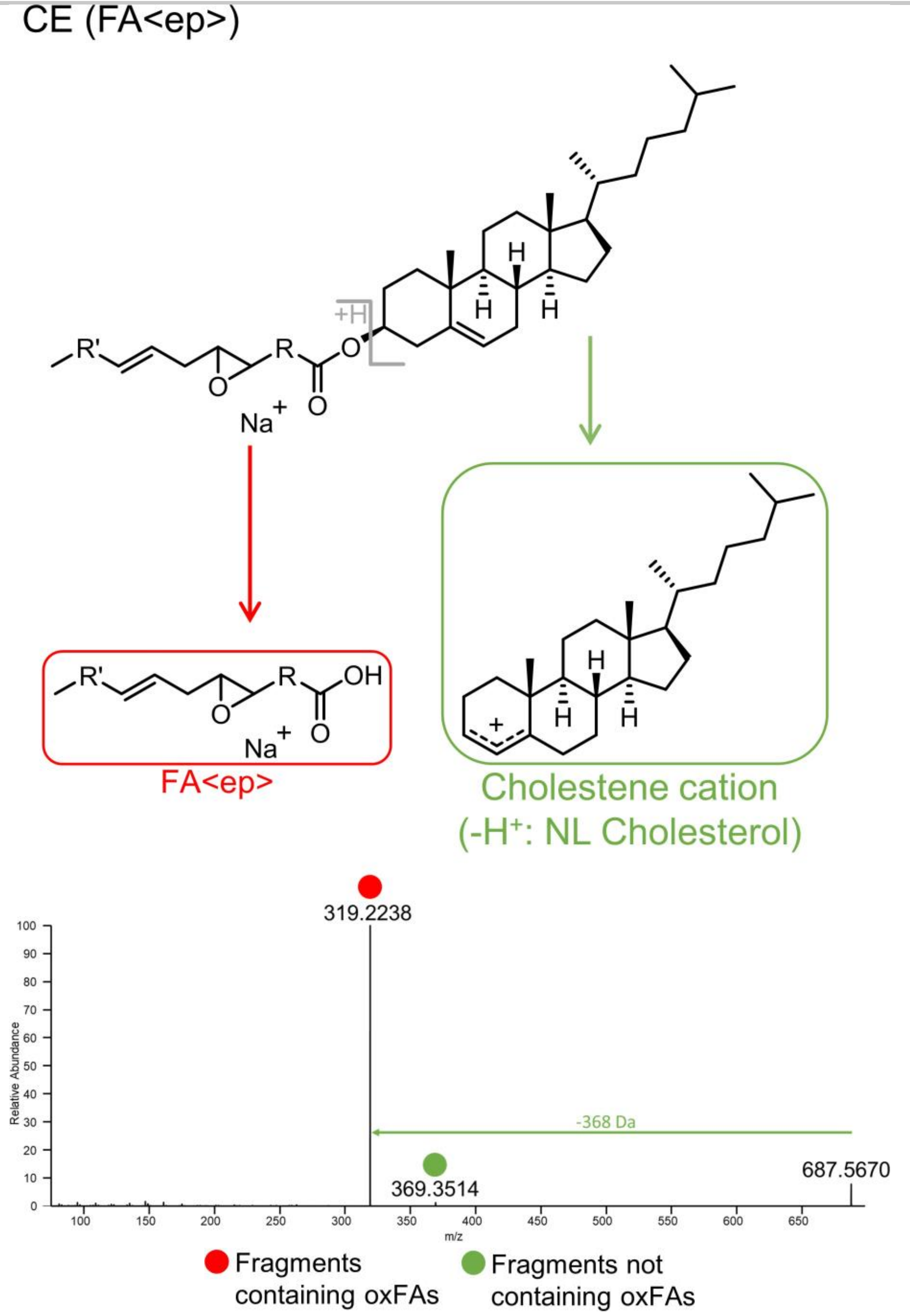	FA(18:0<ep>)	$C_{18}H_{34}O_3Na$	321.2400
**CE(18:1<ep>)**	$C_{45}H_{76}O_3Na$	687.5687		FA(18:1<ep>)	$C_{18}H_{32}O_3Na$	319.2244
**CE(20:3<ep>)**	$C_{47}H_{76}O_3Na$	711.5687		FA(20:3<ep>)	$C_{20}H_{32}O_3Na$	343.2244

Full-length oxygenated: <OOH>

$[M+Na]^+$ adducts

Precursor	Chemical Formula & *m/z*	MS² Fragmentation	Mod.-specific fragment	Chemical Formula & *m/z*	Pos.-specific fragment	Chemical Formula	*m/z*
**CE(18:1<OOH>)**	$C_{45}H_{78}O_4Na$ 705.5792	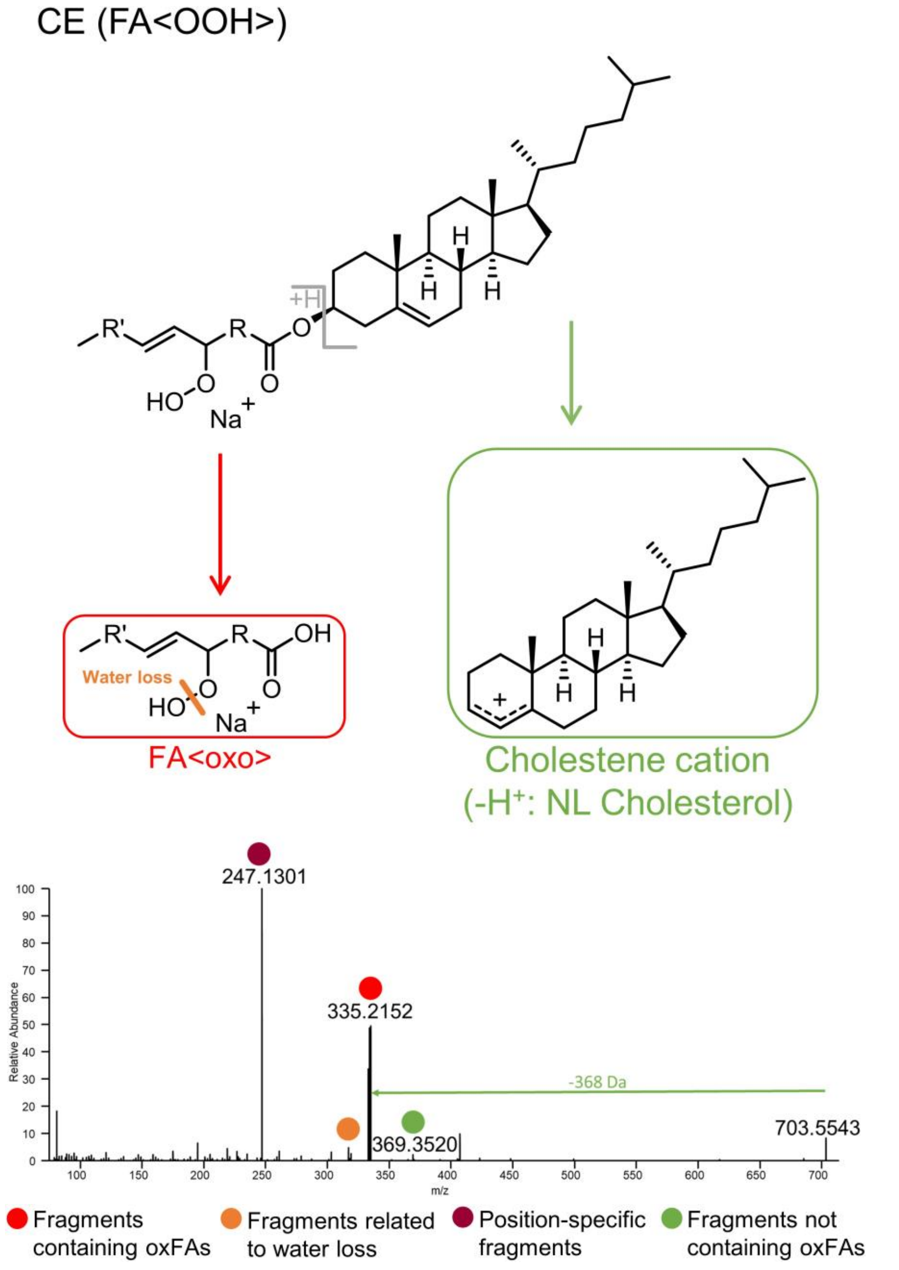 **FA fragmentation pattern:** 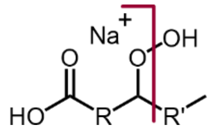	**FA(18:1<OOH>)** H₂O loss	**$C_{18}H_{34}O_4Na$ 337.2349** $C_{18}H_{32}O_3Na$ 319.2244	9 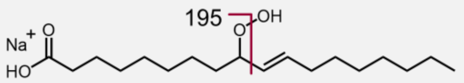 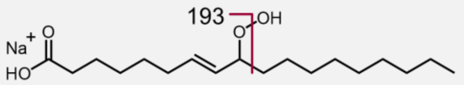	$C_9H_{16}O_3Na$<br>$C_9H_{14}O_3Na$	195.0992<br>193.0835
**CE(18:2<OOH>)**	$C_{45}H_{76}O_4Na$ 703.5636		**FA(18:2<OOH>)** H₂O loss	**$C_{18}H_{32}O_4Na$ 335.2193** $C_{18}H_{30}O_3Na$ 317.2087	9 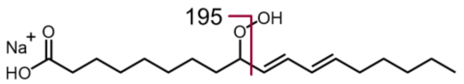	$C_9H_{16}O_3Na$	195.0992
					10 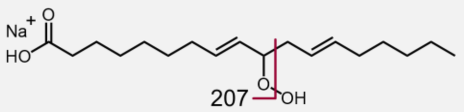	$C_{10}H_{16}O_3Na$	207.0992
					11 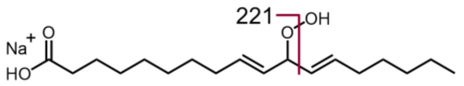	$C_{11}H_{18}O_3Na$	221.1148
					12 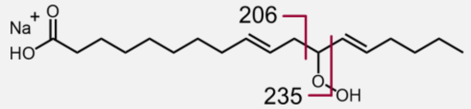	$C_{11}H_{19}O_2Na$<br>$C_{12}H_{20}O_3Na$	206.1277[10]<br>235.1305
					13 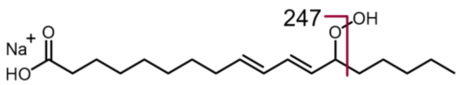	$C_{13}H_{20}O_3Na$	247.1305
**CE(20:4<OOH>)**	$C_{47}H_{76}O_4Na$ 727.5636		**FA(20:4<OOH>)** H₂O loss	**$C_{20}H_{32}O_4Na$ 359.2193** $C_{20}H_{30}O_3Na$ 341.2087	5 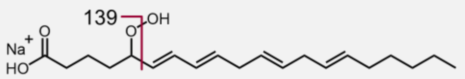	$C_5H_8O_3Na$	139.0366
					6 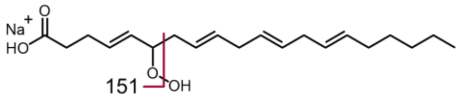	$C_6H_8O_3Na$	151.0366
					8 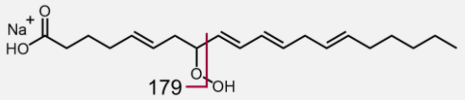	$C_8H_{12}O_3Na$	179.0679
					9 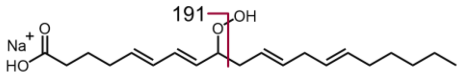	$C_9H_{12}O_3Na$	191.0679

Full-length oxygenated: <OOH> (continued)

$[M+Na]^+$ adducts

Precursor	Chemical Formula & *m/z*	MS² Fragmentation	Mod.-specific fragment	Chemical Formula & *m/z*	Pos.-specific fragment	Chemical Formula	*m/z*
**CE(20:4<OOH>)**	$C_{47}H_{76}O_4Na$ 727.5636		**FA(20:4<OOH>)** H₂O loss	**$C_{20}H_{32}O_4Na$ 359.2193** $C_{20}H_{30}O_3Na$ 341.2087	11 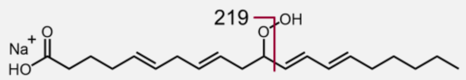	$C_{11}H_{16}O_3Na$	219.0992
					12 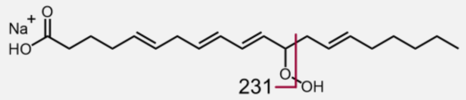	$C_{12}H_{16}O_3Na$	231.0992
					14 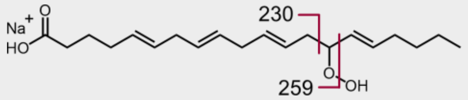	$C_{13}H_{19}O_2Na$ $C_{14}H_{20}O_3Na$	230.1277[9] 259.1305
					15 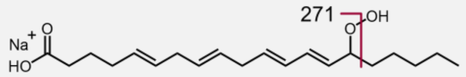	$C_{15}H_{20}O_3Na$	271.1305

[9] See *Ito, J., Mizuochi, S., Nakagawa, K., Kato, S., Miyazawa, T., 2015. Tandem Mass Spectrometry Analysis of Linoleic and Arachidonic Acid Hydroperoxides via Promotion of Alkali Metal Adduct Formation. Anal. Chem. 87, 4980–4987.*

TRIACYLGLYCEROLS

Oxidatively truncated: <COOH>

$[M+Na]^+$ adducts

Mod. position	Precursor	Chemical Formula	*m/z*	MS² Fragmentation	Mod./pos.-specific fragment	Chemical Formula	*m/z*
**4**	TG(16:0/16:0/4:0<COOH>)	$C_{39}H_{72}O_8Na$	691.5119	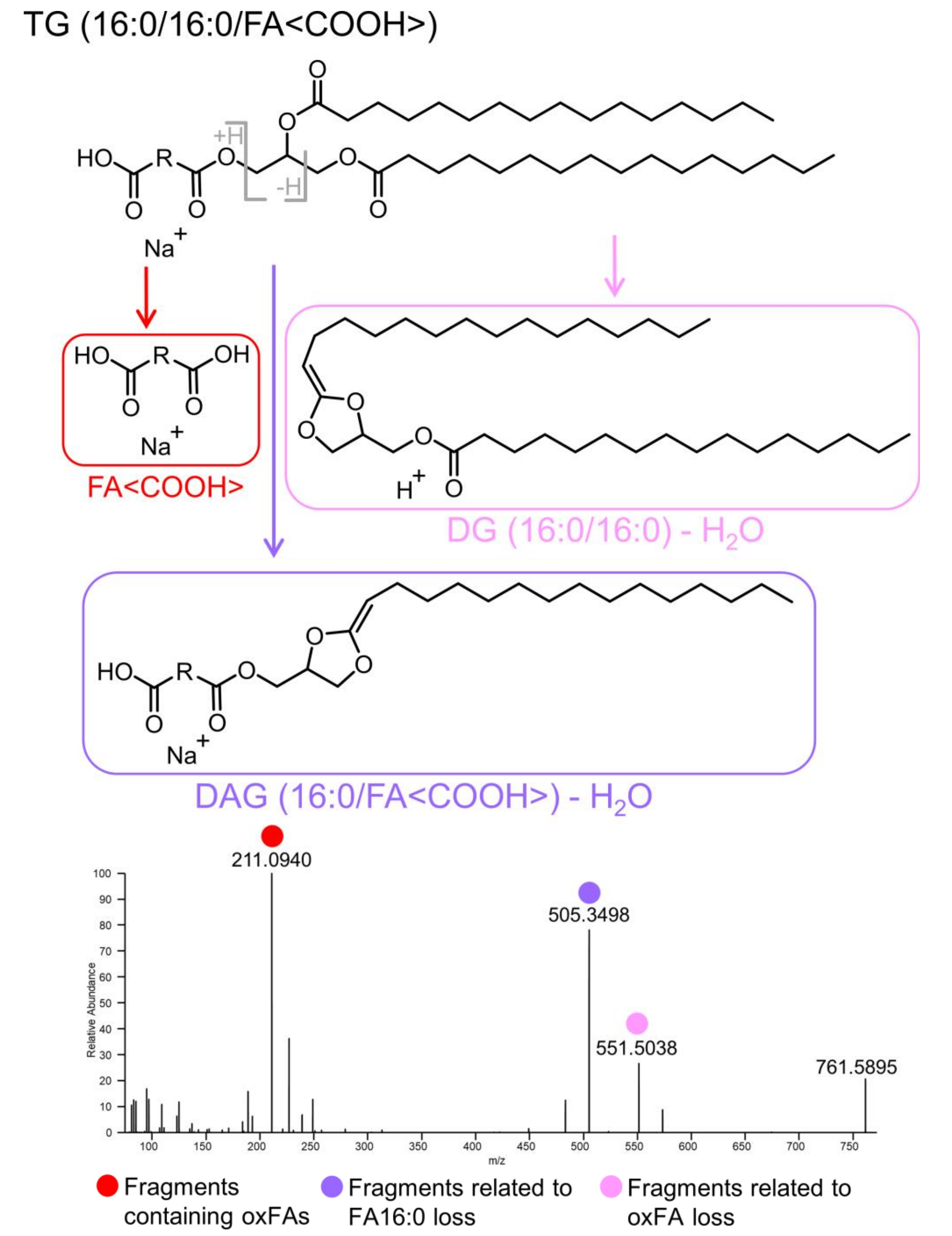	FA(4:0<COOH>)	$C_4H_6O_4Na$	141.0158
**5**	TG(16:0/16:0/5:0<COOH>)	$C_{40}H_{74}O_8Na$	705.5276		FA(5:0<COOH>)	$C_5H_8O_4Na$	155.0315
**7**	TG(16:0/16:0/7:0<COOH>)	$C_{42}H_{78}O_8Na$	733.5589		FA(7:0<COOH>)	$C_7H_{12}O_4Na$	183.0628
**8**	TG(16:0/16:0/8:0<COOH>)	$C_{43}H_{80}O_8Na$	747.5745		FA(8:0<COOH>)	$C_8H_{14}O_4Na$	197.0784
**9**	TG(16:0/16:0/9:0<COOH>)	$C_{44}H_{82}O_8Na$	761.5902		FA(9:0<COOH>)	$C_9H_{16}O_4Na$	211.0941
**10**	TG(16:0/16:0/10:0<COOH>)	$C_{45}H_{84}O_8Na$	775.6058		FA(10:0<COOH>)	$C_{10}H_{18}O_4Na$	225.1097
	TG(16:0/16:0/10:1<COOH>)	$C_{45}H_{82}O_8Na$	773.5902		FA(10:1<COOH>)	$C_{10}H_{16}O_4Na$	223.0941
**11**	TG(16:0/16:0/11:1<COOH>)	$C_{46}H_{84}O_8Na$	787.6058		FA(11:1<COOH>)	$C_{11}H_{18}O_4Na$	237.1097
	TG(16:0/16:0/11:2<COOH>)	$C_{46}H_{82}O_8Na$	785.5902		FA(11:2<COOH>)	$C_{11}H_{16}O_4Na$	235.0941
**12**	TG(16:0/16:0/12:1<COOH>)	$C_{47}H_{86}O_8Na$	801.6215		FA(12:1<COOH>)	$C_{12}H_{20}O_4Na$	251.1254
	TG(16:0/16:0/12:2<COOH>)	$C_{47}H_{84}O_8Na$	799.6058		FA(12:2<COOH>)	$C_{12}H_{18}O_4Na$	249.1097
**13**	TG(16:0/16:0/13:2<COOH>)	$C_{48}H_{86}O_8Na$	813.6215		FA(13:2<COOH>)	$C_{13}H_{20}O_4Na$	263.1254
	TG(16:0/16:0/13:3<COOH>)	$C_{48}H_{84}O_8Na$	811.6058		FA(13:3<COOH>)	$C_{13}H_{18}O_4Na$	261.1097
**14**	TG(16:0/16:0/14:3<COOH>)	$C_{49}H_{86}O_8Na$	825.6215		FA(14:3<COOH>)	$C_{14}H_{20}O_4Na$	275.1254

Oxidatively truncated: <oxo>

$[M+Na]^+$ adducts

Mod. position	Precursor	Chemical Formula	*m/z*	MS² Fragmentation	Mod./pos.-specific fragment	Chemical Formula	*m/z*
**4**	TG(16:0/16:0/4:0<oxo>)	$C_{39}H_{72}O_7Na$	675.5170	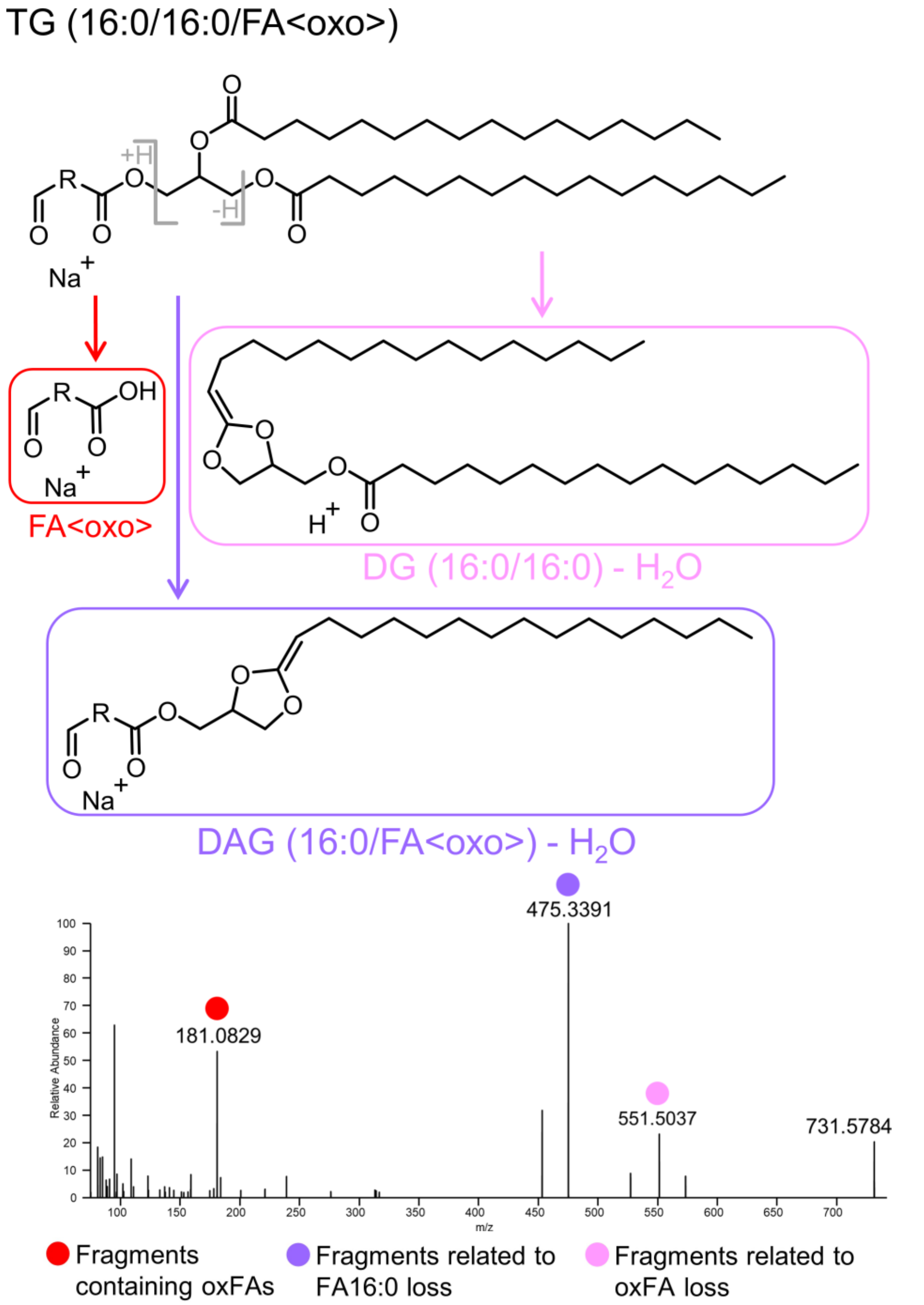	FA(4:0<oxo>)	$C_4H_6O_3Na$	125.0209
**5**	TG(16:0/16:0/5:0<oxo>)	$C_{40}H_{74}O_7Na$	689.5327		FA(5:0<oxo>)	$C_5H_8O_3Na$	139.0366
**7**	TG(16:0/16:0/7:0<oxo>)	$C_{42}H_{78}O_7Na$	717.5640		FA(7:0<oxo>)	$C_7H_{12}O_3Na$	167.0679
**8**	TG(16:0/16:0/8:0<oxo>)	$C_{43}H_{80}O_7Na$	731.5796		FA(8:0<oxo>)	$C_8H_{14}O_3Na$	181.0835
**9**	TG(16:0/16:0/9:0<oxo>)	$C_{44}H_{82}O_7Na$	745.5953		FA(9:0<oxo>)	$C_9H_{16}O_3Na$	195.0992
**10**	TG(16:0/16:0/10:0<oxo>)	$C_{45}H_{84}O_7Na$	759.6109		FA(10:0<oxo>)	$C_{10}H_{18}O_3Na$	209.1148
	TG(16:0/16:0/10:1<oxo>)	$C_{45}H_{82}O_7Na$	757.5953		FA(10:1<oxo>)	$C_{10}H_{16}O_3Na$	207.0992
**11**	TG(16:0/16:0/11:1<oxo>)	$C_{46}H_{84}O_7Na$	771.6109		FA(11:1<oxo>)	$C_{11}H_{18}O_3Na$	221.1148
	TG(16:0/16:0/11:2<oxo>)	$C_{46}H_{82}O_7Na$	769.5953		FA(11:2<oxo>)	$C_{11}H_{16}O_3Na$	219.0992
**12**	TG(16:0/16:0/12:1<oxo>)	$C_{47}H_{86}O_7Na$	785.6266		FA(12:1<oxo>)	$C_{12}H_{20}O_3Na$	235.1305
	TG(16:0/16:0/12:2<oxo>)	$C_{47}H_{84}O_7Na$	783.6109		FA(12:2<oxo>)	$C_{12}H_{18}O_3Na$	233.1148
**13**	TG(16:0/16:0/13:2<oxo>)	$C_{48}H_{86}O_7Na$	797.6266		FA(13:2<oxo>)	$C_{13}H_{20}O_3Na$	247.1305
	TG(16:0/16:0/13:3<oxo>)	$C_{48}H_{84}O_7Na$	795.6109		FA(13:3<oxo>)	$C_{13}H_{18}O_3Na$	245.1148
**14**	TG(16:0/16:0/14:3<oxo>)	$C_{49}H_{86}O_7Na$	809.6266		FA(14:3<oxo>)	$C_{14}H_{20}O_3Na$	259.1305

Full-length oxygenated: <oxo>

$[M+Na]^+$ adducts

Precursor	Chemical Formula	*m/z*	MS² Fragmentation	Mod.-specific fragment	Chemical Formula	*m/z*
**TG(16:0/16:0/18:1<oxo>)**	$C_{53}H_{98}O_7Na$	869.7205	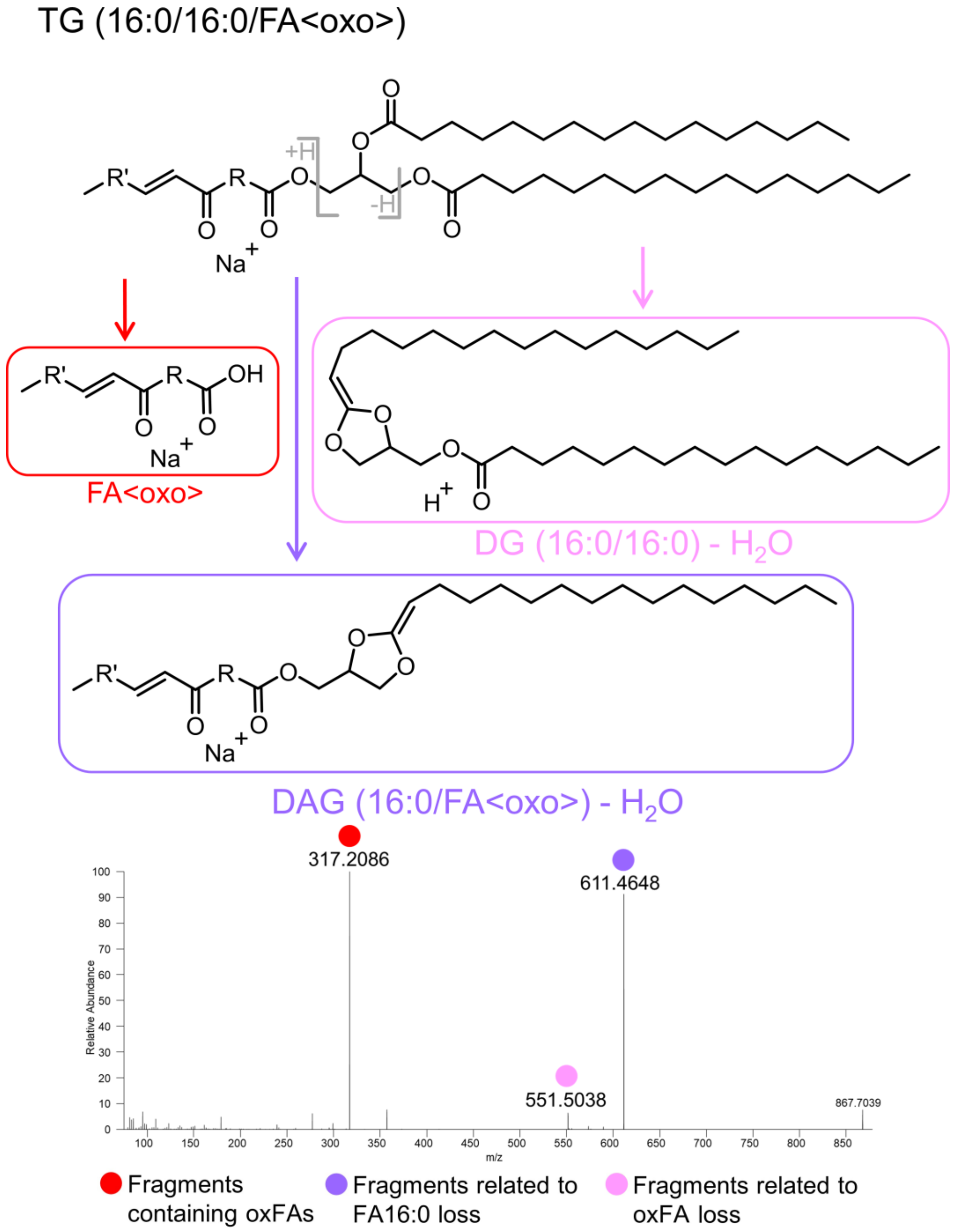	FA(18:1<oxo>)	$C_{18}H_{32}O_3Na$	319.2244
**TG(16:0/16:0/18:2<oxo>)**	$C_{53}H_{96}O_7Na$	867.7048		FA(18:2<oxo>)	$C_{18}H_{30}O_3Na$	317.2087
**TG(16:0/16:0/20:4<oxo>)**	$C_{55}H_{96}O_7Na$	891.7048		FA(20:4<oxo>)	$C_{20}H_{30}O_3Na$	341.2087

Full-length oxygenated: <OH>

$[M+Na]^+$ adducts

Precursor	Chemical Formula	*m/z*	MS² Fragmentation	Mod.-specific fragment	Chemical Formula	*m/z*
**TG(16:0/16:0/18:1<OH>)**	$C_{53}H_{100}O_7Na$	871.7361	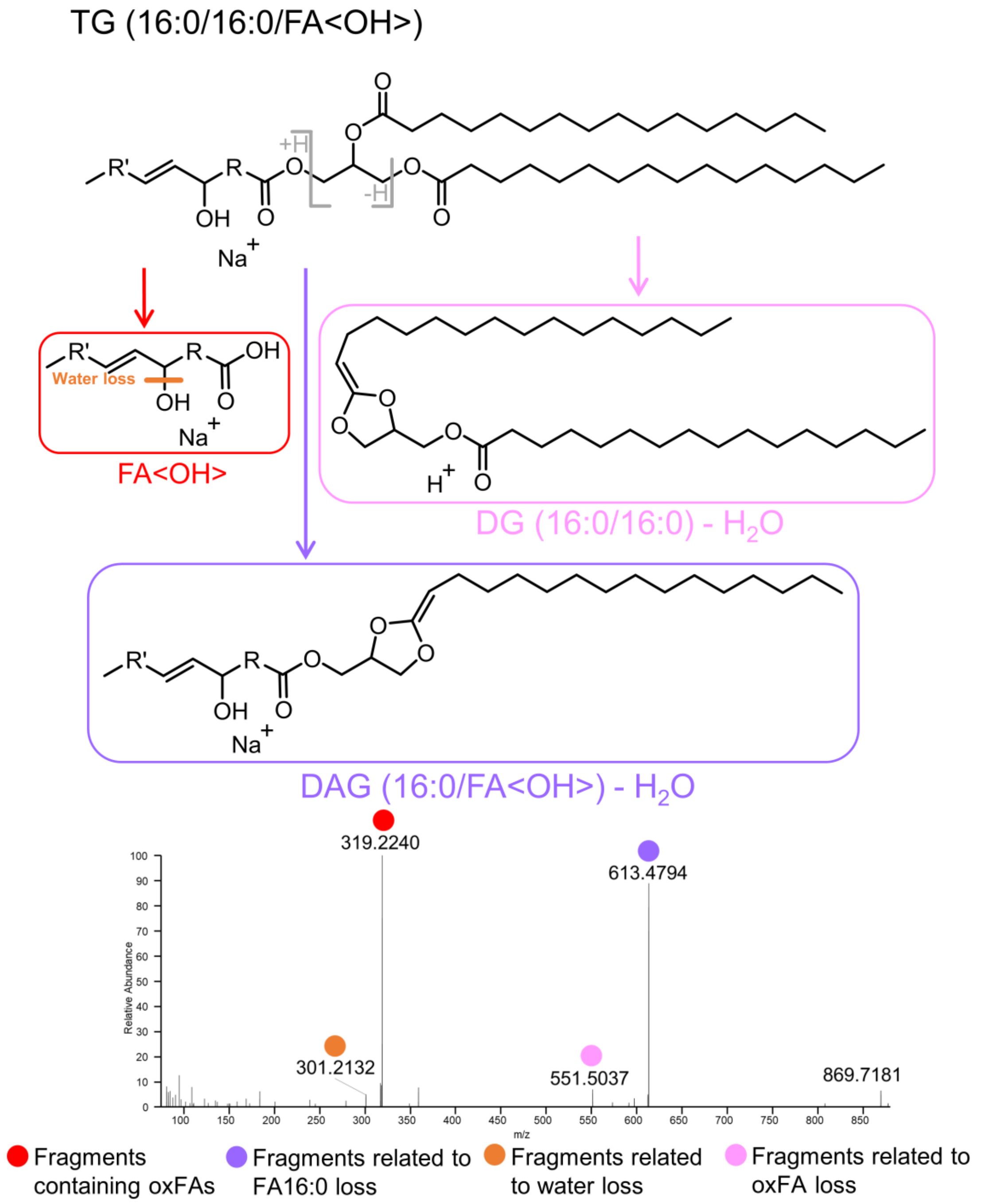	**FA(18:1<OH>)**<br>H_2O loss	**$C_{18}H_{34}O_3Na$**<br>$C_{18}H_{32}O_2Na$	**321.2400**<br>303.2295
**TG(16:0/16:0/18:2<OH>)**	$C_{53}H_{98}O_7Na$	869.7205		**FA(18:2<OH>)**<br>H_2O loss	**$C_{18}H_{32}O_3Na$**<br>$C_{18}H_{30}O_2Na$	**319.2244**<br>301.2138
**TG(16:0/16:0/20:4<OH>)**	$C_{55}H_{98}O_7Na$	893.7205		**FA(20:4<OH>)**<br>H_2O loss	**$C_{20}H_{32}O_3Na$**<br>$C_{20}H_{30}O_2Na$	**343.2244**<br>325.2138

Full-length oxygenated: <ep>

$[M+Na]^+$ adducts

Precursor	Chemical Formula	*m/z*	MS^2 Fragmentation	Mod.-specific fragment	Chemical Formula	*m/z*
**TG(16:0/16:0/18:0<ep>)**	$C_{53}H_{100}O_7Na$	871.7361	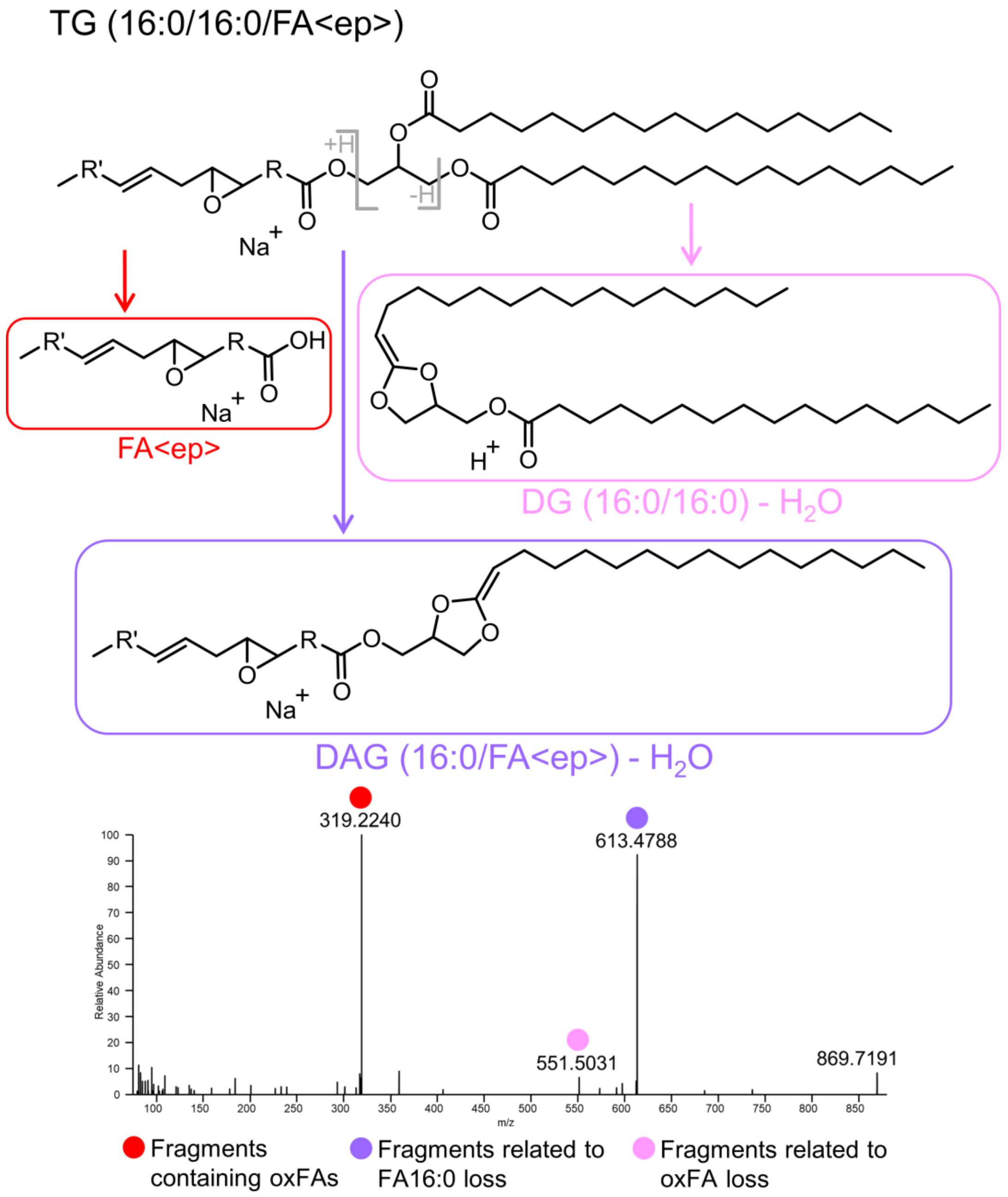	FA(18:0<ep>)	$C_{18}H_{34}O_3Na$	321.2400
**TG(16:0/16:0/18:1<ep>)**	$C_{53}H_{98}O_7Na$	869.7205		FA(18:1<ep>)	$C_{18}H_{32}O_3Na$	319.2244
**TG(16:0/16:0/20:3<ep>)**	$C_{55}H_{98}O_7Na$	893.7205		FA(20:3<ep>)	$C_{20}H_{32}O_3Na$	343.2244

Full-length oxygenated: <OOH>

$[M+Na]^+$ adducts

Precursor	Chemical Formula & *m/z*	MS^2 Fragmentation	Mod.-specific fragment	Chemical Formula & *m/z*	Pos.-specific fragment	Chemical Formula	*m/z*
**TG(16:0/16:0/18:1<OOH>)**	$C_{53}H_{100}O_8Na$ 887.7310	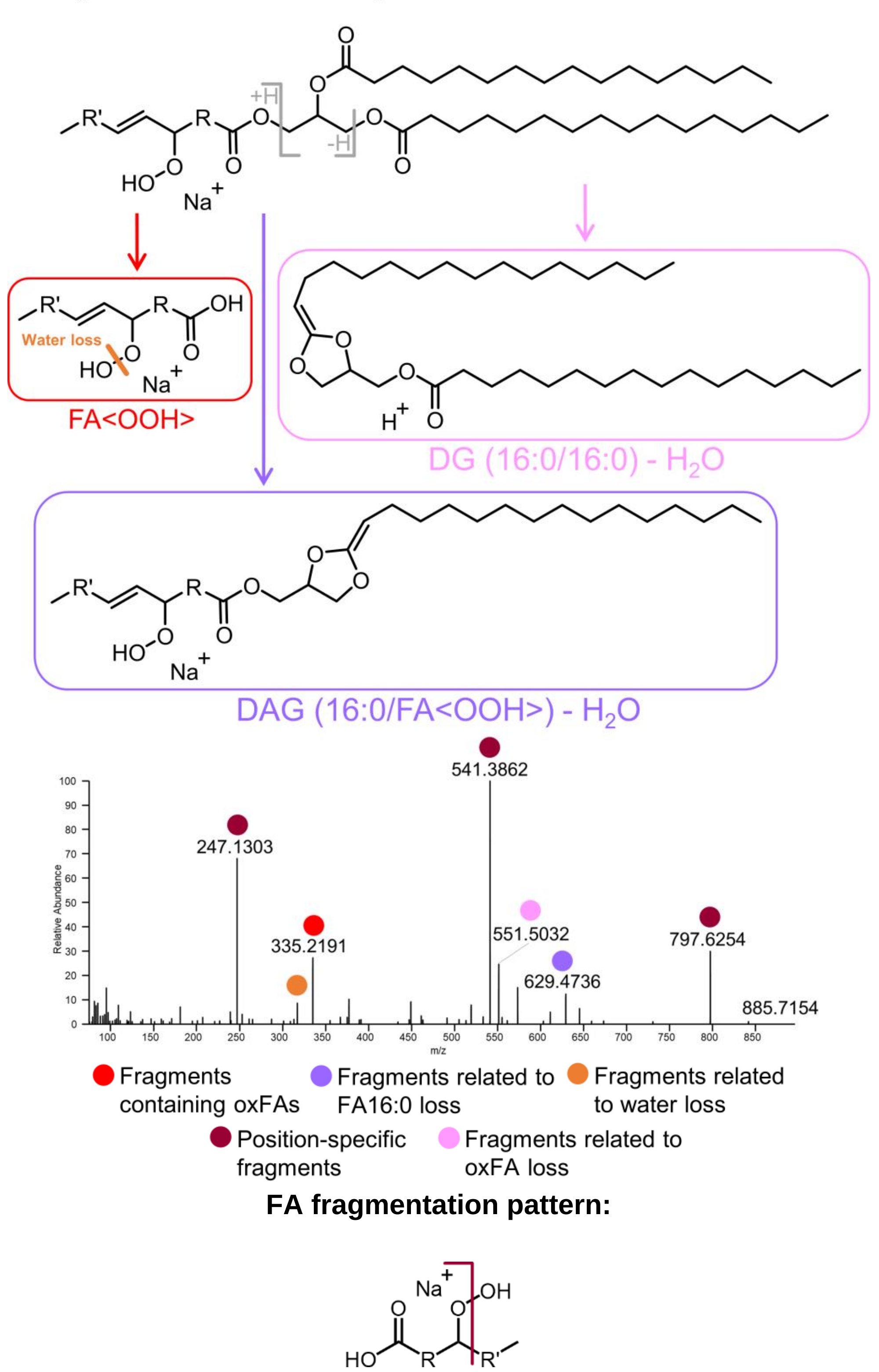	**FA(18:1<OOH>)**<br><br>H_2O loss	**$C_{18}H_{34}O_4Na$ 337.2349**<br>$C_{18}H_{32}O_3Na$ 319.2244	9 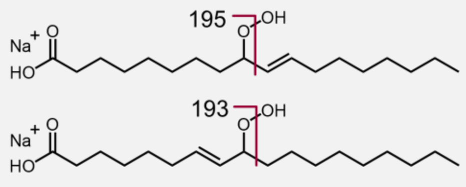	$C_9H_{16}O_3Na$<br>$C_9H_{14}O_3Na$	195.0992<br>193.0835
**TG(16:0/16:0/18:2<OOH>)**	$C_{53}H_{98}O_8Na$ 885.7154		**FA(18:2<OOH>)**<br><br>H_2O loss	**$C_{18}H_{32}O_4Na$ 335.2193**<br><br>$C_{18}H_{30}O_3Na$ 317.2087	9 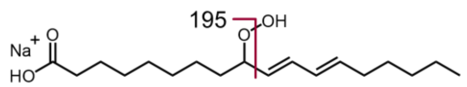	$C_9H_{16}O_3Na$	195.0992
					10 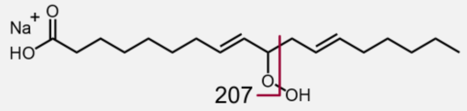	$C_{10}H_{16}O_3Na$	207.0992
					11 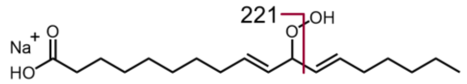	$C_{11}H_{18}O_3Na$	221.1148
					12 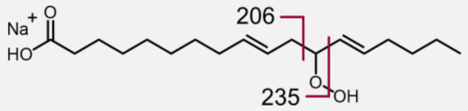	$C_{11}H_{19}O_2Na$<br>$C_{12}H_{20}O_3Na$	206.1277[11]<br>235.1305
					13 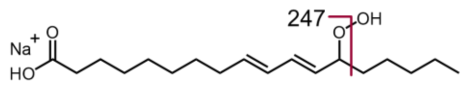	$C_{13}H_{20}O_3Na$	247.1305
**TG(16:0/16:0/20:4<OOH>)**	$C_{55}H_{98}O_8Na$ 909.7154		**FA(20:4<OOH>)**<br><br>H_2O loss	**$C_{20}H_{32}O_4Na$ 359.2193**<br><br>$C_{20}H_{30}O_3Na$ 341.2087	5 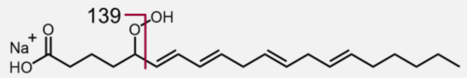	$C_5H_8O_3Na$	139.0366
					6 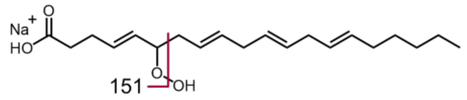	$C_6H_8O_3Na$	151.0366
					8 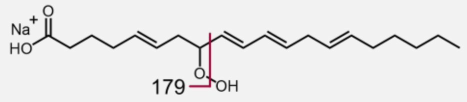	$C_8H_{12}O_3Na$	179.0679

Full-length oxygenated: <OOH> (continued)

$[M+Na]^+$ adducts

Precursor	Chemical Formula & *m/z*	MS² Fragmentation	Mod.-specific fragment	Chemical Formula & *m/z*	Pos.-specific fragment	Chemical Formula	*m/z*
**TG(16:0/16:0/20:4<OOH>)**	$C_{55}H_{98}O_8Na$ 909.7154		**FA(20:4<OOH>)**<br><br>H_2O loss	**$C_{20}H_{30}O_3Na$ 359.2193**<br><br>$C_{20}H_{32}O_4Na$ 341.2087	9 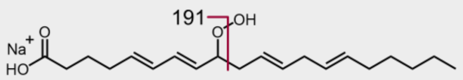	$C_9H_{12}O_3Na$	191.0679
					11 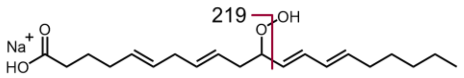	$C_{11}H_{16}O_3Na$	219.0992
					12 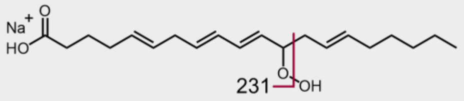	$C_{12}H_{16}O_3Na$	231.0992
					14 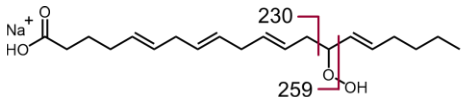	$C_{13}H_{19}O_2Na$<br>$C_{14}H_{20}O_3Na$	230.1277[10]<br>259.1305
					15 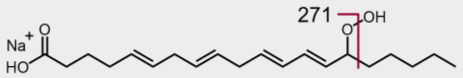	$C_{15}H_{20}O_3Na$	271.1305

[10] See *Ito, J., Mizuochi, S., Nakagawa, K., Kato, S., Miyazawa, T., 2015. Tandem Mass Spectrometry Analysis of Linoleic and Arachidonic Acid Hydroperoxides via Promotion of Alkali Metal Adduct Formation. Anal. Chem. 87, 4980–4987.*

REFERENCES

MS/MS for dioxygenated derivatives of FA(18:2) and FA(20:4) esterified into PC with some position-specific fragment ions indicated (negative ion mode):

- Matsuoka, Y., Takahashi, M., Sugiura, Y., Izumi, Y., Nishiyama, K., Nishida, M., Suematsu, M., Bamba, T., Yamada, K., 2021. Structural library and visualization of endogenously oxidized phosphatidylcholines using mass spectrometry-based techniques. Nat. Commun. 12, 6339. https://doi.org/10.1038/s41467-021-26633-w

MS/MS of the regioisomeric FA(20:4<OH>), FA(20:4<oxo>), FA(20:3<ep>), FA(20:4<OOH>) (negative ion mode):

- Murphy, R.C., 2015. Tandem mass spectrometry of lipids: molecular analysis of complex lipids, New developments in mass spectrometry. Royal Society of Chemistry, Cambridge.
- Murphy, R.C., Barkley, R.M., Zemski Berry, K., Hankin, J., Harrison, K., Johnson, C., Krank, J., McAnoy, A., Uhlson, C., Zarini, S., 2005. Electrospray ionization and tandem mass spectrometry of eicosanoids. Anal. Biochem. 346, 1–42. https://doi.org/10.1016/j.ab.2005.04.042

Characteristic ions in MS/MS of the regioisomeric FA(18:2<OH>), FA(18:2<oxo>), FA(20:4<OH>), FA(20:4<oxo>), FA(20:3<ep>) in FFA forms (negative ion mode):

- Derogis, P.B.M.C., Chaves-Fillho, A.B., Miyamoto, S., 2019. Characterization of Hydroxy and Hydroperoxy Polyunsaturated Fatty Acids by Mass Spectrometry, in: Trostchansky, A., Rubbo, H. (Eds.), Bioactive Lipids in Health and Disease, Advances in Experimental Medicine and Biology. Springer International Publishing, Cham, pp. 21–35. https://doi.org/10.1007/978-3-030-11488-6_2
- Levison, B.S., 2013. Quantification of fatty acid oxidation products using online high-performance liquid chromatography tandem mass spectrometry. Free Radic. Biol. Med. 12. https://doi.org/10.1016/j.freeradbiomed.2013.03.001

Characteristic ions in MS/MS of the regioisomeric FA(18:2<oxo>), FA(20:4<oxo>) (negative ion mode); use with caution, some fragmentation routes are questioned:

- Garscha, U., Nilsson, T., Oliw, E.H., 2008. Enantiomeric separation and analysis of unsaturated hydroperoxy fatty acids by chiral column chromatography-mass spectrometry. J. Chromatogr. B 872, 90–98. https://doi.org/10.1016/j.jchromb.2008.07.013

Characteristic ions in MS/MS of FA(18:2<OH{8}>) (negative ion mode):

- Garscha, U., Oliw, E.H., 2007. Steric analysis of 8-hydroxy- and 10-hydroxyoctadecadienoic acids and dihydroxyoctadecadienoic acids formed from 8R-hydroperoxyoctadecadienoic acid by hydroperoxide isomerases. Anal. Biochem. 367, 238–246. https://doi.org/10.1016/j.ab.2007.04.045

MS/MS of the regioisomeric FA(18:2<OOH>), FA(20:4<OOH>) (positive ion mode, $[M+Na]^+$ adducts):

- Ito, J., Mizuochi, S., Nakagawa, K., Kato, S., Miyazawa, T., 2015. Tandem Mass Spectrometry Analysis of Linoleic and Arachidonic Acid Hydroperoxides via Promotion of Alkali Metal Adduct Formation. Anal. Chem. 87, 4980–4987. https://doi.org/10.1021/acs.analchem.5b00851

Characteristic ions in MS/MS of the regioisomeric FA(18:2<OH>), FA(20:4<OH>), FA(20:3<ep>) in PC-esterified forms (negative ion mode):

- Aoyagi, R., Ikeda, K., Isobe, Y., Arita, M., 2017. Comprehensive analyses of oxidized phospholipids using a measured MS/MS spectra library. J. Lipid Res. 58, 2229–2237. https://doi.org/10.1194/jlr.D077123 (see also MS/MS in *.msp format: http://prime.psc.riken.jp/compms/msdial/download/msp/MSMS-Neg-RikenOxPLs.msp)

Characteristic ions in MS/MS of the regioisomeric FA(20:4<OH>) in PC-esterified forms (negative ion mode):

- Mazaleuskaya, L.L., Salamatipour, A., Sarantopoulou, D., Weng, L., FitzGerald, G.A., Blair, I.A., Mesaros, C., 2018. Analysis of HETEs in human whole blood by chiral UHPLC-ECAPCI/HRMS. J. Lipid Res. 59, 564–575. https://doi.org/10.1194/jlr.D081414

MS/MS of the regioisomeric FA(20:4<OH>) in FFA and PE-esterified forms (negative ion mode; see supplementary files there):

- Slatter, D.A., Aldrovandi, M., O'Connor, A., Allen, S.M., Brasher, C.J., Murphy, R.C., Mecklemann, S., Ravi, S., Darley-Usmar, V., O'Donnell, V.B., 2016. Mapping the Human Platelet Lipidome Reveals Cytosolic Phospholipase A2 as a Regulator of Mitochondrial Bioenergetics during Activation. Cell Metab. 23, 930–944. https://doi.org/10.1016/j.cmet.2016.04.001

LIPIDMAPS MS/MS spectra

(negative ionization mode, CE 30V)
https://www.lipidmaps.org/resources/standards/index.php?lipid_category=FA

Name / LIPIDMAPS ID
FA(18:1<ep{9-10}>) / LMFA02000037
FA(18:1<ep{12-13}>) / LMFA02000038
FA(18:2<oxo{13}>) / LMFA02000016
FA(18:2<OH{9}>) / LMFA02000036, LMFA02000188
FA(18:2<OH{13}>) / LMFA02000035, LMFA02000228
FA(18:2<OOH{9}>) / LMFA02000012
FA(18:2<OOH{13}>) / LMFA02000034
FA(20:3<ep{5-6}>) / LMFA03080002
FA(20:3<ep{8-9}>) / LMFA03080003
FA(20:3<ep{11-12}>) / LMFA03080004
FA(20:3<ep{14-15}>) / LMFA03080005
FA(20:4<oxo{5}>) / LMFA03060011
FA(20:4<oxo{12}>) / LMFA03060019
FA(20:4<OH{5}>) / LMFA03060002
FA(20:4<OH{8}>) / LMFA03060006
FA(20:4<OH{9}>) / LMFA03060089
FA(20:4<OH{11}>) / LMFA03060003
FA(20:4<OH{12}>) / LMFA03060007, LMFA03060008
FA(20:4<OH{15}>) / LMFA03060001
FA(20:4<OOH{5}>) / LMFA03060012
FA(20:4<OOH{12}>) / LMFA03060013
FA(20:4<OOH{15}>) / LMFA03060014

METLIN MS/MS spectra

(negative ion mode, CE 10, 20V)
https://metlin.scripps.edu/landing_page.php?pgcontent=mainPage

Name / METLIN ID

FA(18:0<ep{9-10}>) / 36008
FA(18:1<ep{9-10}>) / 43441
FA(18:1<ep{12-13}>) / 43442
FA(18:2<OH{9}>) / 35487, 45660, 45662
FA(18:2<OH{13}>) / 35490, 45665, 45667
FA(18:2<oxo{9}>) / 35860
FA(18:2<oxo{13}>) / 36023
FA(18:2<OOH{9}>) / 36019, 64785
FA(18:2<OOH{13}>) / 36036, 64784
FA(20:4<OH{5}>) / 36336, 45646, 45684
FA(20:4<OH{8}>) / 36286, 3840, 45730
FA(20:4<OH{9}>) / 36290, 45649, 45650
FA(20:4<OH{11}>) / 36337, 3838, 45056
FA(20:4<OH{12}>) / 3841, 45054, 45653
FA(20:4<OH{15}>) / 3836, 45651
FA(20:4<oxo{5}>) / 3844
FA(20:4<oxo{12}>) / 36285
FA(20:4<OOH{5}>) / 36281
FA(20:4<OOH{12}>) / 3845
FA(20:4<OOH{15}>) / 3846
