# Supplementary material for: Analytical and computational workflow for in-depth analysis of oxidized complex lipids in blood plasma
Source: Nat Commun. 2022 Nov 1;13:6547. doi: 10.1038/s41467-022-33225-9 (PMC9626469; doi:10.1038/s41467-022-33225-9)

Supplementary Data 6.

MS2 spectra acquired using stDDA and used for the annotation of **oxidized PC** (oxPC) species in group pooled blood plasma samples of obese non-diabetic (OND) and obese with type 2 diabetes (OT2D) individuals. Structure-related fragmentation ions are colour-codes according to the legend provided. Annotated lipids for each group pool are sorted by their precursor *m/z*.

OND_pool

PC(16:1_18:2<O>)
RT 12.0

[oxPC+HCOO]-   XIC 816.5396 NL: 2.08E3

- Fragments containing oxFAs
- Fragments related to water loss
- Fragments not containing oxFAs
- Fragments containing methylated oxFAs
- Fragments related to CO_2 loss
- Position-specific fragments

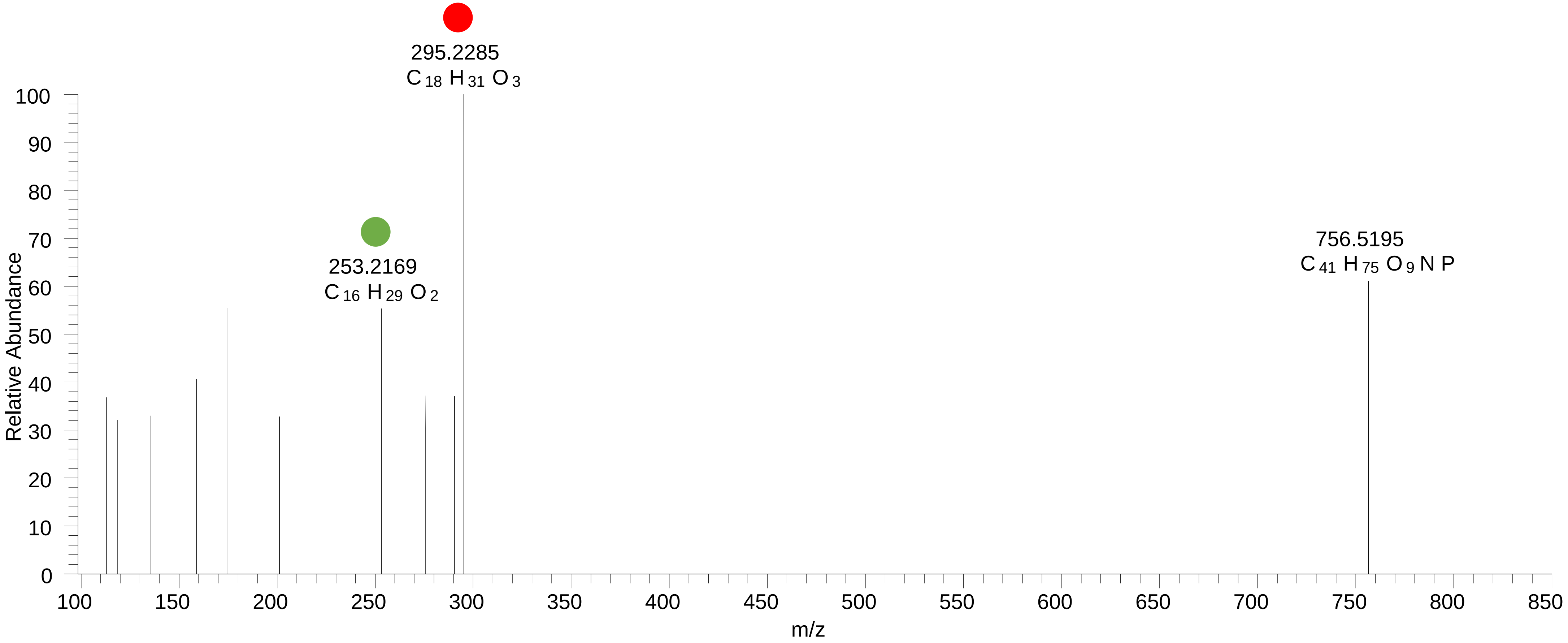

PC(16:0_18:3<O>)
RT 12.4

[oxPC+HCOO]- XIC 816.5396 NL: 1.10E3

Fragments containing oxFAs

Fragments related to water loss

Fragments not containing oxFAs

Fragments containing methylated oxFAs

Fragments related to CO_2 loss

Position-specific fragments

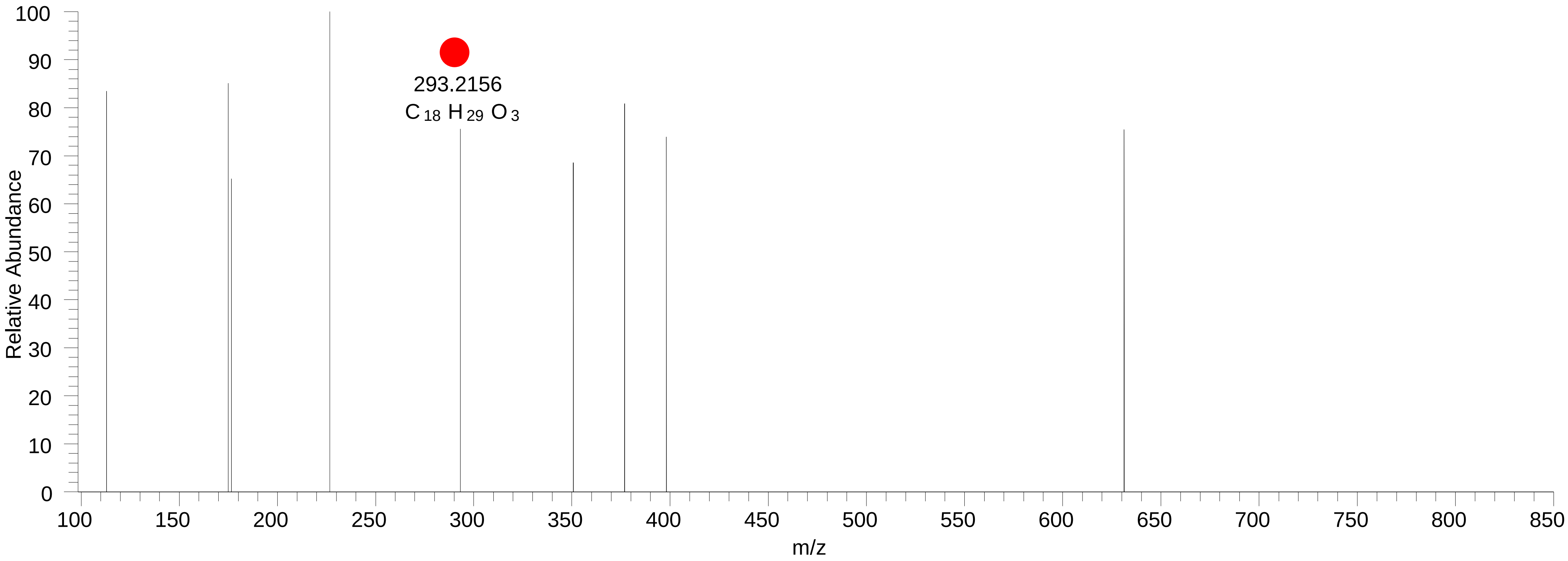

PC(16:0_18:2<OH>)
RT 12.6

[oxPC+HCOO]- XIC 818.5553 NL: 1.35E4

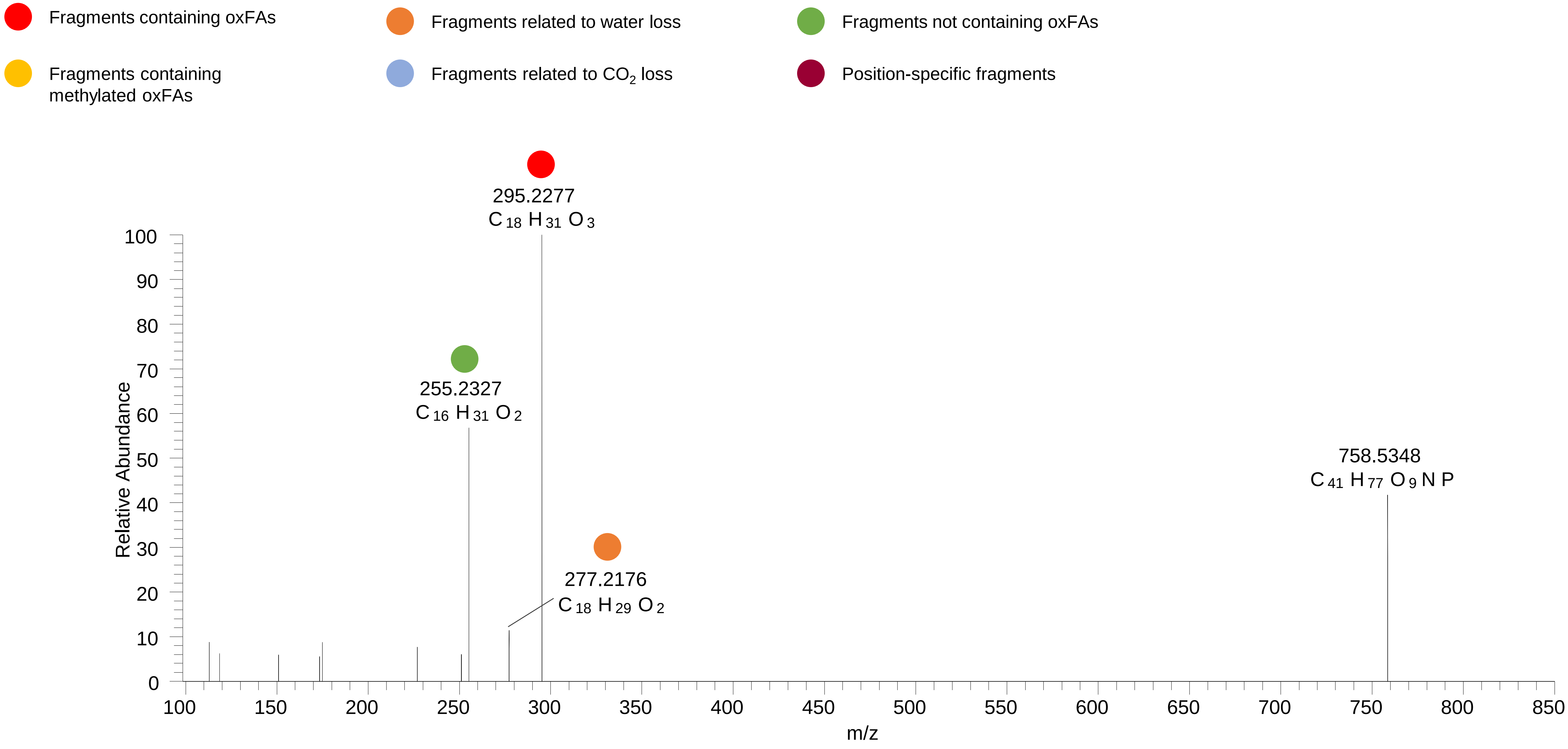

PC(16:0_18:2<OH{13}>)
RT 13.4

[oxPC+HCOO]- XIC 818.5553 NL: 9.74E4

Fragments containing oxFAs

Fragments related to water loss

Fragments not containing oxFAs

Fragments containing methylated oxFAs

Fragments related to CO_2 loss

Position-specific fragments

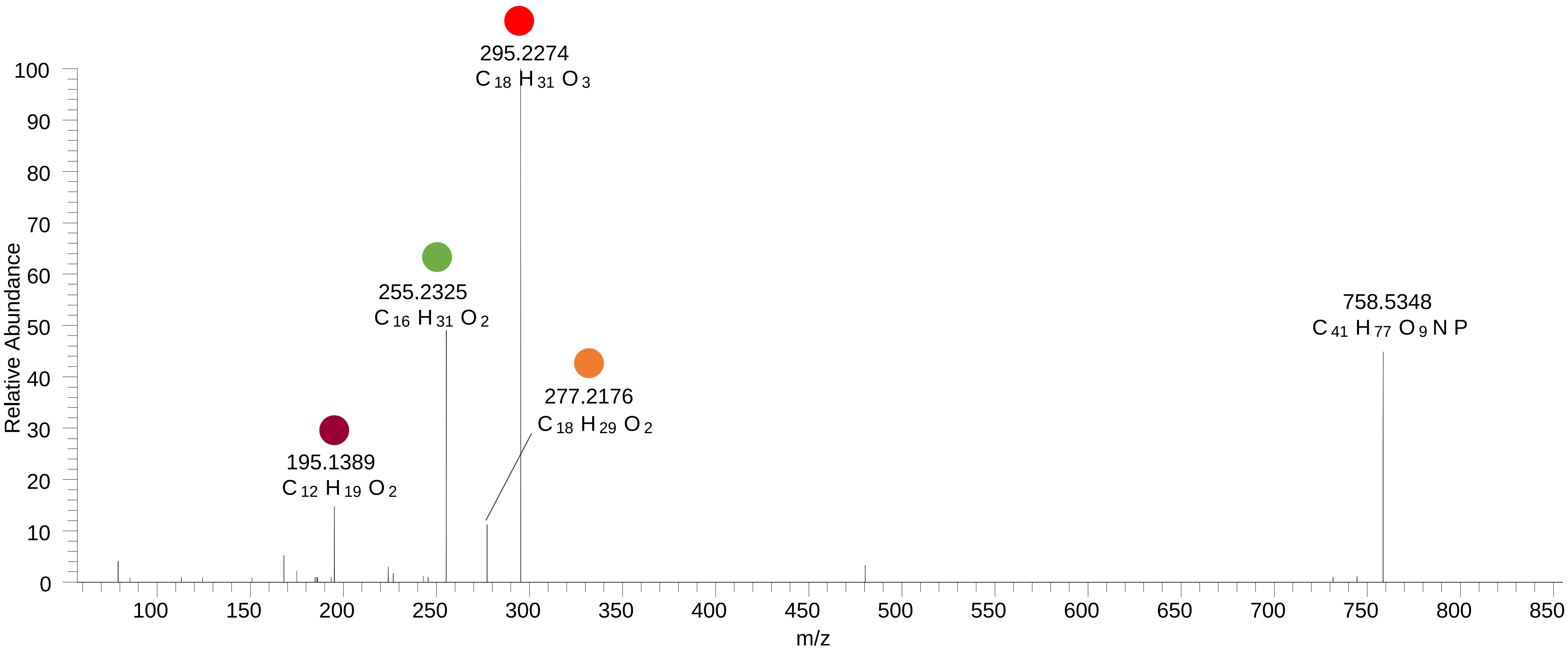

PC(16:0_18:2<OH{9}>)
RT 13.6

[oxPC+HCOO]- XIC 818.5553 NL: 9.27E4

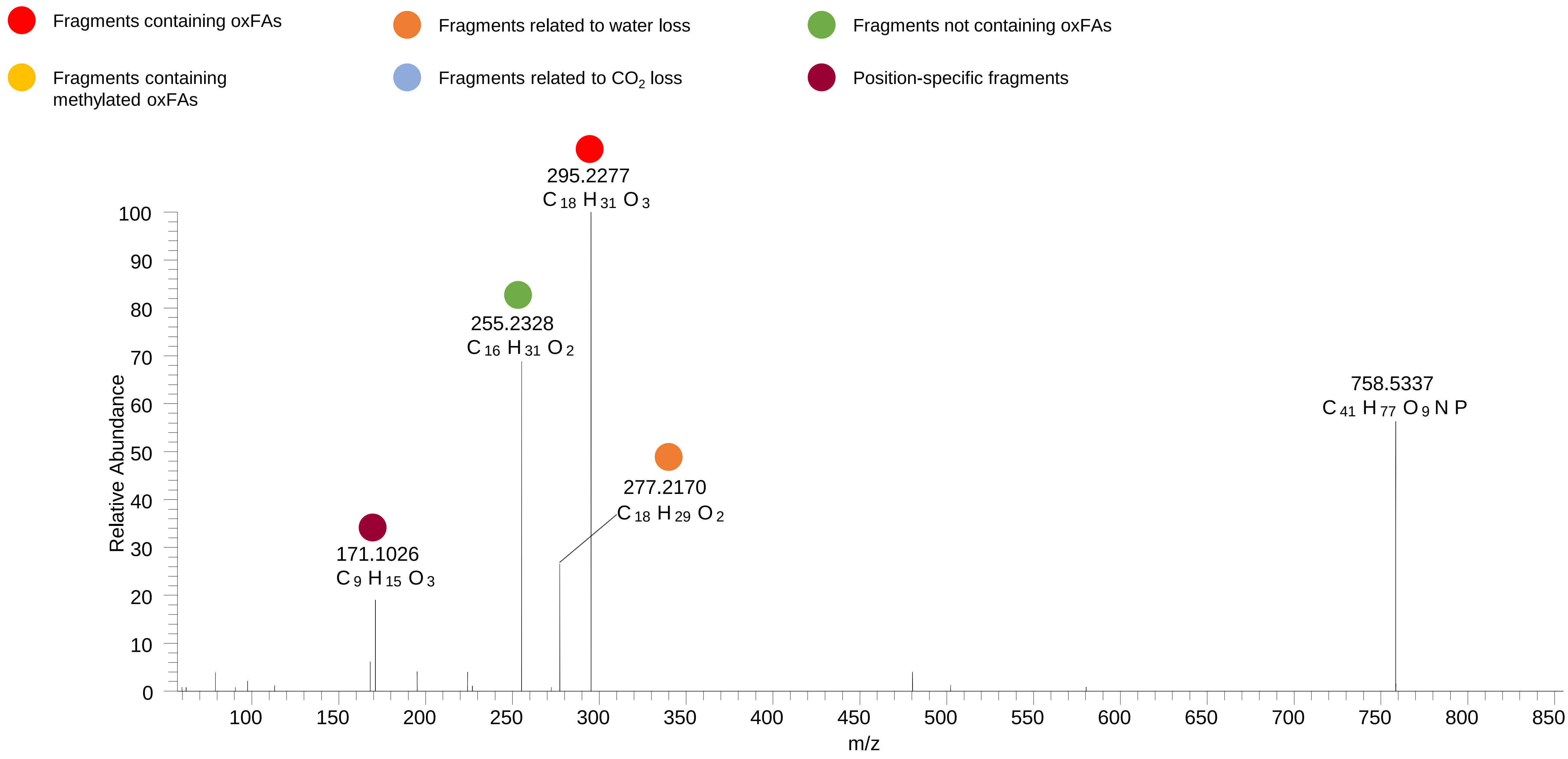

PC(16:0<O>_18:2)
RT 15.7

[oxPC+HCOO]- XIC 818.5553 NL: 6.25E4

- Fragments containing oxFAs
- Fragments related to water loss
- Fragments not containing oxFAs
- Fragments containing methylated oxFAs
- Fragments related to CO_2 loss
- Position-specific fragments

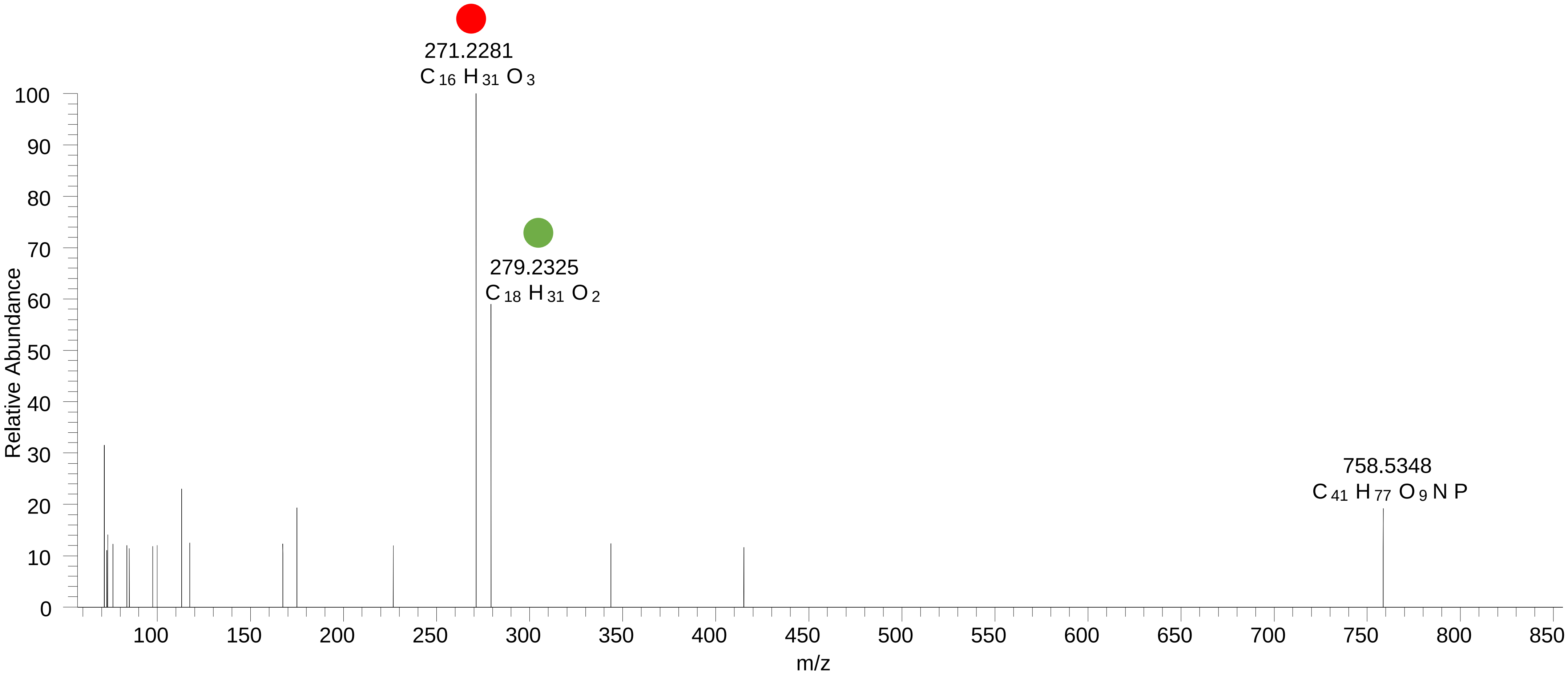

PC(16:0_18:1<O>)
RT 14.2

[oxPC+HCOO]- XIC 820.5709 NL: 6.89E3

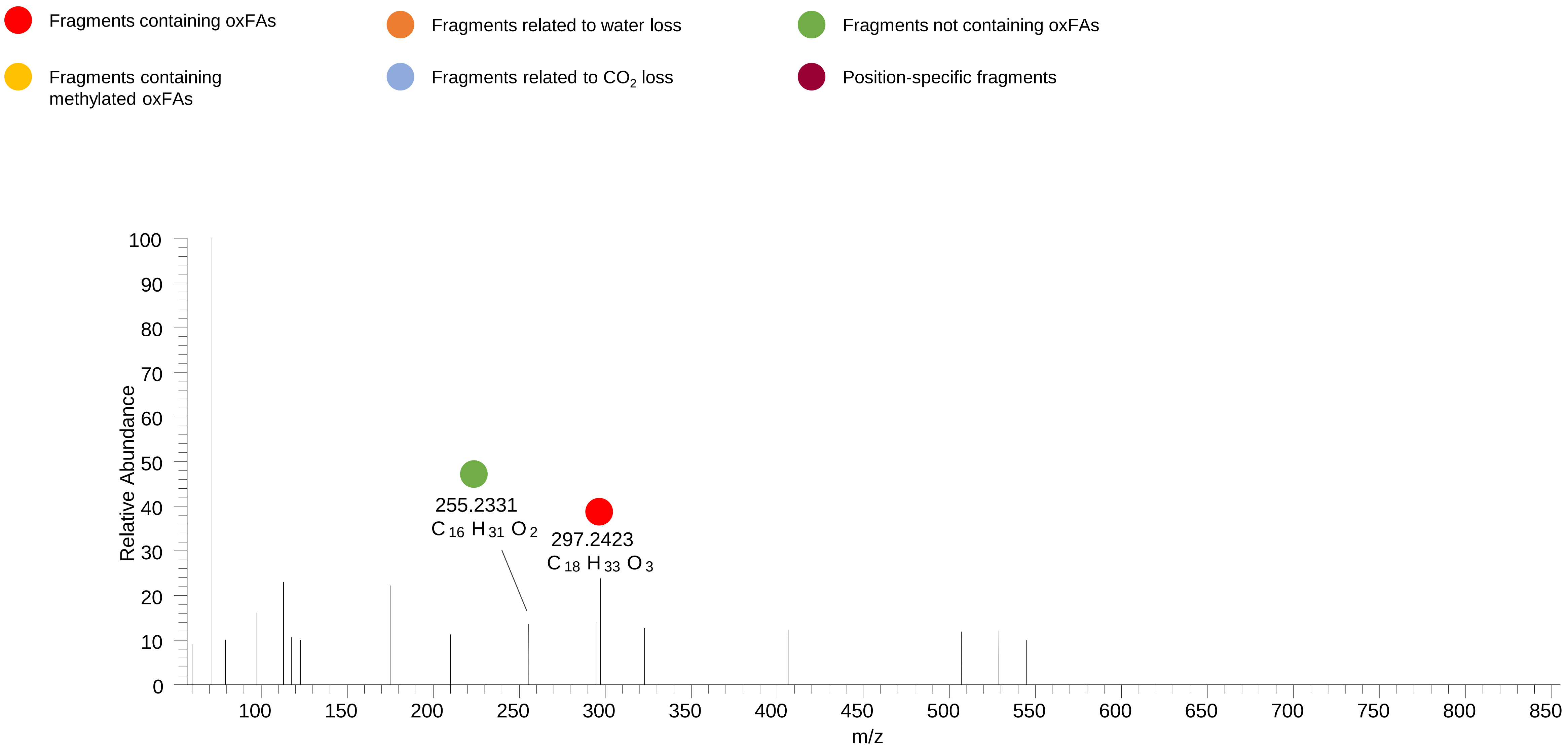

PC(16:0<O>_18:1)
RT 16.8-17.1

[oxPC+HCOO]- XIC 820.5709 NL: 5.36E3

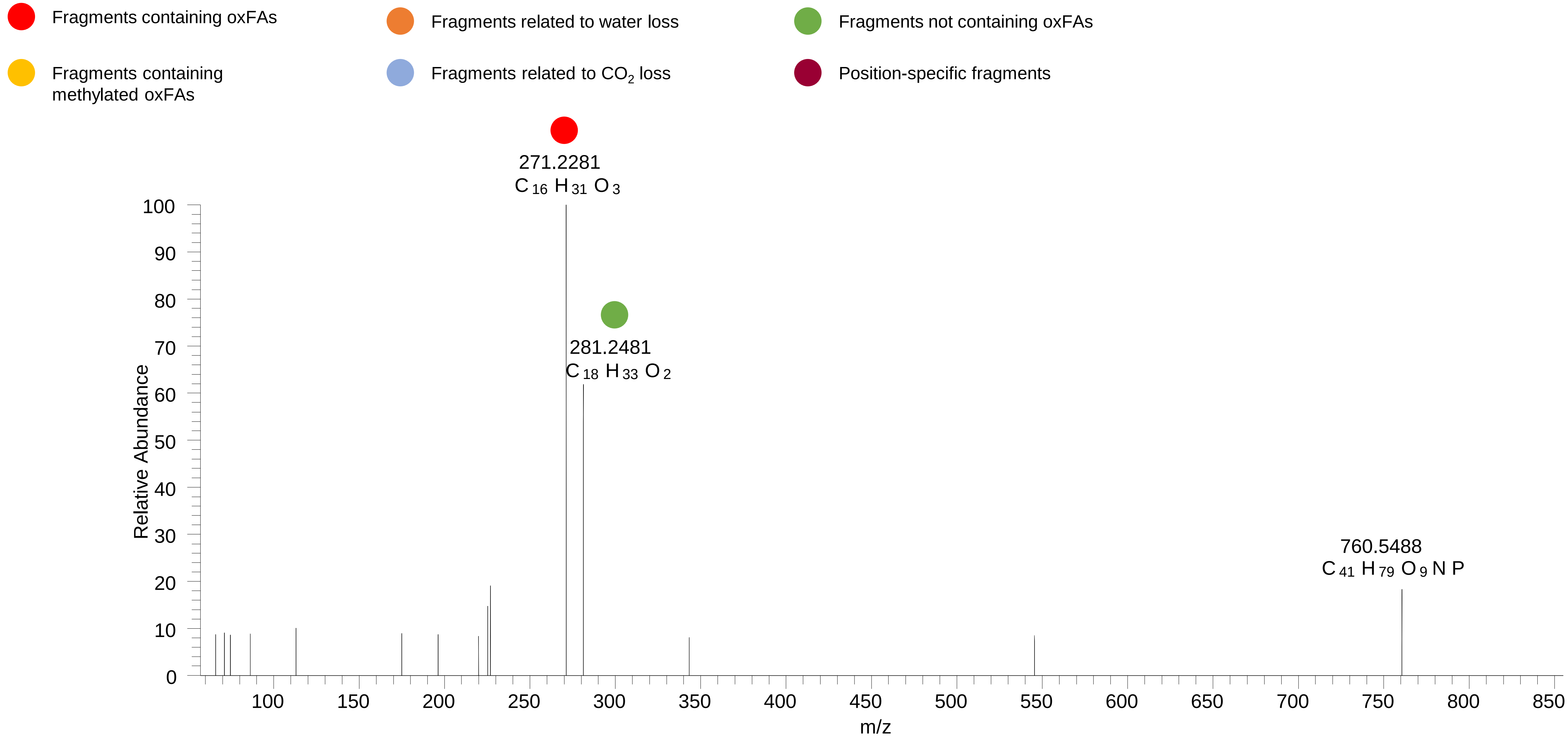

PC(16:0_18:2<OH,O{9}>)
RT 11.6

[oxPC+HCOO]- XIC 834.5502 NL: 3.30E3

- Fragments containing oxFAs
- Fragments related to water loss
- Fragments not containing oxFAs
- Fragments containing methylated oxFAs
- Fragments related to CO_2 loss
- Position-specific fragments

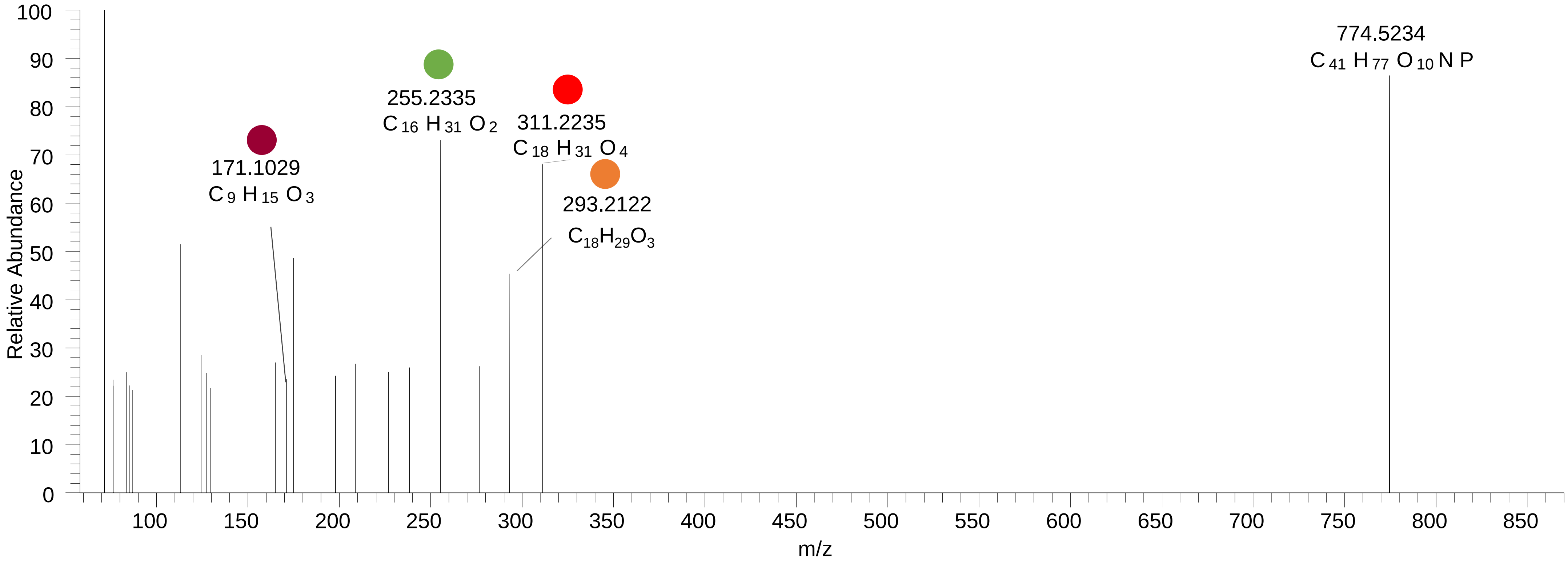

PC(16:0_18:2<OOH>)
RT 12.8

[oxPC+HCOO]- XIC 834.5502 NL: 4.70E3

- Fragments containing oxFAs
- Fragments related to water loss
- Fragments not containing oxFAs
- Fragments containing methylated oxFAs
- Fragments related to CO_2 loss
- Position-specific fragments

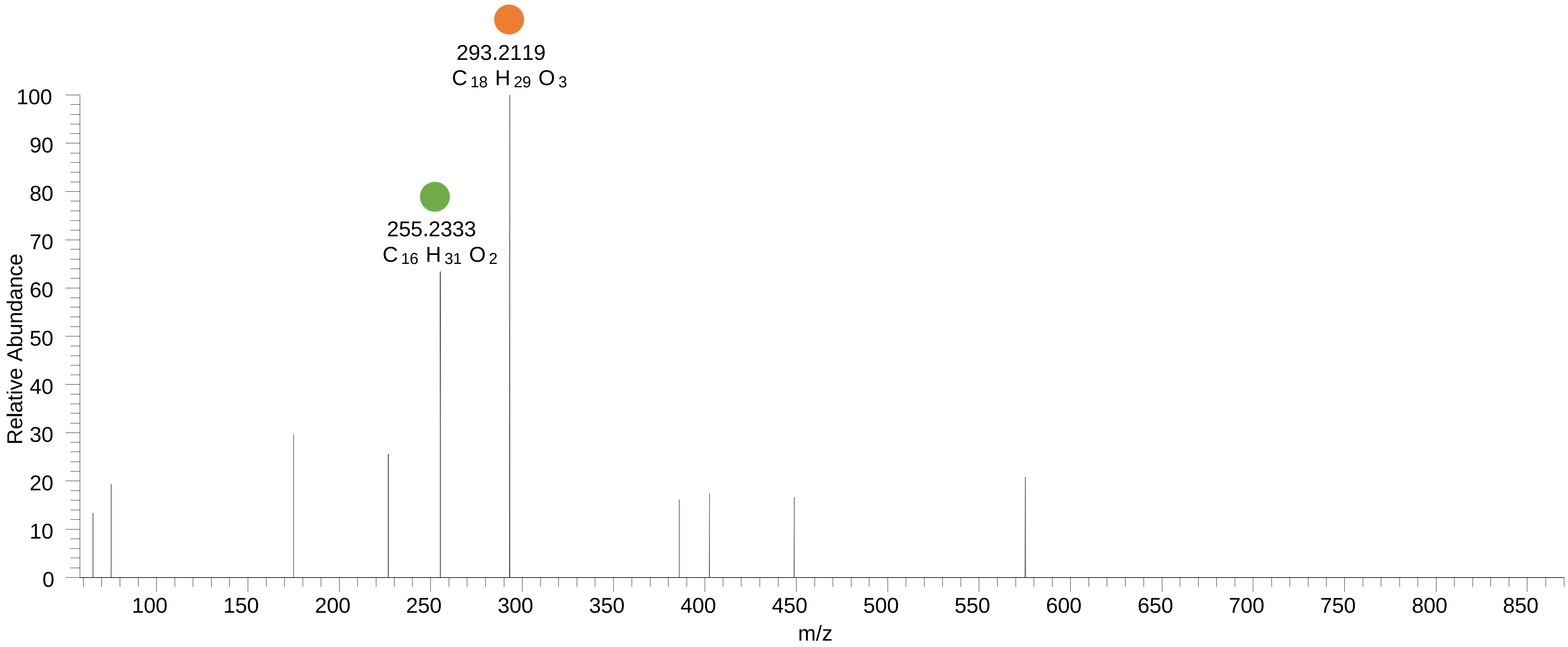

PC(16:0_18:2<OOH{13}>)
RT 13.1-13.4

[oxPC+HCOO]- XIC 834.5502 NL: 2.69E4

- Fragments containing oxFAs
- Fragments related to water loss
- Fragments not containing oxFAs
- Fragments containing methylated oxFAs
- Fragments related to CO_2 loss
- Position-specific fragments

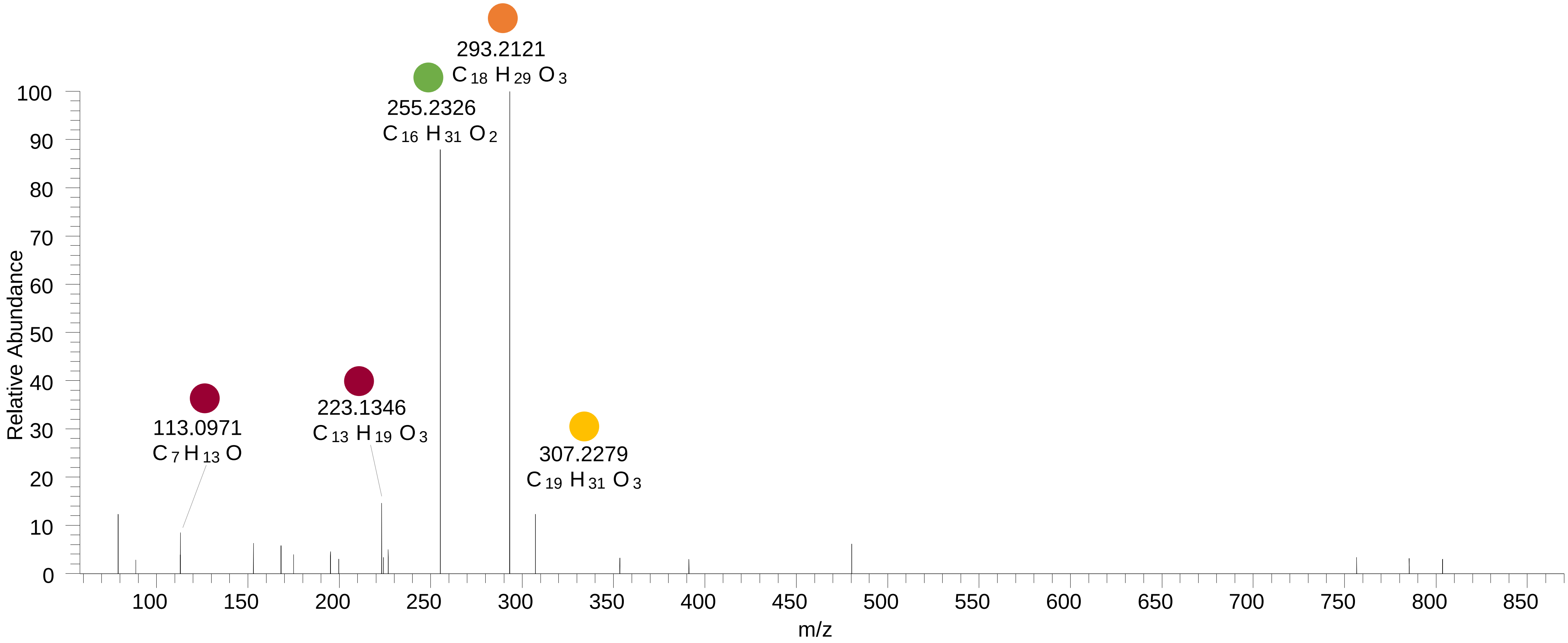

PC(16:0_18:2<OOH{9}>)
RT 13.6

[oxPC+HCOO]- XIC 834.5502 NL: 1.93E4

- Fragments containing oxFAs
- Fragments related to water loss
- Fragments not containing oxFAs
- Fragments containing methylated oxFAs
- Fragments related to CO_2 loss
- Position-specific fragments

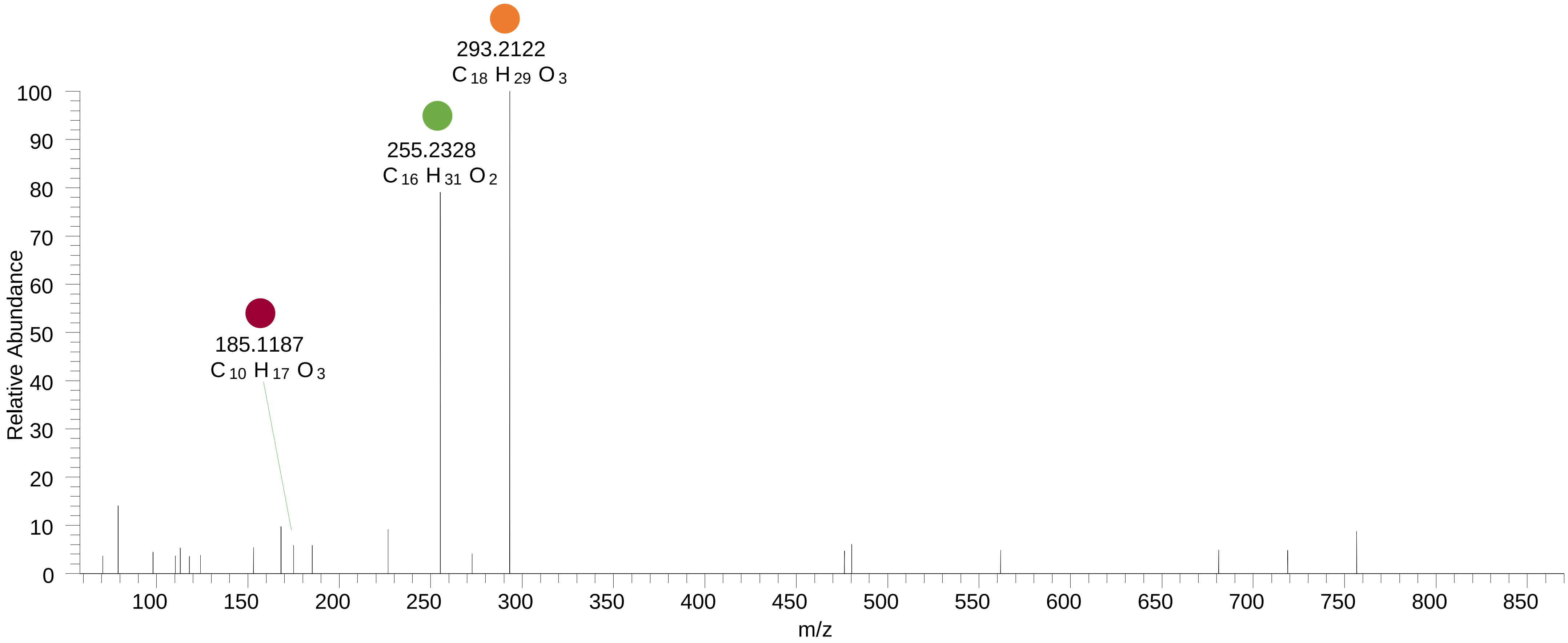

PC(16:0_18:1<OOH>)
RT 14.3

[oxPC+HCOO]- XIC 836.5658 NL: 5.31E3

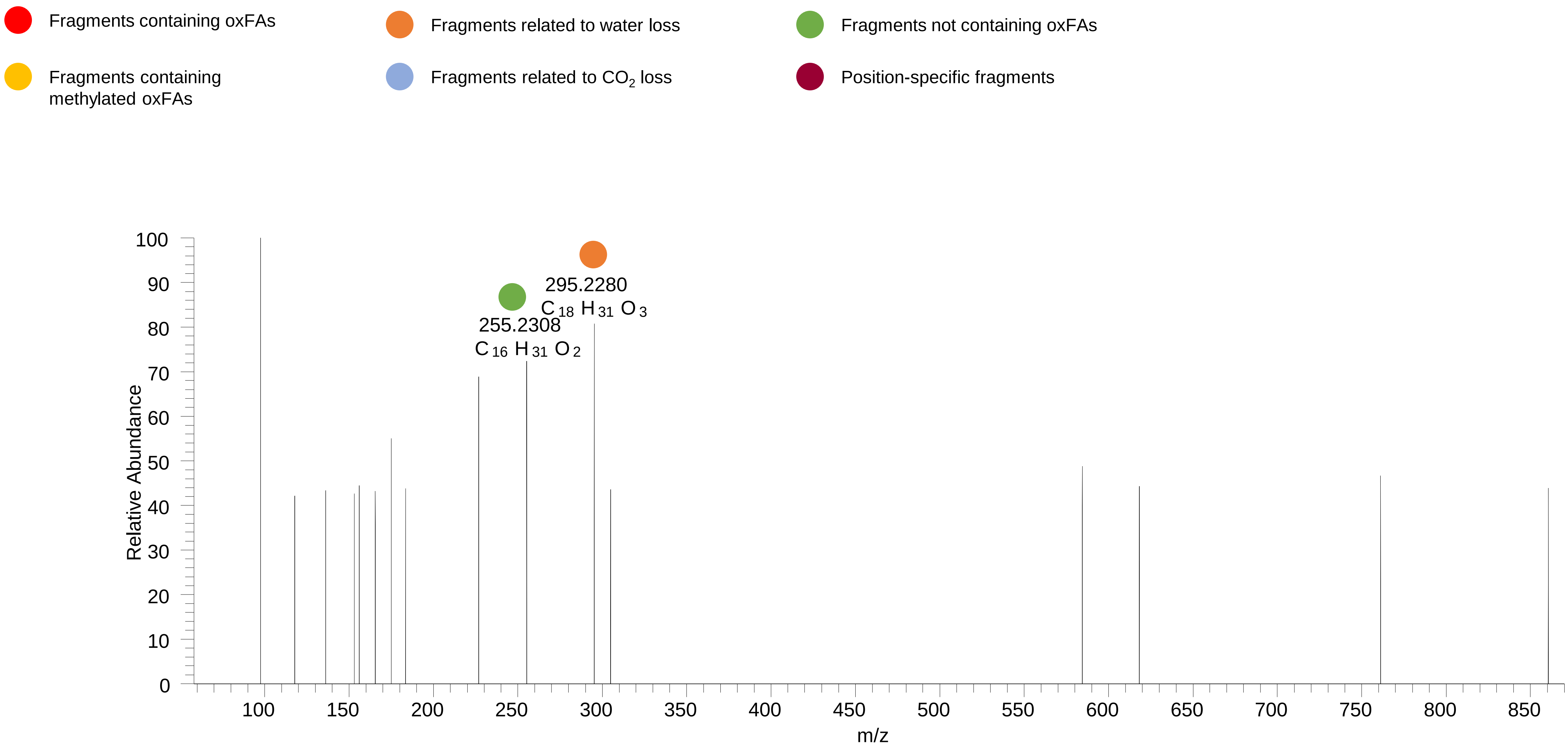

PC(16:0_20:5<O>)
RT 12.6-13.0

[oxPC+HCOO]- XIC 840.5396 NL: 2.93E3

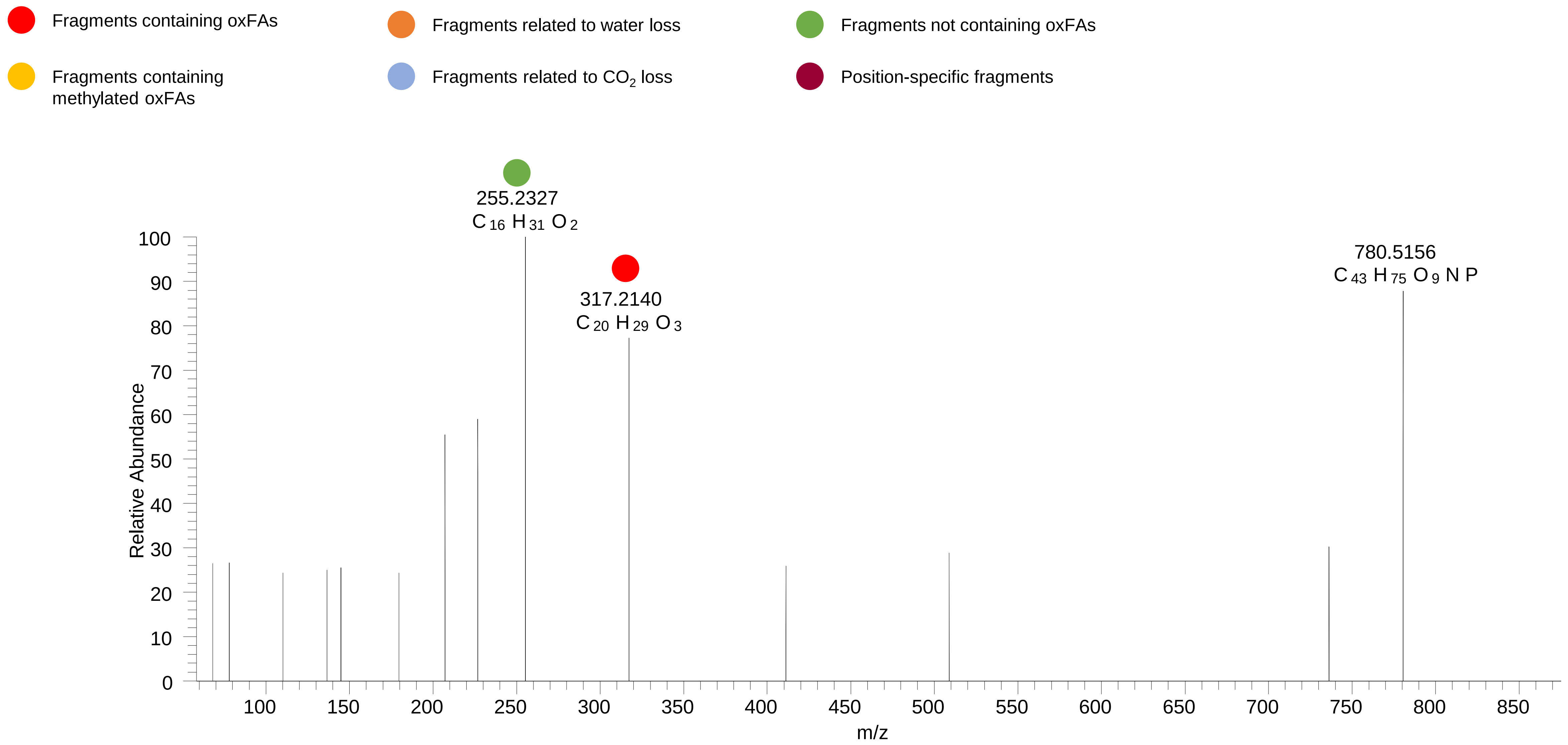

PC(18:2_18:2<OH>)
RT 12.5

[oxPC+HCOO]- XIC 842.5553 NL: 2.80E3

- Fragments containing oxFAs
- Fragments related to water loss
- Fragments not containing oxFAs
- Fragments containing methylated oxFAs
- Fragments related to CO_2 loss
- Position-specific fragments

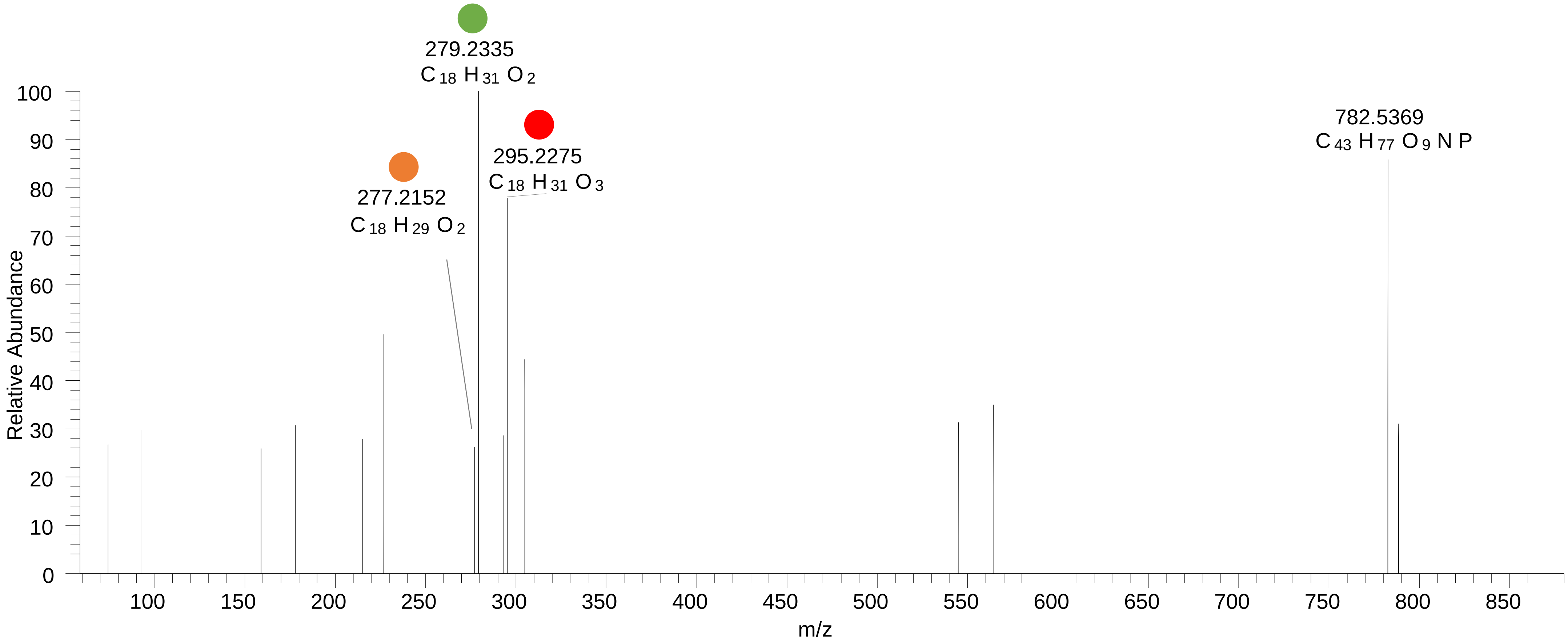

PC(16:0_20:4<OH{13}>)
RT 13.2

[oxPC+HCOO]- XIC 842.5553 NL: 5.46E3

- Fragments containing oxFAs
- Fragments related to water loss
- Fragments not containing oxFAs
- Fragments containing methylated oxFAs
- Fragments related to CO_2 loss
- Position-specific fragments

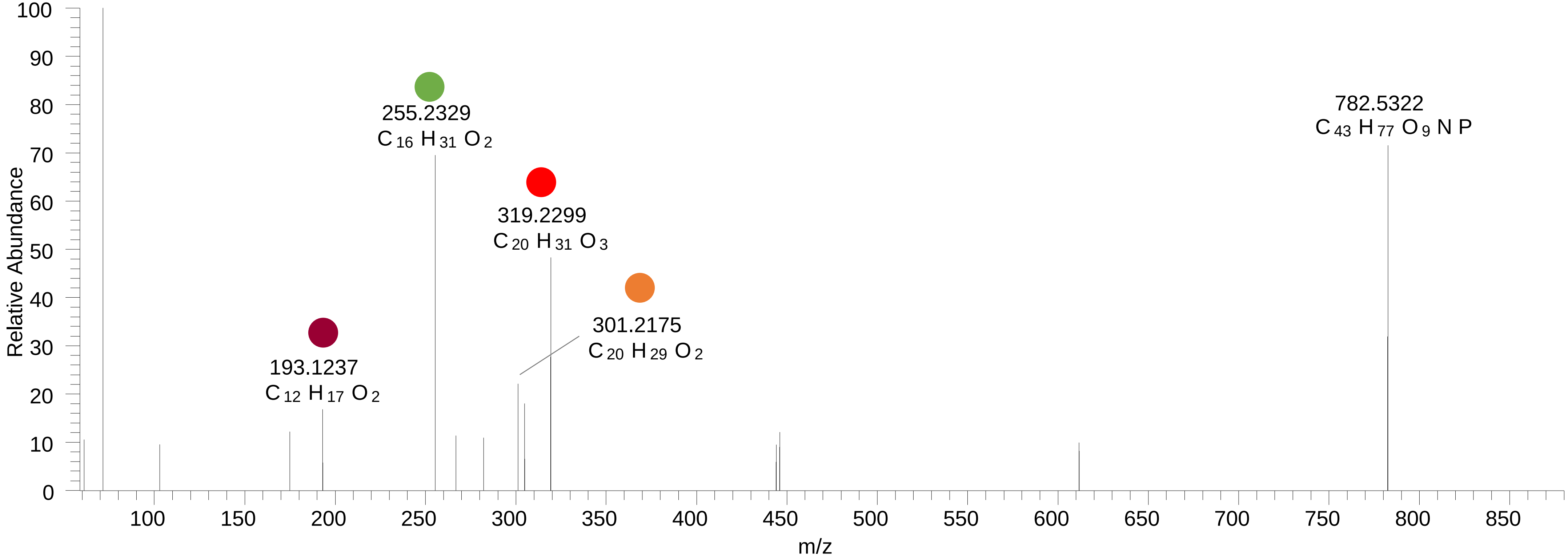

PC(16:0_20:4<OH{15}>)
RT 13.6

[oxPC+HCOO]- XIC 842.5553 NL: 2.08E4

- Fragments containing oxFAs
- Fragments related to water loss
- Fragments not containing oxFAs
- Fragments containing methylated oxFAs
- Fragments related to CO_2 loss
- Position-specific fragments

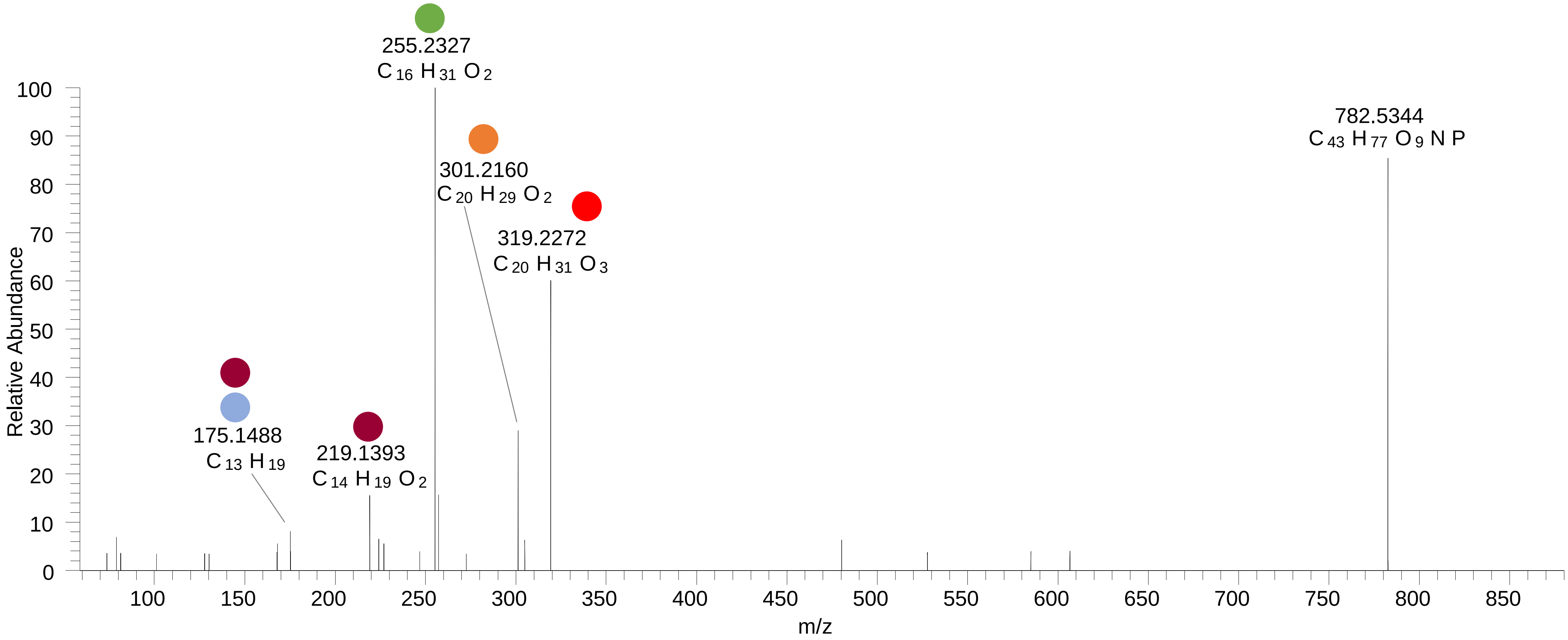

PC(16:0_20:4<OH{11}>)
PC(16:0_20:4<OH{12}>)
RT 14.0

[oxPC+HCOO]- XIC 842.5553 NL: 2.87E4

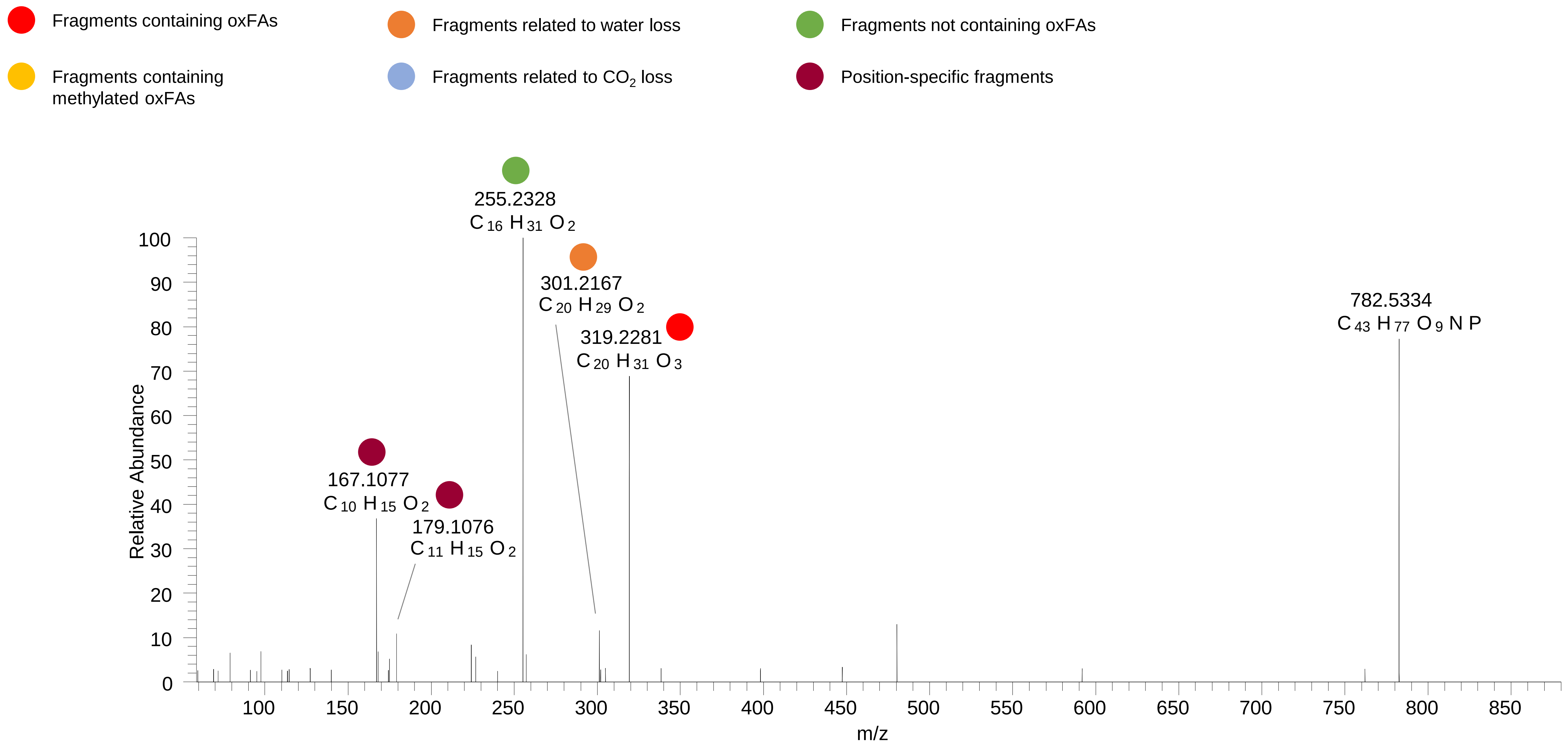

PC(16:0_20:4<OH{8}>)
PC(16:0_20:4<OH{9}>)
RT 14.5

[oxPC+HCOO]- XIC 842.5553 NL: 3.17E4

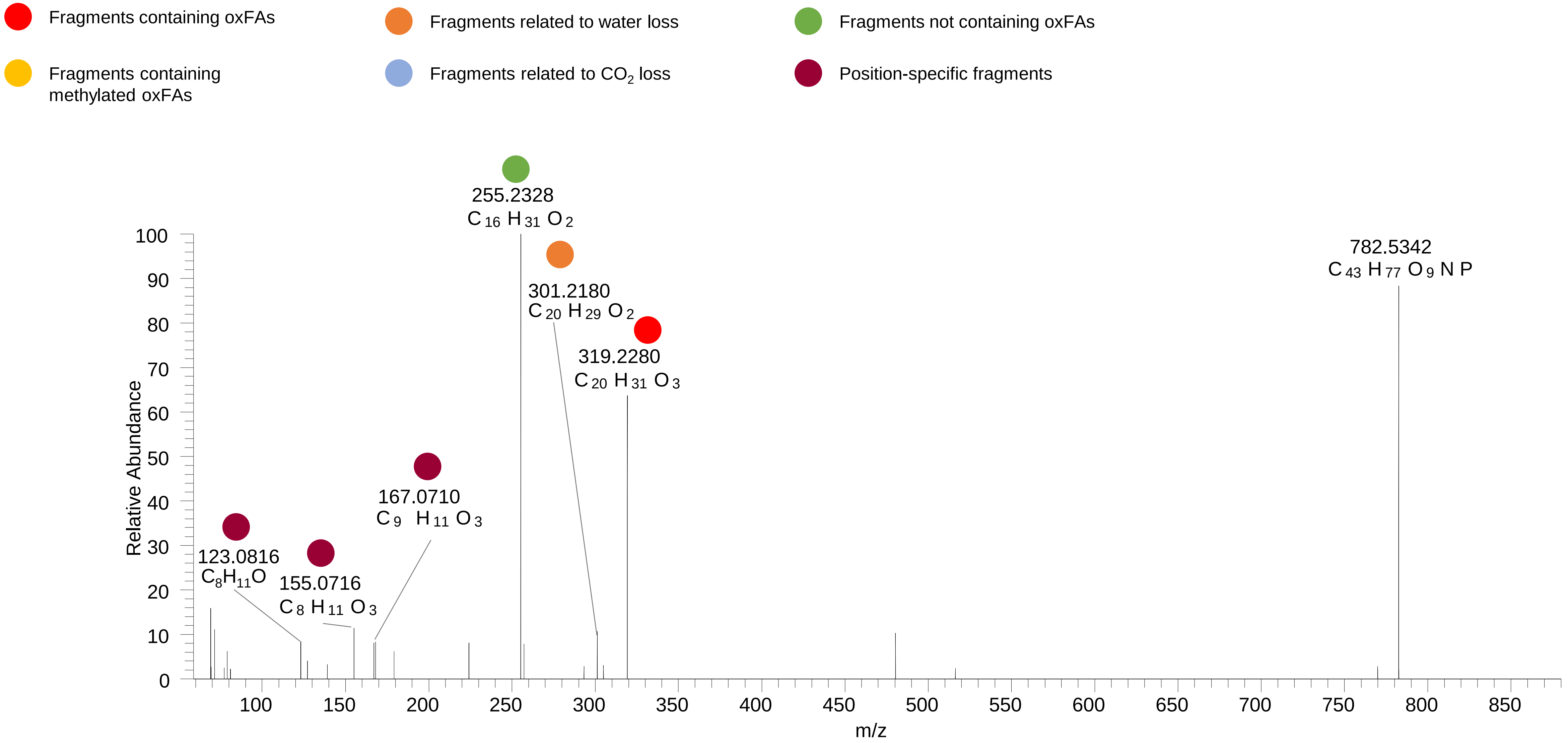

PC(16:0_20:4<OH{5}>)
RT 15.1

[oxPC+HCOO]- XIC 842.5553 NL: 6.83E3

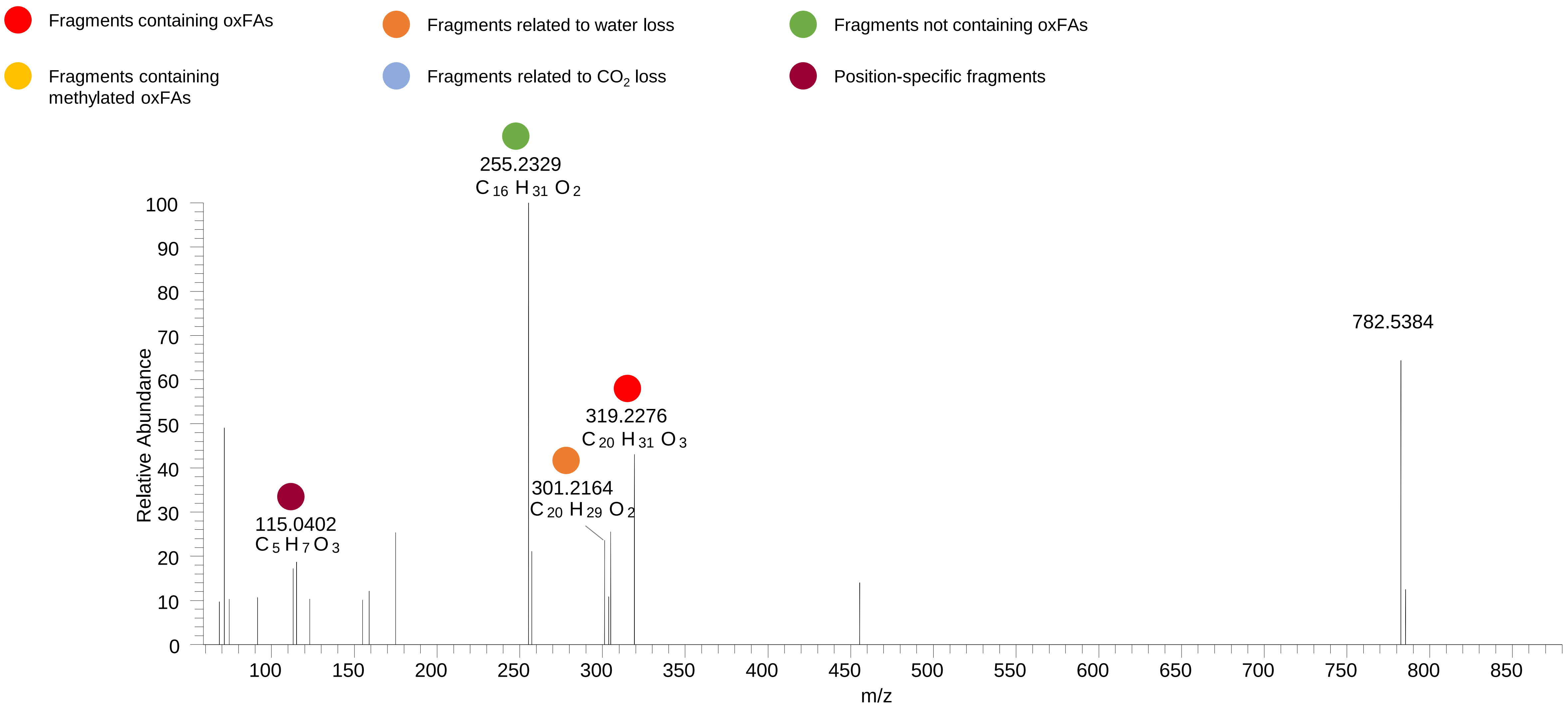

PC(18:1_18:2<O>)
RT 12.8

[oxPC+HCOO]- XIC 844.5709 NL: 5.31E3

- Fragments containing oxFAs
- Fragments related to water loss
- Fragments not containing oxFAs
- Fragments containing methylated oxFAs
- Fragments related to CO_2 loss
- Position-specific fragments

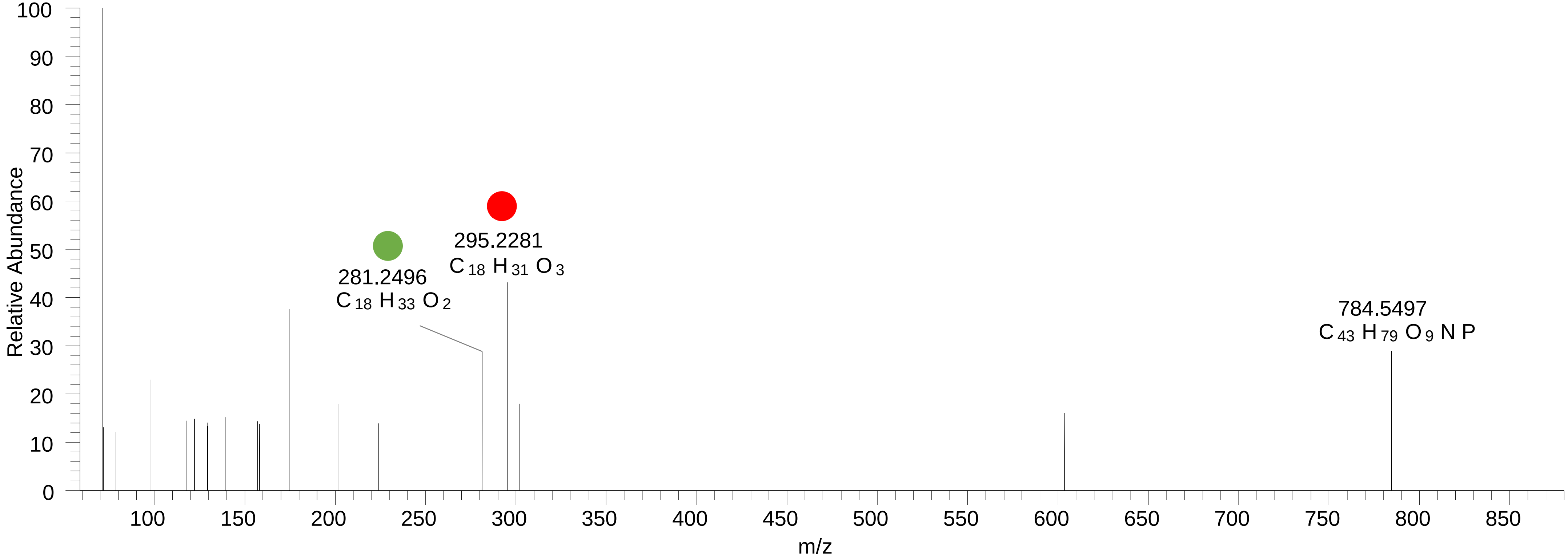

PC(16:0_20:3<OH{12}>)
RT 14.3

[oxPC+HCOO]- XIC 844.5709 NL: 2.08E4

- Fragments containing oxFAs
- Fragments related to water loss
- Fragments not containing oxFAs
- Fragments containing methylated oxFAs
- Fragments related to CO_2 loss
- Position-specific fragments

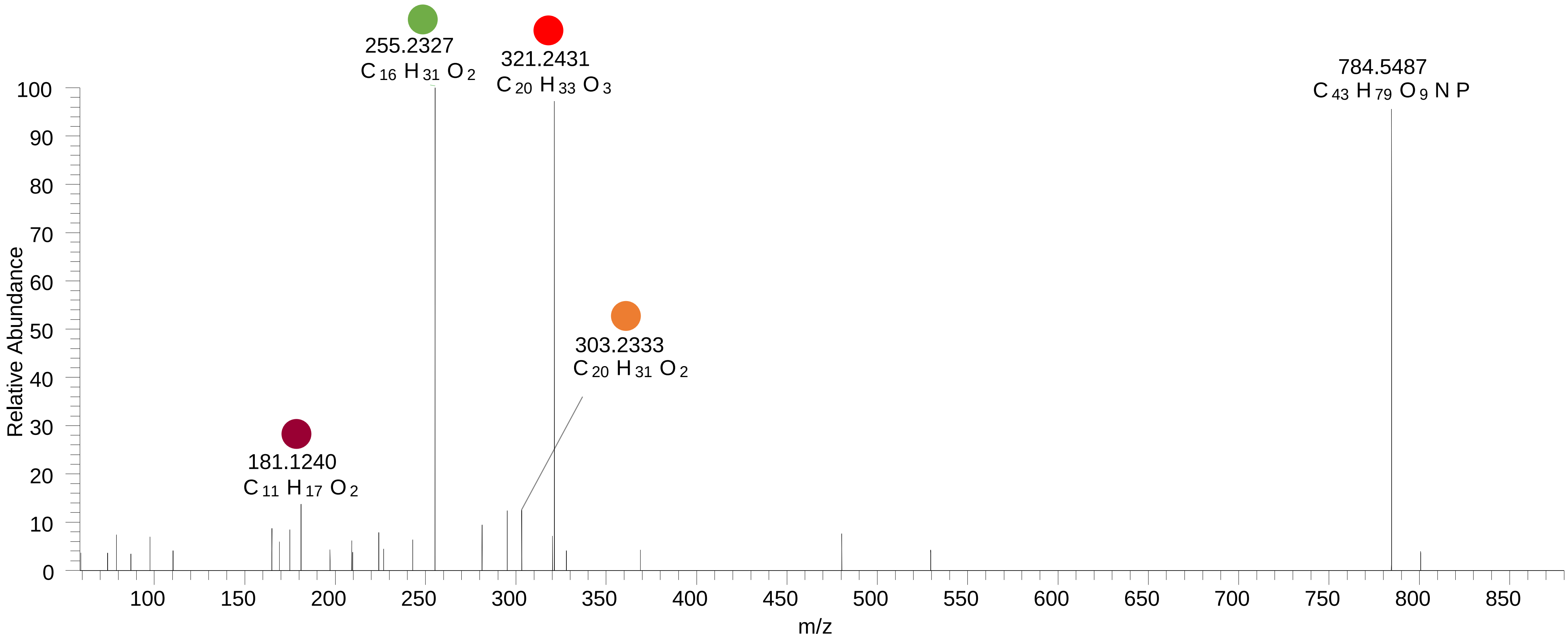

PC(18:0_18:2<O>)
PC(16:0_20:2<OH>)
RT 14.6

[oxPC+HCOO]-   XIC 846.5866 NL: 5.96E3

- Fragments containing oxFAs
- Fragments related to water loss
- Fragments not containing oxFAs
- Fragments containing methylated oxFAs
- Fragments related to CO_2 loss
- Position-specific fragments

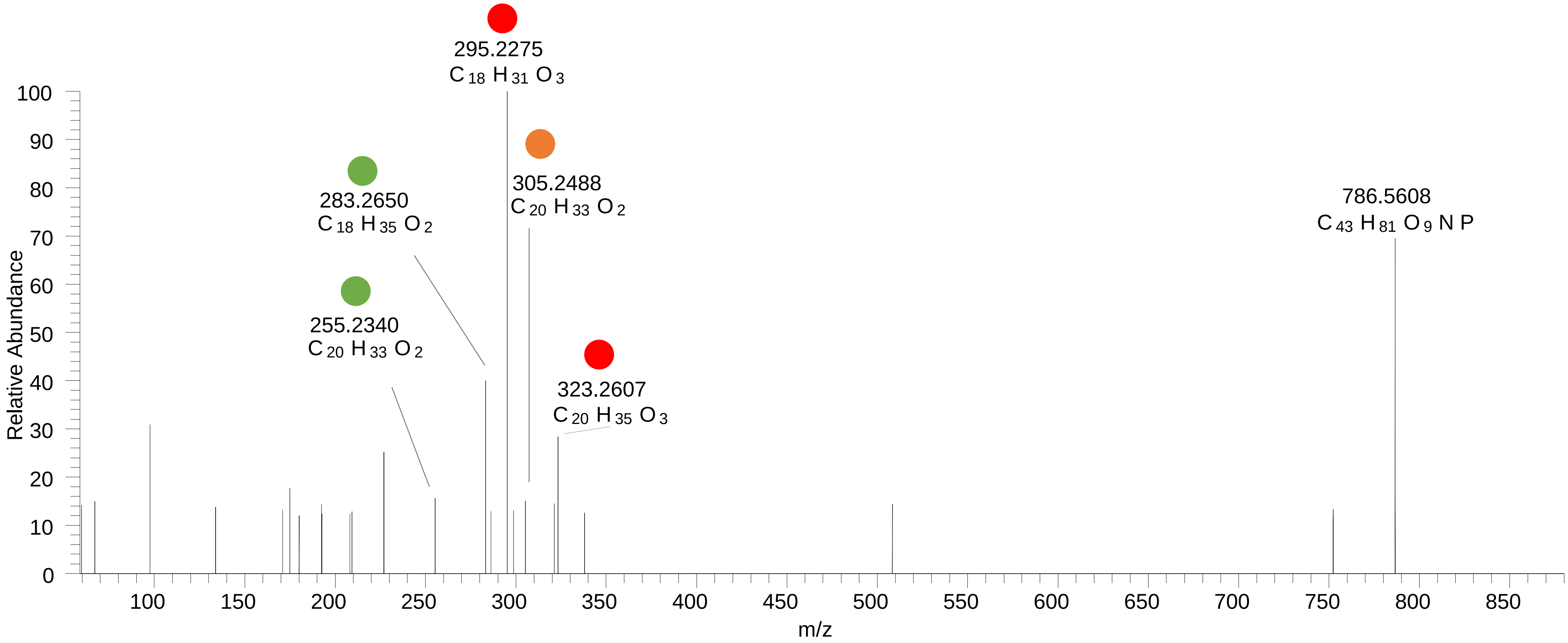

PC(18:0_18:2<O>)
RT 14.8

[oxPC+HCOO]- XIC 846.5866 NL: 2.75E3

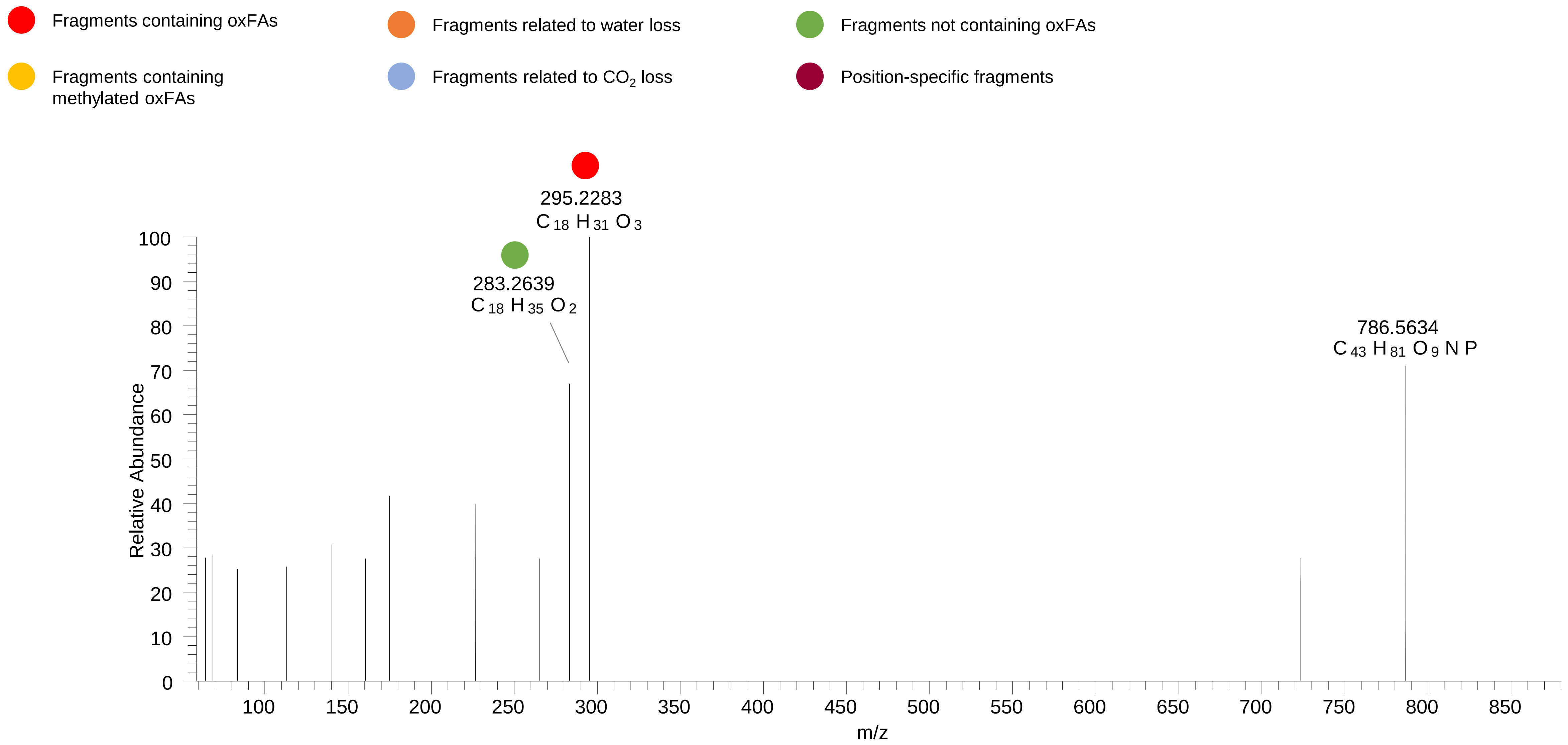

PC(18:0_18:2<OH{13}>)

RT 15.0

[oxPC+HCOO]- XIC 846.5866 NL: 4.09E4

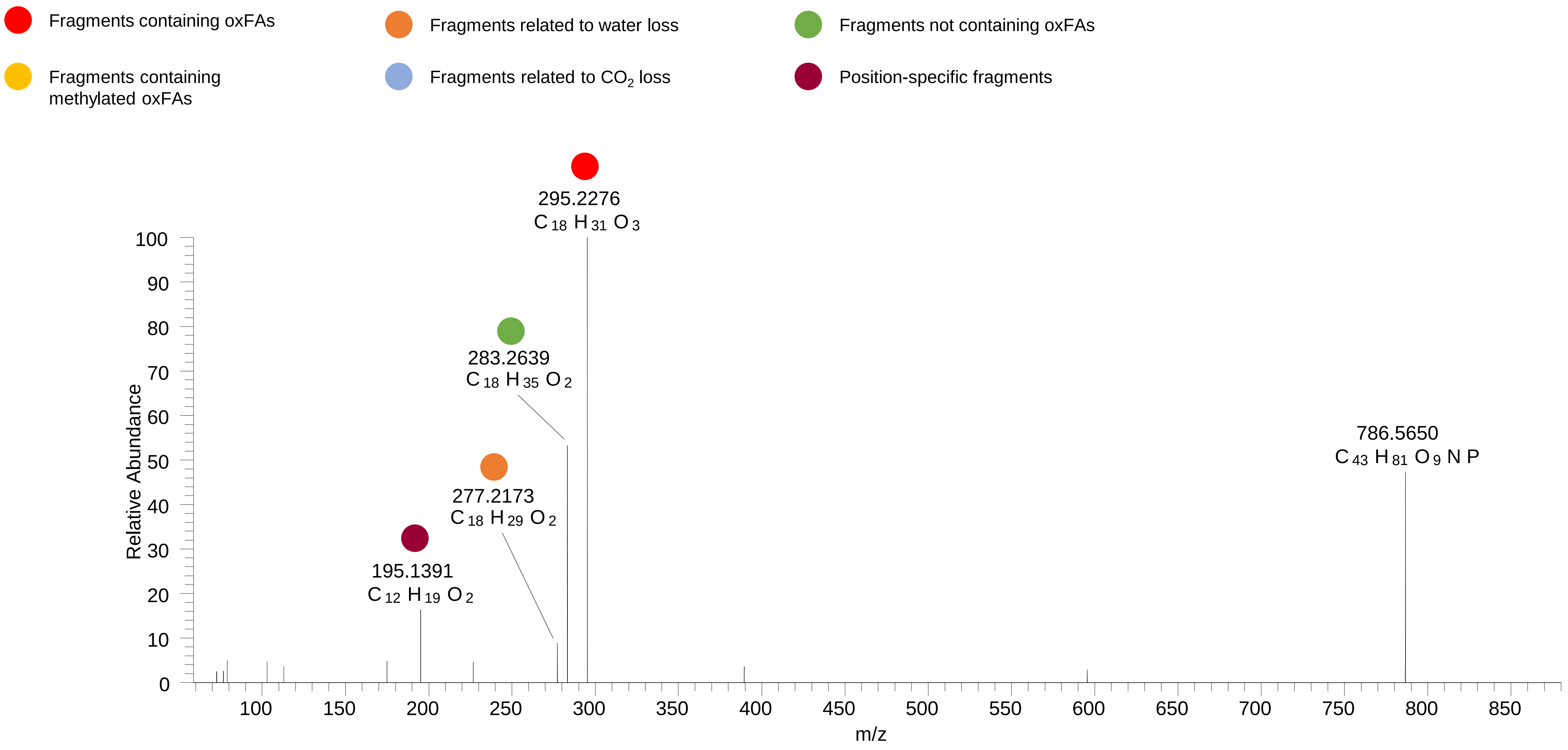

PC(18:0_18:2<OH{9}>)
RT 15.3

[oxPC+HCOO]-   XIC 846.5866 NL: 3.95E4

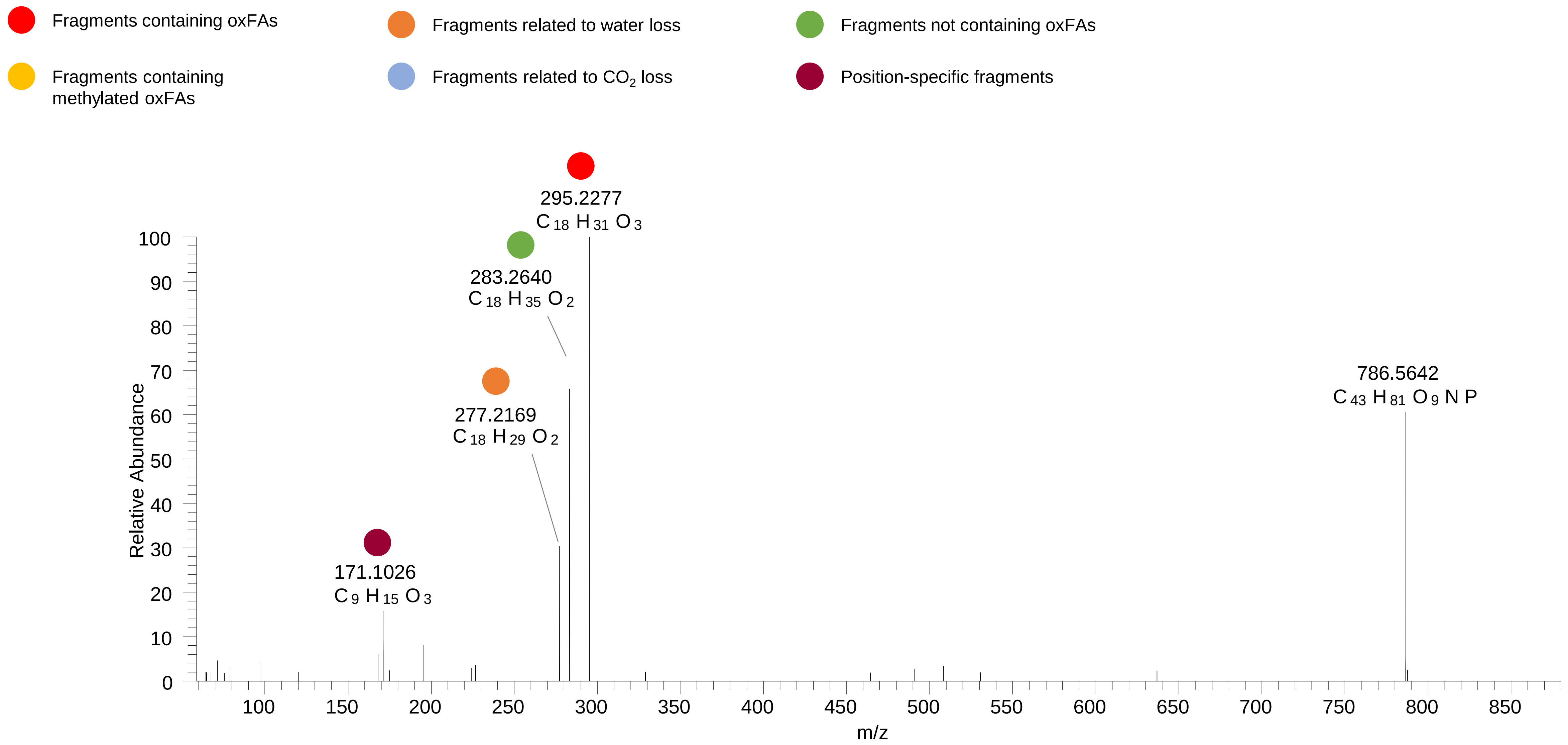

PC(16:0_18:2<OOH,OH>)
RT 11.7

[oxPC+HCOO]- XIC 850.5451 NL: 8.02E3

- Fragments containing oxFAs
- Fragments related to water loss
- Fragments not containing oxFAs
- Fragments containing methylated oxFAs
- Fragments related to CO_2 loss
- Position-specific fragments

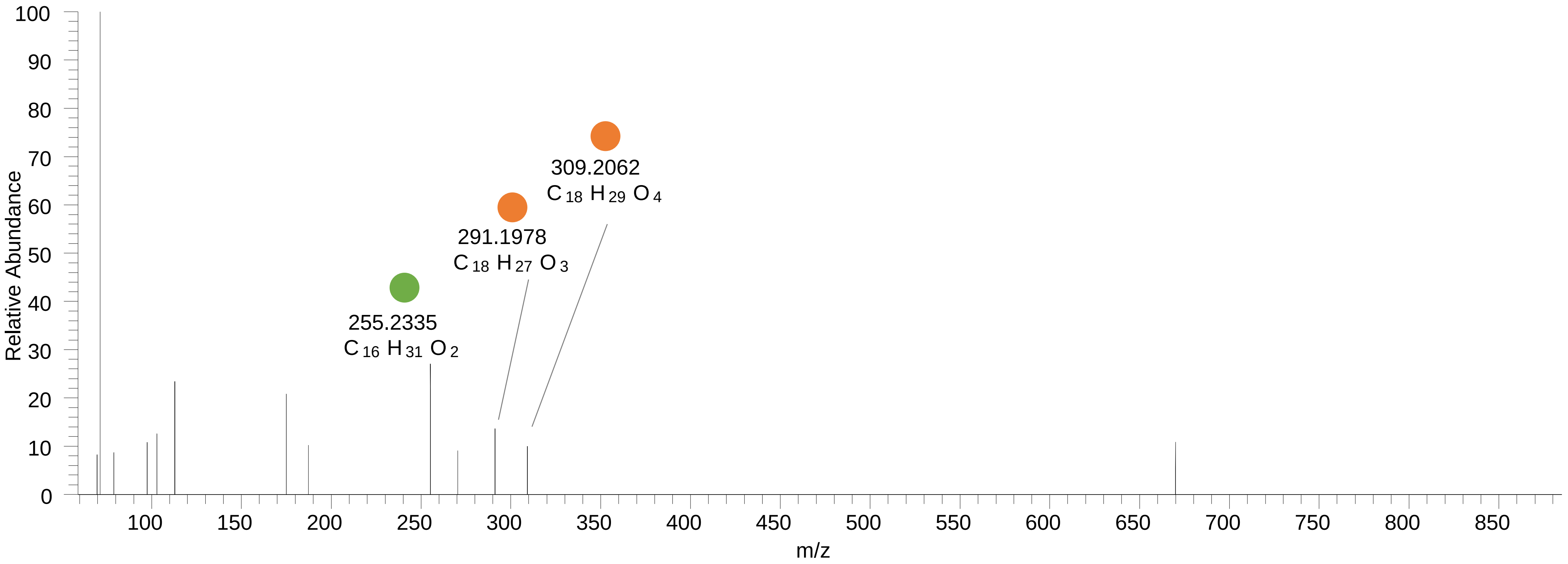

PC(16:0_20:4<OH,O>)
RT 11.1

[oxPC+HCOO]- XIC 858.5502 NL: 6.06E3

- Fragments containing oxFAs
- Fragments related to water loss
- Fragments not containing oxFAs
- Fragments containing methylated oxFAs
- Fragments related to CO_2 loss
- Position-specific fragments

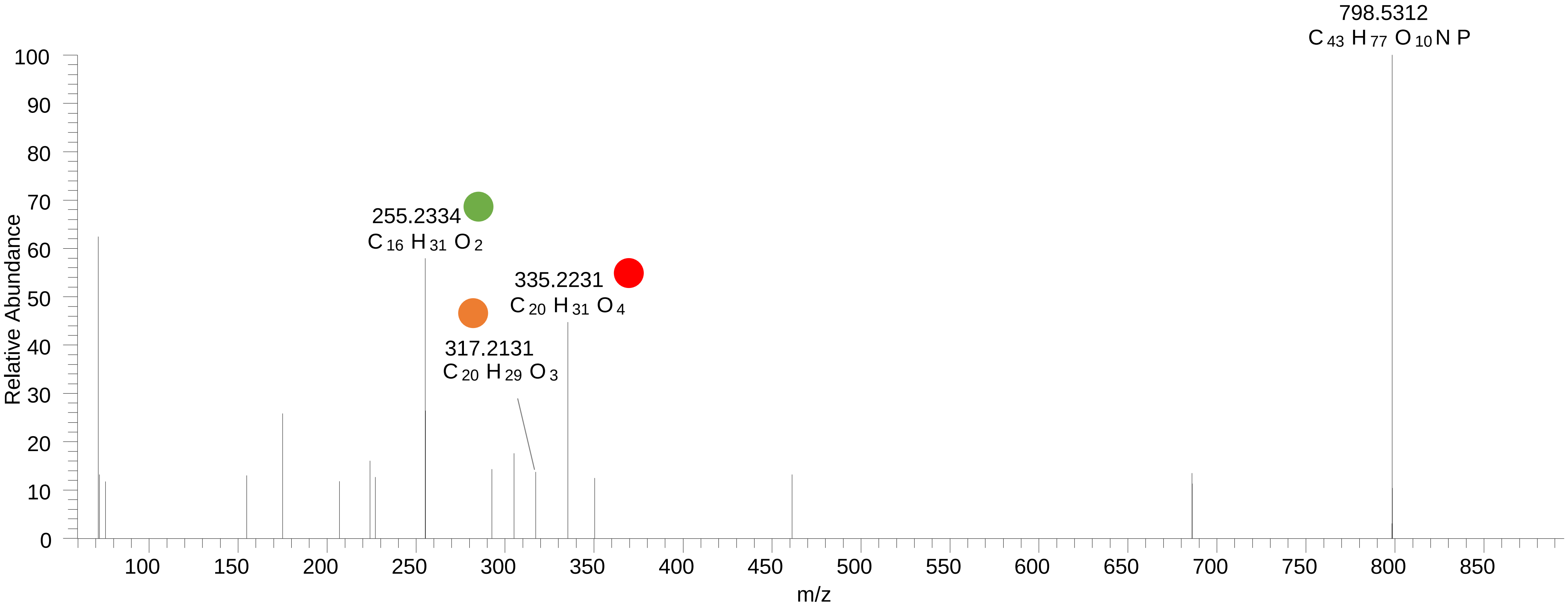

PC(16:0_20:4<2O>)
RT 12.0

[oxPC+HCOO]-   XIC 858.5502 NL: 3.17E3

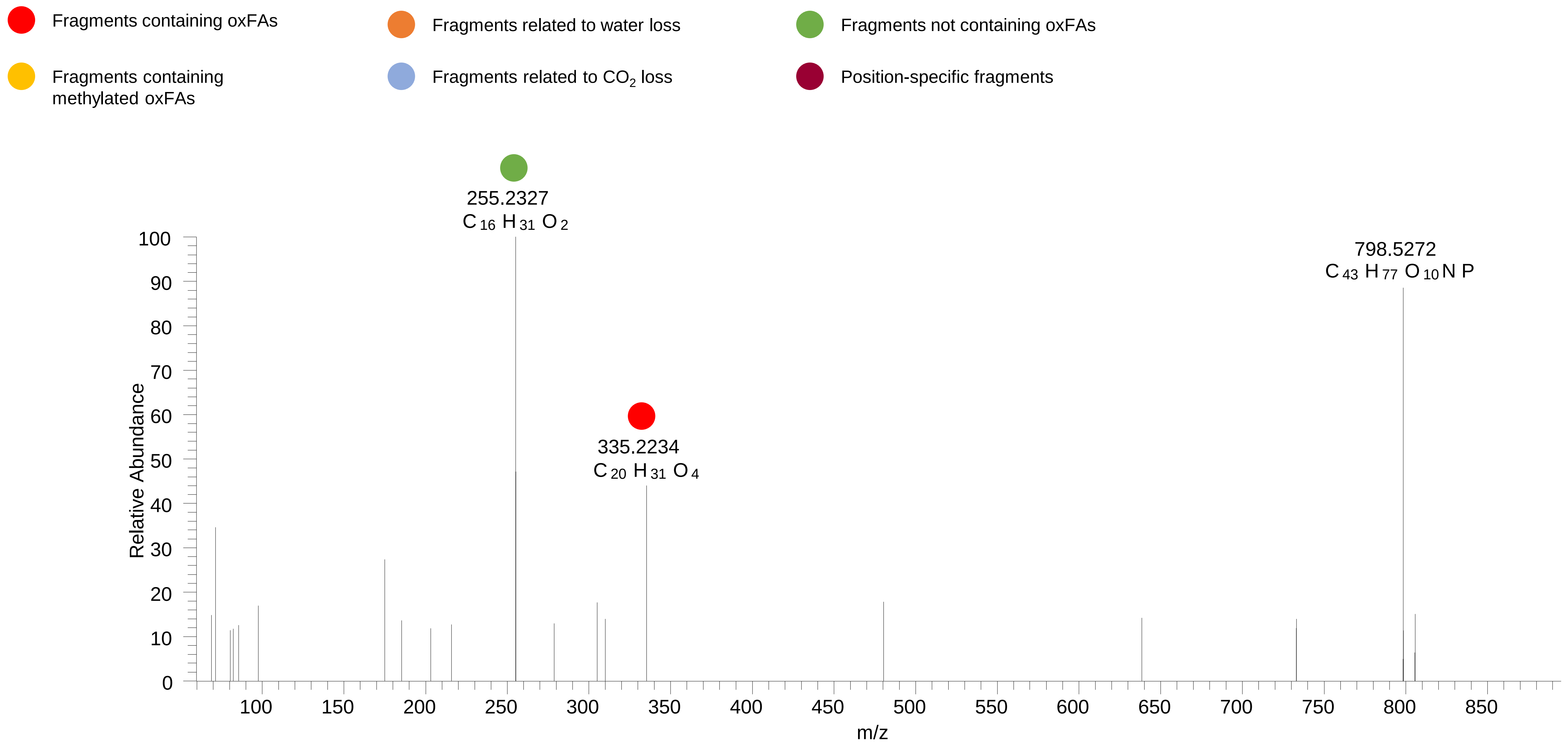

PC(16:0_20:4<OOH>)
RT 13.3

[oxPC+HCOO]- XIC 858.5502 NL: 2.41E3

- Fragments containing oxFAs
- Fragments related to water loss
- Fragments not containing oxFAs
- Fragments containing methylated oxFAs
- Fragments related to CO_2 loss
- Position-specific fragments

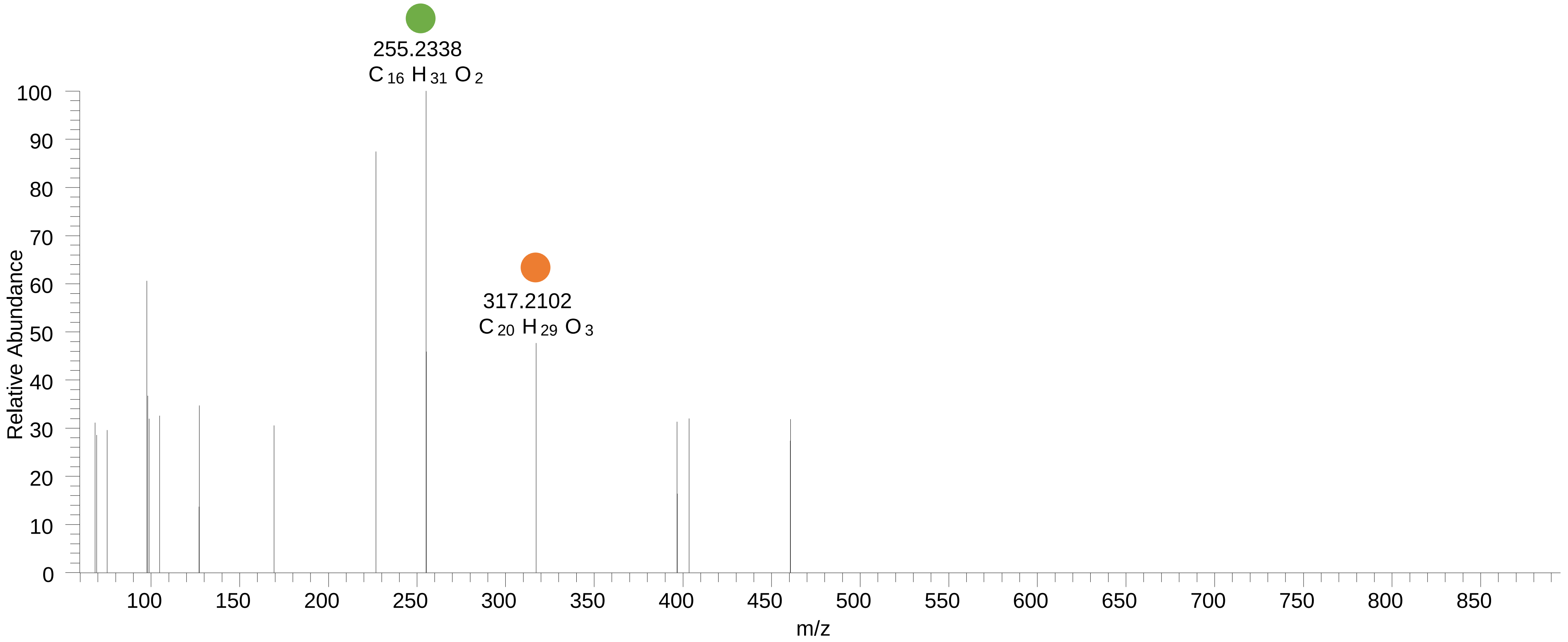

PC(16:0_20:4<OOH{15}>)
RT 13.5

[oxPC+HCOO]-   XIC 858.5502 NL: 5.87E3

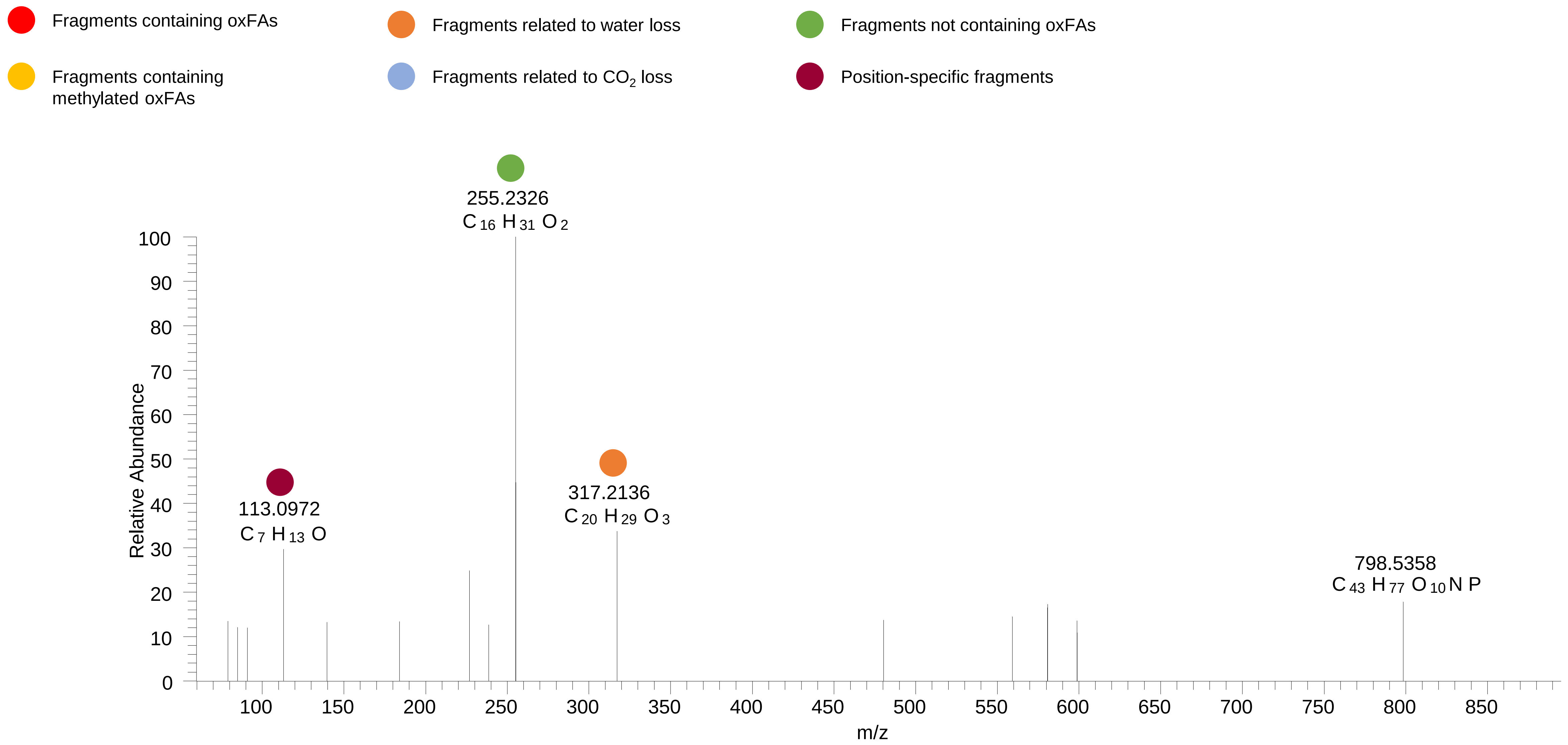

PC(16:0_20:4<OOH{12}>)
RT 13.9

[oxPC+HCOO]- XIC 858.5502 NL: 6.70E3

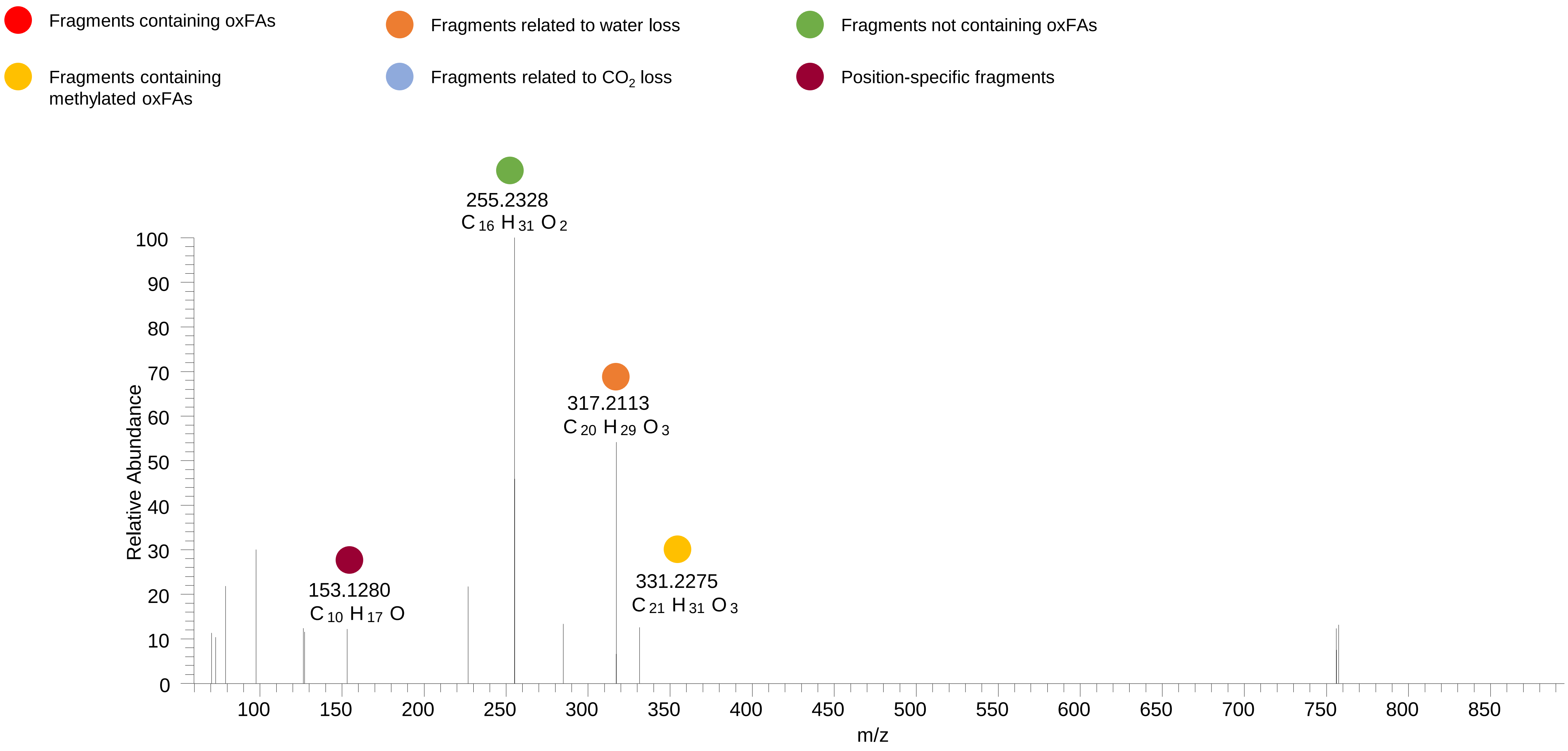

PC(16:0_20:4<OOH{7}>)
RT 14.4

[oxPC+HCOO]- XIC 858.5502 NL: 7.78E3

- Fragments containing oxFAs
- Fragments related to water loss
- Fragments not containing oxFAs
- Fragments containing methylated oxFAs
- Fragments related to CO_2 loss
- Position-specific fragments

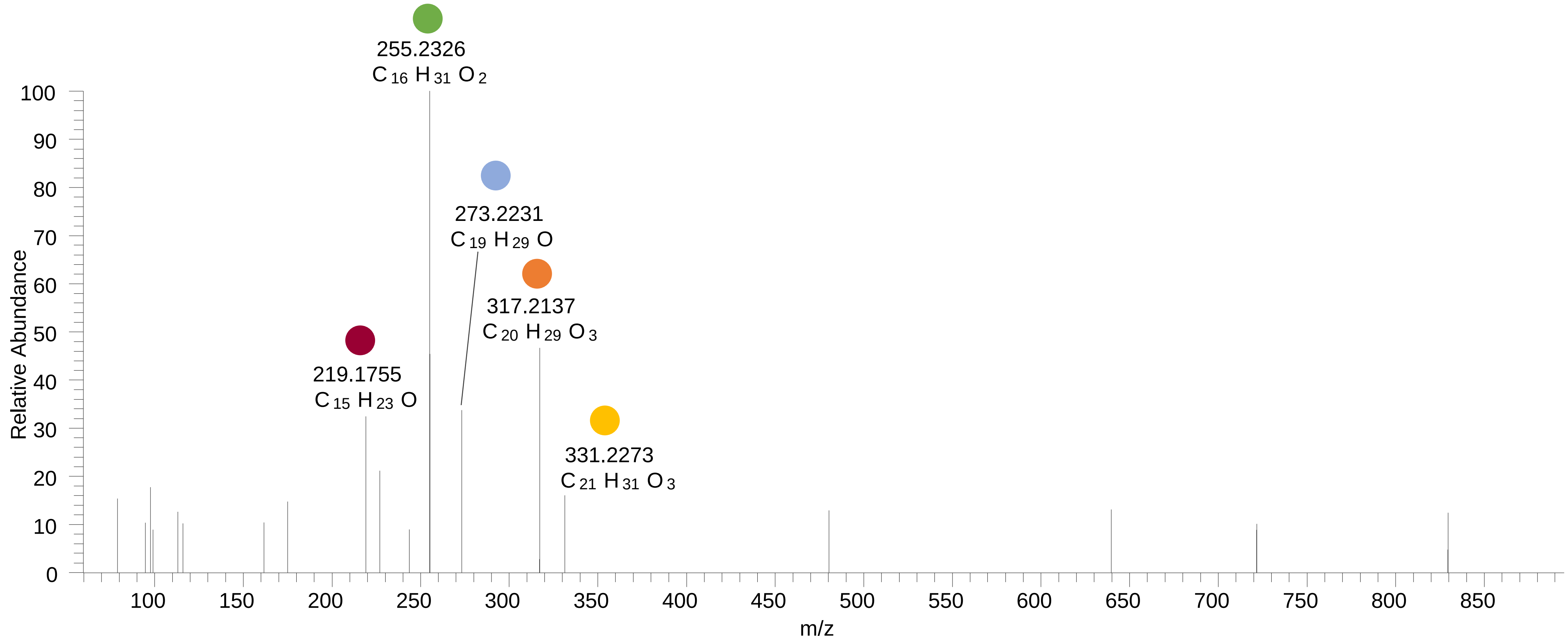

PC(18:1_18:2<OOH>)
PC(16:0_20:3<OOH>)
RT 13.8

[oxPC+HCOO]- XIC 860.5658 NL: 4.77E3

Fragments containing oxFAs

Fragments related to water loss

Fragments not containing oxFAs

Fragments containing methylated oxFAs

Fragments related to CO_2 loss

Position-specific fragments

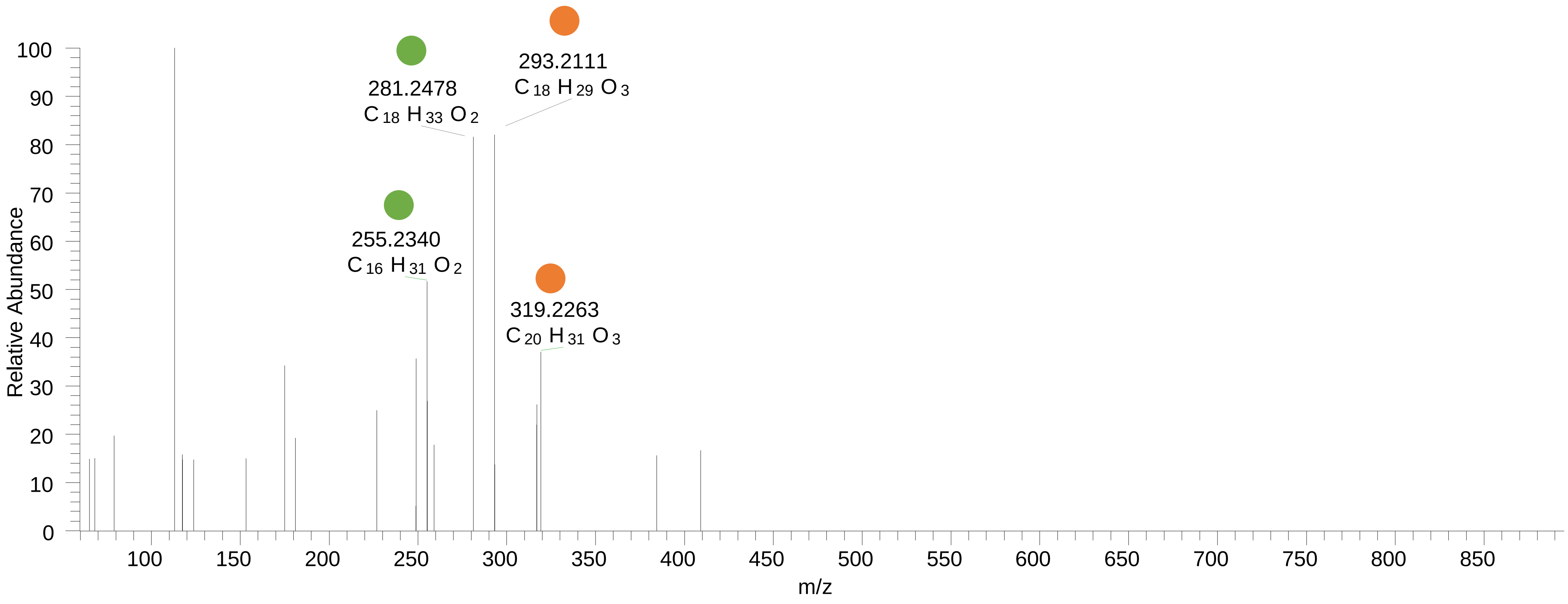

PC(16:0_20:3<OOH>)
RT 14.2

[oxPC+HCOO]- XIC 860.5658 NL: 5.68E3

- Fragments containing oxFAs
- Fragments related to water loss
- Fragments not containing oxFAs
- Fragments containing methylated oxFAs
- Fragments related to CO_2 loss
- Position-specific fragments

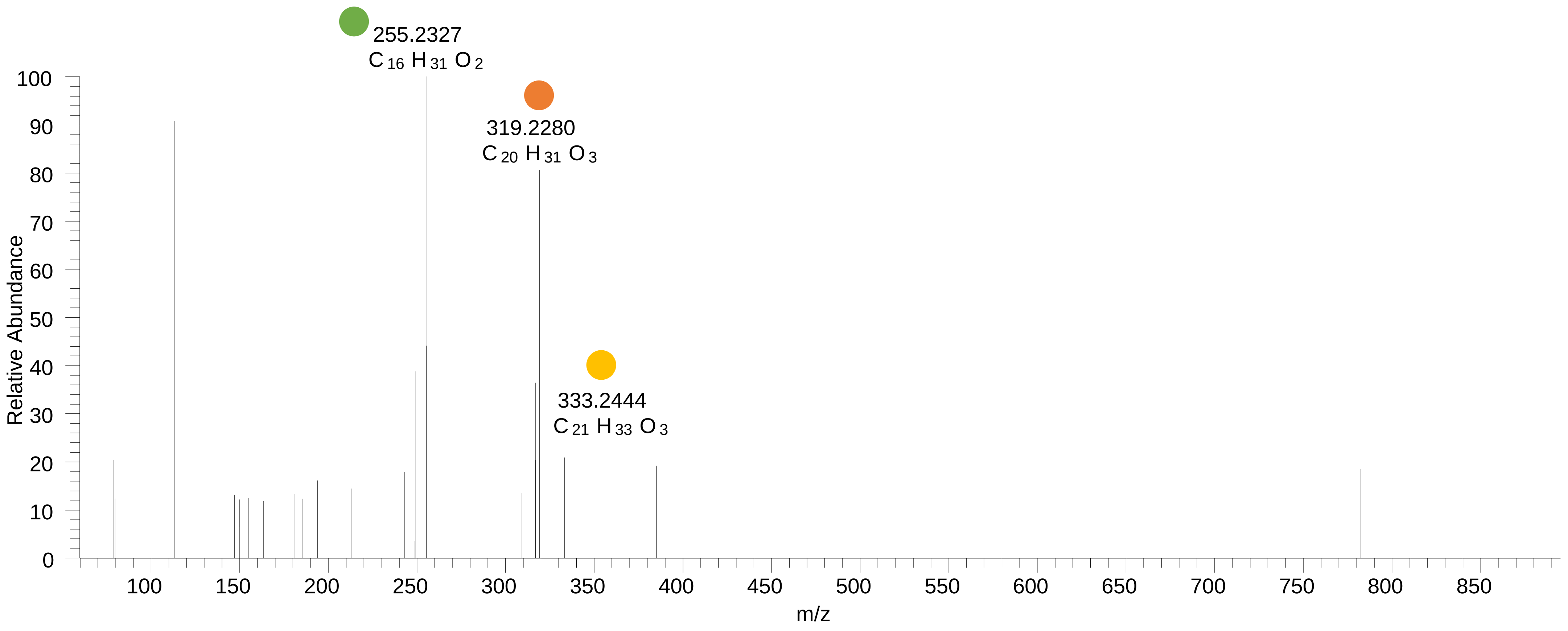

PC(18:0_18:2<OOH>)
RT 14.6

[oxPC+HCOO]- XIC 862.5815 NL: 3.51E3

- Fragments containing oxFAs
- Fragments related to water loss
- Fragments not containing oxFAs
- Fragments containing methylated oxFAs
- Fragments related to CO_2 loss
- Position-specific fragments

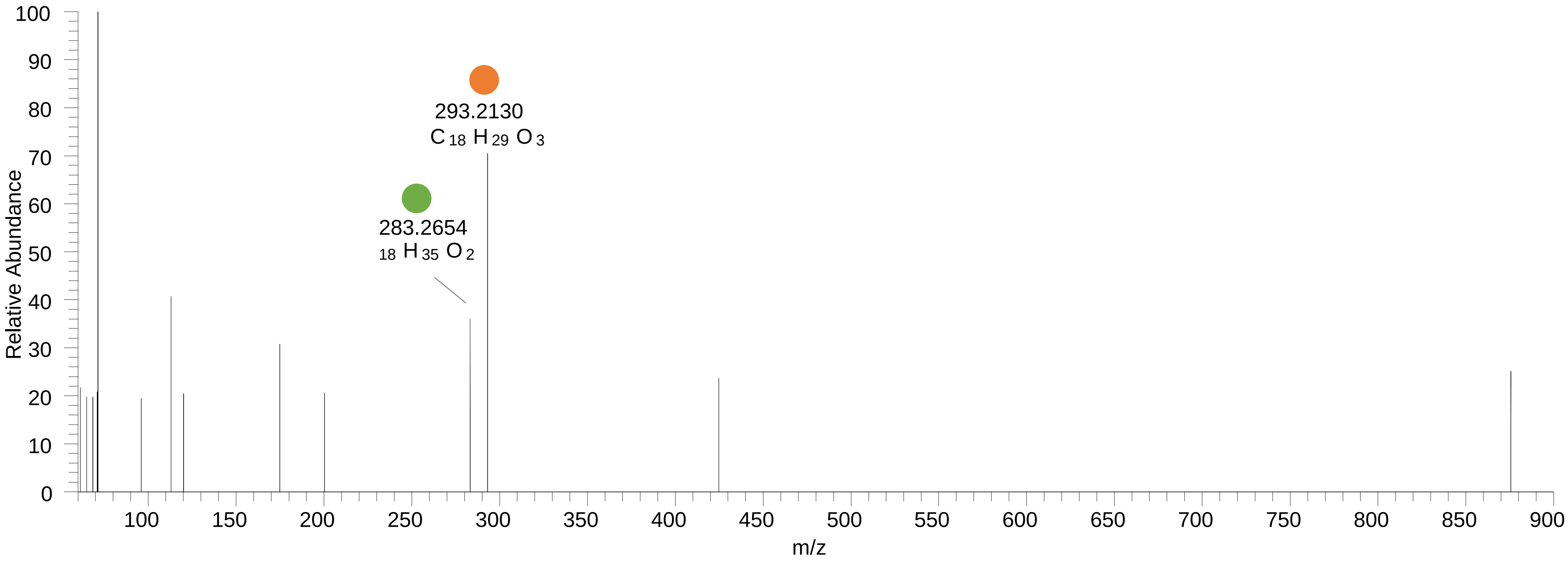

PC(18:0_18:2<OOH{13}>)
RT 15.1

[oxPC+HCOO]- XIC 862.5815 NL: 7.63E2

- Fragments containing oxFAs
- Fragments related to water loss
- Fragments not containing oxFAs
- Fragments containing methylated oxFAs
- Fragments related to CO_2 loss
- Position-specific fragments

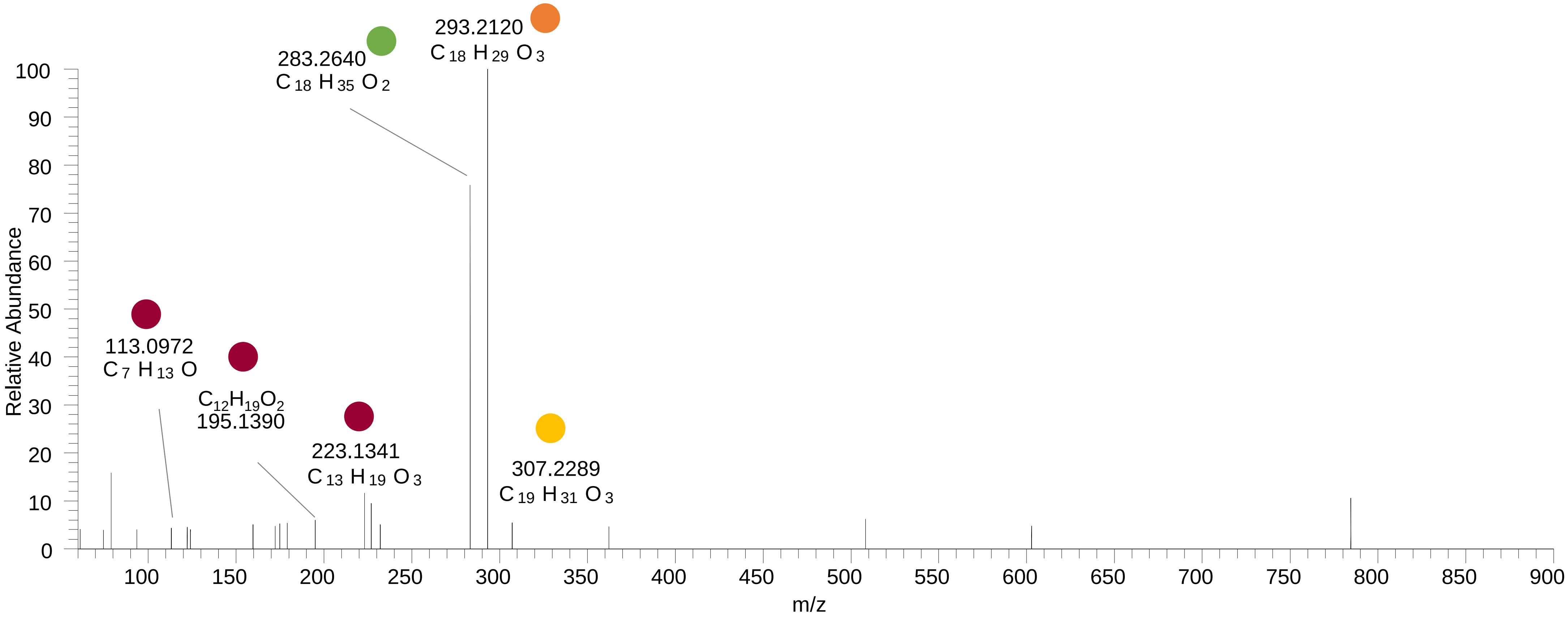

PC(16:0_22:6<O{16}>)
RT 13.5

[oxPC+HCOO]-   XIC 866.5553 NL: 1.08E4

- Fragments containing oxFAs
- Fragments related to water loss
- Fragments not containing oxFAs
- Fragments containing methylated oxFAs
- Fragments related to CO_2 loss
- Position-specific fragments

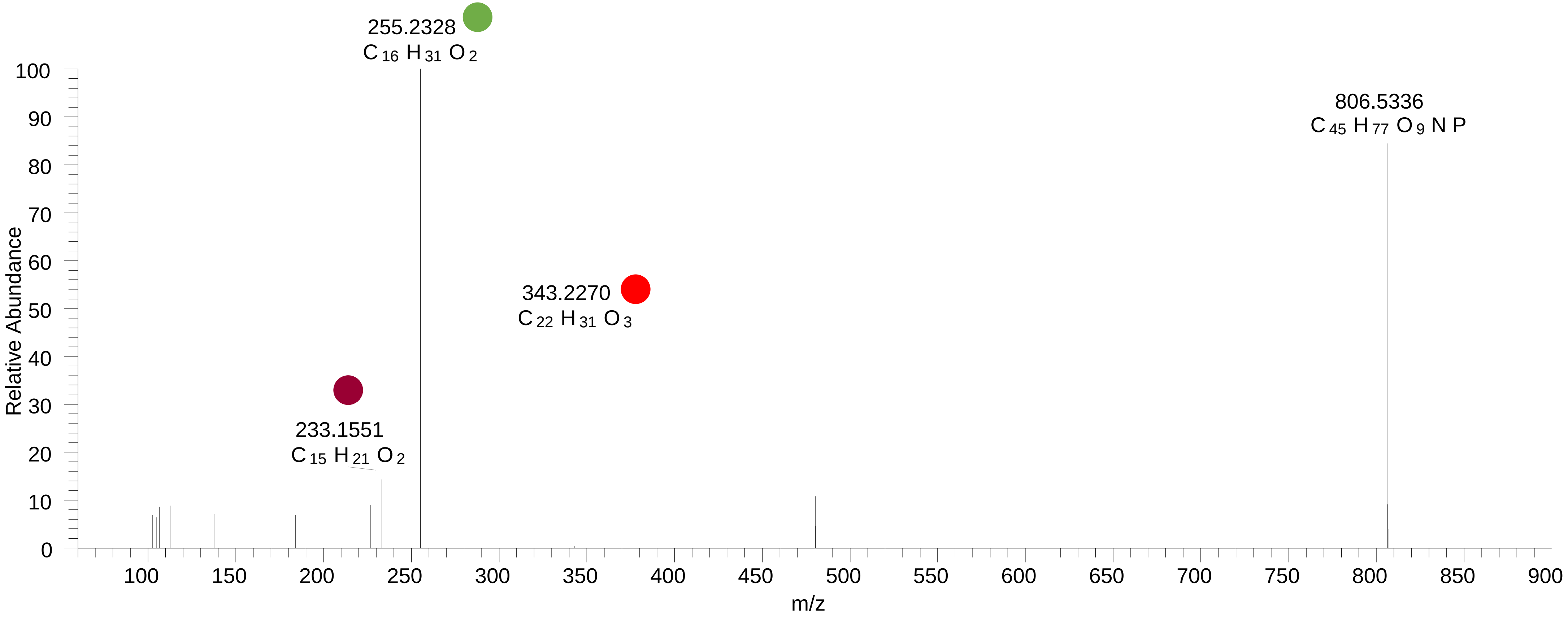

PC(16:0_22:5<ep{7-8}>)
RT 14.5

[oxPC+HCOO]- XIC 866.5553 NL: 8.08E3

- Fragments containing oxFAs
- Fragments related to water loss
- Fragments not containing oxFAs
- Fragments containing methylated oxFAs
- Fragments related to CO_2 loss
- Position-specific fragments

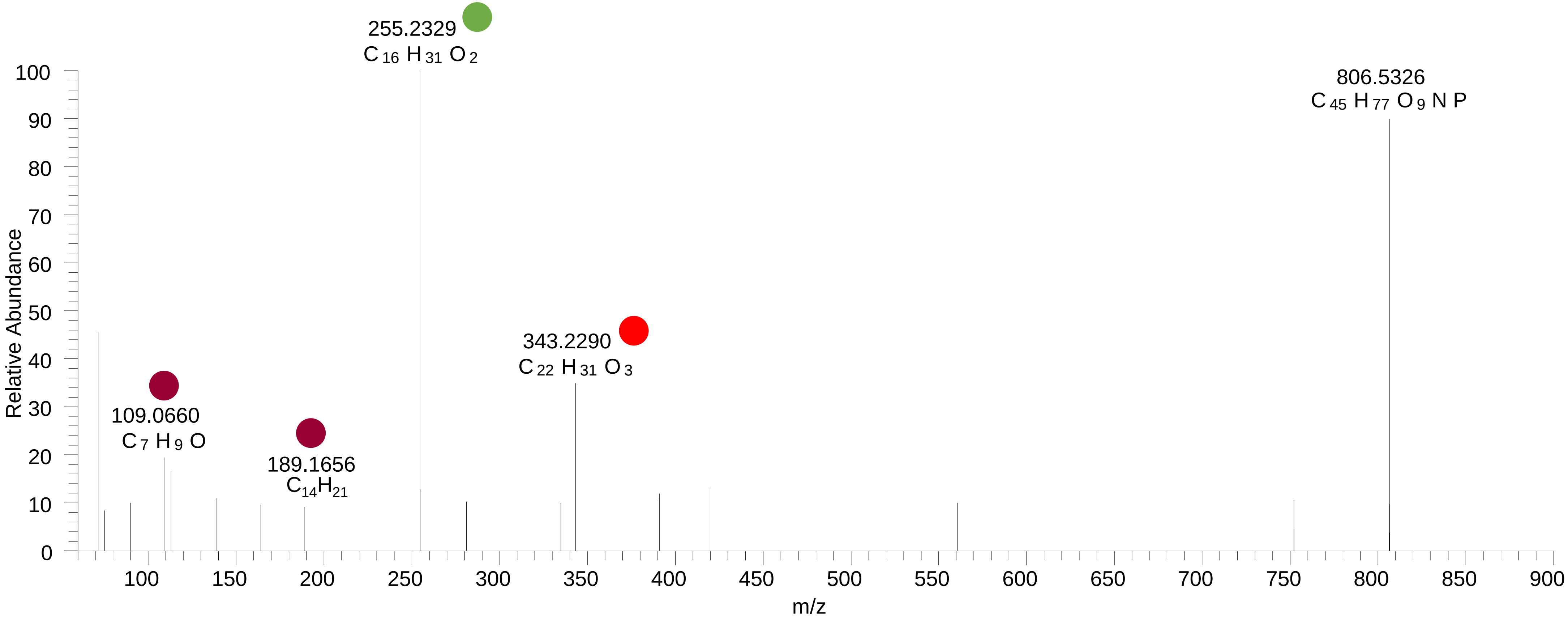

PC(16:0_22:5<OH>)
RT 13.7

[oxPC+HCOO]- XIC 868.5709 NL: 3.96E3

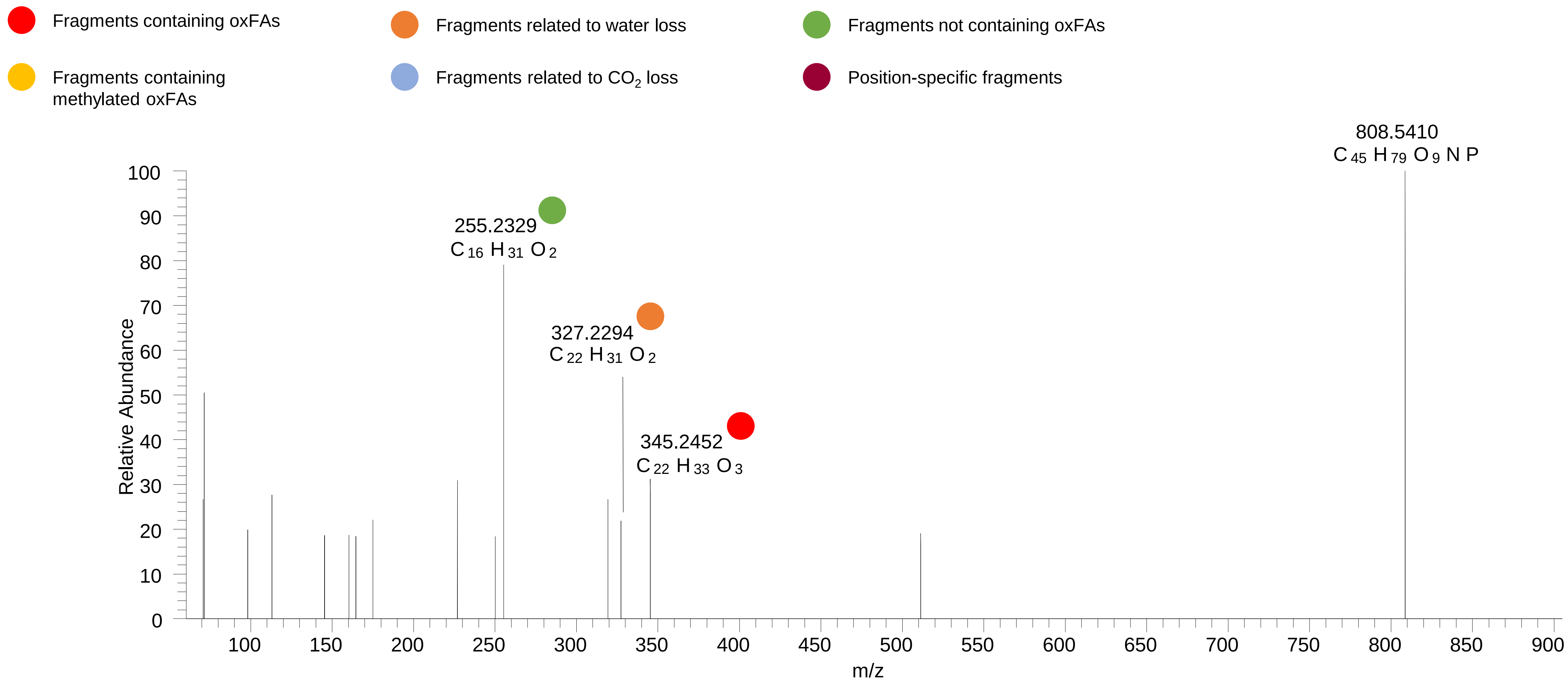

PC(16:0_22:5<OH>)
PC(18:1_20:4<O>)
RT 13.8-14.5

[oxPC+HCOO]- XIC 868.5709 NL: 8.32E3

- Fragments containing oxFAs
- Fragments related to water loss
- Fragments not containing oxFAs
- Fragments containing methylated oxFAs
- Fragments related to CO_2 loss
- Position-specific fragments

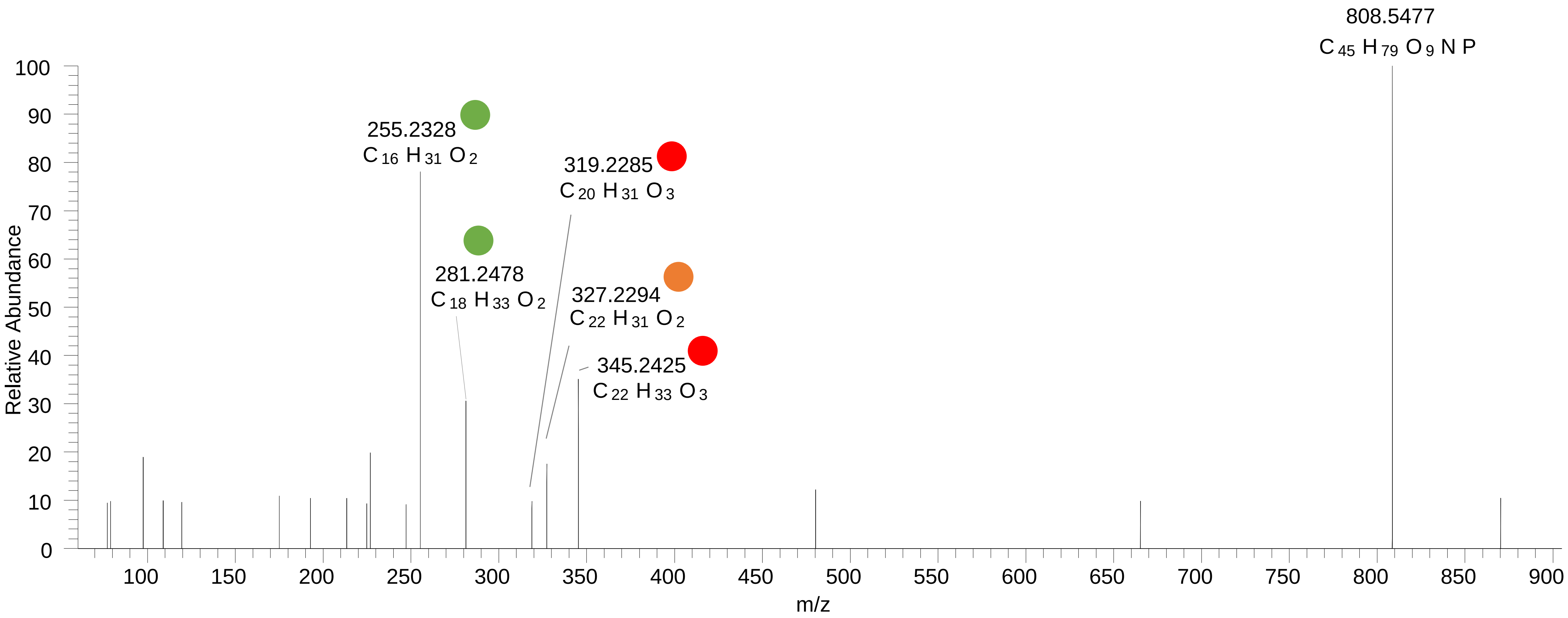

PC(18:0_20:5<O>)
PC(18:1_20:4<O>)
RT 14.7-15.0

[oxPC+HCOO]- XIC 868.5709 NL: 3.85E3

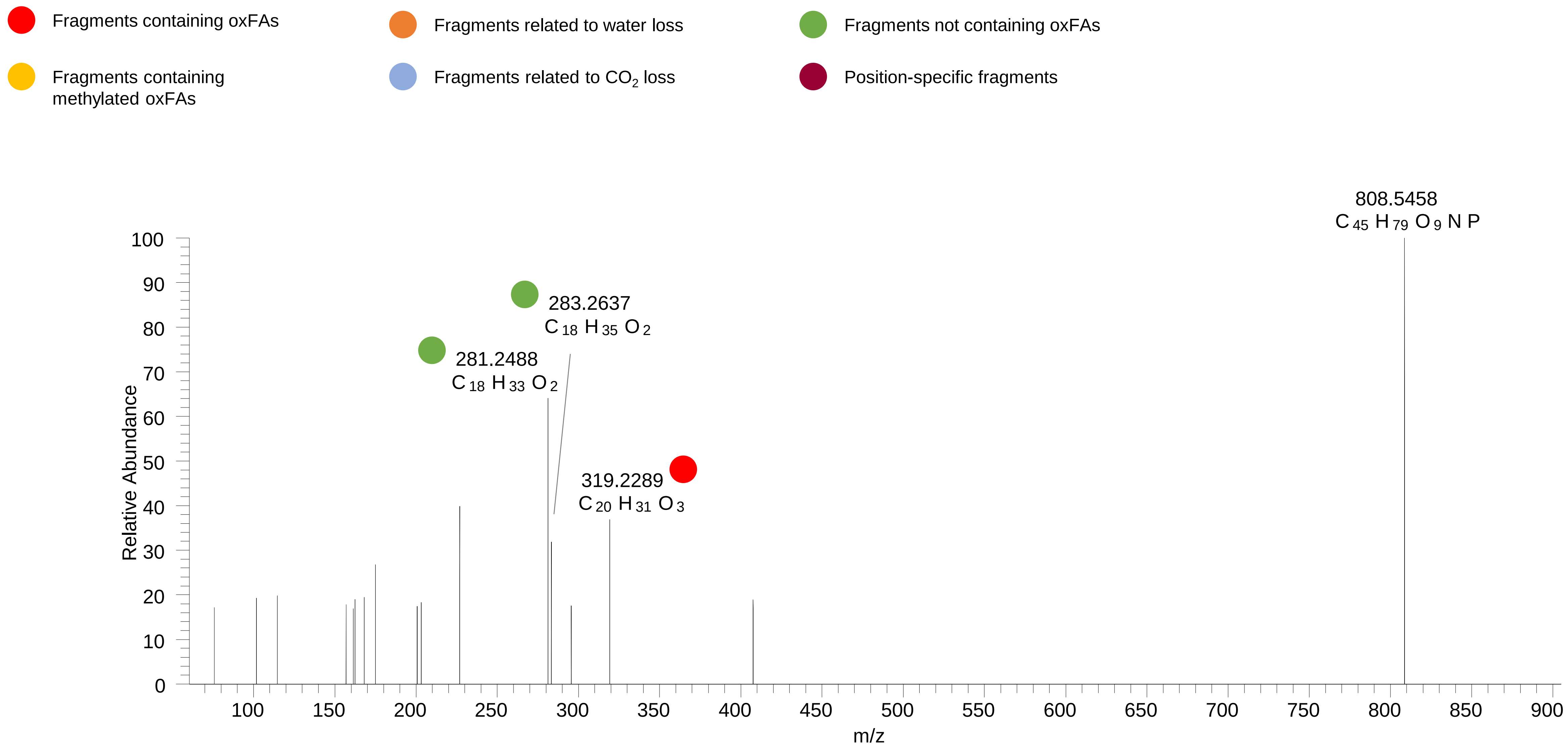

PC(18:1_20:3<O>)
RT 14.4

[oxPC+HCOO]- XIC 870.5866 NL: 5.48E3

- Fragments containing oxFAs
- Fragments related to water loss
- Fragments not containing oxFAs
- Fragments containing methylated oxFAs
- Fragments related to CO_2 loss
- Position-specific fragments

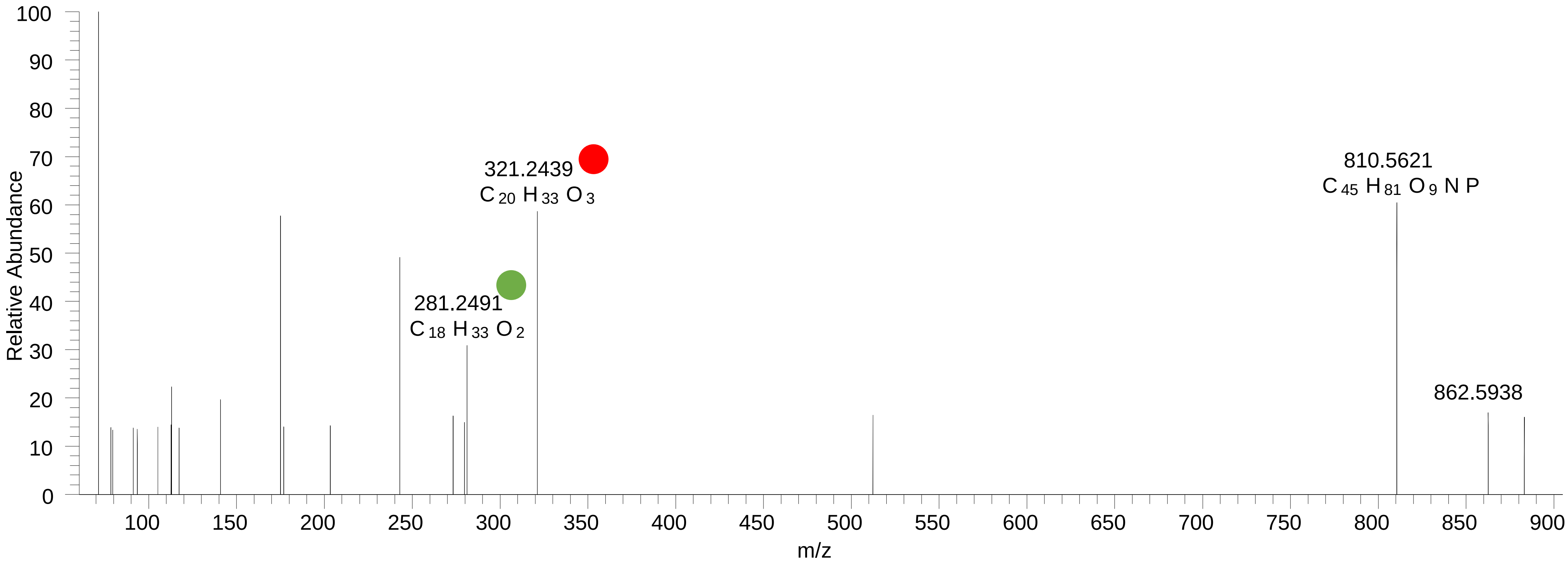

PC(18:0_20:4<OH>)
RT 15.3

[oxPC+HCOO]- XIC 870.5866 NL: 8.66E3

- Fragments containing oxFAs
- Fragments related to water loss
- Fragments not containing oxFAs
- Fragments containing methylated oxFAs
- Fragments related to CO_2 loss
- Position-specific fragments

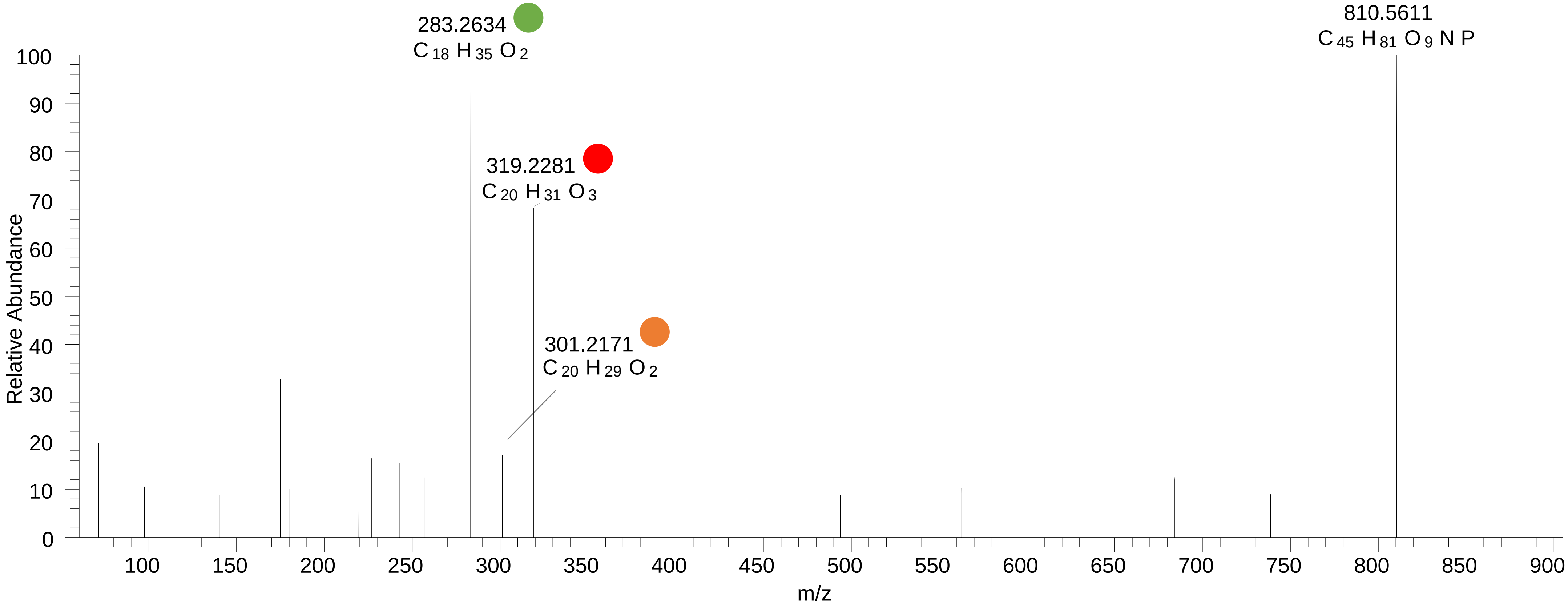

PC(18:0_20:4<OH{11}>)
RT 15.7

[oxPC+HCOO]- XIC 870.5866 NL: 1.24E4

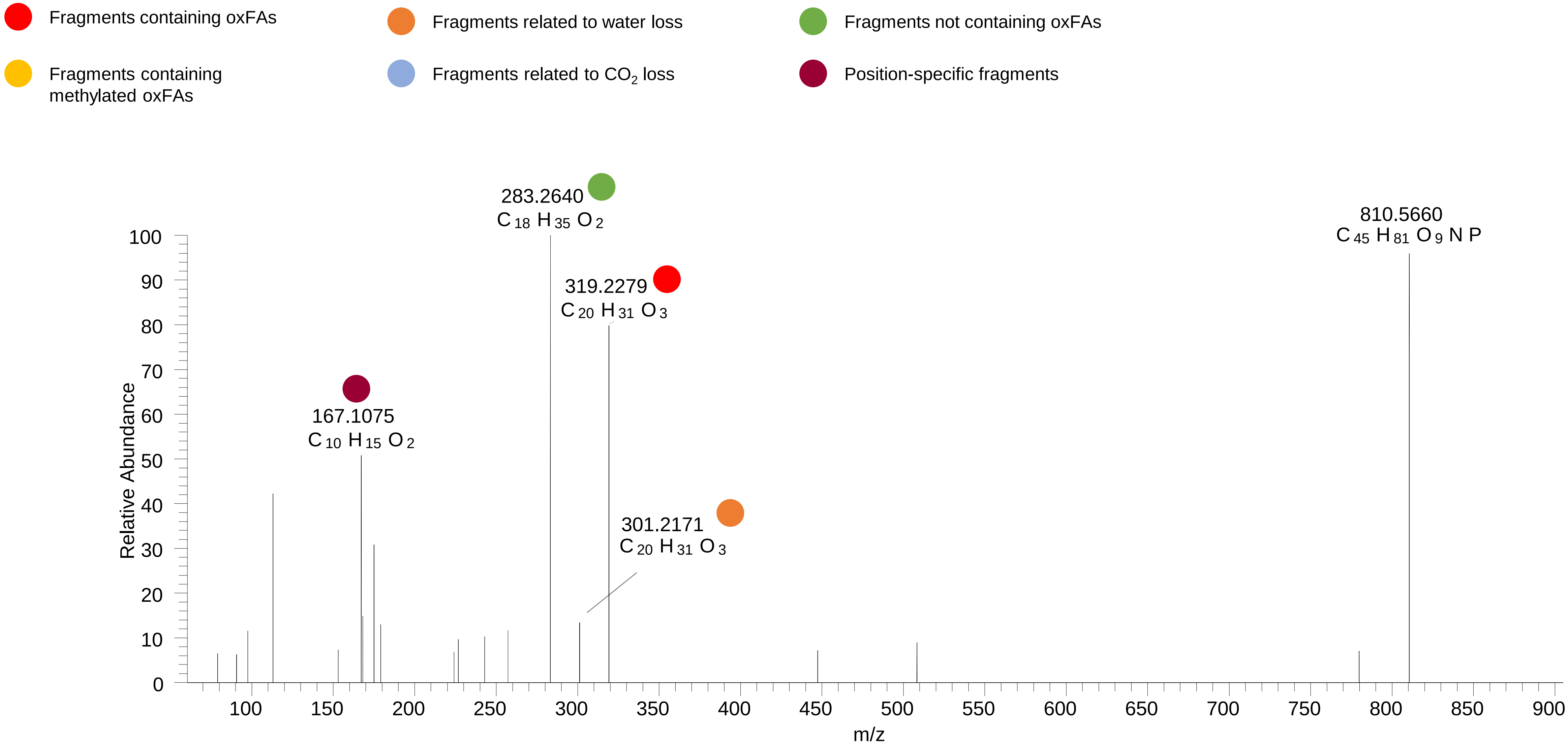

PC(18:0_20:4<OH{8}>)
PC(18:0_20:4<OH{9}>)
RT 16.2

[oxPC+HCOO]- XIC 870.5866 NL: 1.87E4

- Fragments containing oxFAs
- Fragments related to water loss
- Fragments not containing oxFAs
- Fragments containing methylated oxFAs
- Fragments related to CO_2 loss
- Position-specific fragments

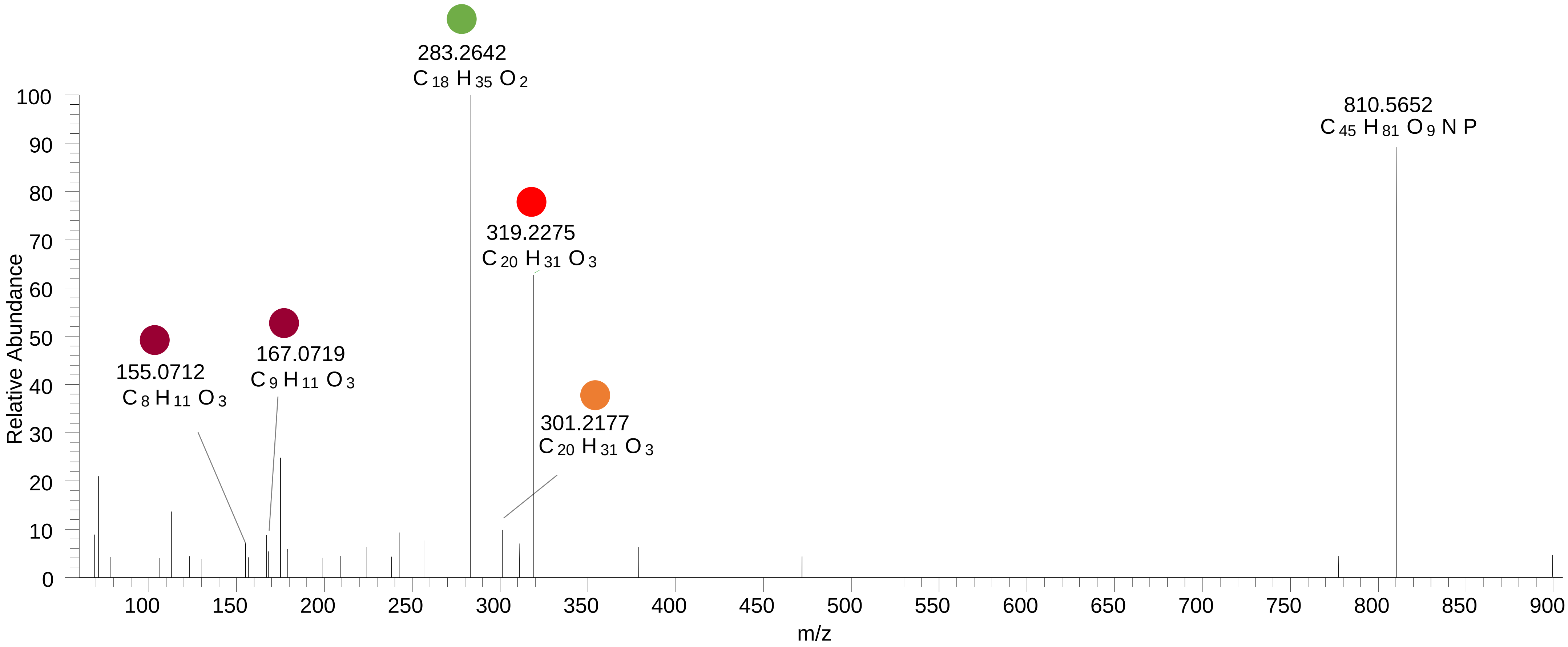

PC(16:0_22:6<2O>)
RT 10.8-14.4

[oxPC+HCOO]- XIC 882.5502 NL: 2.46E3

- Fragments containing oxFAs
- Fragments related to water loss
- Fragments not containing oxFAs
- Fragments containing methylated oxFAs
- Fragments related to CO_2 loss
- Position-specific fragments

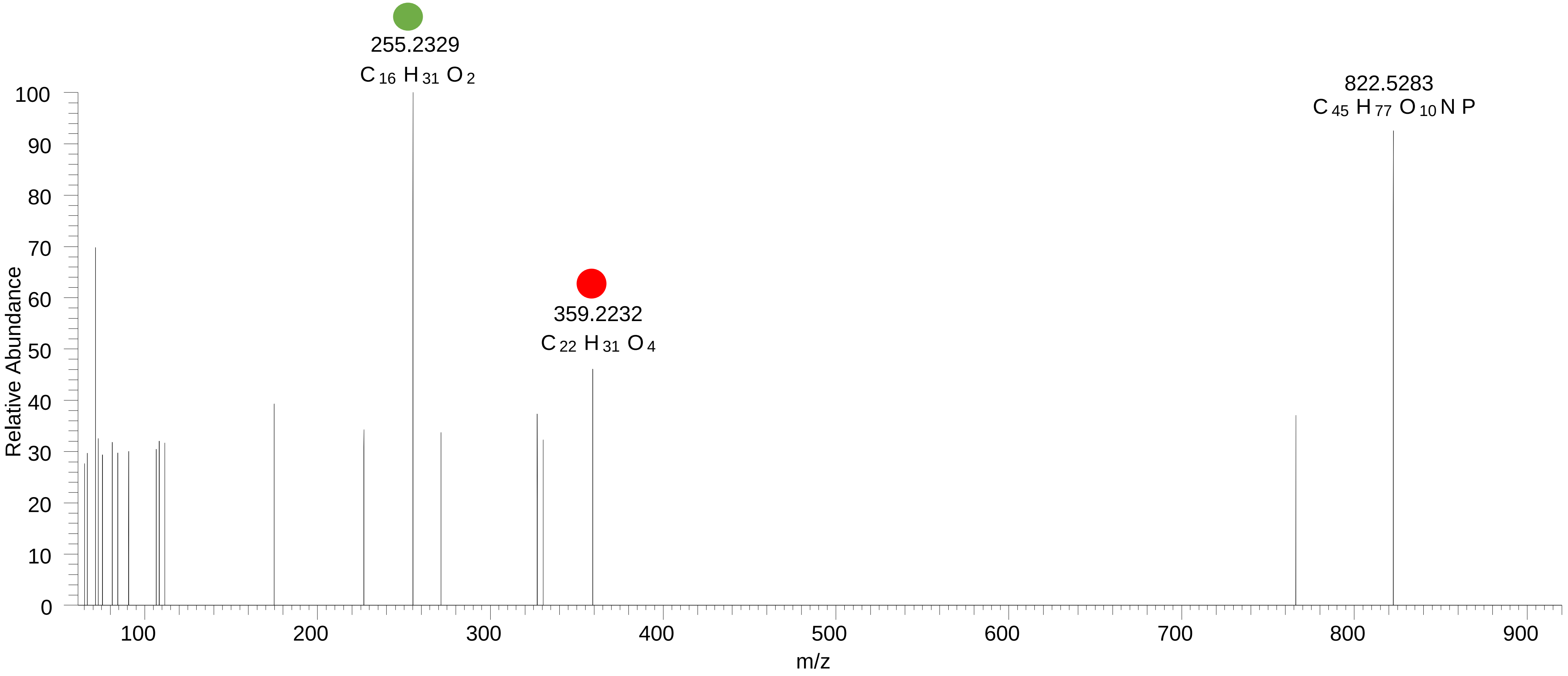

PC(18:0_20:4<2O>)
RT 13.1

[oxPC+HCOO]- XIC 886.5815 NL: 4.43E3

- Fragments containing oxFAs
- Fragments related to water loss
- Fragments not containing oxFAs
- Fragments containing methylated oxFAs
- Fragments related to CO_2 loss
- Position-specific fragments

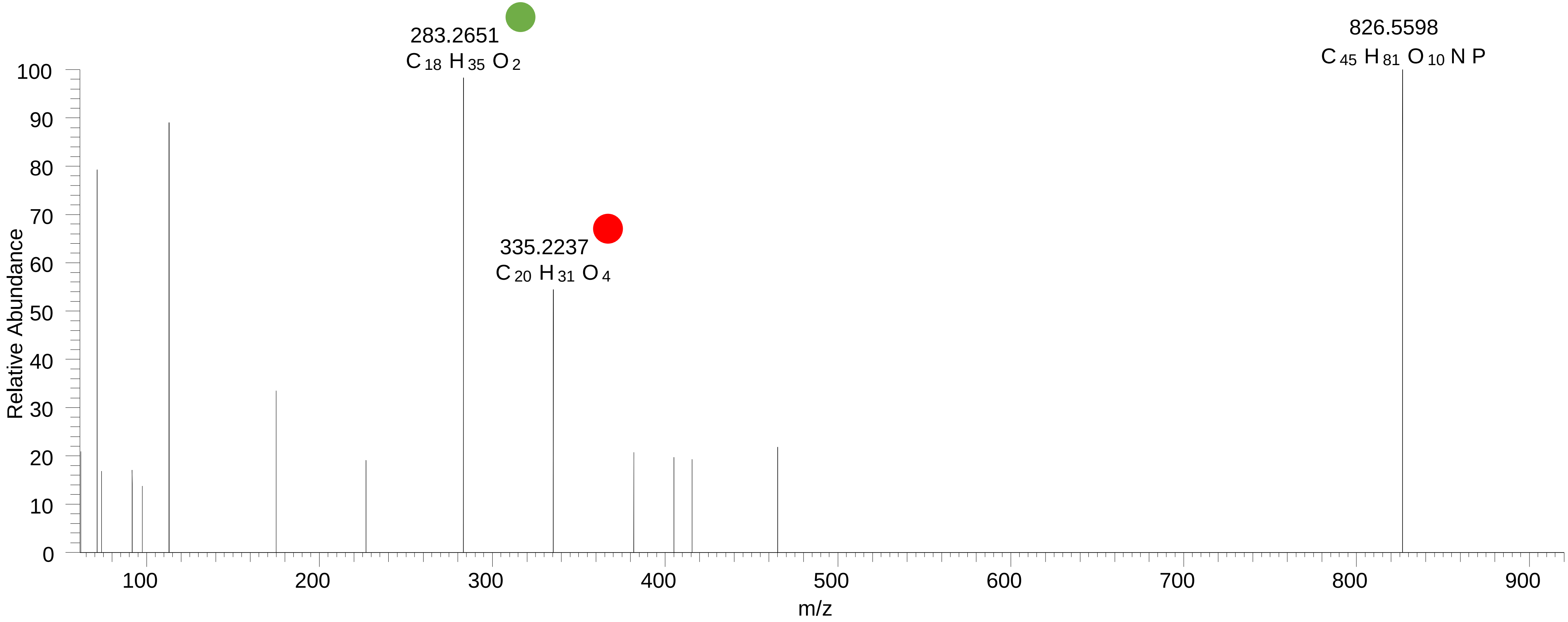

PC(18:0_20:4<OOH{15}>)
RT 15.3

[oxPC+HCOO]- XIC 886.5815 NL: 3.37E3

- Fragments containing oxFAs
- Fragments related to water loss
- Fragments not containing oxFAs
- Fragments containing methylated oxFAs
- Fragments related to CO_2 loss
- Position-specific fragments

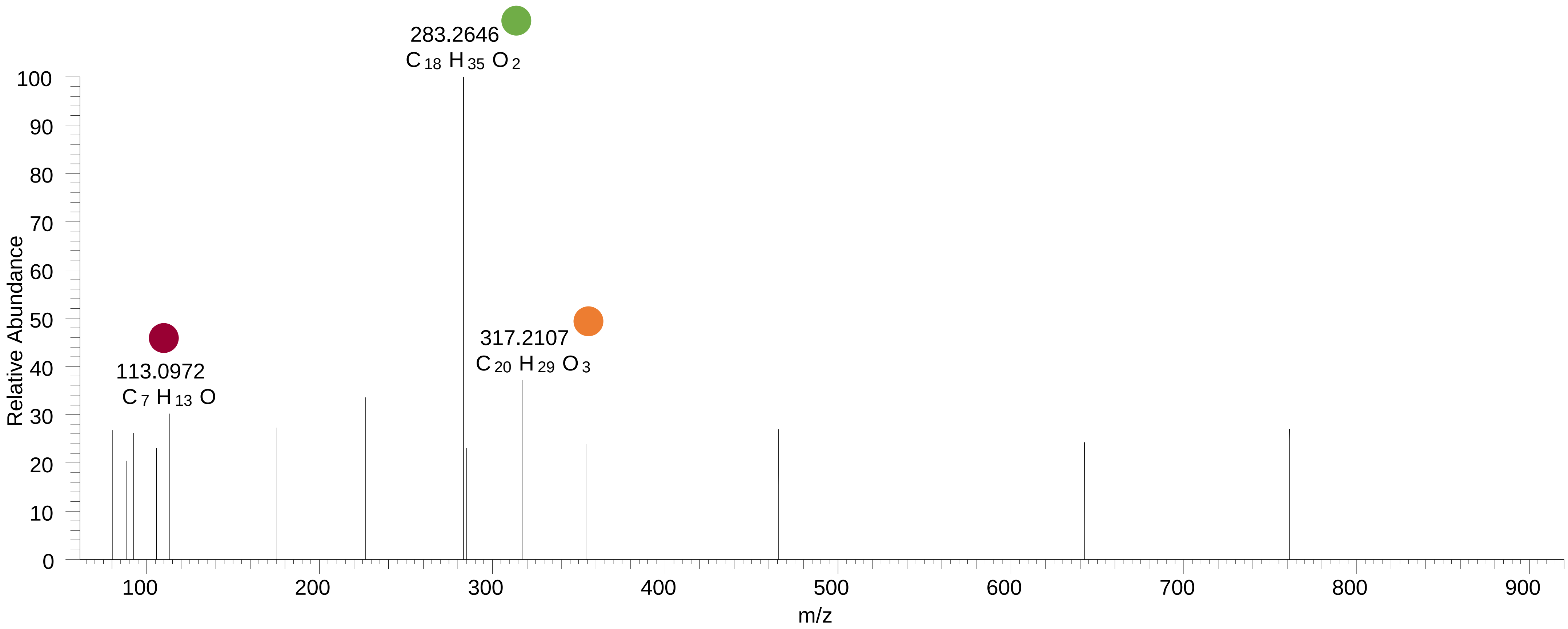

PC(18:0_20:4<OOH>)
RT 15.6

[oxPC+HCOO]- XIC 886.5815 NL: 5.62E3

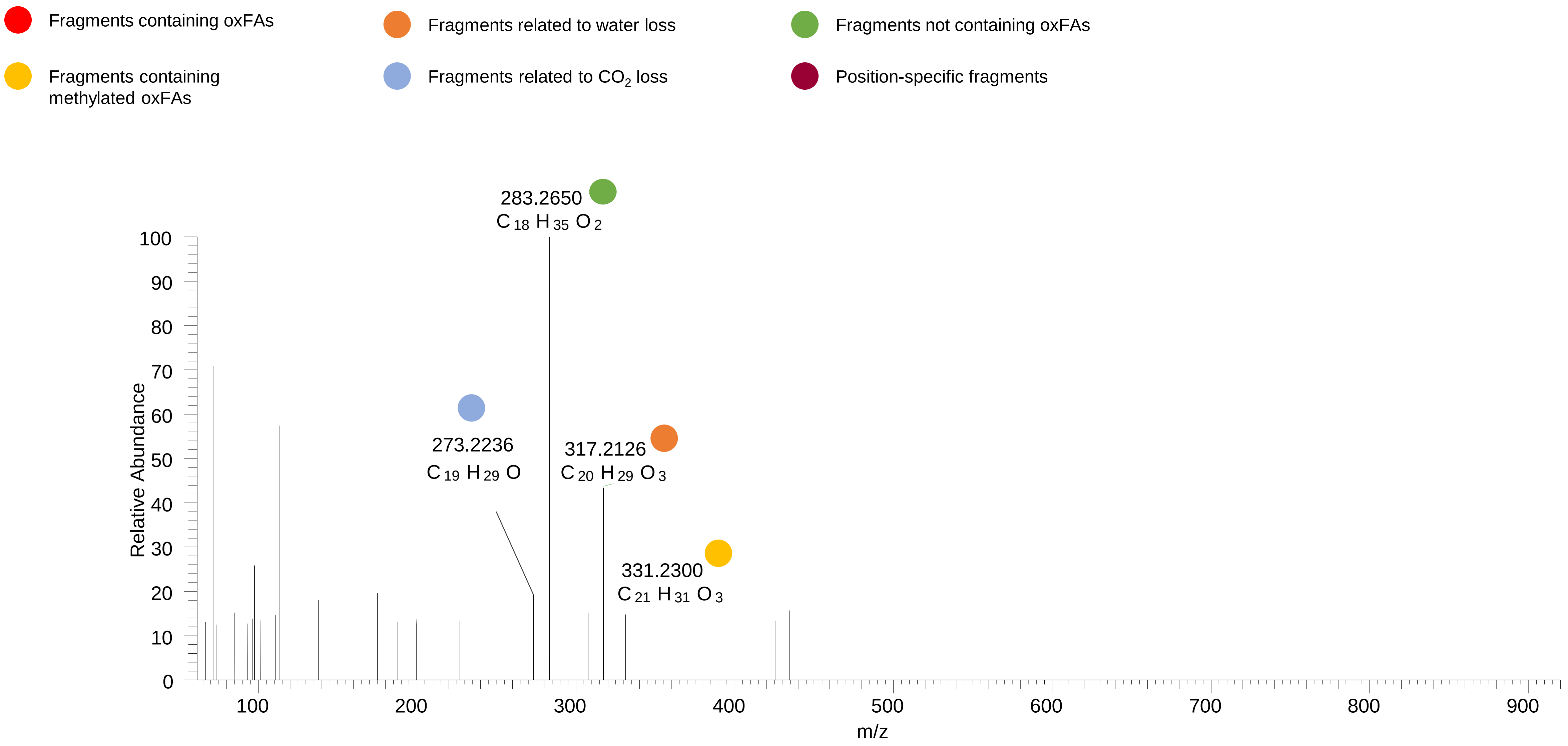

PC(18:0_20:4<OOH{7}>)
RT 16.0

[oxPC+HCOO]- XIC 886.5815 NL: 4.54E3

- Fragments containing oxFAs
- Fragments related to water loss
- Fragments not containing oxFAs
- Fragments containing methylated oxFAs
- Fragments related to CO_2 loss
- Position-specific fragments

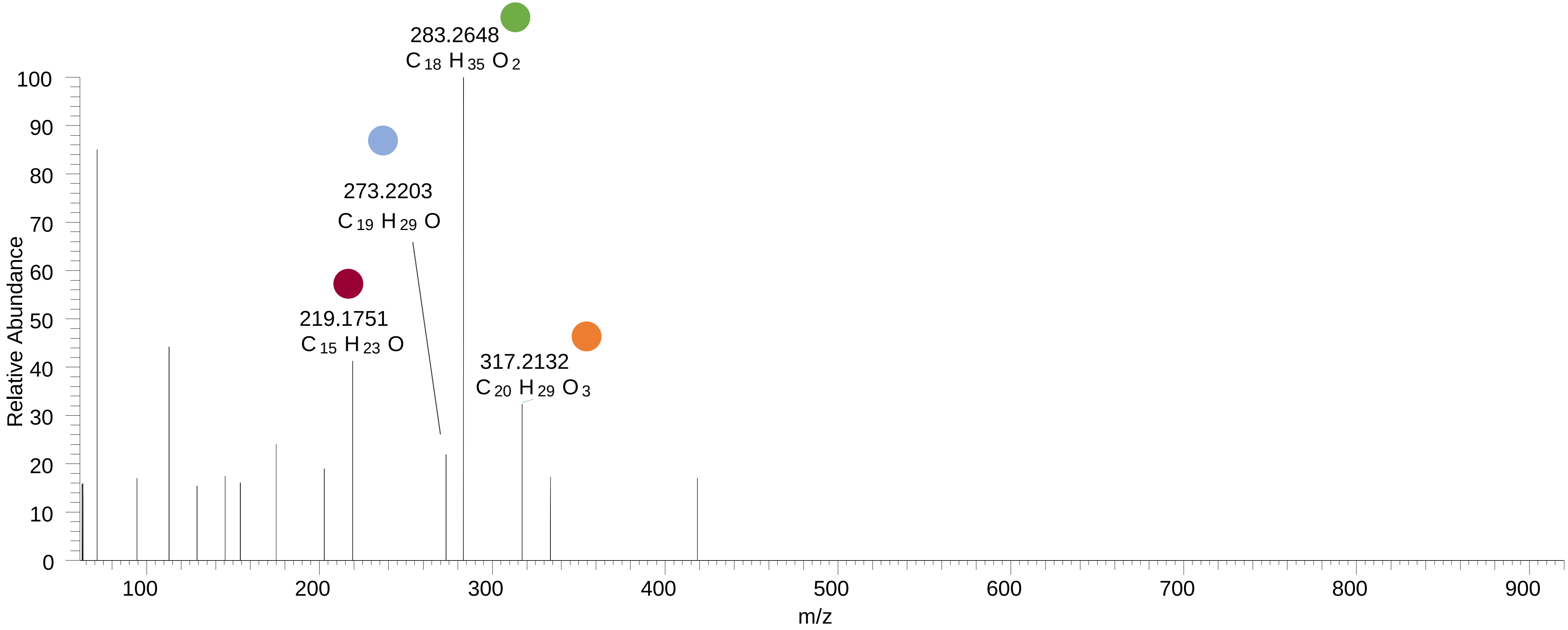

PC(18:0_22:6<O>)
RT 15.2-16.2

[oxPC+HCOO]- XIC 894.5866 NL: 3.96E3

- Fragments containing oxFAs
- Fragments related to water loss
- Fragments not containing oxFAs
- Fragments containing methylated oxFAs
- Fragments related to CO_2 loss
- Position-specific fragments

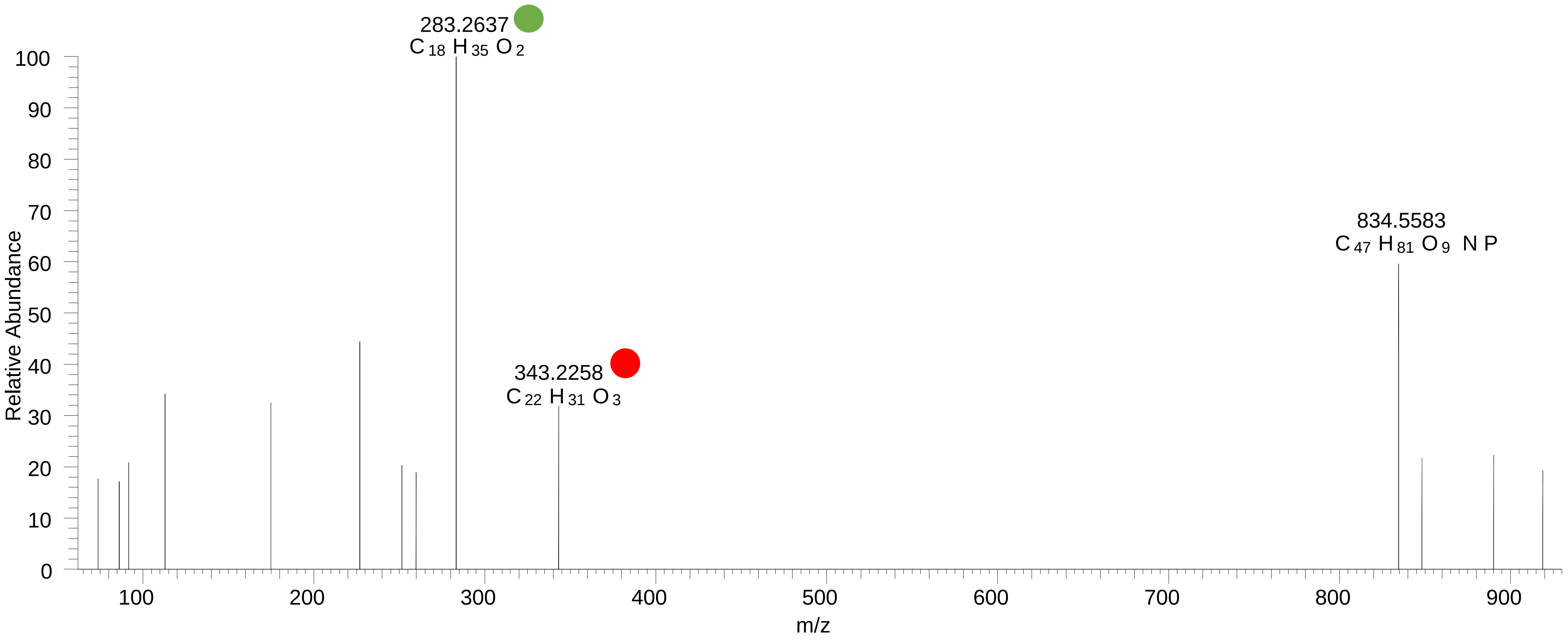

OT2D_pool

PC(16:1_18:2<O>)
RT 11.9-12.0

[oxPC+HCOO]-   XIC 816.5396 NL: 5.11E3

- Fragments containing oxFAs
- Fragments related to water loss
- Fragments not containing oxFAs
- Fragments containing methylated oxFAs
- Fragments related to CO_2 loss
- Position-specific fragments

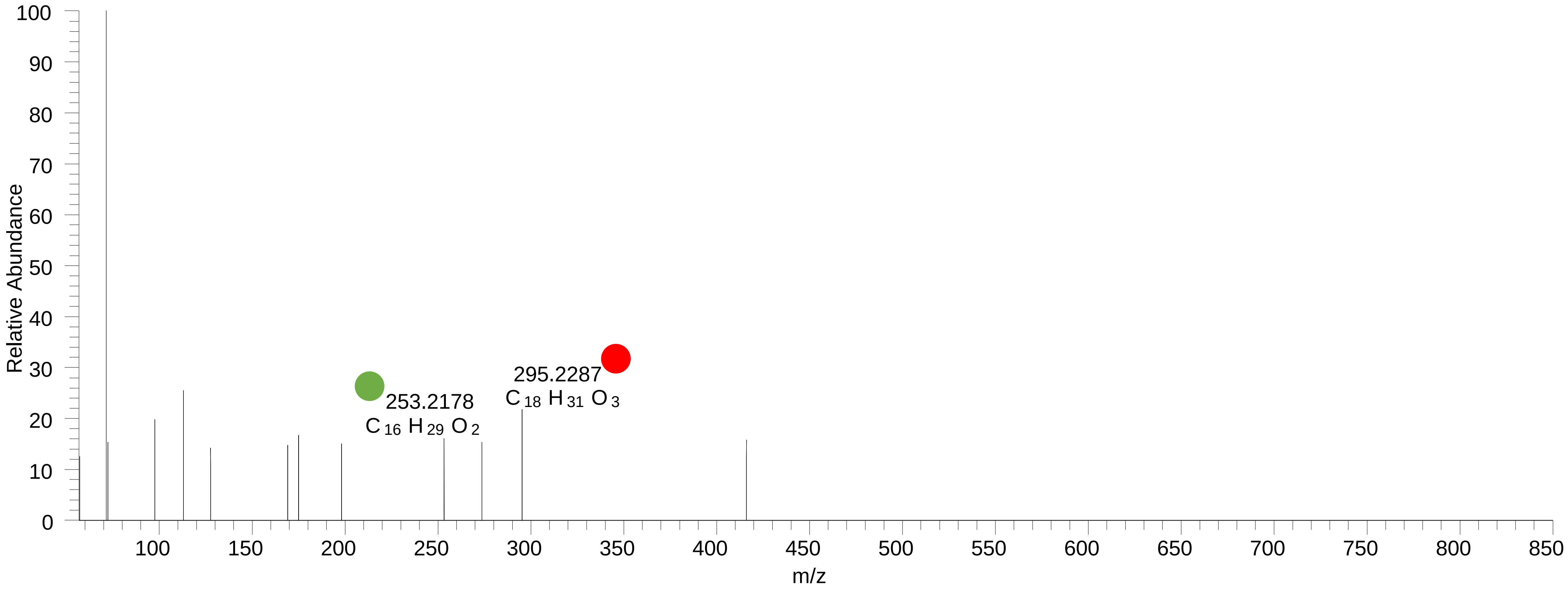

PC(16:0_18:2<OH>)
RT 12.6

[oxPC+HCOO]- XIC 818.5553 NL: 1.70E4

- Fragments containing oxFAs
- Fragments related to water loss
- Fragments not containing oxFAs
- Fragments containing methylated oxFAs
- Fragments related to CO_2 loss
- Position-specific fragments

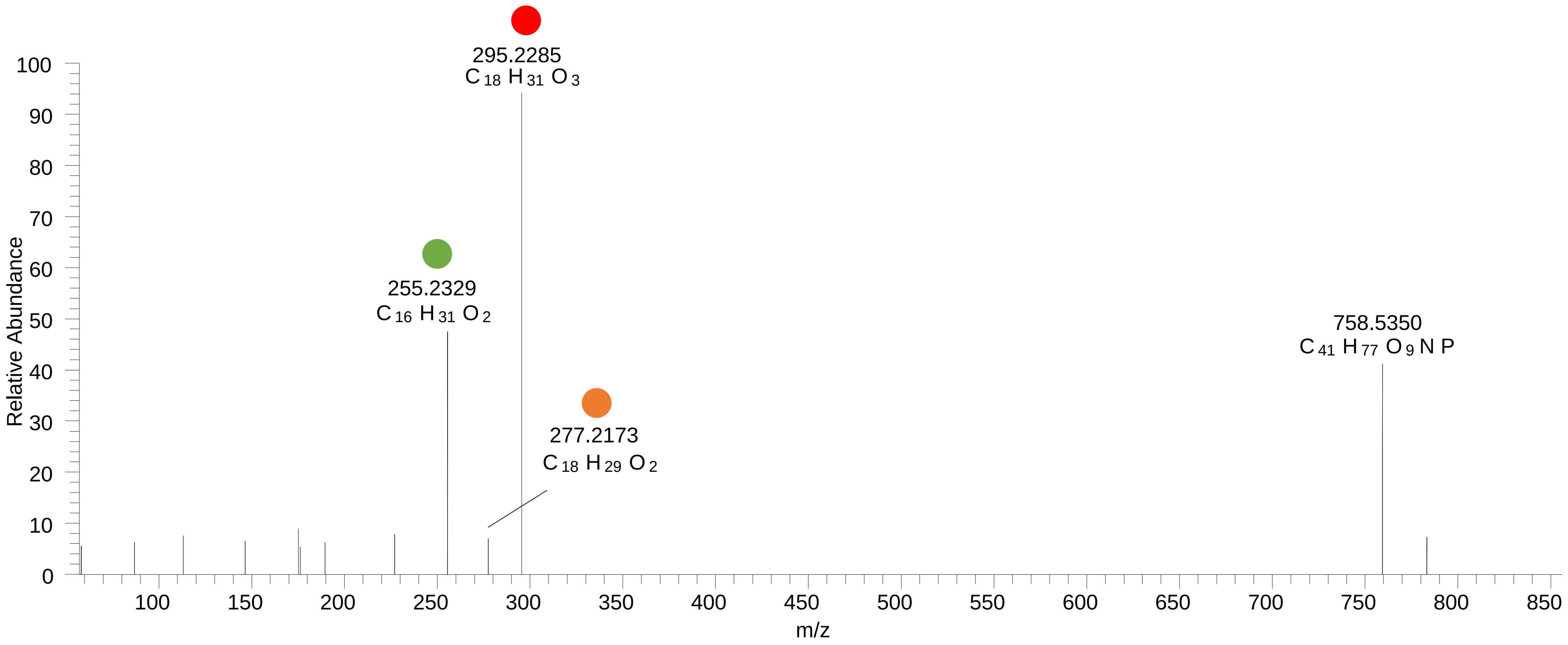

PC(16:0_18:2<OH{13}>)
RT 13.4

[oxPC+HCOO]- XIC 818.5553 NL: 1.27E5

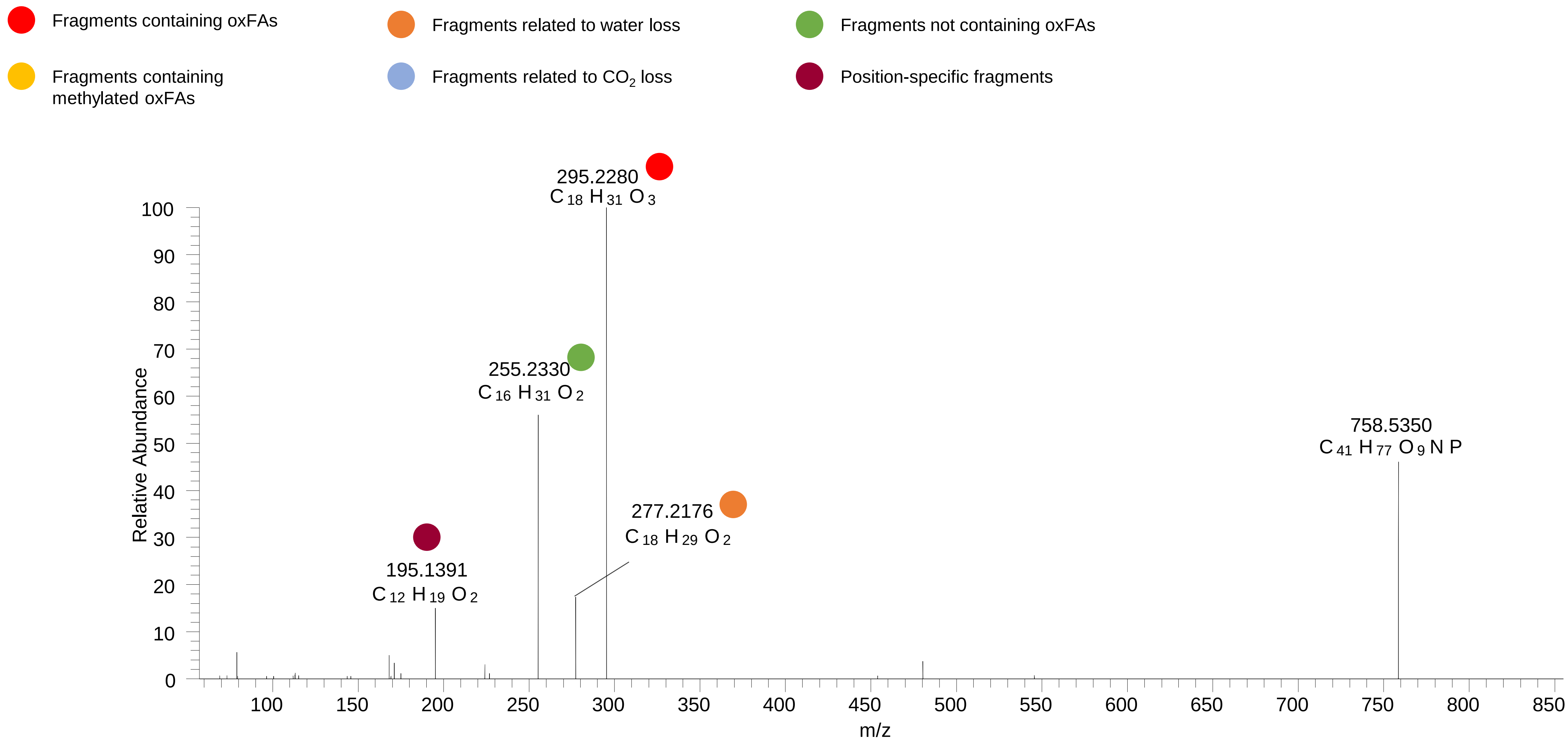

PC(16:0_18:2<OH{9}>)
RT 13.6

[oxPC+HCOO]-   XIC 818.5553 NL: 1.15E5

- Fragments containing oxFAs
- Fragments related to water loss
- Fragments not containing oxFAs
- Fragments containing methylated oxFAs
- Fragments related to CO_2 loss
- Position-specific fragments

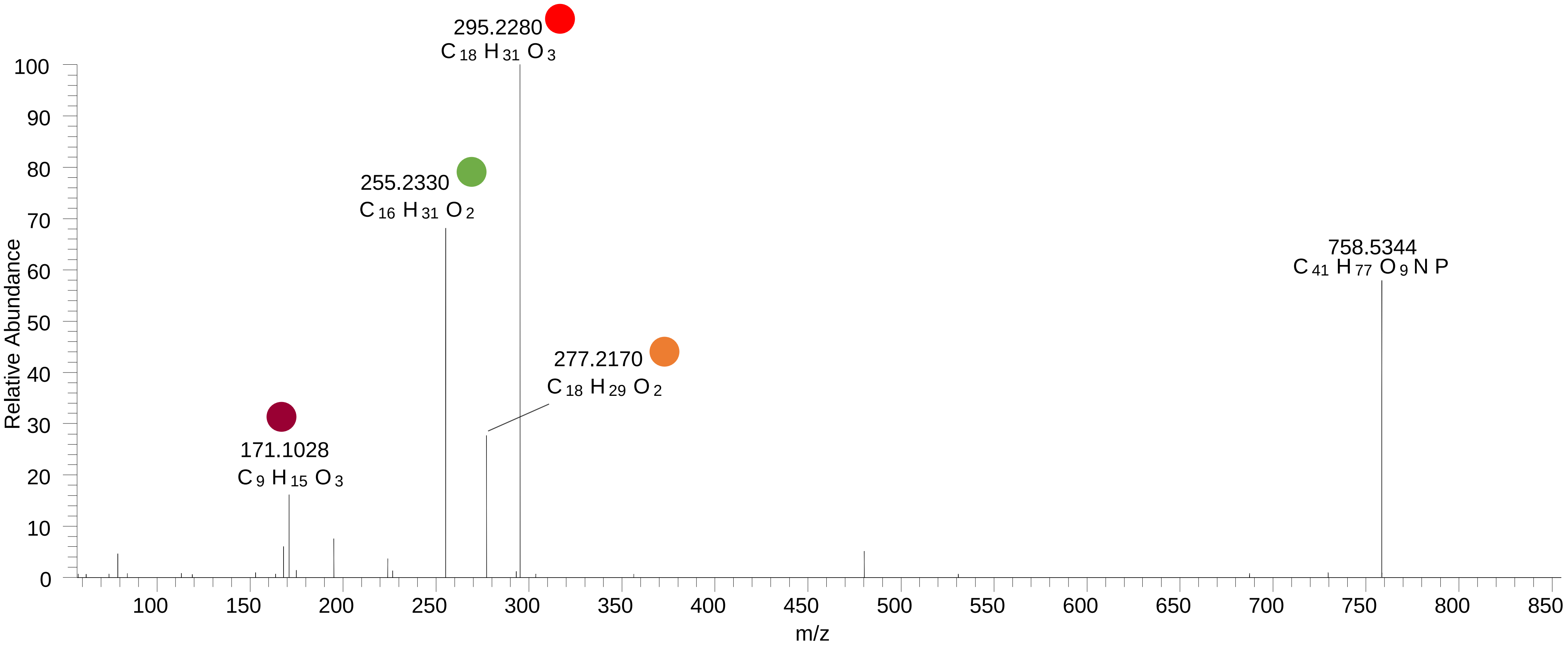

PC(16:0<O>_18:2)
RT 15.7

[oxPC+HCOO]- XIC 818.5553 NL: 4.49E3

- Fragments containing oxFAs
- Fragments related to water loss
- Fragments not containing oxFAs
- Fragments containing methylated oxFAs
- Fragments related to CO_2 loss
- Position-specific fragments

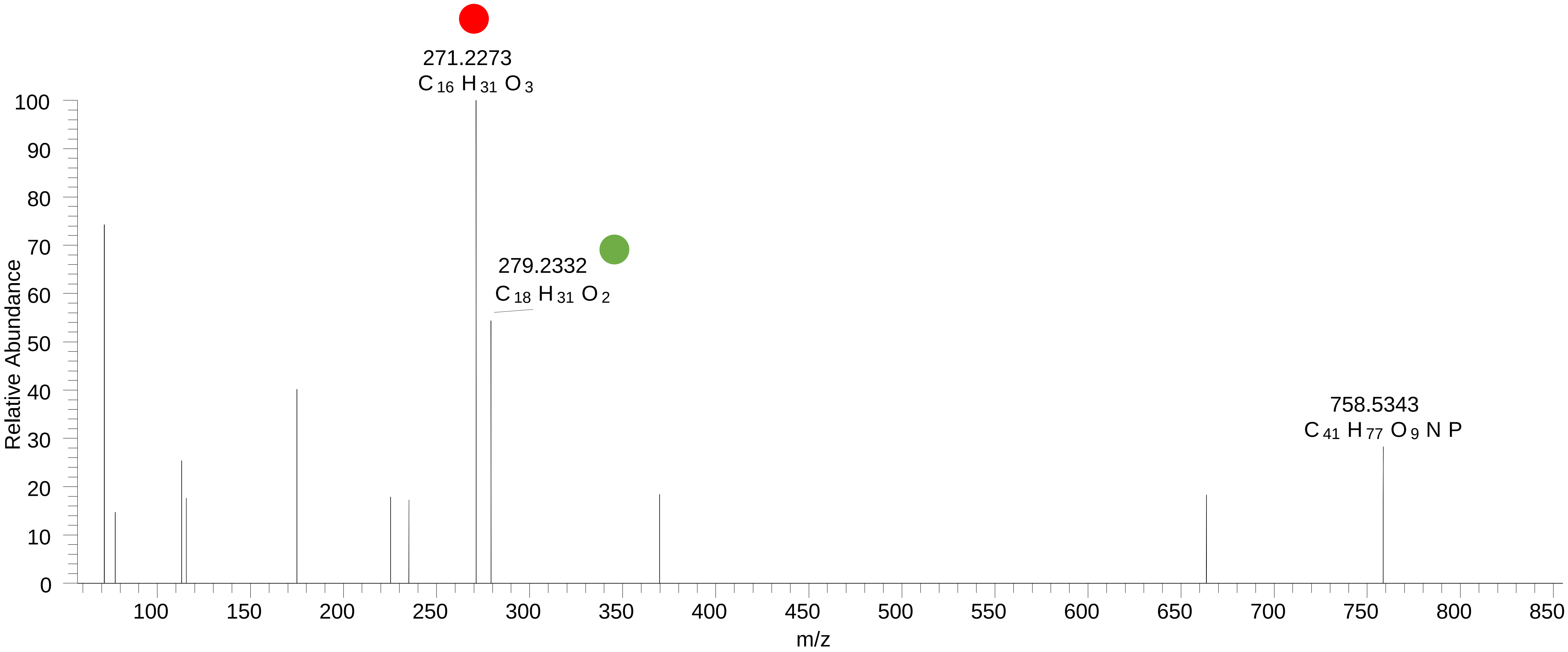

PC(16:0_18:1<O>)
RT 14.4

[oxPC+HCOO]- XIC 820.5709 NL: 2.04E3

- Fragments containing oxFAs
- Fragments related to water loss
- Fragments not containing oxFAs
- Fragments containing methylated oxFAs
- Fragments related to CO_2 loss
- Position-specific fragments

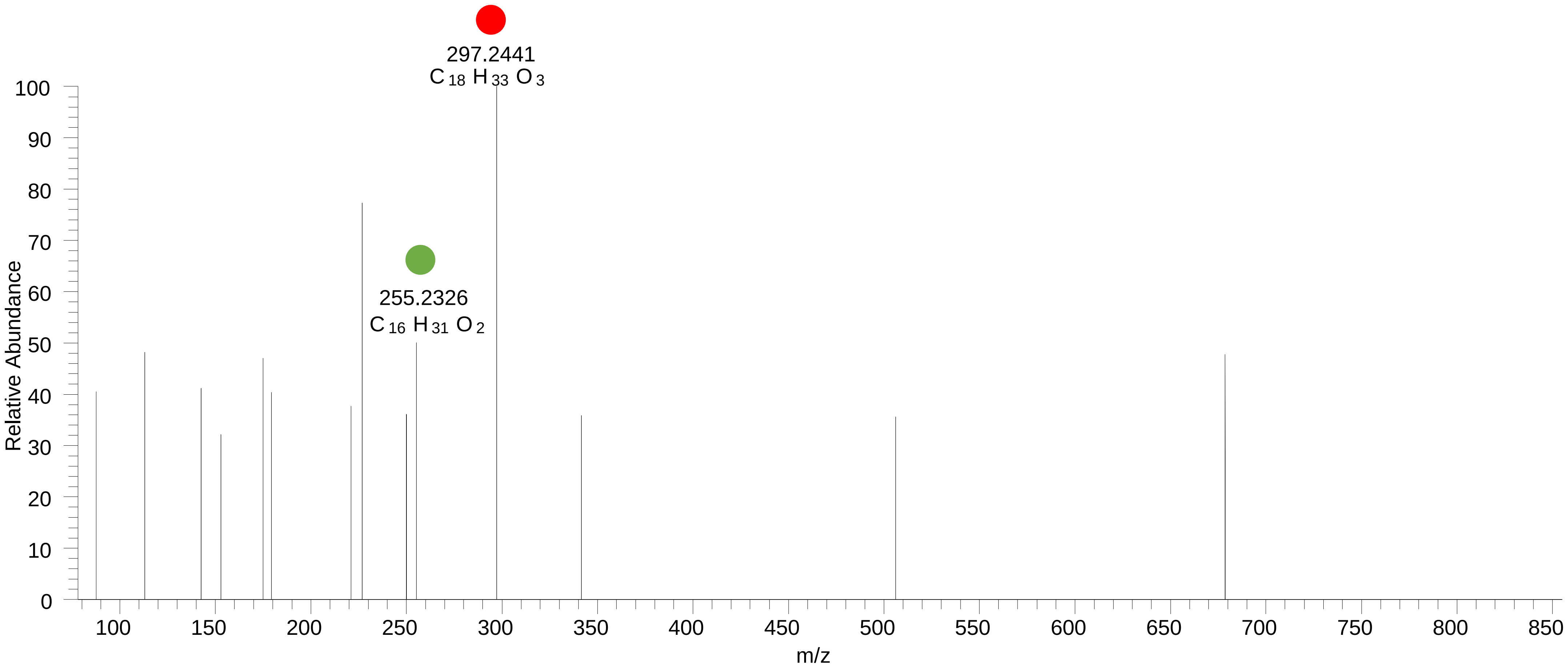

PC(16:0<O>_18:1)
RT 16.8-17.1

[oxPC+HCOO]- XIC 820.5709 NL: 4.78E3

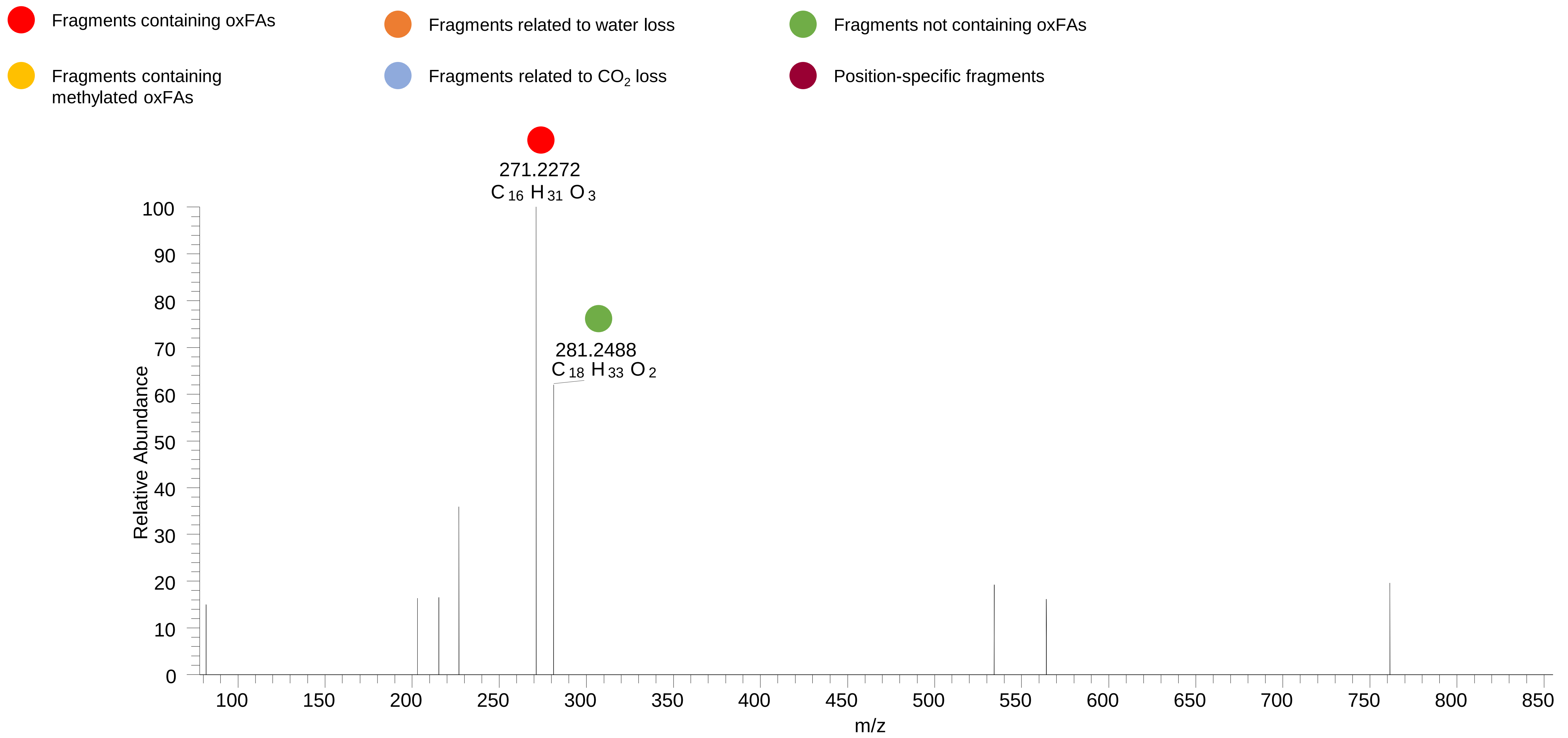

PC(16:0_18:2<OH,O{9}>)
RT 11.6

[oxPC+HCOO]- XIC 834.5502 NL: 4.04E3

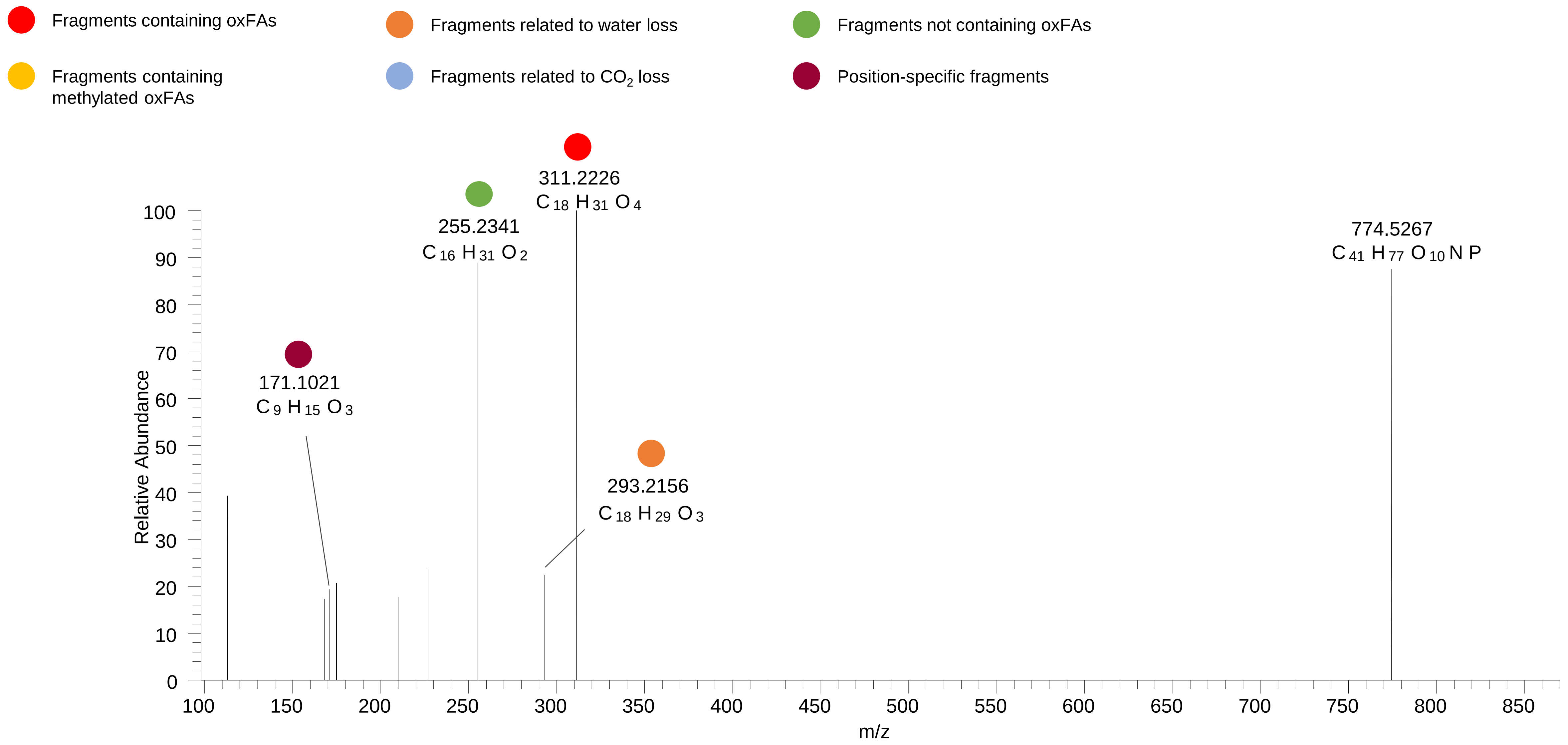

PC(16:0_18:2<2O>)
RT 12.0

[oxPC+HCOO]- XIC 834.5502 NL: 2.57E3

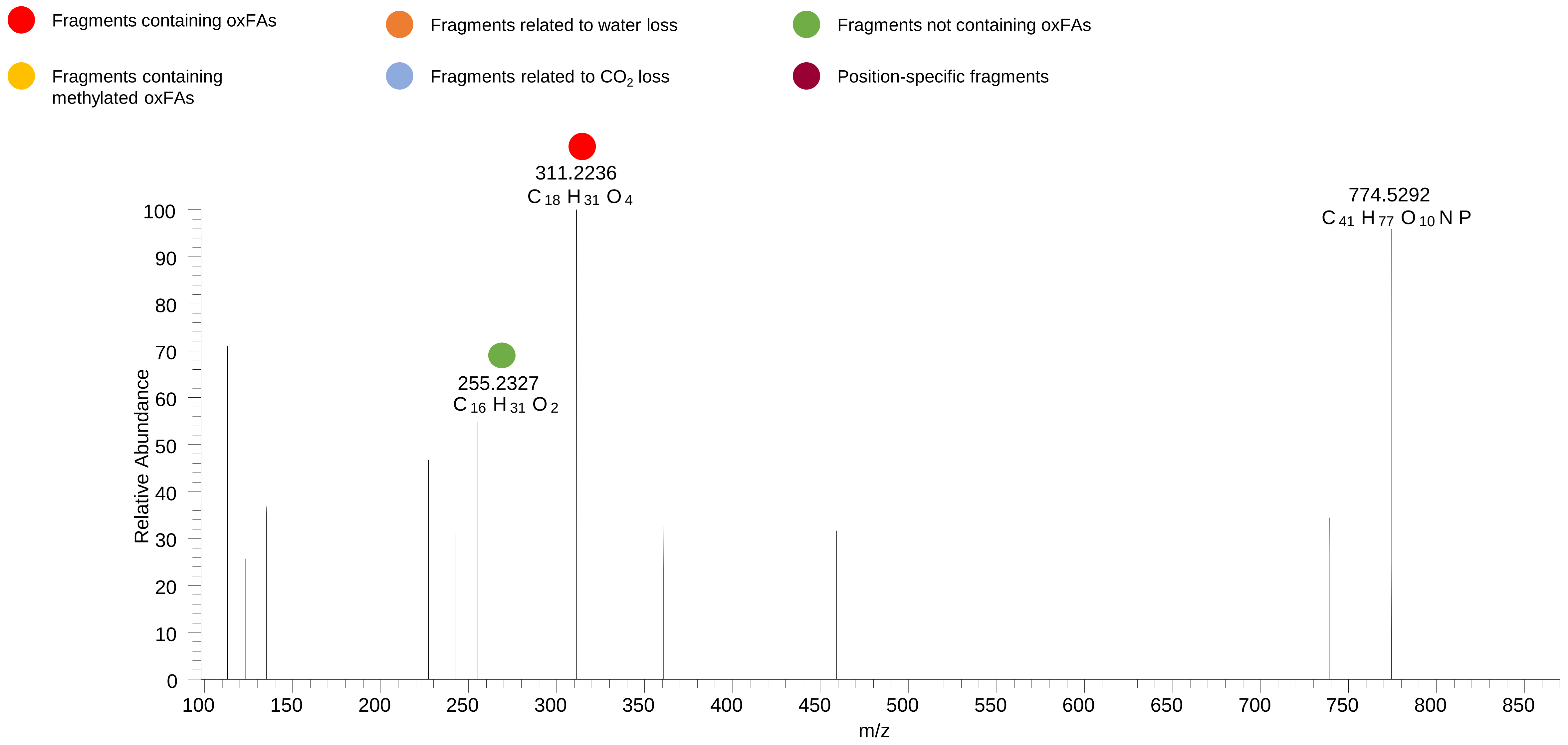

PC(16:0_18:2<OOH>)
RT 12.6

[oxPC+HCOO]- XIC 834.5502 NL: 2.31E3

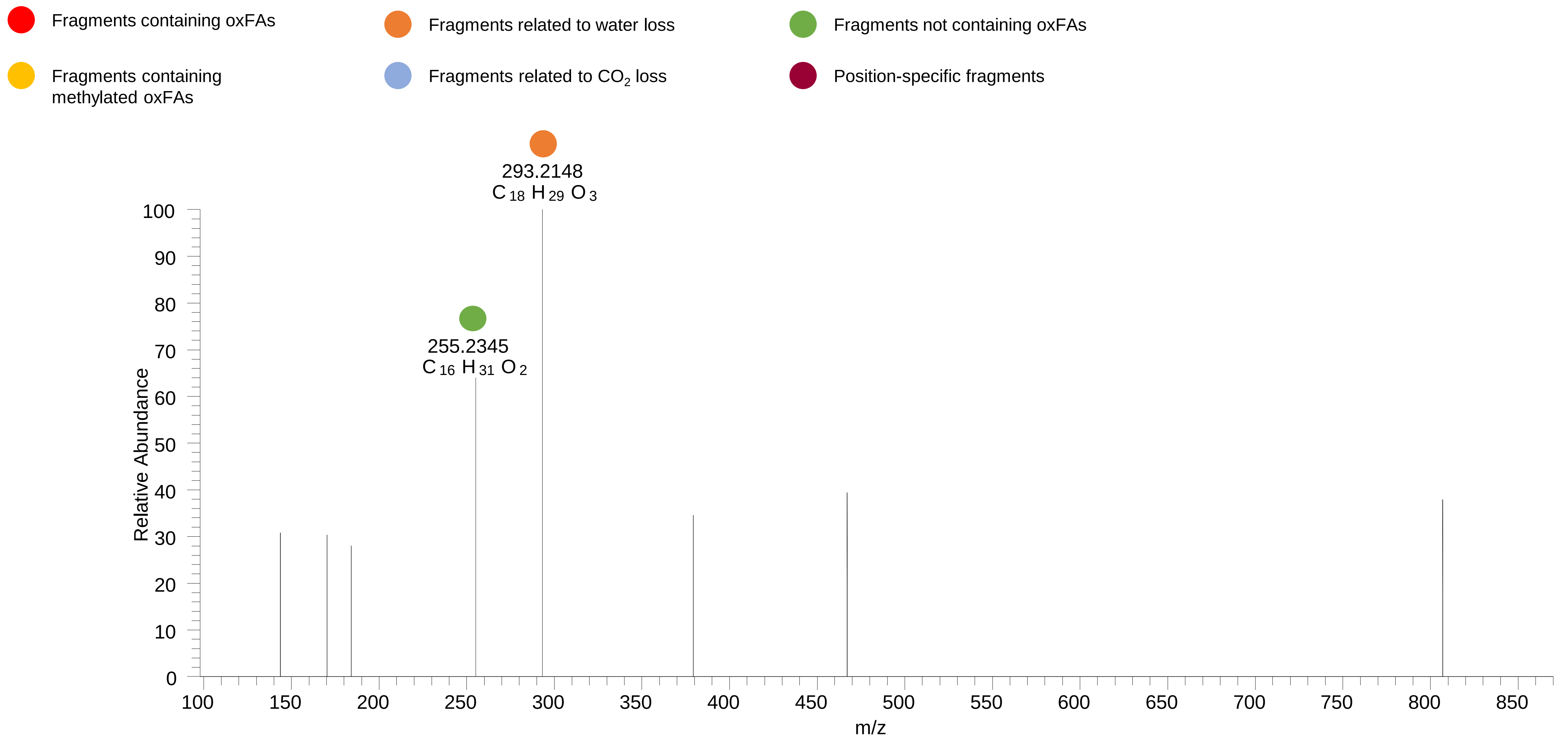

PC(16:0_18:2<OOH{9}>)
PC(16:0_18:2<OOH{13}>)
RT 13.2-13.4

[oxPC+HCOO]- XIC 834.5502 NL: 4.09E4

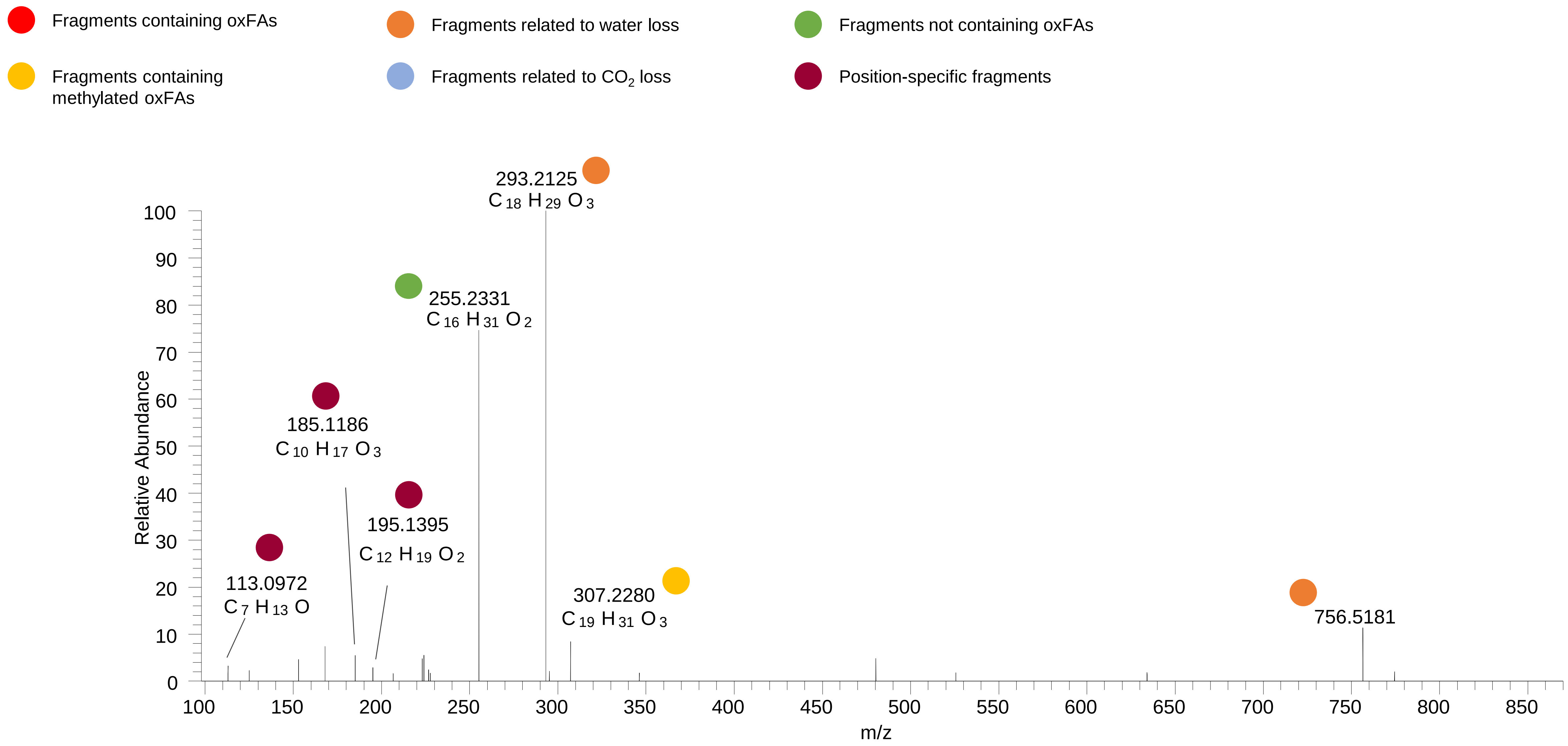

PC(16:0_18:1<OOH>)
RT 14.2-14.3

[oxPC+HCOO]- XIC 836.5658 NL: 1.46E3

- Fragments containing oxFAs
- Fragments related to water loss
- Fragments not containing oxFAs
- Fragments containing methylated oxFAs
- Fragments related to CO_2 loss
- Position-specific fragments

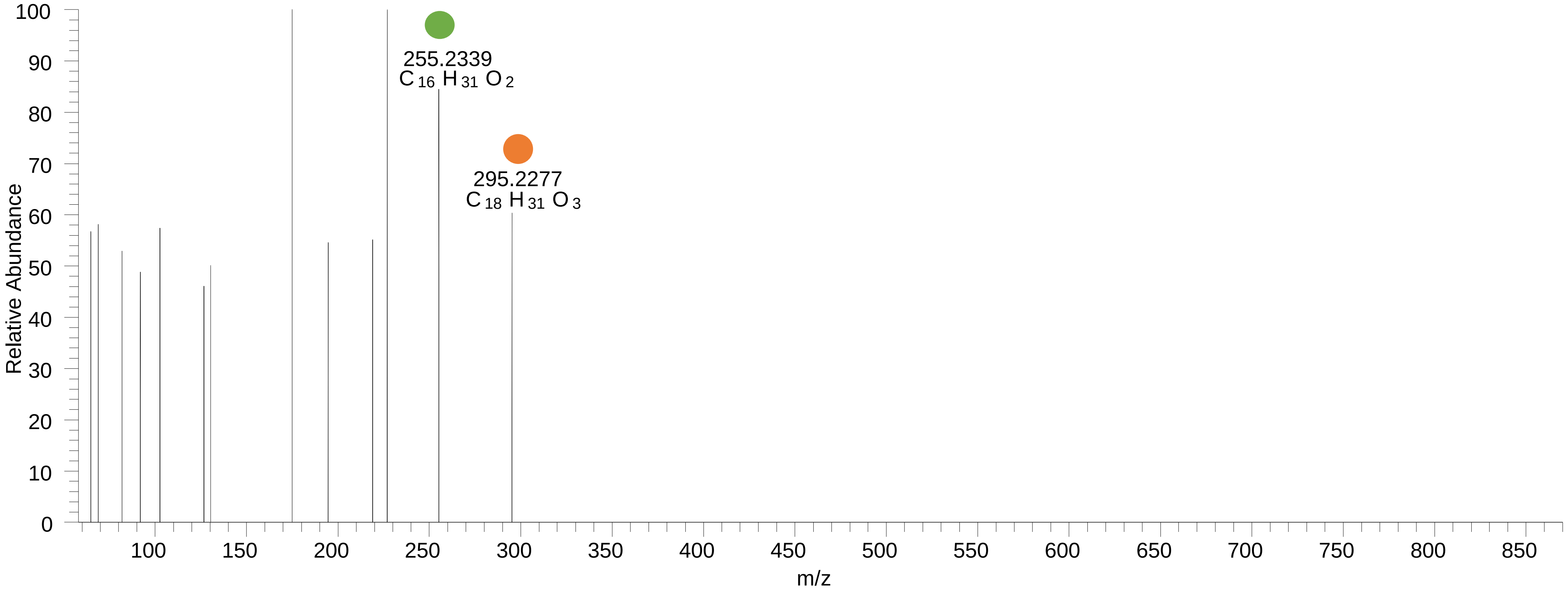

PC(16:0_20:5<O>)
RT 12.8

[oxPC+HCOO]- XIC 840.5396 NL: 1.85E3

Fragments containing oxFAs

Fragments related to water loss

Fragments not containing oxFAs

Fragments containing methylated oxFAs

Fragments related to CO_2 loss

Position-specific fragments

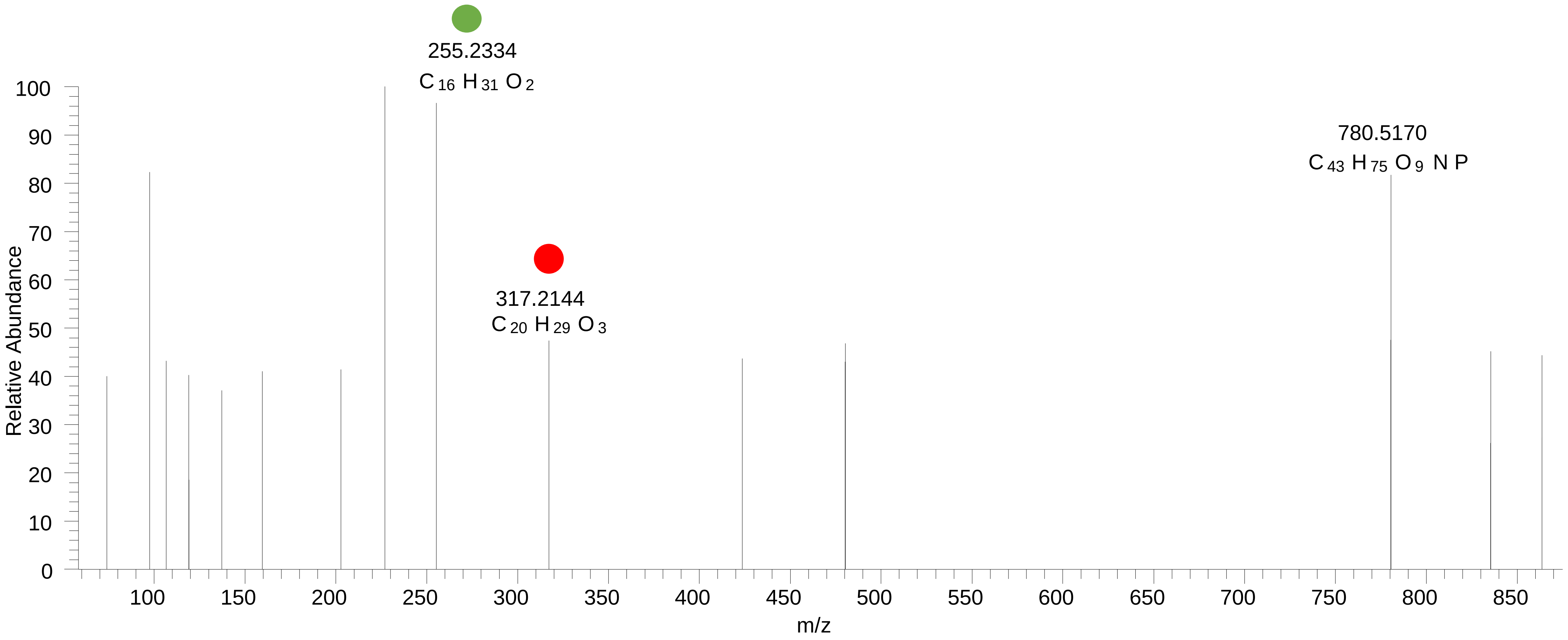

PC(16:0_20:4<OH{13}>)
RT 13.2

[oxPC+HCOO]- XIC 842.5553 NL: 5.08E3

- Fragments containing oxFAs
- Fragments related to water loss
- Fragments not containing oxFAs
- Fragments containing methylated oxFAs
- Fragments related to CO_2 loss
- Position-specific fragments

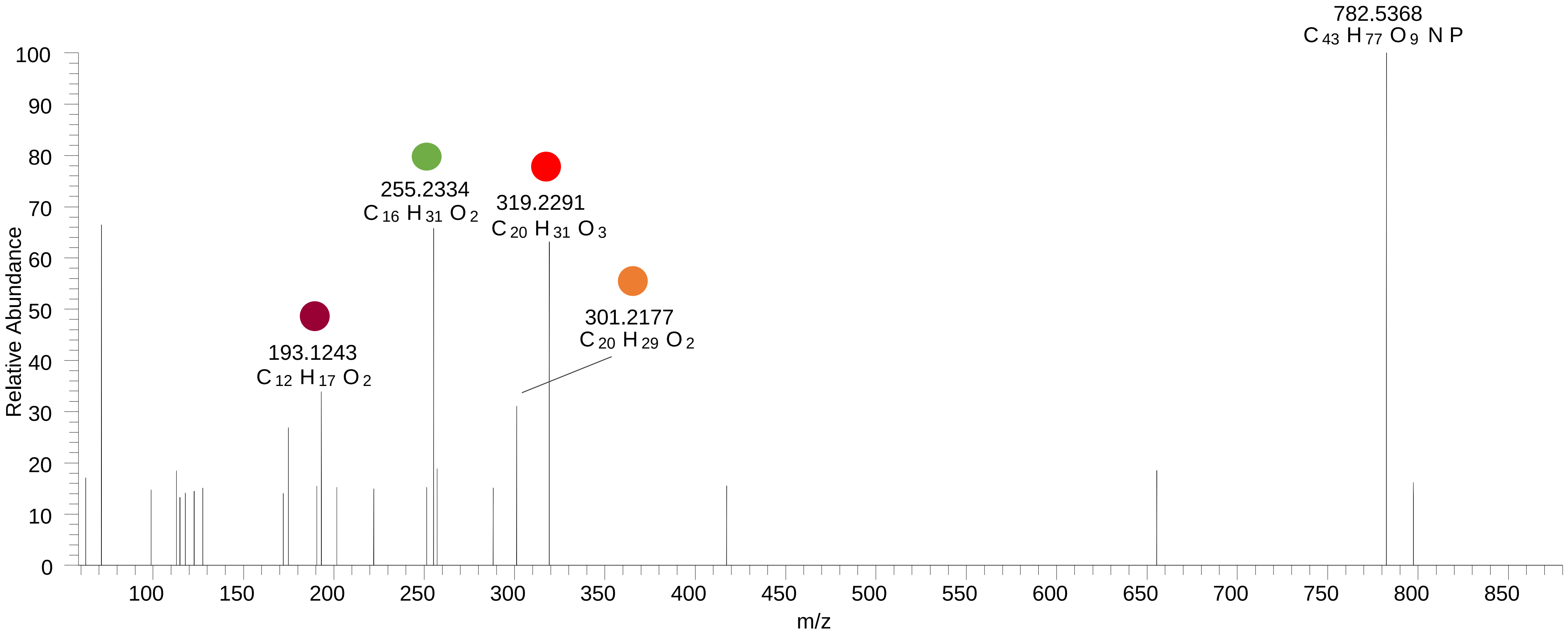

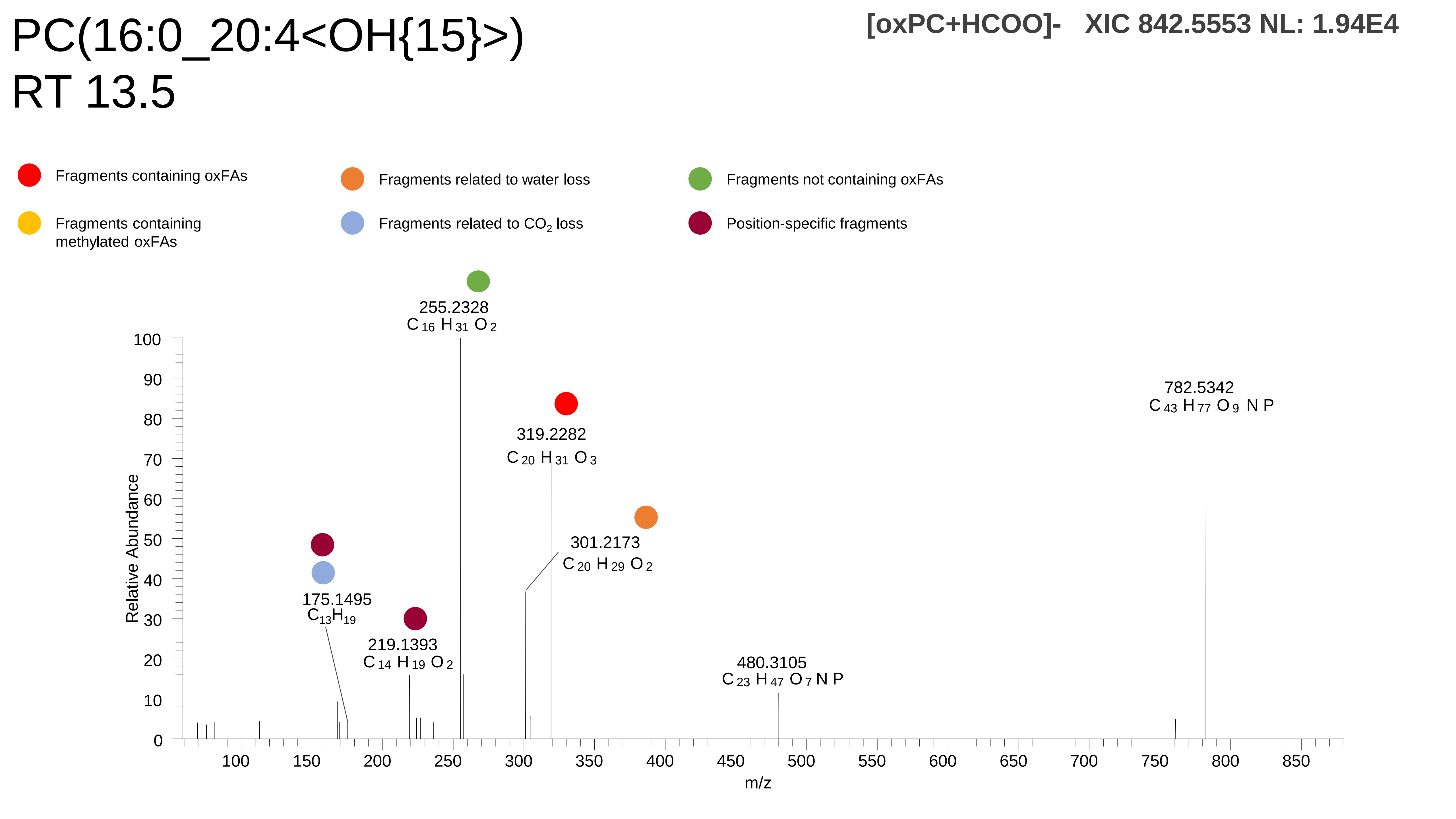
PC(16:0_20:4<OH{15}>)
RT 13.5
[oxPC+HCOO]- XIC 842.5553 NL: 1.94E4
Fragments containing oxFAs
Fragments related to water loss
Fragments not containing oxFAs
Fragments containing methylated oxFAs
Fragments related to CO2 loss
Position-specific fragments
255.2328
C 16 H 31 O 2
319.2282
C 20 H 31 O 3
301.2173
C 20 H 29 O 2
175.1495
C13H19
219.1393
C 14 H 19 O 2
480.3105
C 23 H 47 O 7 N P
782.5342
C 43 H 77 O 9 N P
Relative Abundance
m/z

PC(16:0_20:4<OH{11}>)
RT 13.8

[oxPC+HCOO]- XIC 842.5553 NL: 2.09E4

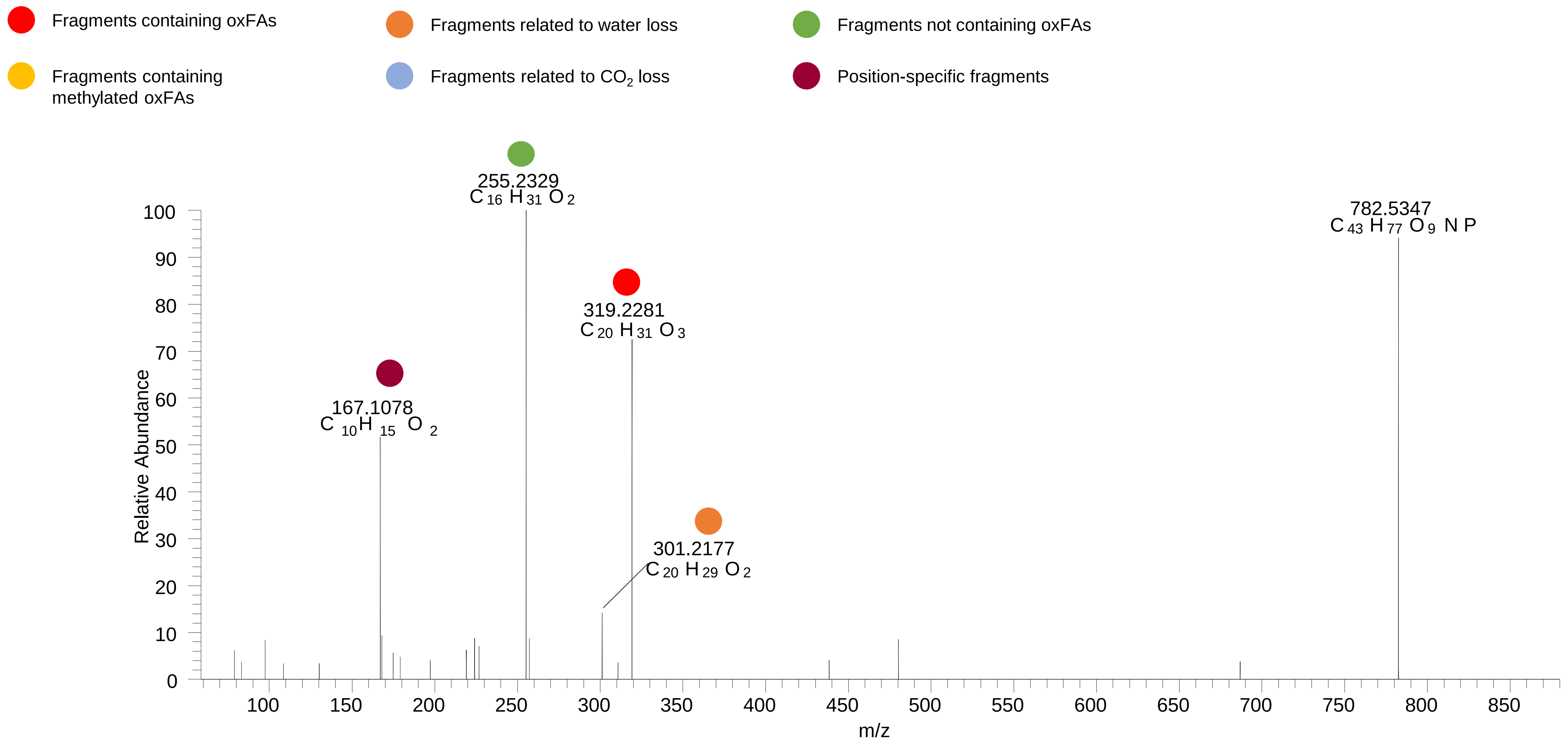

PC(16:0_20:4<OH{11}>)
PC(16:0_20:4<OH{12}>)
RT 14.0

[oxPC+HCOO]- XIC 842.5553 NL: 3.42E4

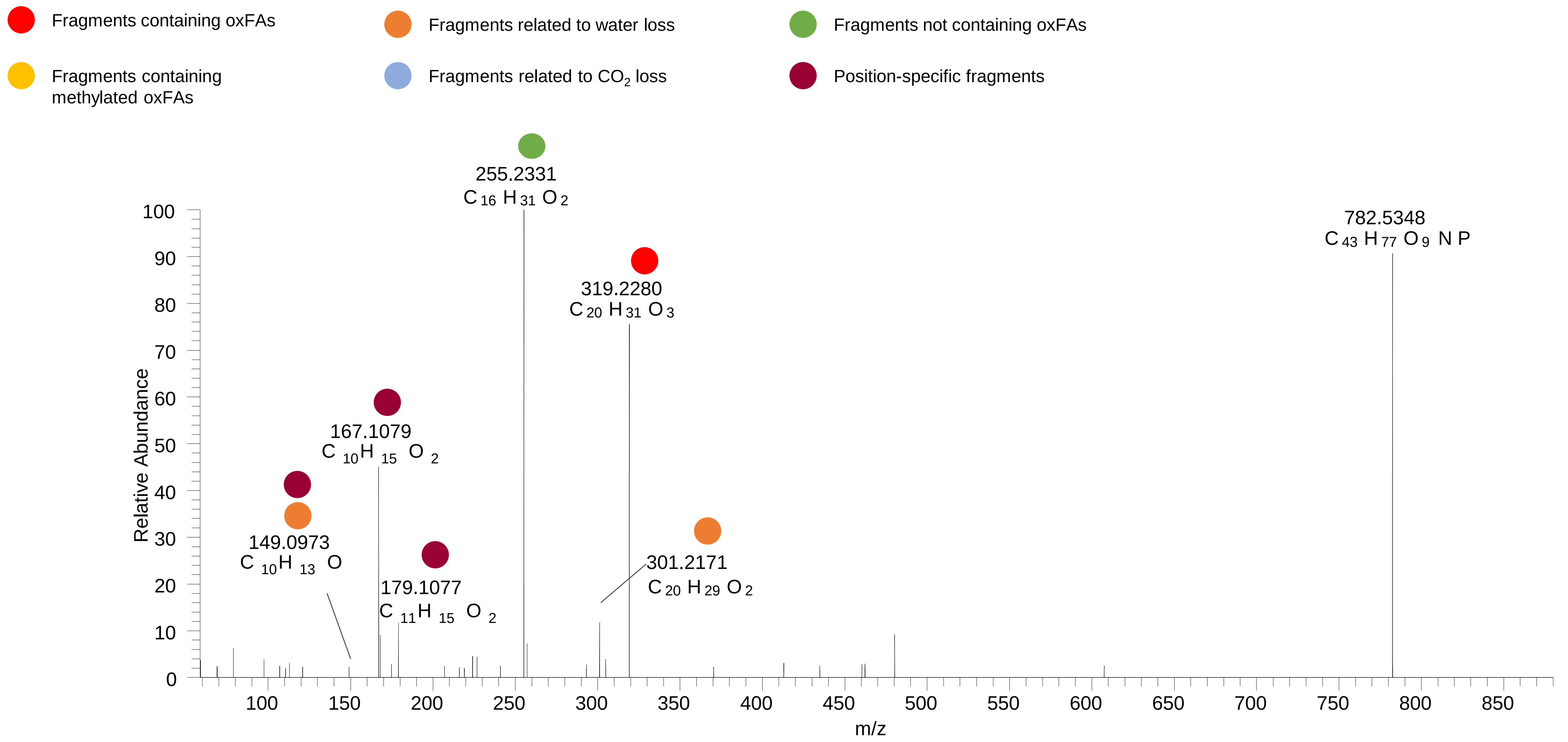

PC(16:0_20:4<OH{8}>)
PC(16:0_20:4<OH{7}>)
RT 14.3-14.4

[oxPC+HCOO]- XIC 842.5553 NL: 2.72E4

Fragments containing oxFAs
Fragments related to water loss
Fragments not containing oxFAs
Fragments containing methylated oxFAs
Fragments related to CO_2 loss
Position-specific fragments

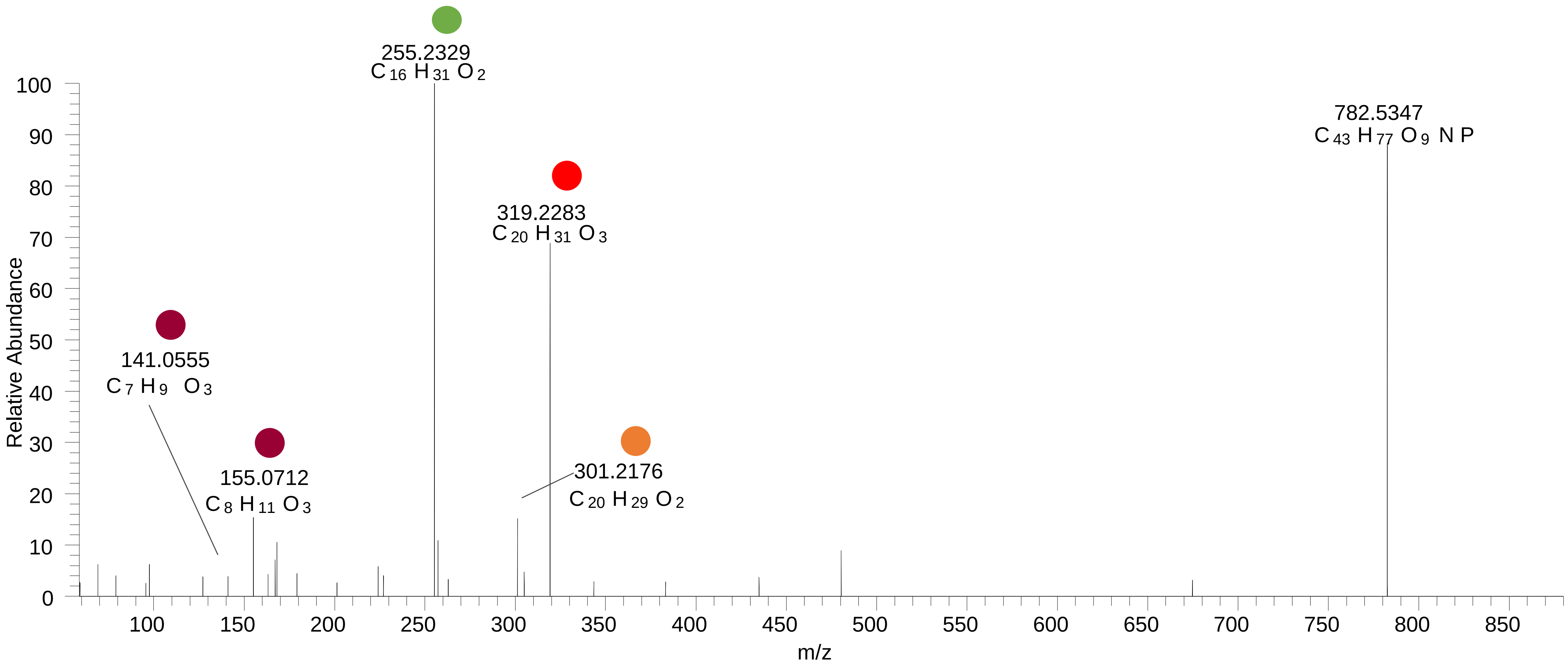

PC(16:0_20:4<OH{5}>)

RT 15.0

[oxPC+HCOO]- XIC 842.5553 NL: 9.39E3

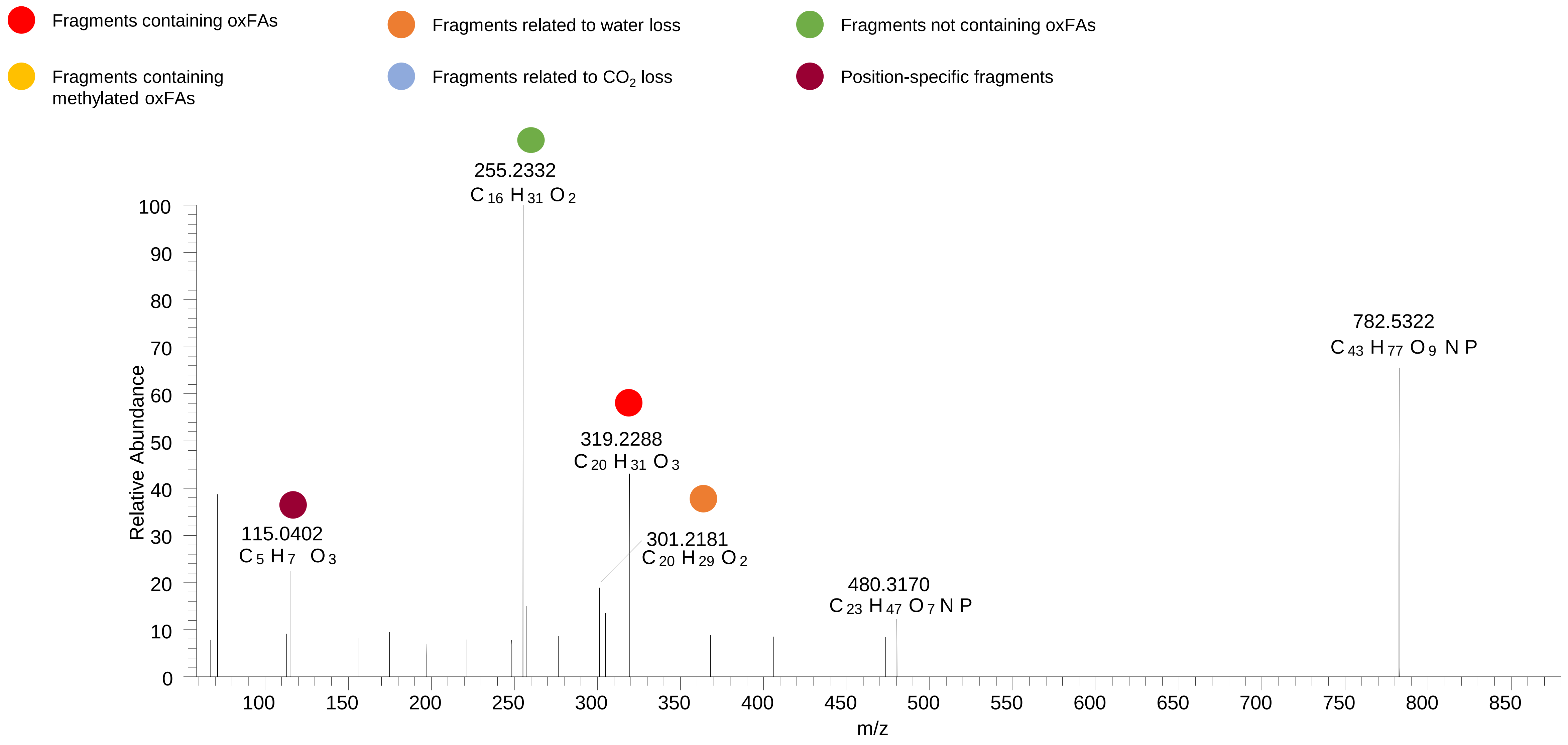

PC(18:1_18:2<O>)
RT 13.0

[oxPC+HCOO]- XIC 844.5709 NL: 2.30E3

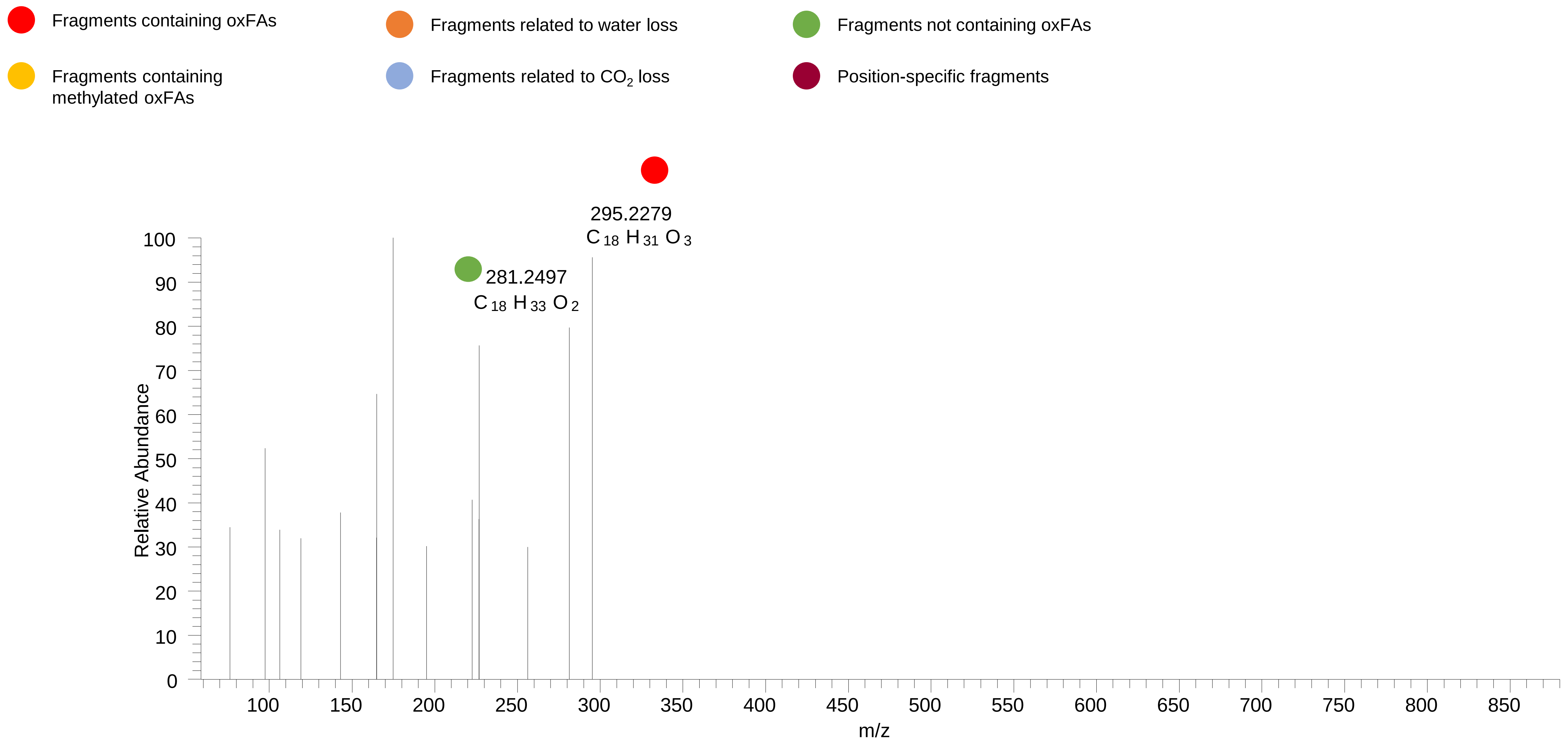

PC(16:0_20:3<OH{11}>)
PC(16:0_20:3<OH{12}>)
RT 14.2-14.3

[oxPC+HCOO]- XIC 844.5709 NL: 2.58E4

- Fragments containing oxFAs
- Fragments related to water loss
- Fragments not containing oxFAs
- Fragments containing methylated oxFAs
- Fragments related to CO_2 loss
- Position-specific fragments

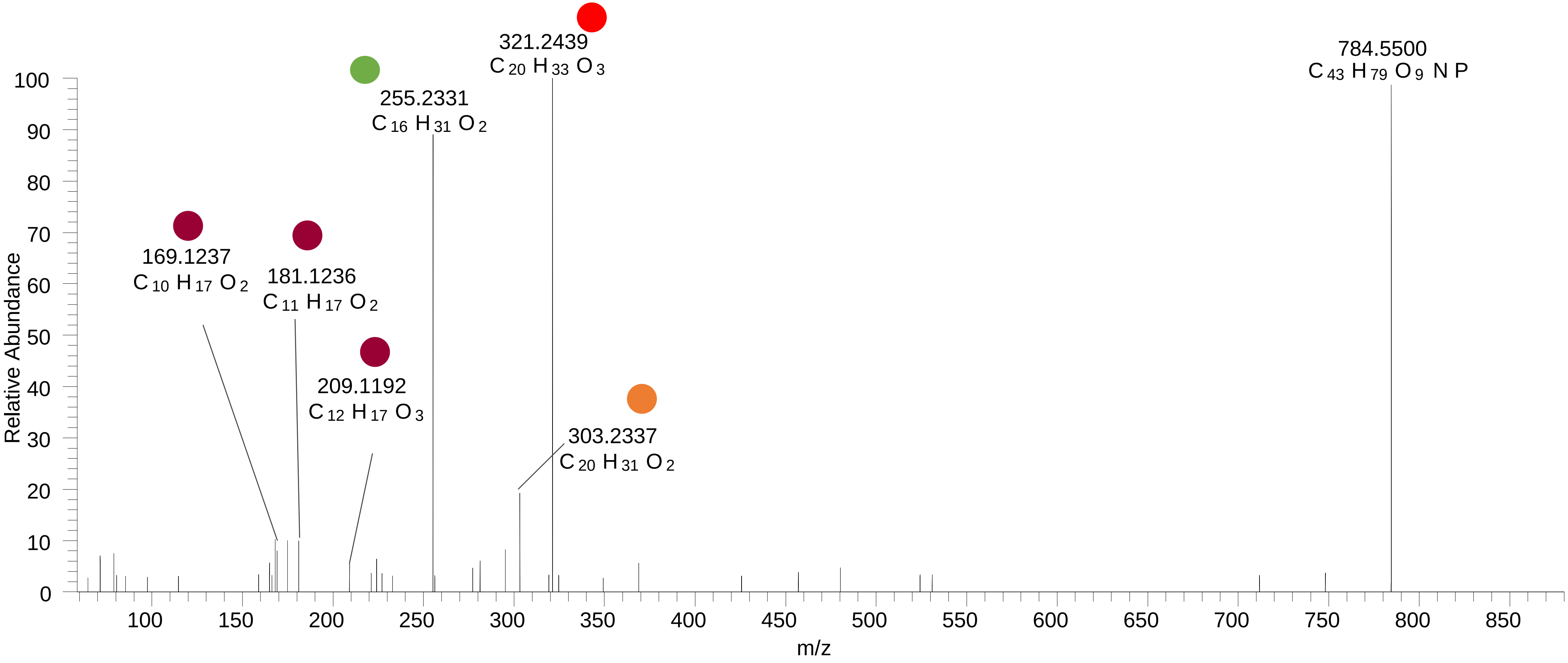

PC(16:0_20:4<2O>)
RT 11.2, 12.0, 12.6

[oxPC+HCOO]- XIC 858.5502 NL: 4.43E3

Fragments containing oxFAs

Fragments related to water loss

Fragments not containing oxFAs

Fragments containing methylated oxFAs

Fragments related to CO_2 loss

Position-specific fragments

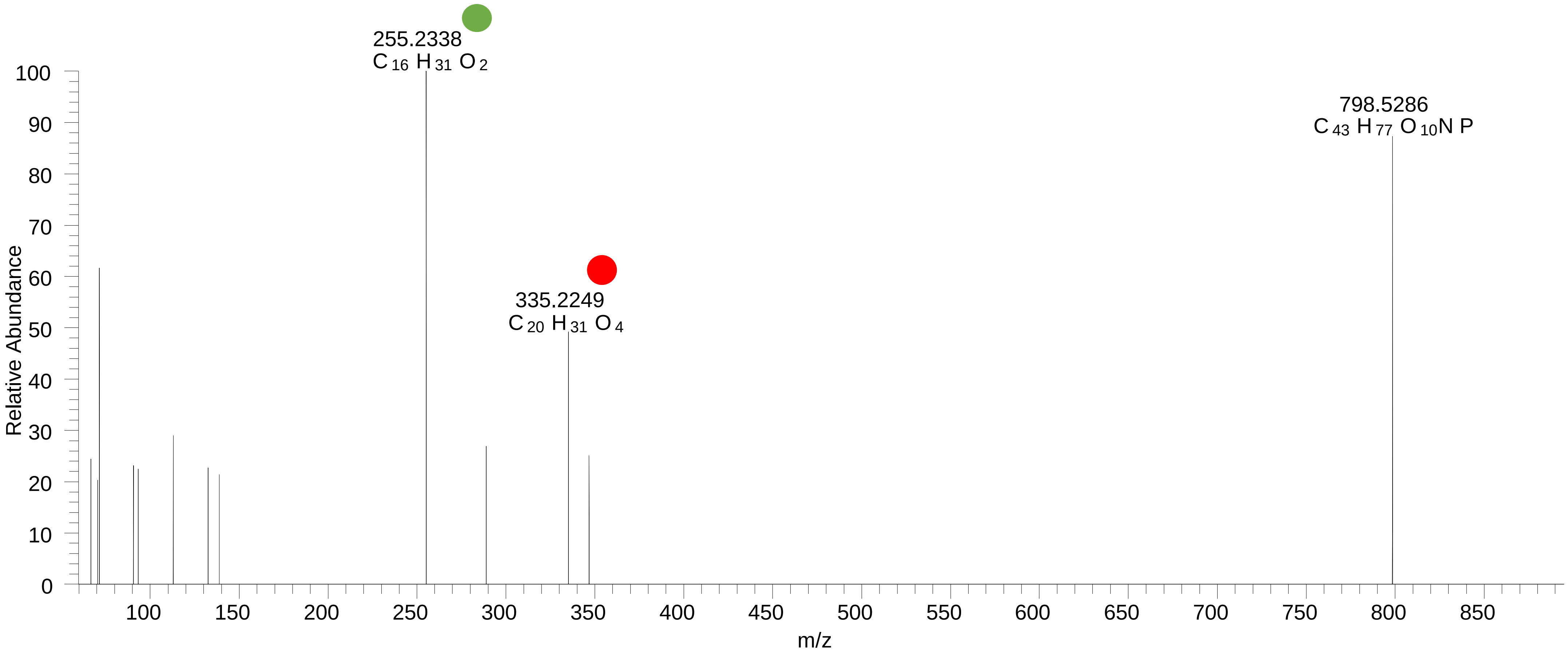

PC(16:0_20:4<OOH{15}>)
RT 13.6

[oxPC+HCOO]- XIC 858.5502 NL: 9.78E3

- Fragments containing oxFAs
- Fragments related to water loss
- Fragments not containing oxFAs
- Fragments containing methylated oxFAs
- Fragments related to CO_2 loss
- Position-specific fragments

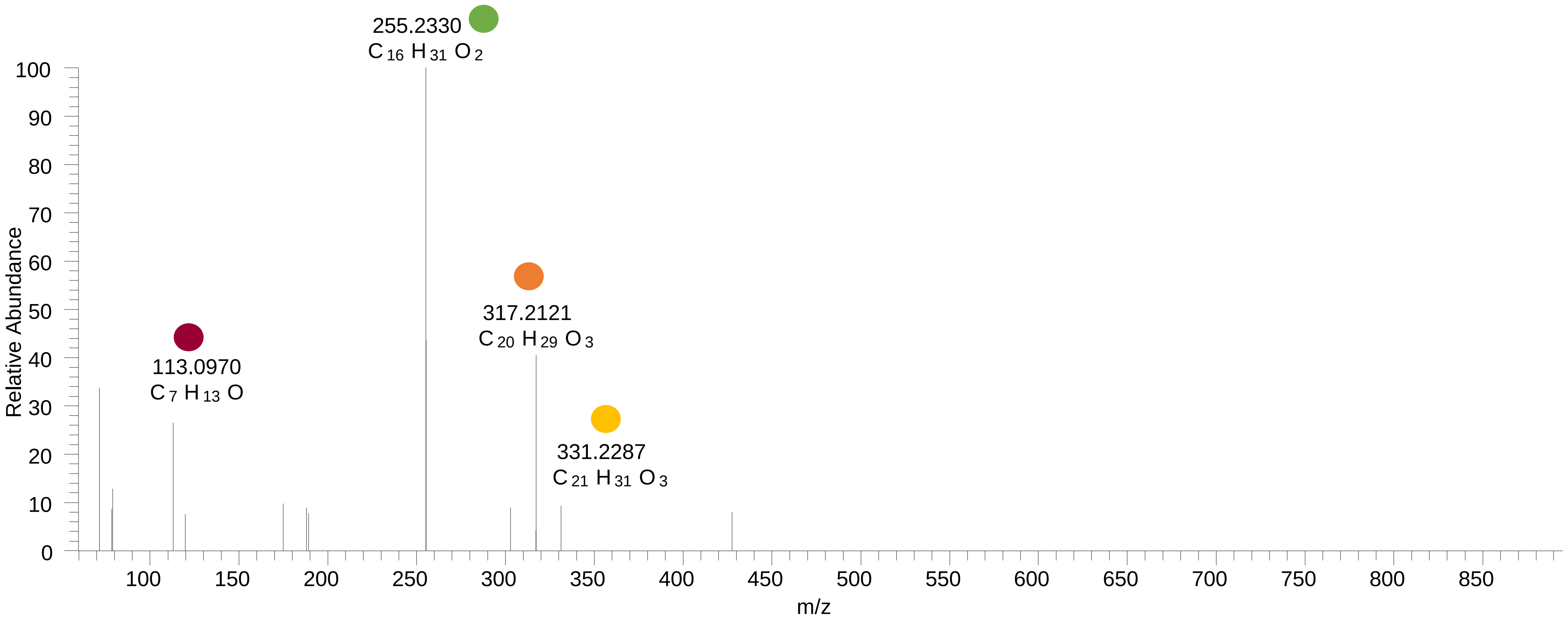

PC(16:0_20:4<OOH{12}>)
RT 13.9

[oxPC+HCOO]- XIC 858.5502 NL: 1.37E4

- Fragments containing oxFAs
- Fragments related to water loss
- Fragments not containing oxFAs
- Fragments containing methylated oxFAs
- Fragments related to CO_2 loss
- Position-specific fragments

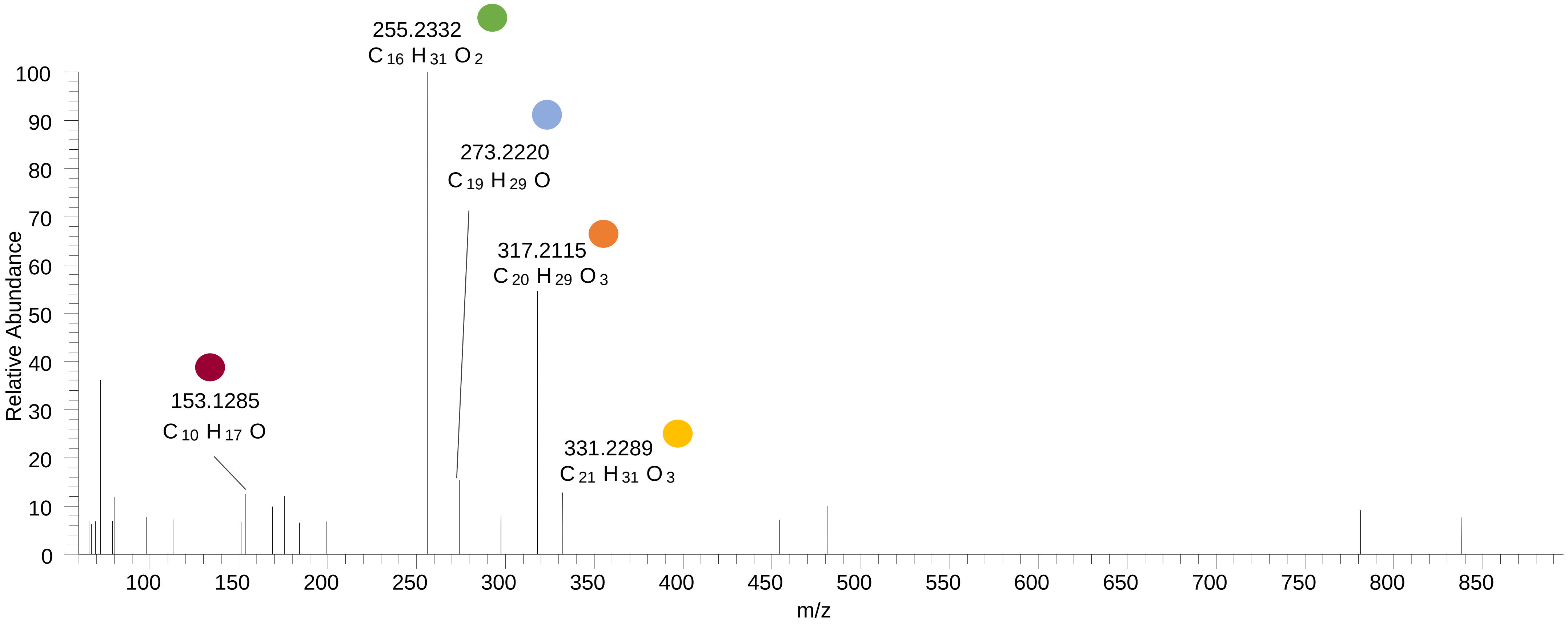

PC(16:0_20:4<OOH{7}>)
RT 14.4

[oxPC+HCOO]- XIC 858.5502 NL: 1.06E4

- Fragments containing oxFAs
- Fragments related to water loss
- Fragments not containing oxFAs
- Fragments containing methylated oxFAs
- Fragments related to CO_2 loss
- Position-specific fragments

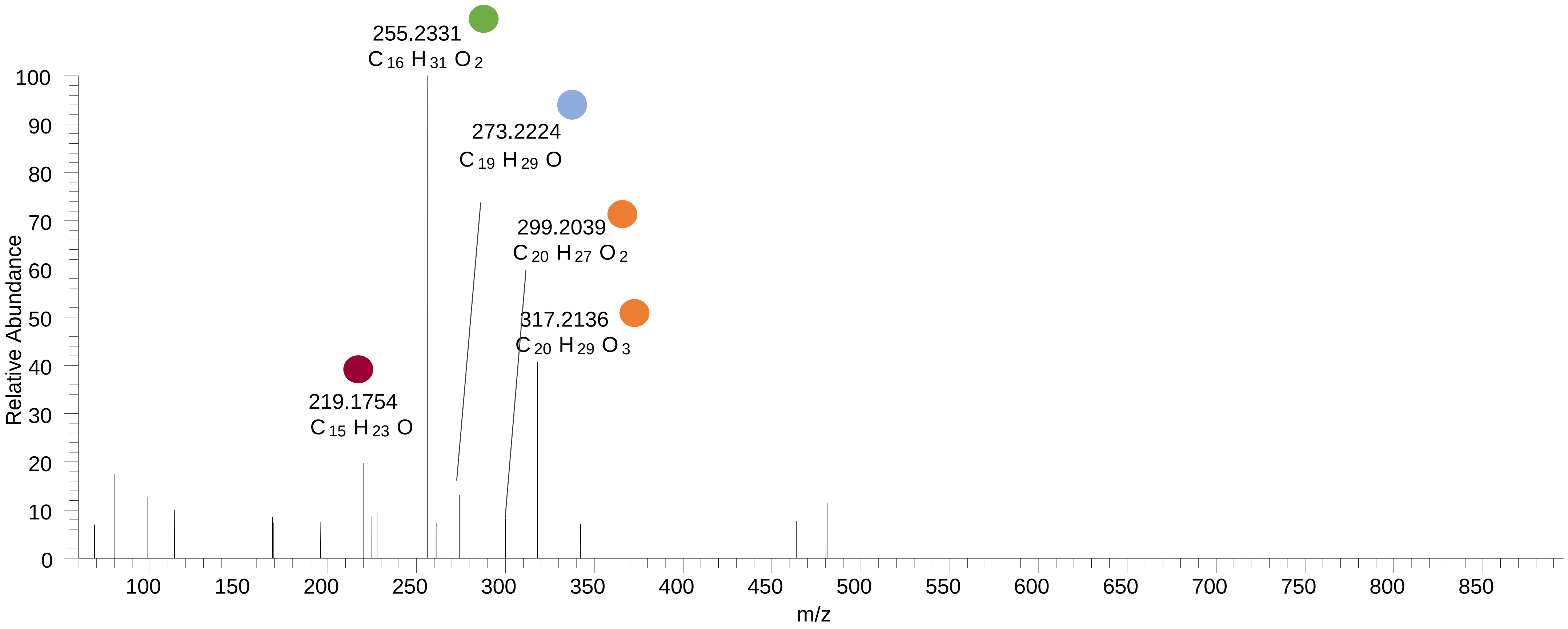

PC(18:1_18:2<OOH>)
PC(16:0_20:3<OOH>)
RT 14.1

[oxPC+HCOO]- XIC 860.5658 NL: 7.47E3

- Fragments containing oxFAs
- Fragments related to water loss
- Fragments not containing oxFAs
- Fragments containing methylated oxFAs
- Fragments related to CO_2 loss
- Position-specific fragments

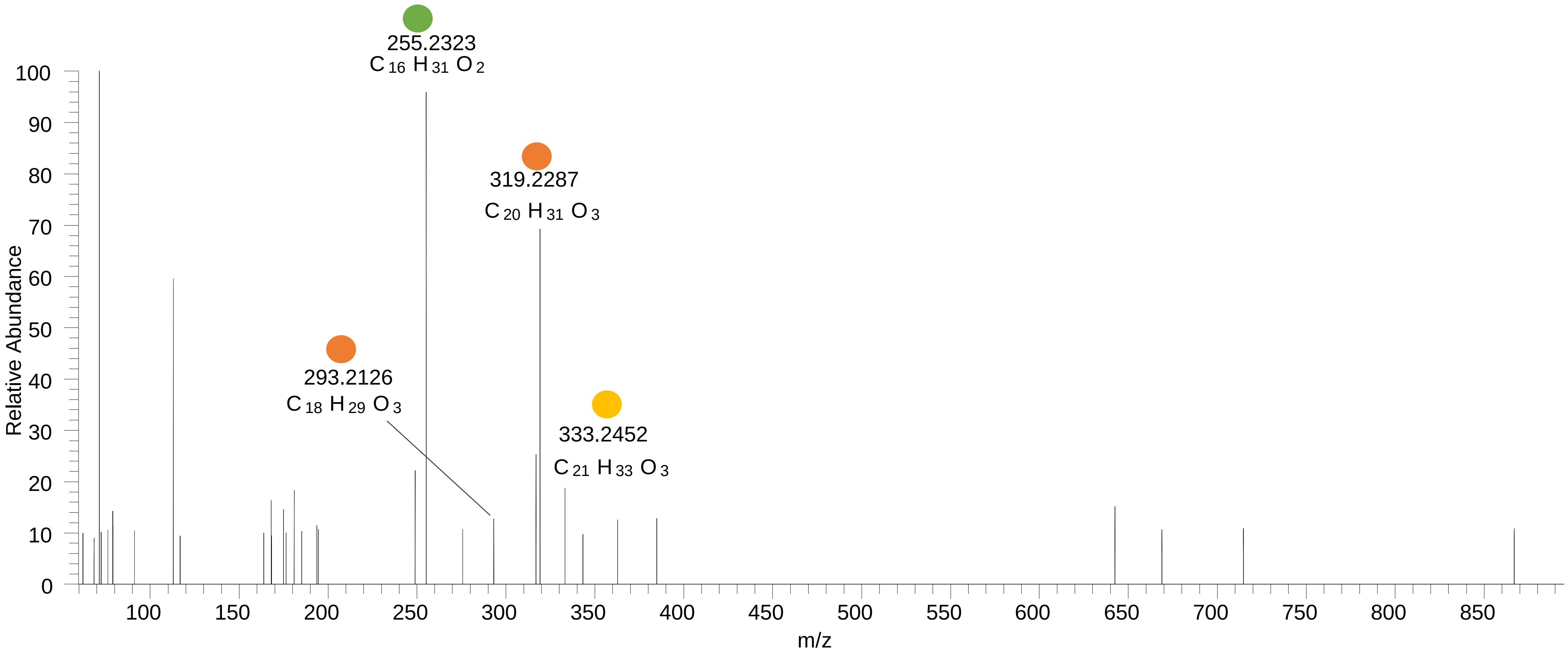

PC(16:0_20:3<OOH>)
RT 14.2

[oxPC+HCOO]- XIC 860.5658 NL: 8.89E3

Fragments containing oxFAs

Fragments related to water loss

Fragments not containing oxFAs

Fragments containing methylated oxFAs

Fragments related to CO_2 loss

Position-specific fragments

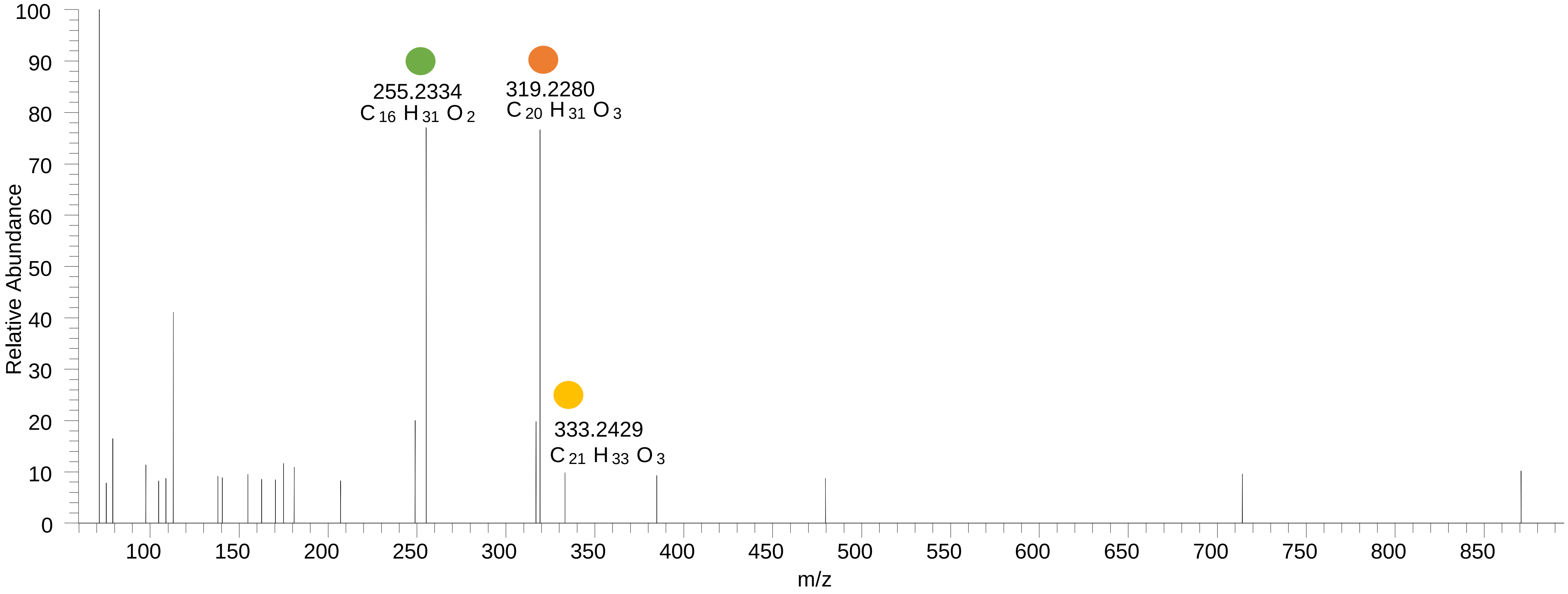

PC(18:0_18:2<2O>)
RT 13.6

[oxPC+HCOO]- XIC 862.5815 NL: 1.57E3

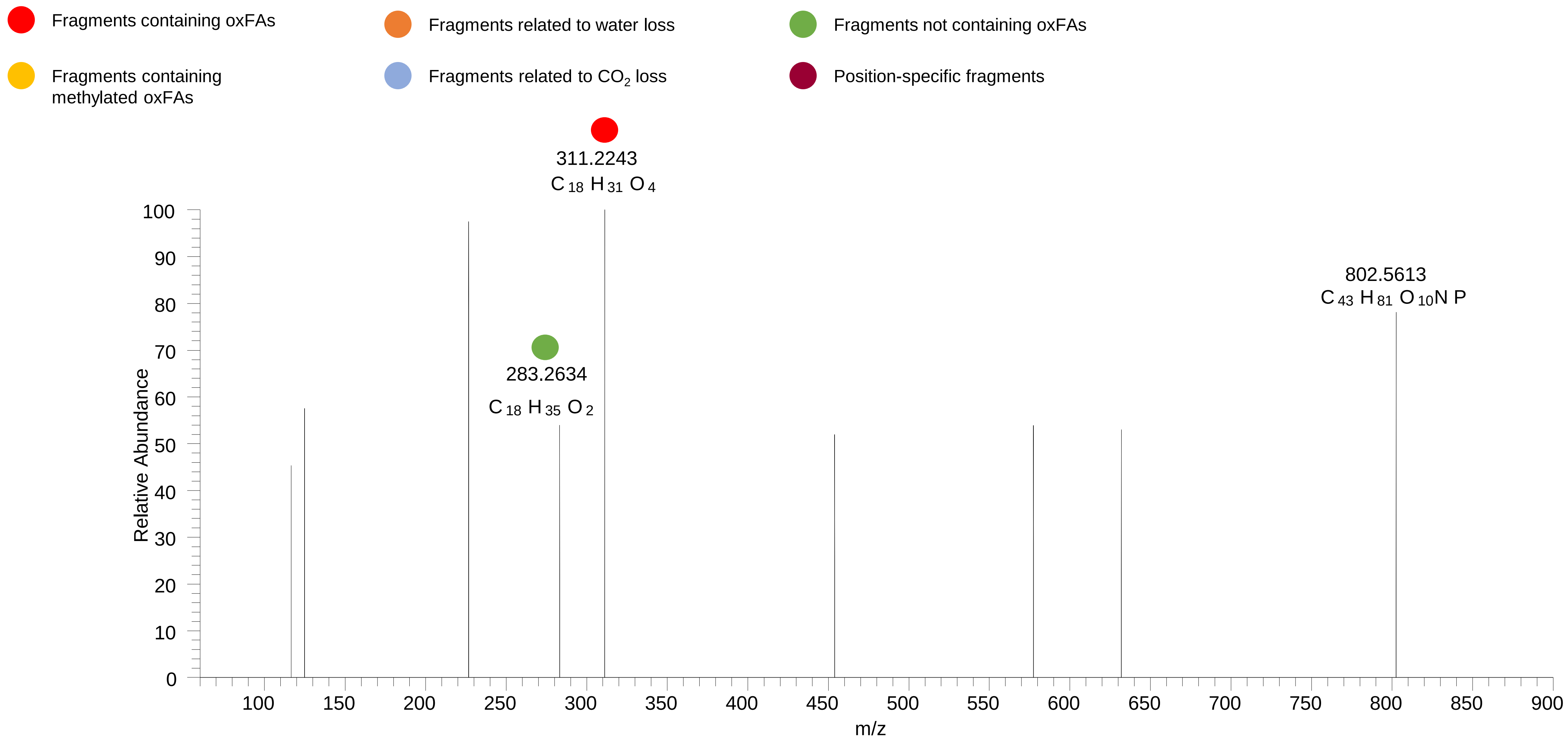

PC(18:0_18:2<OOH>)
RT 14.5

[oxPC+HCOO]- XIC 862.5815 NL: 2.32E3

Fragments containing oxFAs

Fragments related to water loss

Fragments not containing oxFAs

Fragments containing methylated oxFAs

Fragments related to CO_2 loss

Position-specific fragments

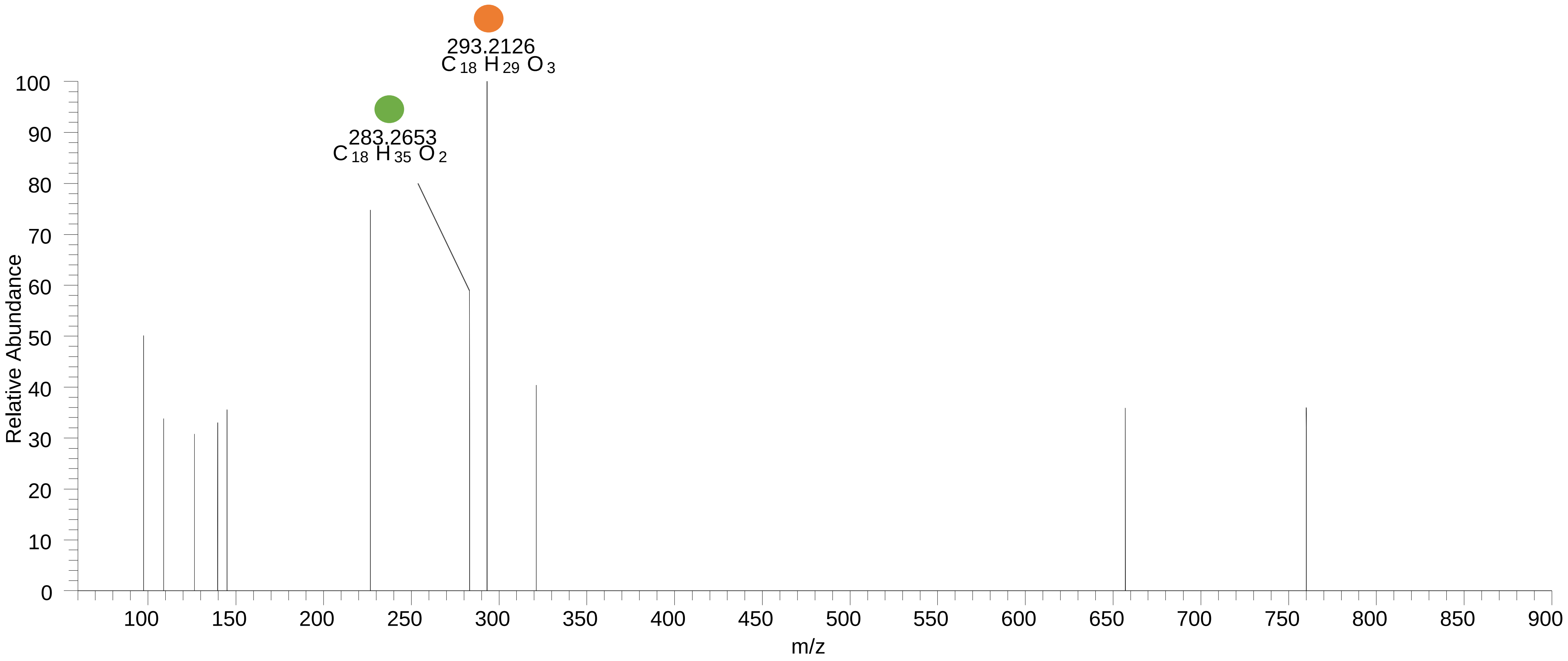

PC(18:0_18:2<OOH{13}>)
RT 15.0

[oxPC+HCOO]- XIC 862.5815 NL: 2.15E4

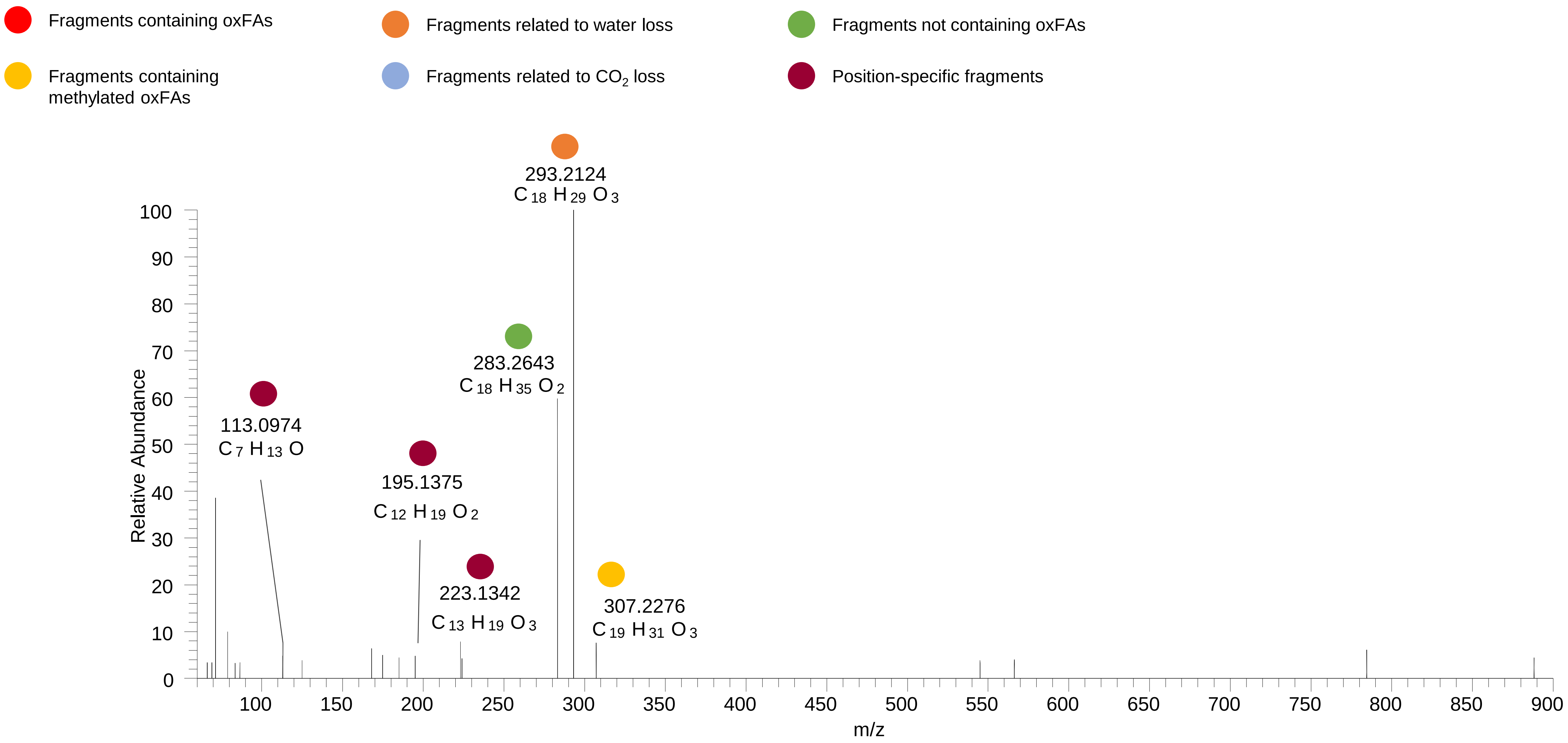

PC(18:0_18:2<OOH{9}>)
RT 15.1

[oxPC+HCOO]- XIC 862.5815 NL: 2.30E4

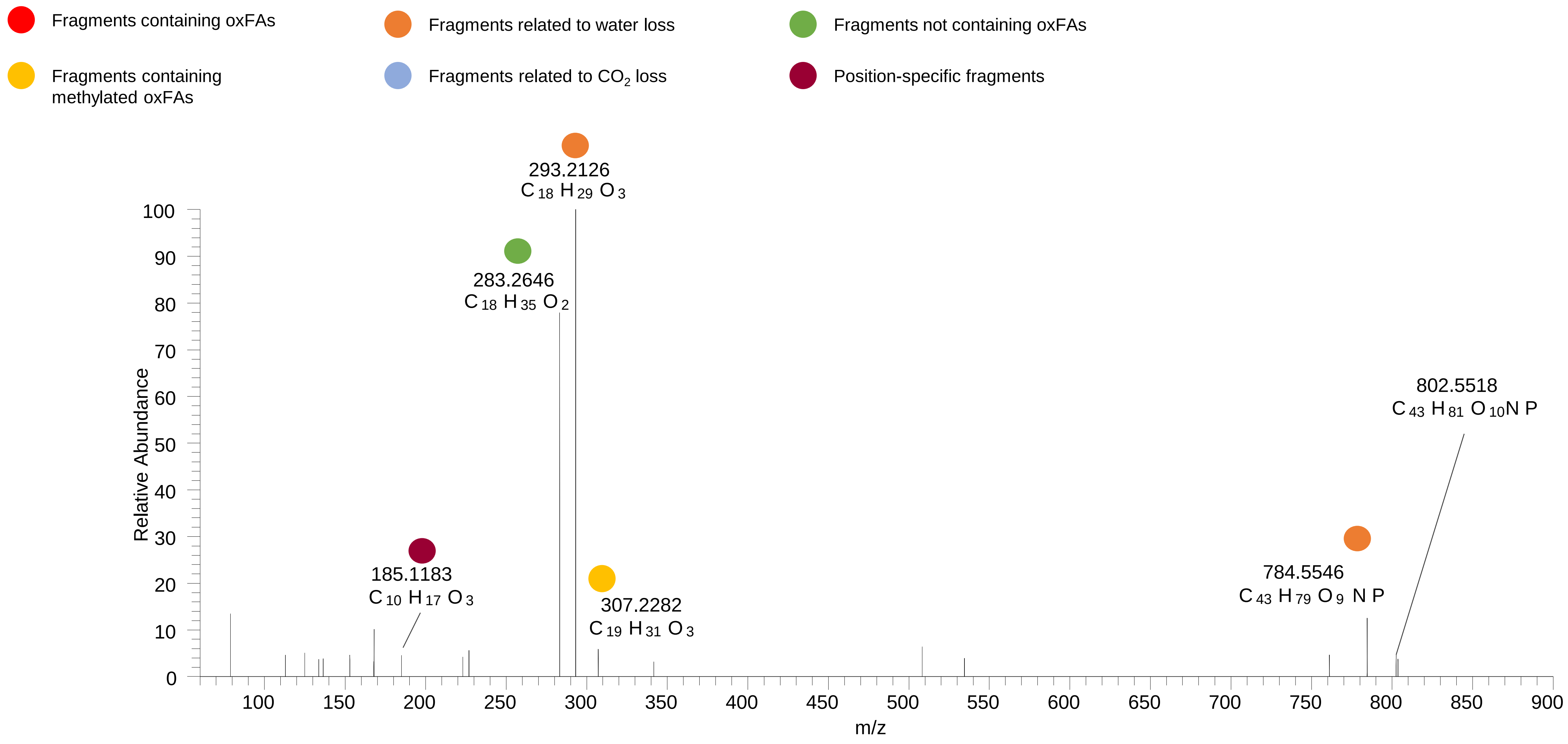

PC(18:0_18:2<OOH>)
RT 15.5

[oxPC+HCOO]- XIC 862.5815 NL: 4.35E3

- Fragments containing oxFAs
- Fragments related to water loss
- Fragments not containing oxFAs
- Fragments containing methylated oxFAs
- Fragments related to CO_2 loss
- Position-specific fragments

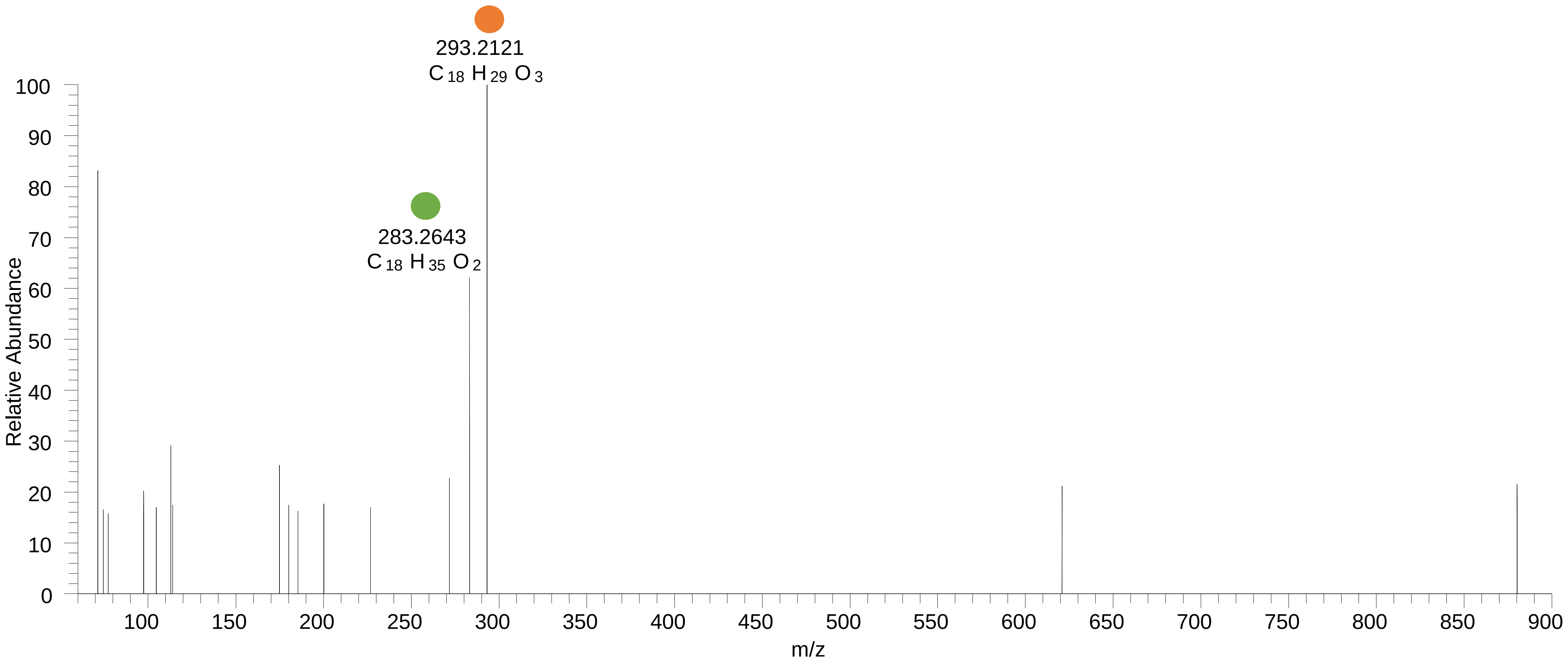

PC(16:0_22:6<O>)
RT 13.1

[oxPC+HCOO]- XIC 866.5553 NL: 5.94E3

- Fragments containing oxFAs
- Fragments related to water loss
- Fragments not containing oxFAs
- Fragments containing methylated oxFAs
- Fragments related to CO_2 loss
- Position-specific fragments

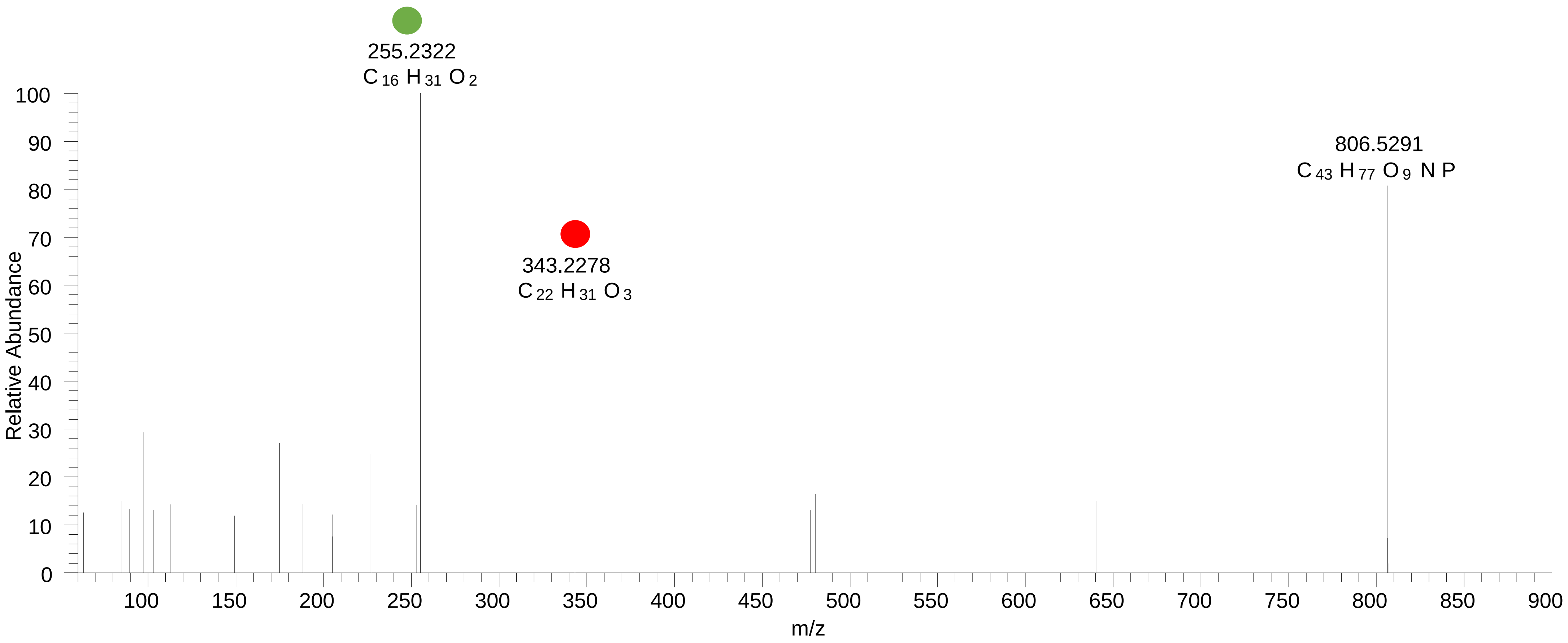

PC(16:0_22:6<OH{16}>)
RT 13.4

[oxPC+HCOO]- XIC 866.5553 NL: 1.09E4

- Fragments containing oxFAs
- Fragments related to water loss
- Fragments not containing oxFAs
- Fragments containing methylated oxFAs
- Fragments related to CO_2 loss
- Position-specific fragments

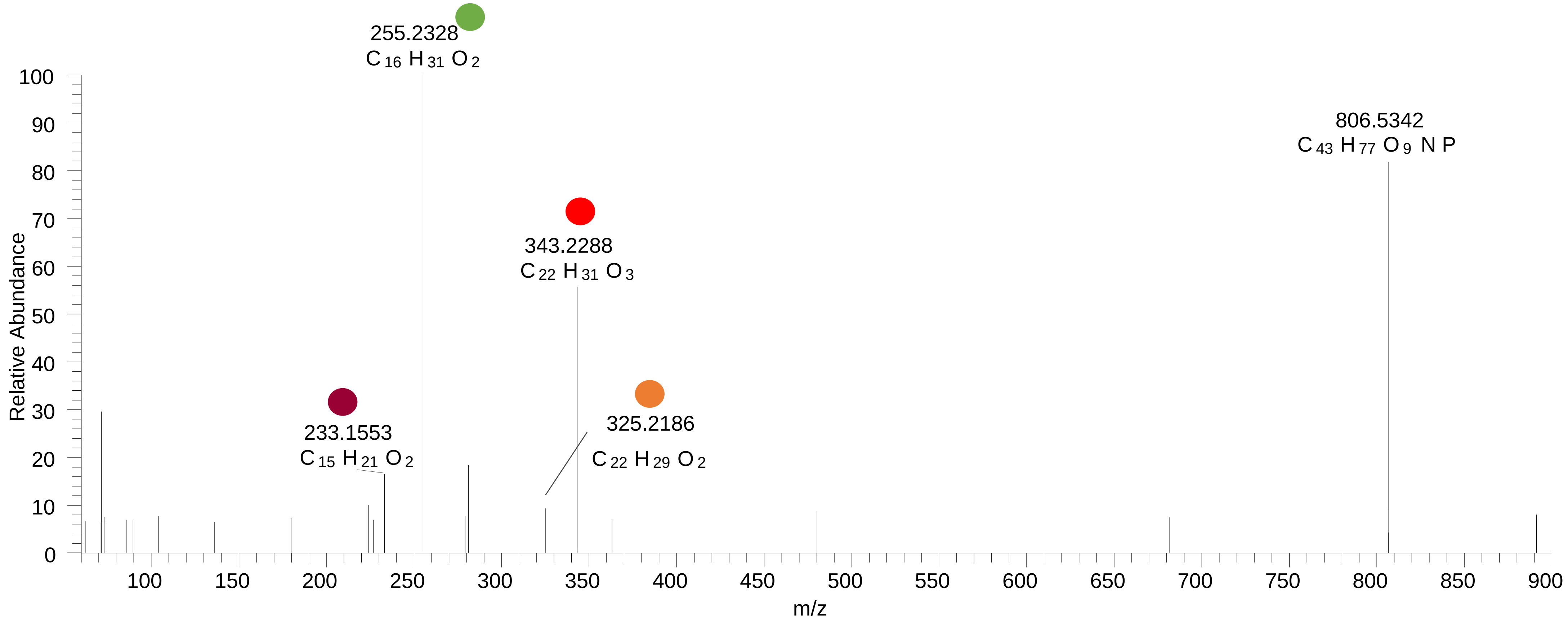

PC(16:0_22:6<O>)
RT 14.4

[oxPC+HCOO]- XIC 866.5553 NL: 1.00E4

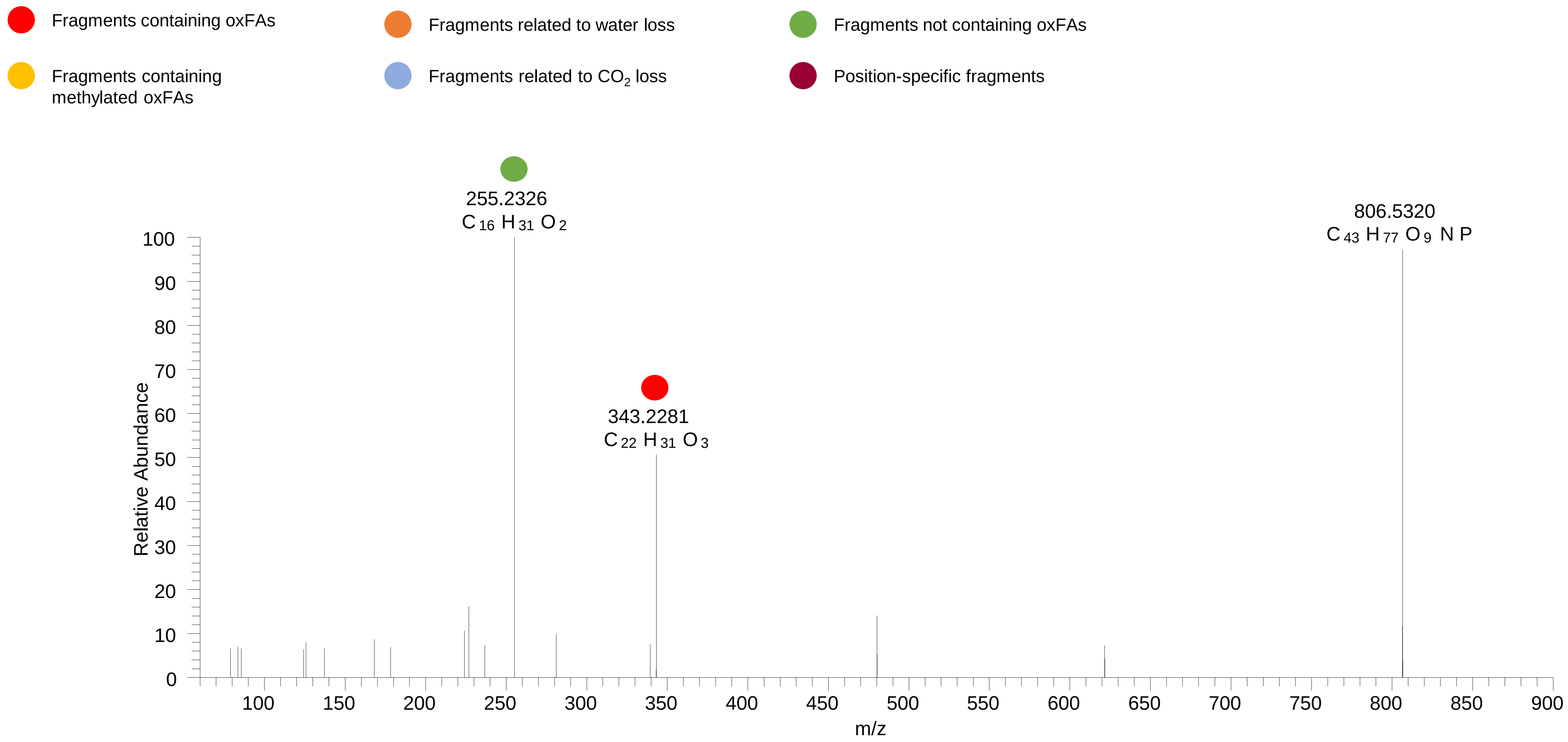

PC(16:0_22:5<OH>)
PC(18:1_20:4<O>)
RT 13.7 -14.6

[oxPC+HCOO]- XIC 868.5709 NL: 4.11E3

- Fragments containing oxFAs
- Fragments related to water loss
- Fragments not containing oxFAs
- Fragments containing methylated oxFAs
- Fragments related to CO_2 loss
- Position-specific fragments

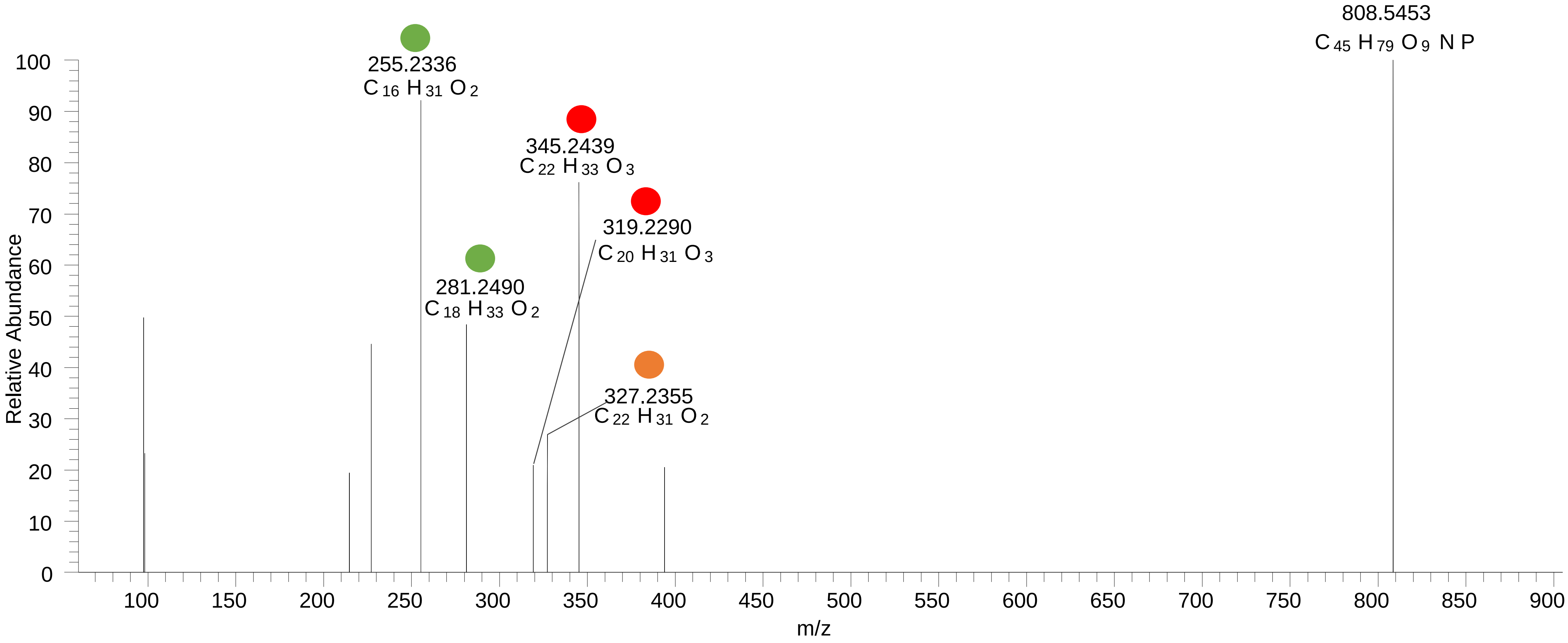

PC(18:1_20:4<O>)
RT 14.9

[oxPC+HCOO]- XIC 868.5709 NL: 4.06E3

Fragments containing oxFAs

Fragments related to water loss

Fragments not containing oxFAs

Fragments containing methylated oxFAs

Fragments related to CO_2 loss

Position-specific fragments

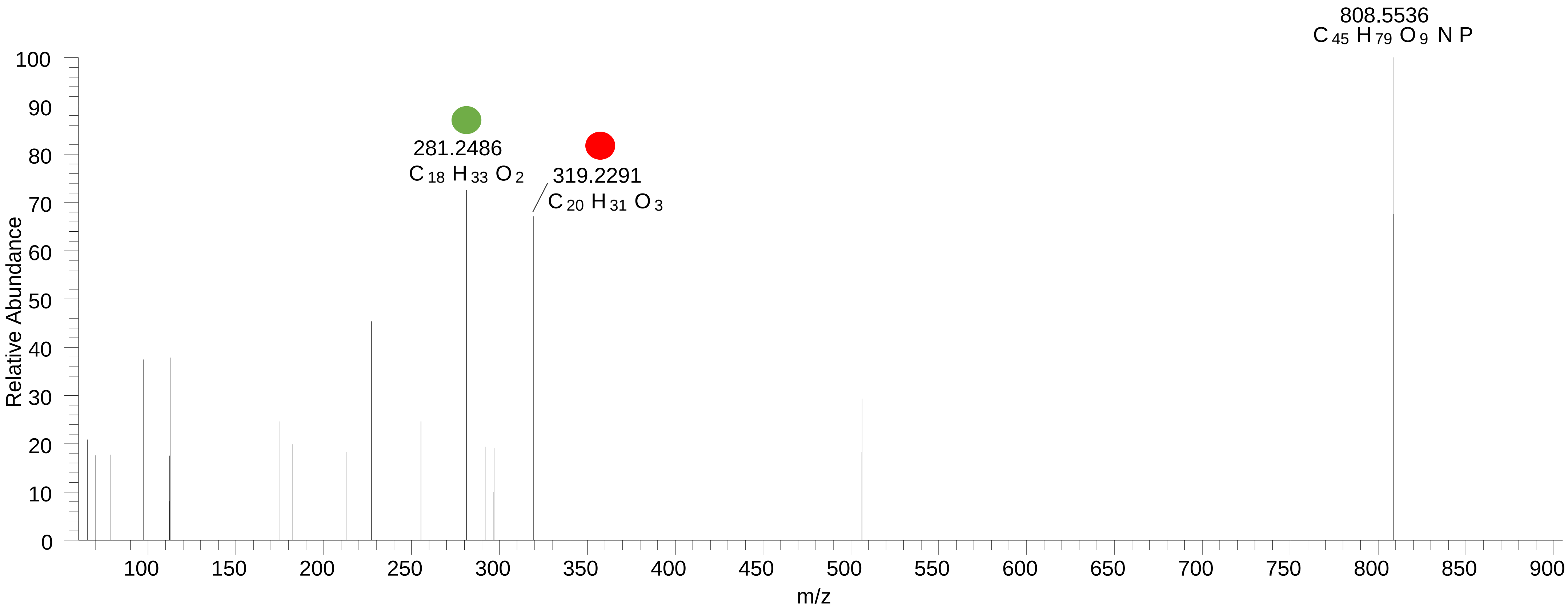

PC(18:1_20:3<O>)
RT 14.5

[oxPC+HCOO]- XIC 870.5866 NL: 3.87E3

Fragments containing oxFAs

Fragments related to water loss

Fragments not containing oxFAs

Fragments containing methylated oxFAs

Fragments related to CO_2 loss

Position-specific fragments

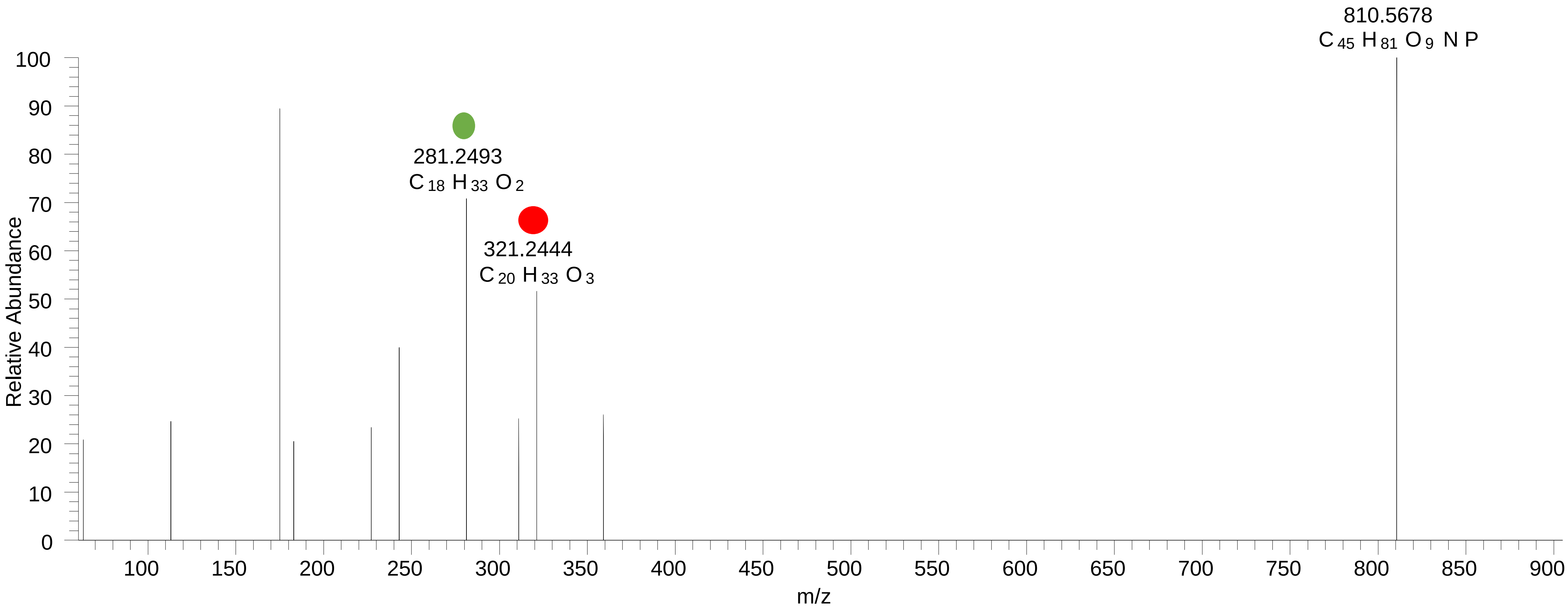

PC(18:0_20:4<OH{15}>)
RT 15.3

[oxPC+HCOO]- XIC 870.5866 NL: 8.43E3

- Fragments containing oxFAs
- Fragments related to water loss
- Fragments not containing oxFAs
- Fragments containing methylated oxFAs
- Fragments related to CO_2 loss
- Position-specific fragments

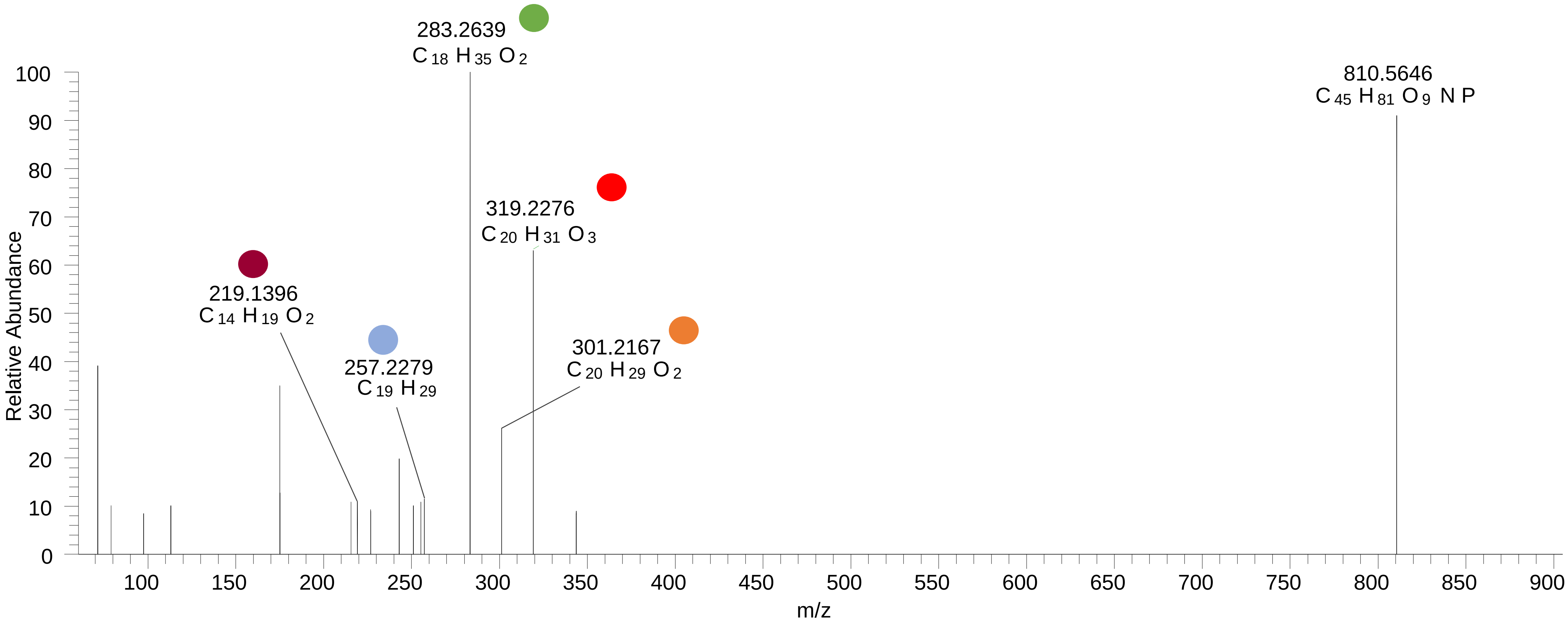

PC(18:0_20:4<OH{11}>)
RT 15.7

[oxPC+HCOO]- XIC 870.5866 NL: 1.46E4

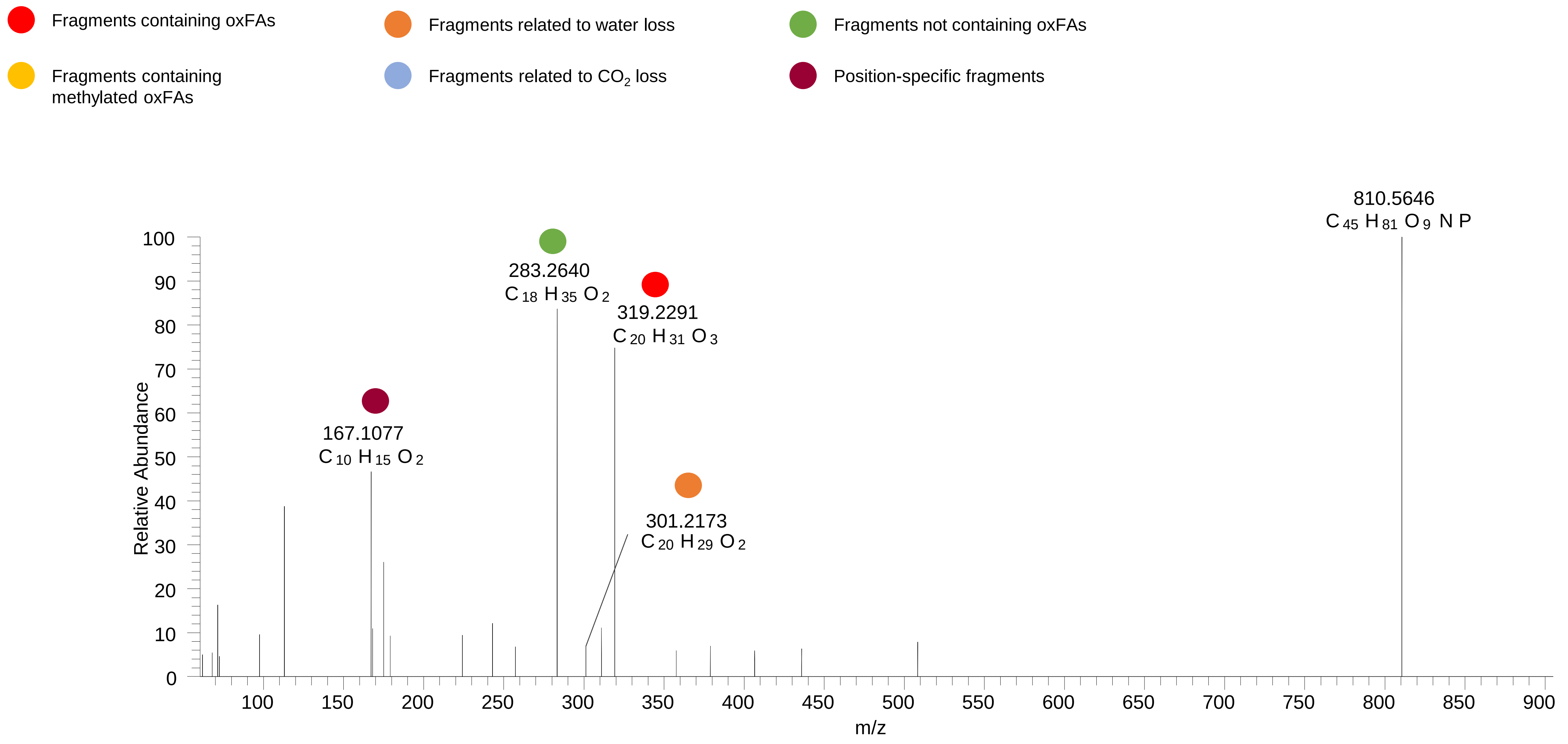

PC(18:0_20:4<OH{12}>)
PC(18:0_20:4<OH{9}>)
PC(18:0_20:4<OH{8}>)
RT 16.2

[oxPC+HCOO]- XIC 870.5866 NL: 1.81E4

- Fragments containing oxFAs
- Fragments related to water loss
- Fragments not containing oxFAs
- Fragments containing methylated oxFAs
- Fragments related to CO_2 loss
- Position-specific fragments

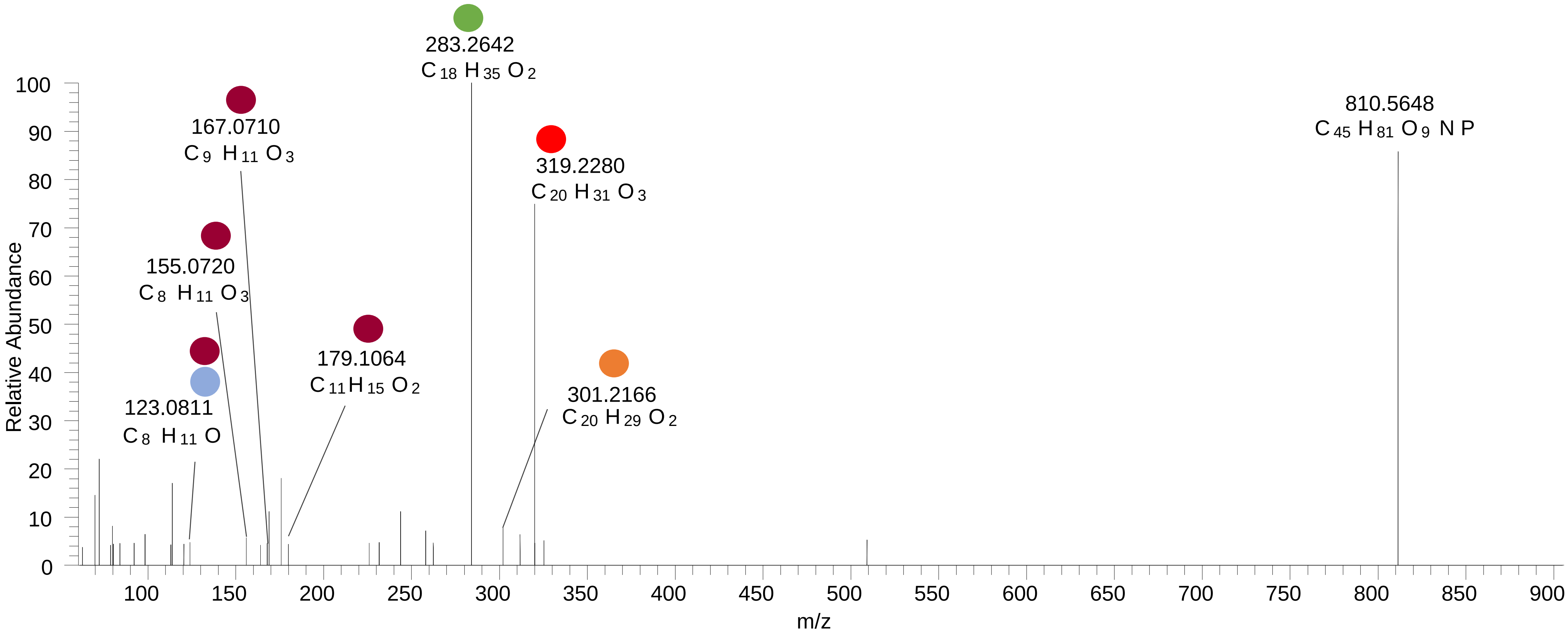

PC(18:0_20:4<O>)
RT 16.7

[oxPC+HCOO]- XIC 870.5866 NL: 3.02E3

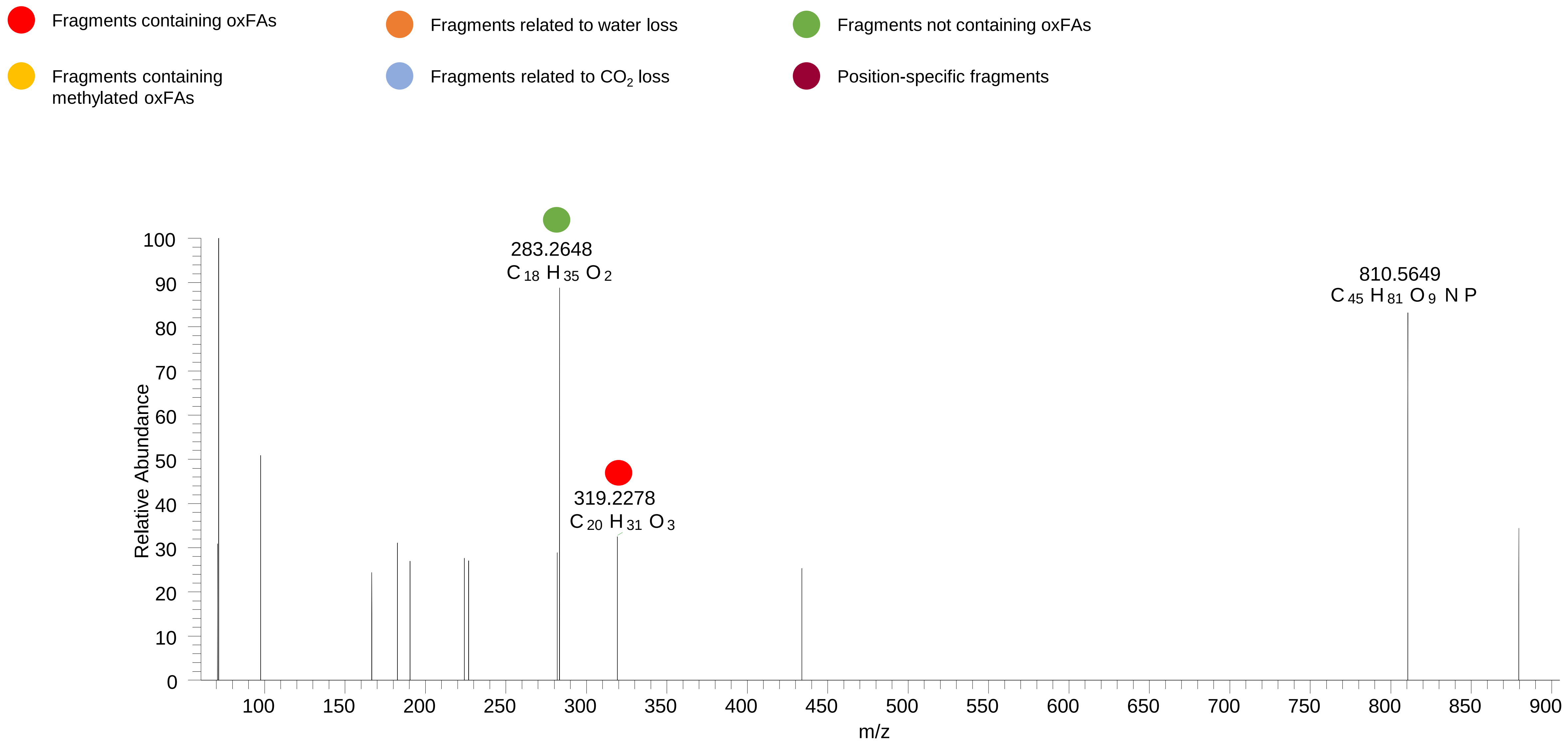

PC(16:0_22:6<2O>)
RT 10.9-13.4

[oxPC+HCOO]- XIC 882.5502 NL: 3.04E3

- Fragments containing oxFAs
- Fragments related to water loss
- Fragments not containing oxFAs
- Fragments containing methylated oxFAs
- Fragments related to CO_2 loss
- Position-specific fragments

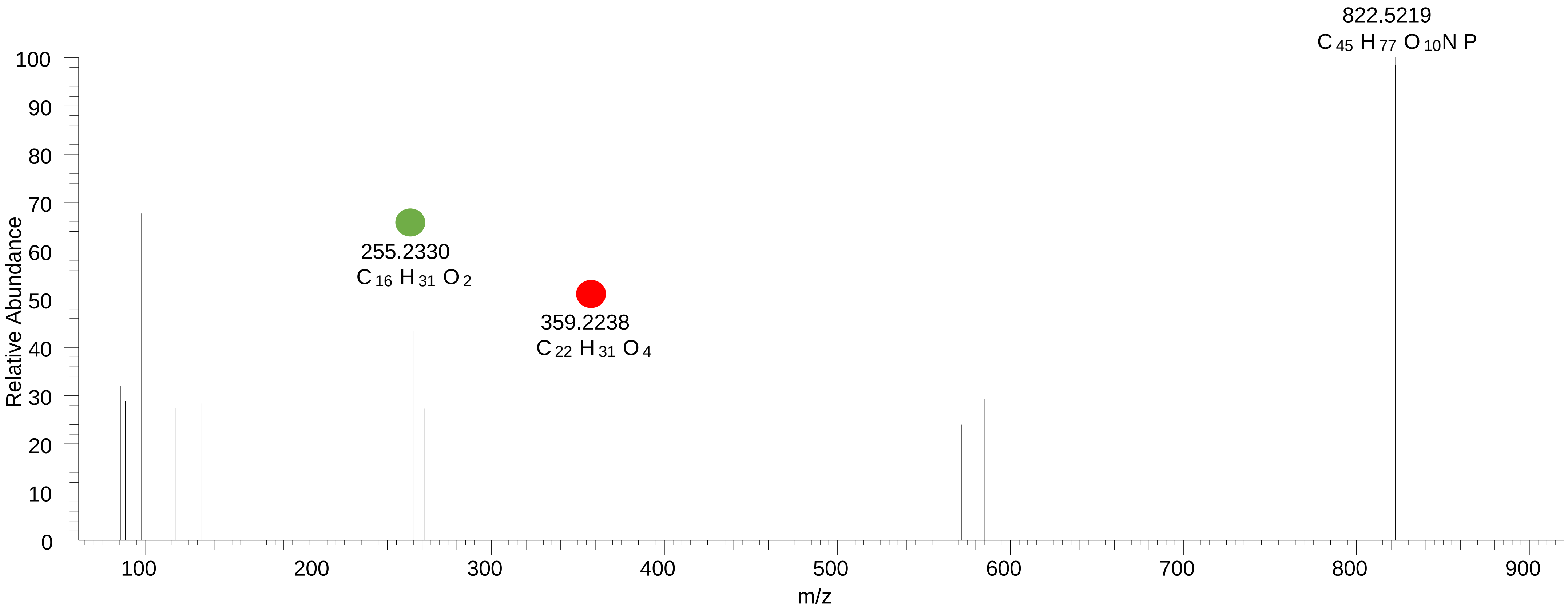

PC(16:0_22:5<OOH>)
RT 12.4

[oxPC+HCOO]- XIC 884.5658 NL: 1.89E3

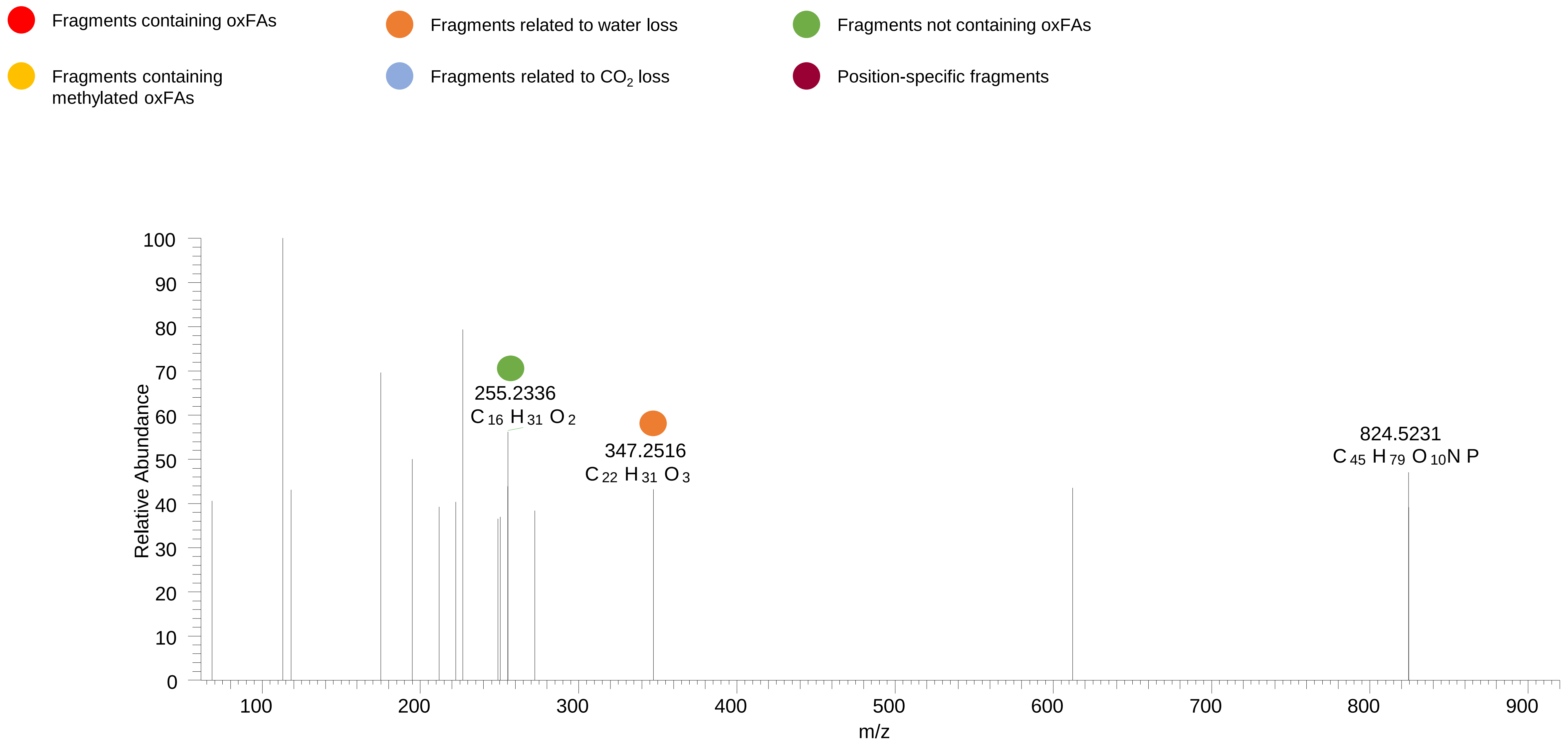

PC(18:0_20:4<2O>)
RT 13.0

[oxPC+HCOO]- XIC 886.5815 NL: 6.57E3

- Fragments containing oxFAs
- Fragments related to water loss
- Fragments not containing oxFAs
- Fragments containing methylated oxFAs
- Fragments related to CO_2 loss
- Position-specific fragments

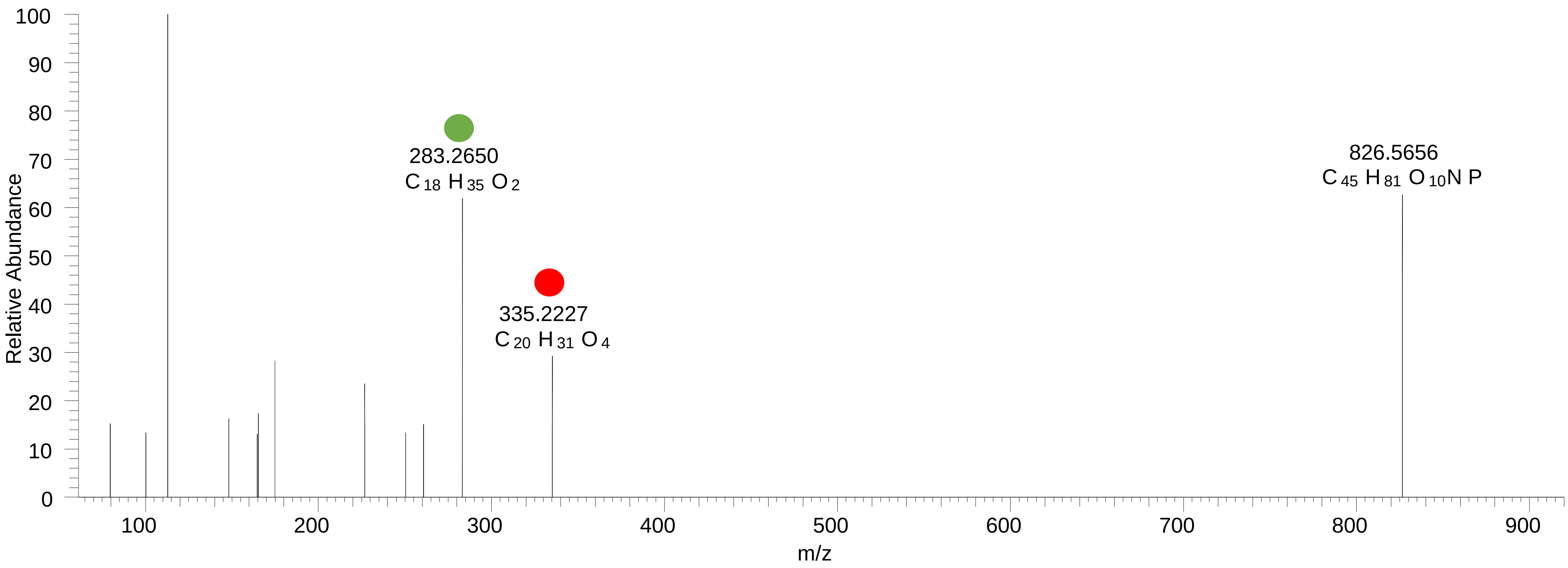

PC(18:0_20:4<OOH>)
RT 14.4 -15.3

[oxPC+HCOO]- XIC 886.5815 NL: 4.08E3

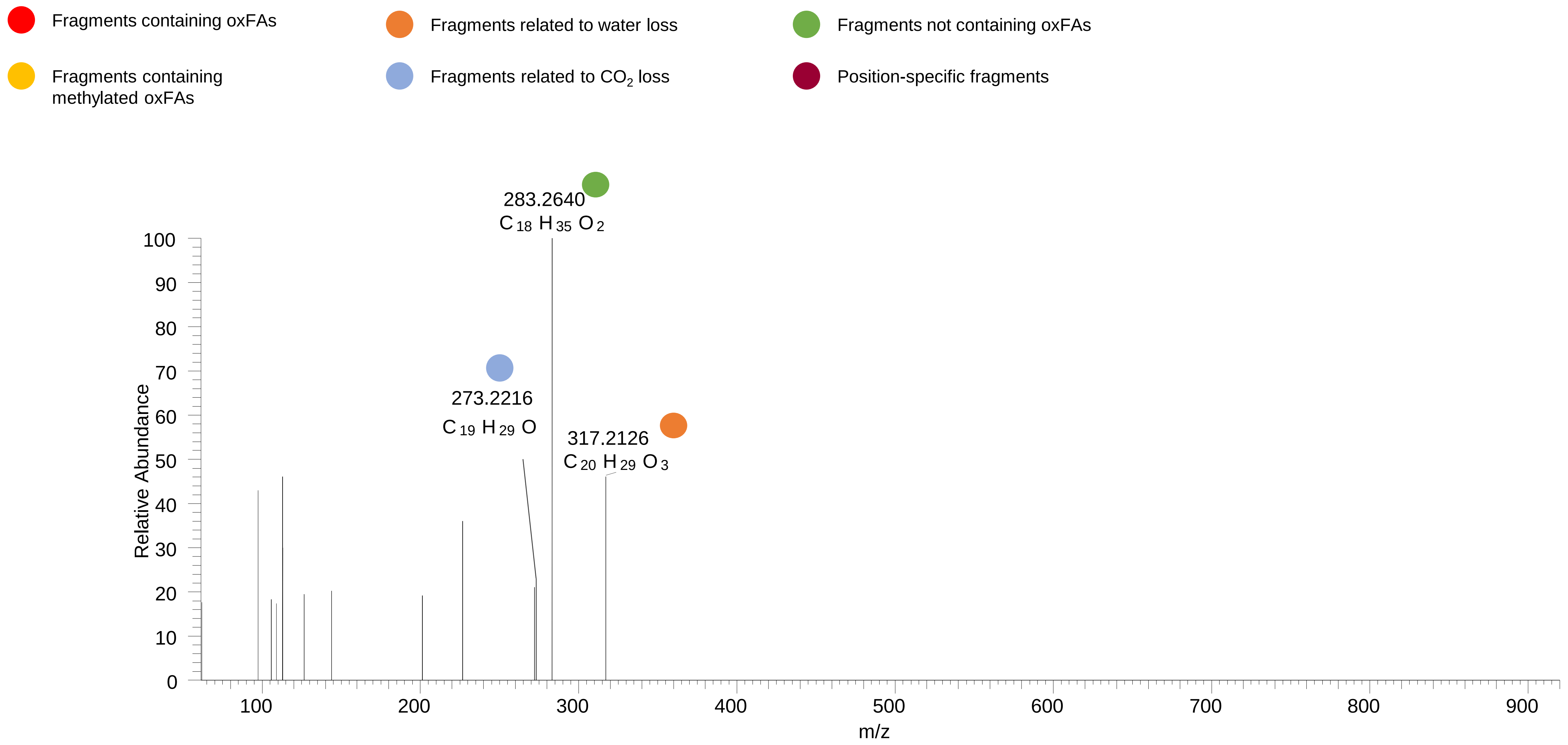

PC(18:0_20:4<OOH{7}>)
RT 16.0

[oxPC+HCOO]- XIC 886.5815 NL: 7.05E3

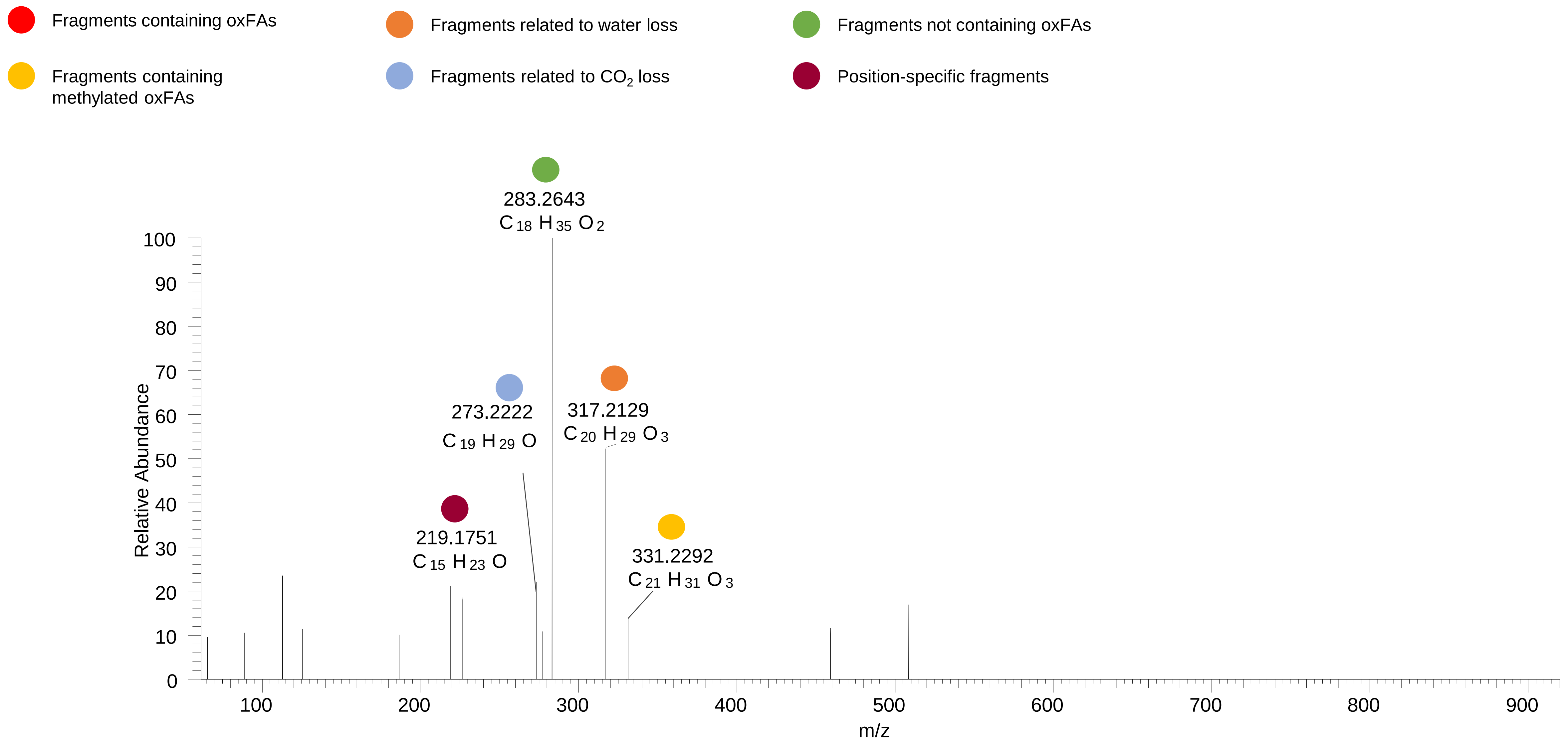

PC(18:0_20:4<OOH>)
RT 16.4

[oxPC+HCOO]- XIC 886.5815 NL: 3.43E3

Fragments containing oxFAs

Fragments related to water loss

Fragments not containing oxFAs

Fragments containing methylated oxFAs

Fragments related to CO_2 loss

Position-specific fragments

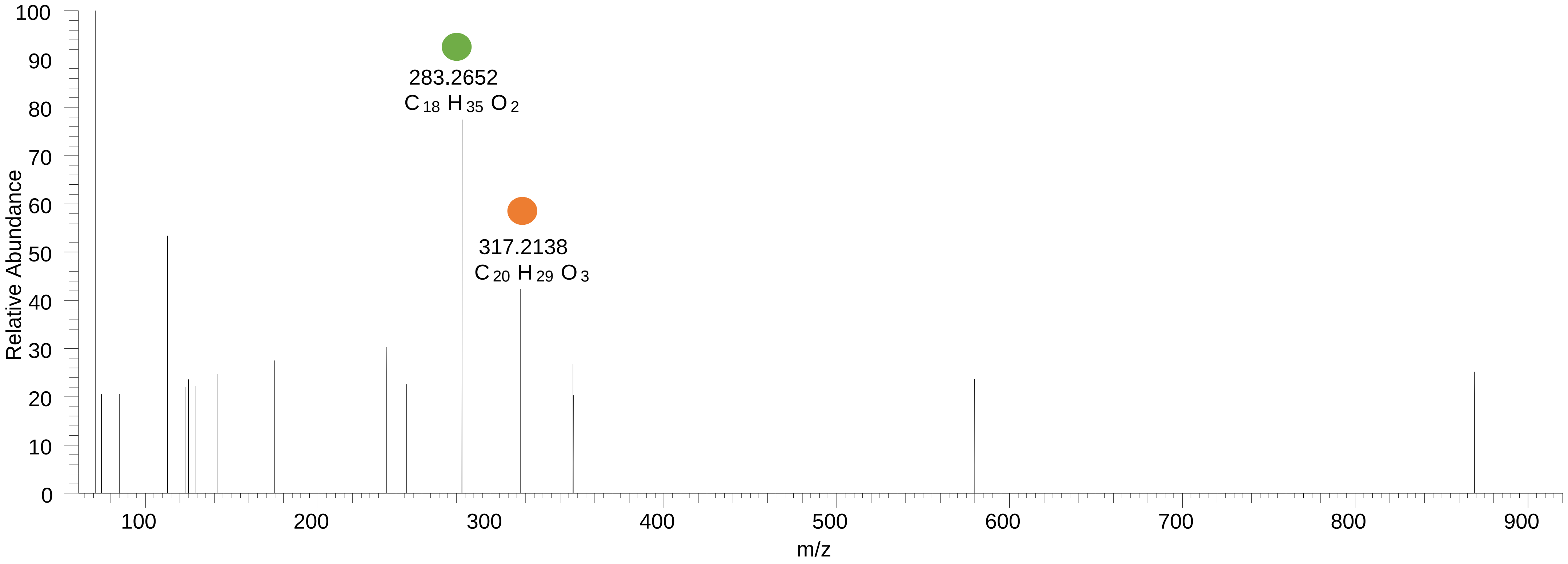

PC(18:0_20:3<OOH>)
RT 15.8

[oxPC+HCOO]- XIC 888.5971 NL: 3.28E3

- Fragments containing oxFAs
- Fragments related to water loss
- Fragments not containing oxFAs
- Fragments containing methylated oxFAs
- Fragments related to CO_2 loss
- Position-specific fragments

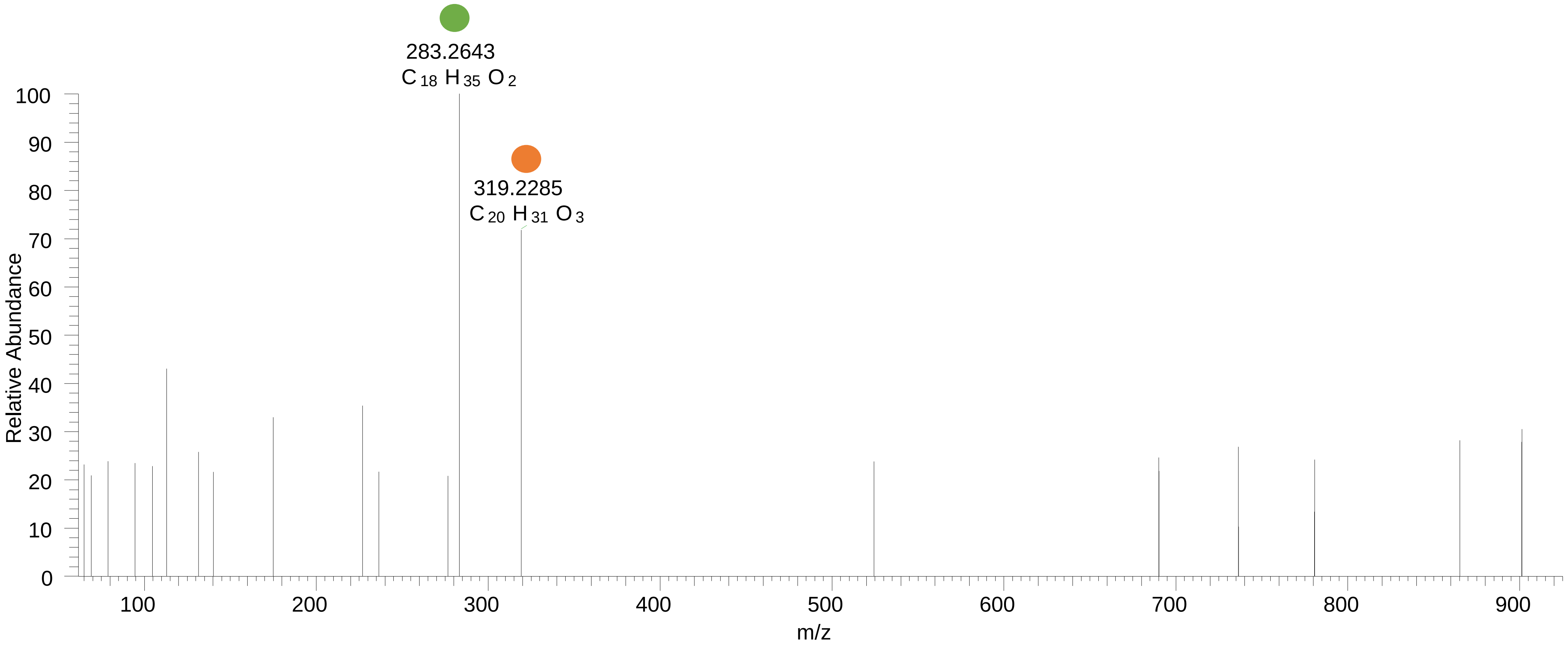

PC(18:0_22:6<O>)
RT 15.1-15.8

[oxPC+HCOO]- XIC 894.5866 NL: 2.88E3

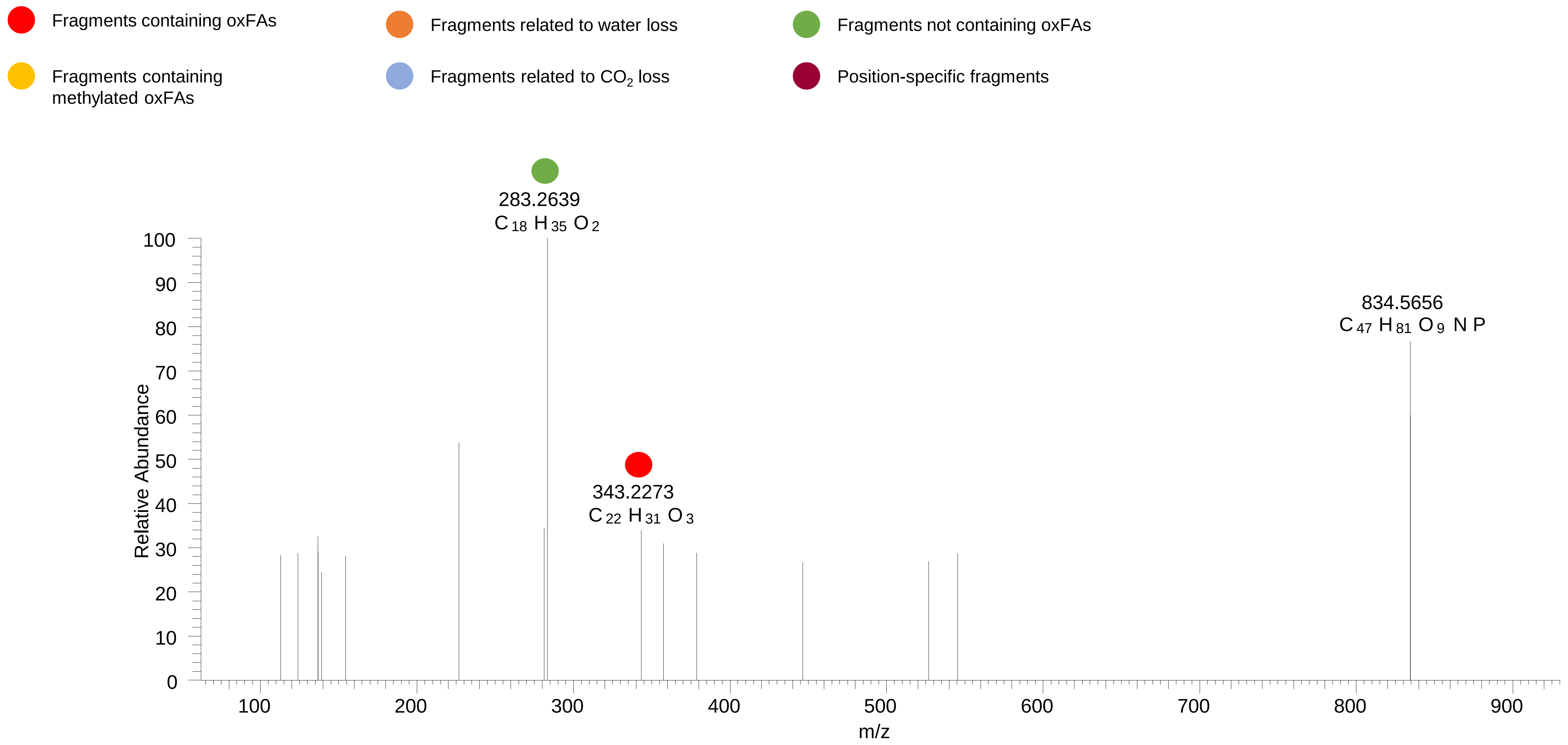

Supplement: Supplementary file 9 — Supplementary Data 6 [file 41467_2022_33225_MOESM9_ESM.pdf]
