# Supplementary material for: Analytical and computational workflow for in-depth analysis of oxidized complex lipids in blood plasma
Source: Nat Commun. 2022 Nov 1;13:6547. doi: 10.1038/s41467-022-33225-9 (PMC9626469; doi:10.1038/s41467-022-33225-9)
Supplement: Supplementary file 15 — Reporting Summary [file 41467_2022_33225_MOESM15_ESM.pdf]

nature portfolio

Corresponding author(s): Maria Fedorova

Last updated by author(s): Aug 10, 2022

Reporting Summary

Nature Portfolio wishes to improve the reproducibility of the work that we publish. This form provides structure for consistency and transparency in reporting. For further information on Nature Portfolio policies, see our Editorial Policies and the Editorial Policy Checklist.

Statistics

For all statistical analyses, confirm that the following items are present in the figure legend, table legend, main text, or Methods section.

n/a	Confirmed	
☐	☒	The exact sample size (*n*) for each experimental group/condition, given as a discrete number and unit of measurement
☐	☒	A statement on whether measurements were taken from distinct samples or whether the same sample was measured repeatedly
☐	☒	The statistical test(s) used AND whether they are one- or two-sided<br>*Only common tests should be described solely by name; describe more complex techniques in the Methods section.*
☒	☐	A description of all covariates tested
☐	☒	A description of any assumptions or corrections, such as tests of normality and adjustment for multiple comparisons
☐	☒	A full description of the statistical parameters including central tendency (e.g. means) or other basic estimates (e.g. regression coefficient) AND variation (e.g. standard deviation) or associated estimates of uncertainty (e.g. confidence intervals)
☒	☐	For null hypothesis testing, the test statistic (e.g. *F*, *t*, *r*) with confidence intervals, effect sizes, degrees of freedom and *P* value noted<br>*Give P values as exact values whenever suitable.*
☒	☐	For Bayesian analysis, information on the choice of priors and Markov chain Monte Carlo settings
☒	☐	For hierarchical and complex designs, identification of the appropriate level for tests and full reporting of outcomes
☒	☐	Estimates of effect sizes (e.g. Cohen's *d*, Pearson's *r*), indicating how they were calculated

Our web collection on statistics for biologists contains articles on many of the points above.

Software and code

Policy information about availability of computer code

Data collection	LC-MS/MS raw data is collected using Xcalibur v.4.2.47 (ThermoFisher Scientific Inc)
Data analysis	Lipostar (version 1.0.6, Molecular Discovery, Hertfordshire, UK), Skyline v.21.1.0.146 (MacCoss Lab), LPPtiger 2.0 (Fedorova Lab; https://github.com/LMAI-TUD/lpptiger2), RT mapping R-scripts (Fedorova Lab; https://github.com/SysMedOs/AdipoAtlasScripts/tree/main/DataVisualization), MetaboAnalyst v.5 (Xia Lab; https://www.metaboanalyst.ca), Genesis v.1.8.1 (Bioinformatics TU-Graz), OriginPro 2019 v. 9.6.0.172 (Academic; OriginLab Corporation)

For manuscripts utilizing custom algorithms or software that are central to the research but not yet described in published literature, software must be made available to editors and reviewers. We strongly encourage code deposition in a community repository (e.g. GitHub). See the Nature Portfolio guidelines for submitting code & software for further information.

Data

Policy information about availability of data

All manuscripts must include a data availability statement. This statement should provide the following information, where applicable:

- Accession codes, unique identifiers, or web links for publicly available datasets
- A description of any restrictions on data availability
- For clinical datasets or third party data, please ensure that the statement adheres to our policy

Data generated during the study (all raw LC-MS/MS files) available in a public repository MassIVE MSV000088608, doi:10.25345/C5SG5C, [https://massive.ucsd.edu/ProteoSAFe/dataset.jsp?accession=MSV000088608]. File names correspond to the following LC-MS/MS datasets (Supplementary Data 11).

Source data are provided with this paper. For lipid identification LIPID MAPS structural database (version December 2017) was used.

Human research participants

Policy information about studies involving human research participants and Sex and Gender in Research.

Reporting on sex and gender	study included blood plasma samples from 94 female and 55 male individuals
Population characteristics	study included blood plasma samples from lean (n=50, BMI < 25kg/m², mean age 44.5), obese (n=50, BMI between 30 and 50, mean age 44), and obese with type II diabetes (n=50, BMI between 30 and 50, mean age 53.9) individuals.
Recruitment	Plasma samples (n=150) were selected from the Leipzig Obesity BioBank that has been approved by the Ethics committee of the University of Leipzig (approval number: 159-12-21052012). All subjects gave written informed consent before contributing samples and data to the biobank.
Ethics oversight	Ethics committee of the University of Leipzig (approval number: 159-12-21052012)

Note that full information on the approval of the study protocol must also be provided in the manuscript.

Field-specific reporting

Please select the one below that is the best fit for your research. If you are not sure, read the appropriate sections before making your selection.

☒ Life sciences ☐ Behavioural & social sciences ☐ Ecological, evolutionary & environmental sciences

For a reference copy of the document with all sections, see nature.com/documents/nr-reporting-summary-flat.pdf

Life sciences study design

All studies must disclose on these points even when the disclosure is negative.

Sample size	samples size was chosen based on the sample availability, considering age and gender distribution
Data exclusions	no data exclusion
Replication	experiments were performed in replicates when applicable; for LC-MS/MS analysis of lipids in blood plasma samples accuracy was measured by relative standard deviation for a given lipid within each experimental group.
Randomization	samples were randomized for targeted analysis
Blinding	method validation experiments were performed without blinding using 3 group-specific pooled samples;<br>blinding was applied for quantification of unoxidized and oxidized lipids in individual samples

Reporting for specific materials, systems and methods

We require information from authors about some types of materials, experimental systems and methods used in many studies. Here, indicate whether each material, system or method listed is relevant to your study. If you are not sure if a list item applies to your research, read the appropriate section before selecting a response.

Materials & experimental systems

n/a	Involved in the study
☒	☐ Antibodies
☒	☐ Eukaryotic cell lines
☒	☐ Palaeontology and archaeology
☒	☐ Animals and other organisms
☒	☐ Clinical data
☒	☐ Dual use research of concern

Methods

n/a	Involved in the study
☒	☐ ChIP-seq
☒	☐ Flow cytometry
☒	☐ MRI-based neuroimaging
